# Supplementary figures and images for: ATR limits Rad18-mediated PCNA monoubiquitination to preserve replication fork and telomerase-independent telomere stability (part 1 of 2)
Source: EMBO J. 2024 Mar 11;43(7):9. doi: 10.1038/s44318-024-00066-9 (PMC10987609; doi:10.1038/s44318-024-00066-9)

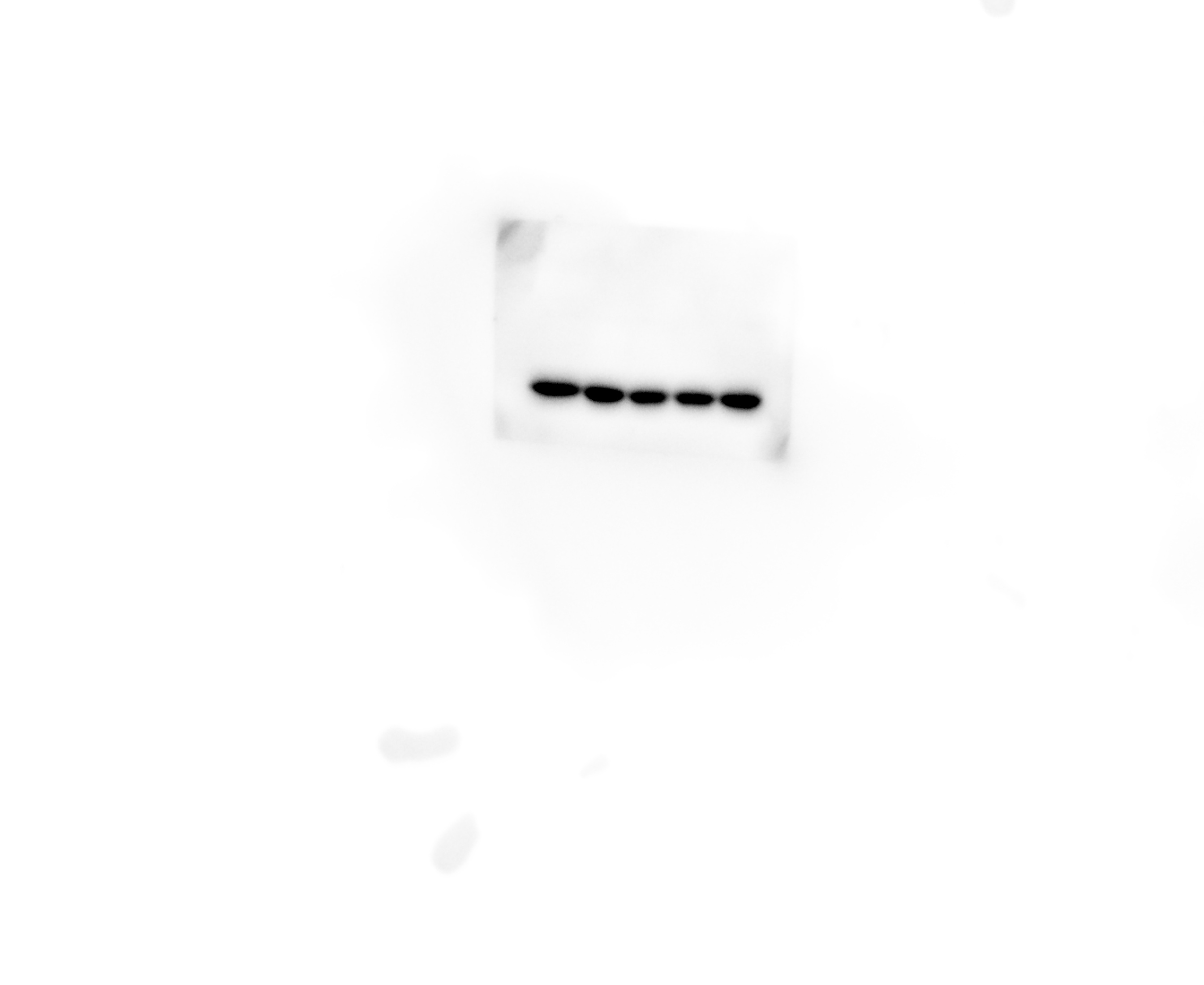

Supplement: Supplementary file 1 — Source Data Fig. 1 [file 44318_2024_66_MOESM1_ESM.zip › Figure 5/C-SFB-SLX4-CoIP/PCNA-input.jpg]

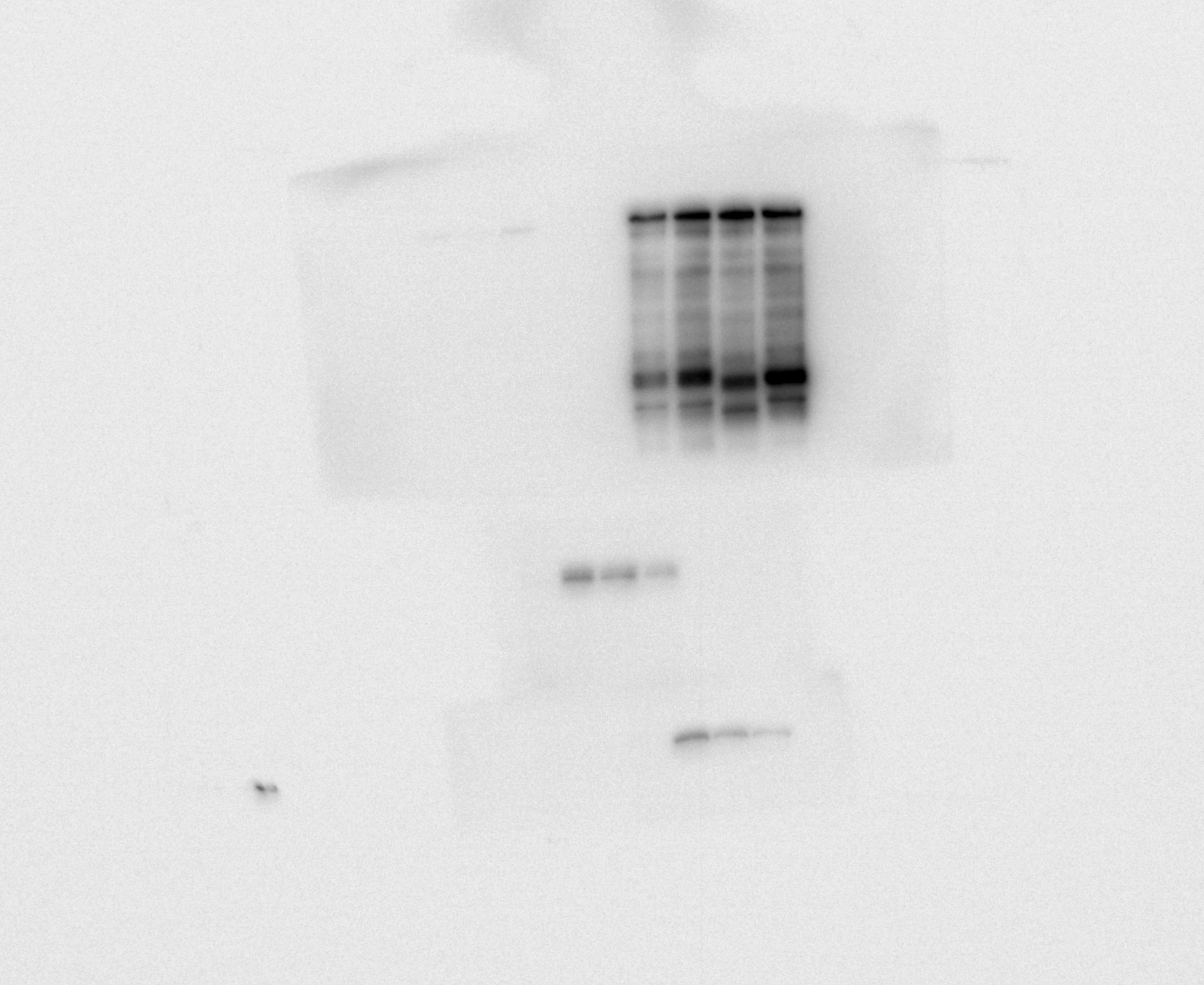

Supplement: Supplementary file 1 — Source Data Fig. 1 [file 44318_2024_66_MOESM1_ESM.zip › Figure 5/C-SFB-SLX4-CoIP/flag-input.jpg]

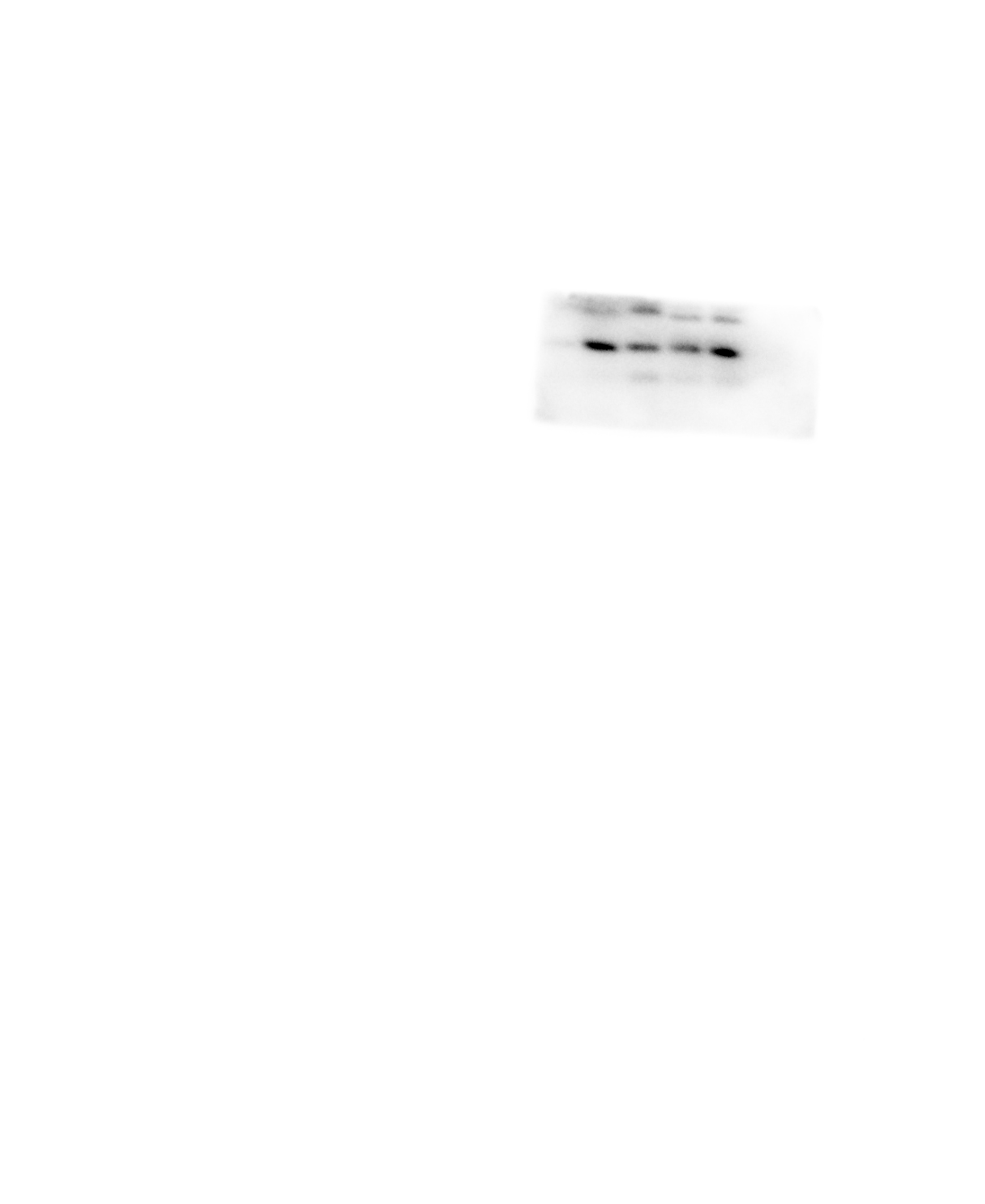

Supplement: Supplementary file 1 — Source Data Fig. 1 [file 44318_2024_66_MOESM1_ESM.zip › Figure 5/C-SFB-SLX4-CoIP/PCNA-CoIP.jpg]

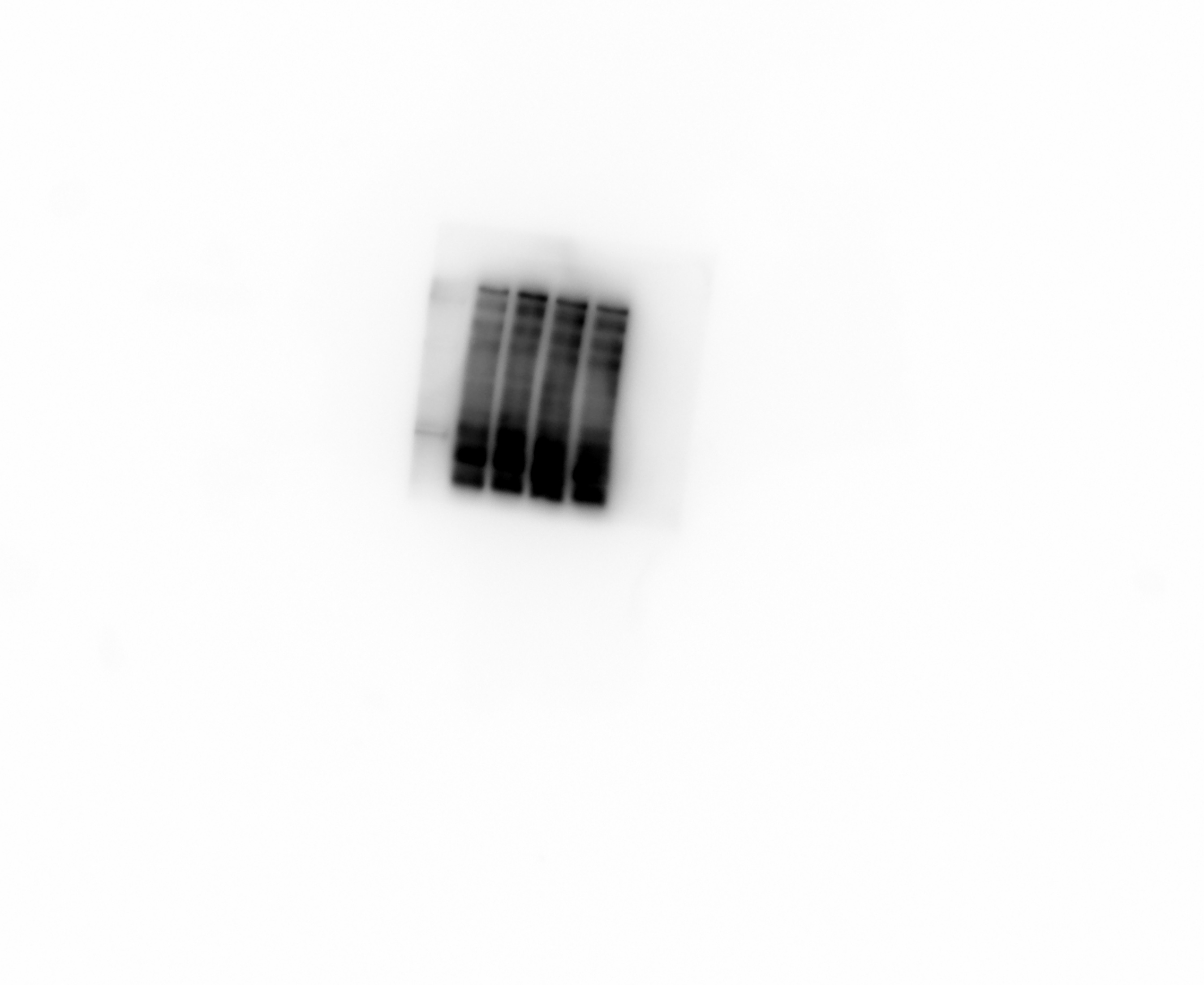

Supplement: Supplementary file 1 — Source Data Fig. 1 [file 44318_2024_66_MOESM1_ESM.zip › Figure 5/C-SFB-SLX4-CoIP/flag-ip.jpg]

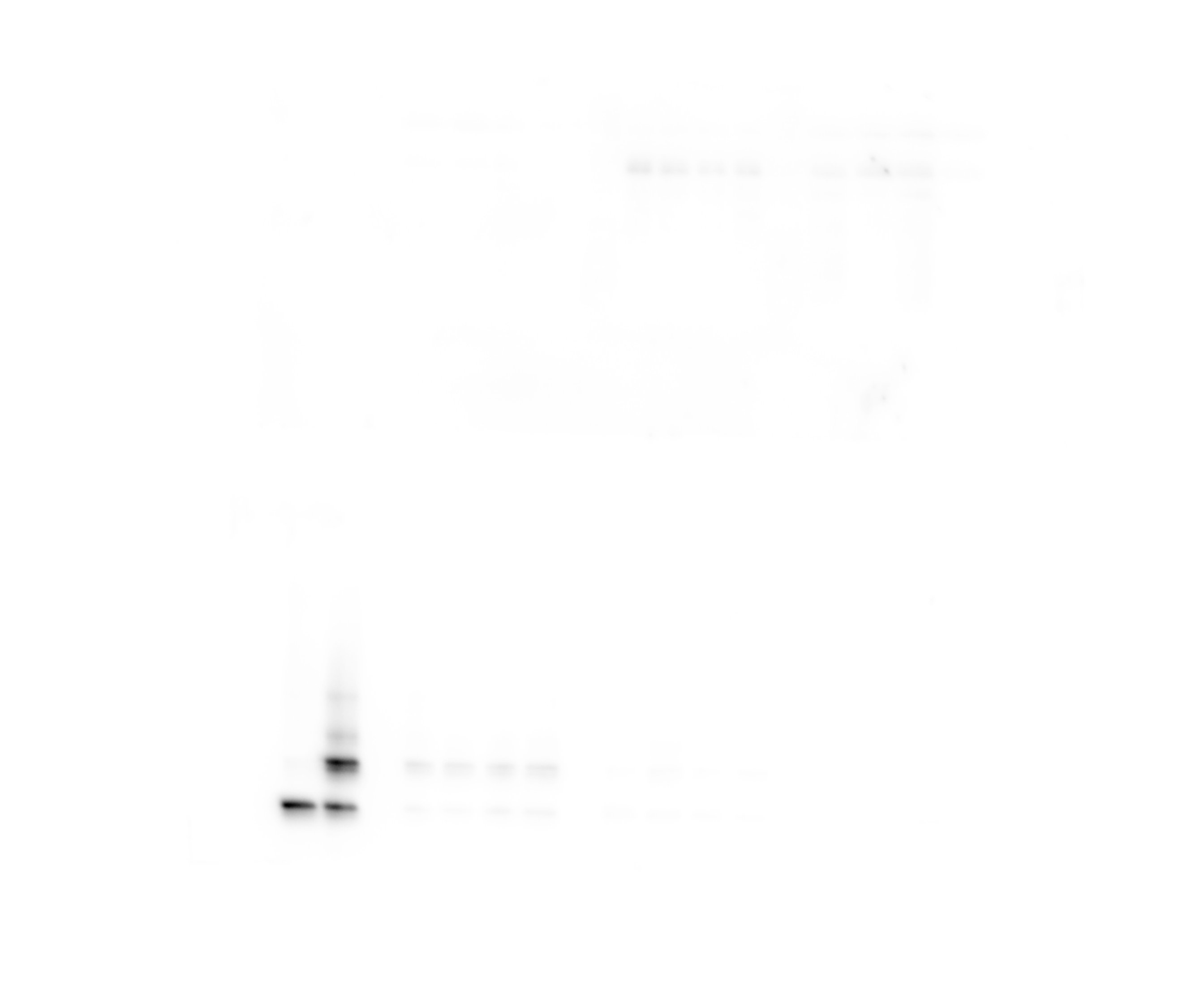

Supplement: Supplementary file 1 — Source Data Fig. 1 [file 44318_2024_66_MOESM1_ESM.zip › Figure 5/D-UbPCNA/PCNA-UB.jpg]

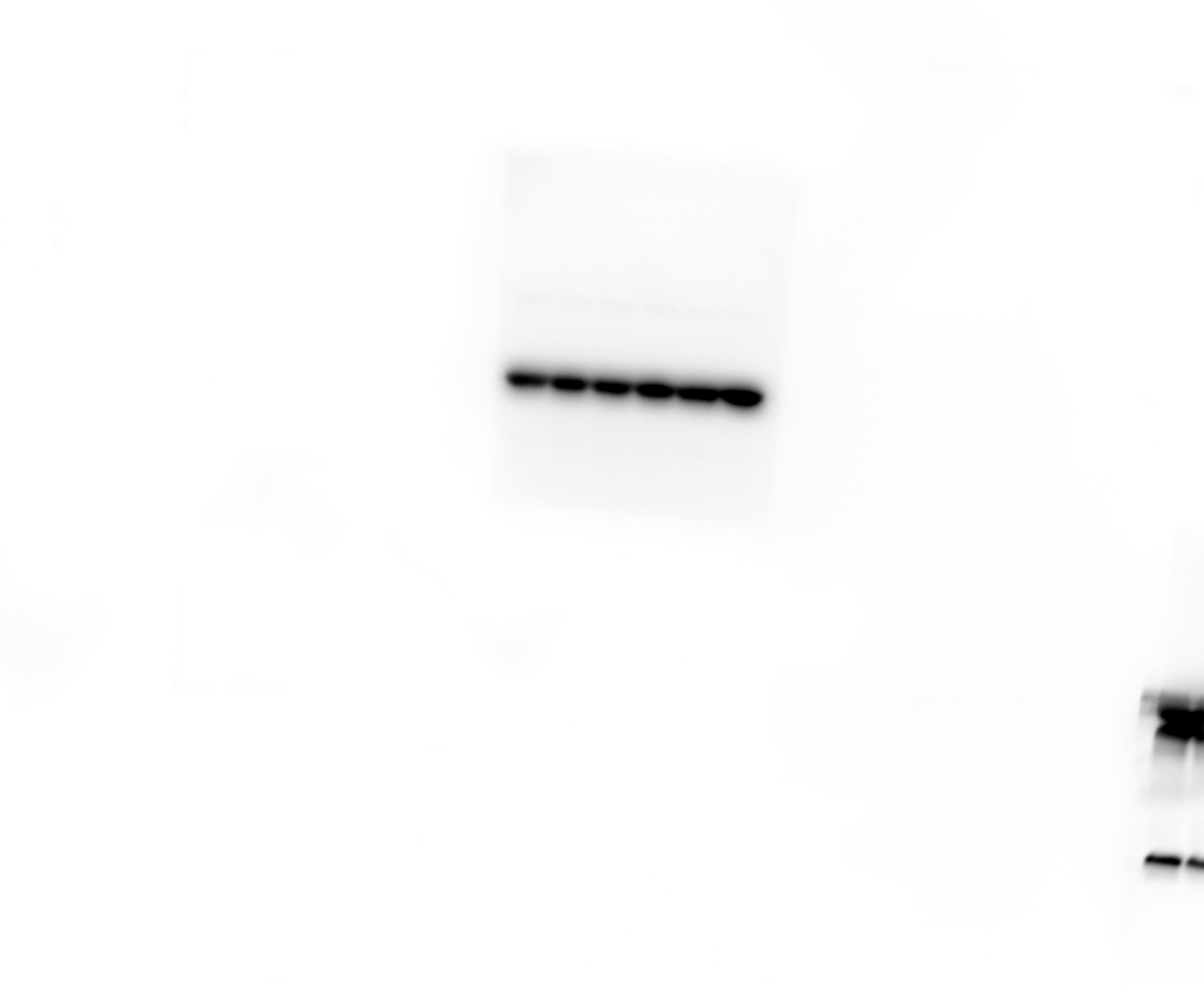

Supplement: Supplementary file 1 — Source Data Fig. 1 [file 44318_2024_66_MOESM1_ESM.zip › Figure 5/B-SFB-SLX4-UbPCNA-CoIP/pcna-input.jpg]

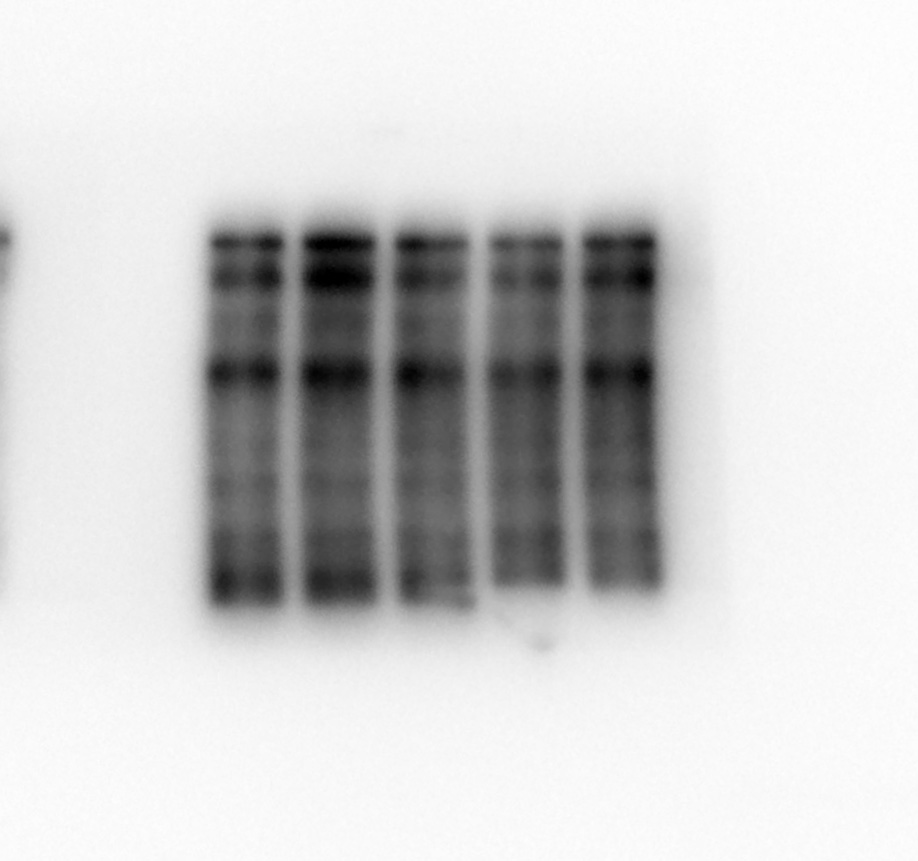

Supplement: Supplementary file 1 — Source Data Fig. 1 [file 44318_2024_66_MOESM1_ESM.zip › Figure 5/B-SFB-SLX4-UbPCNA-CoIP/flag-input.jpg]

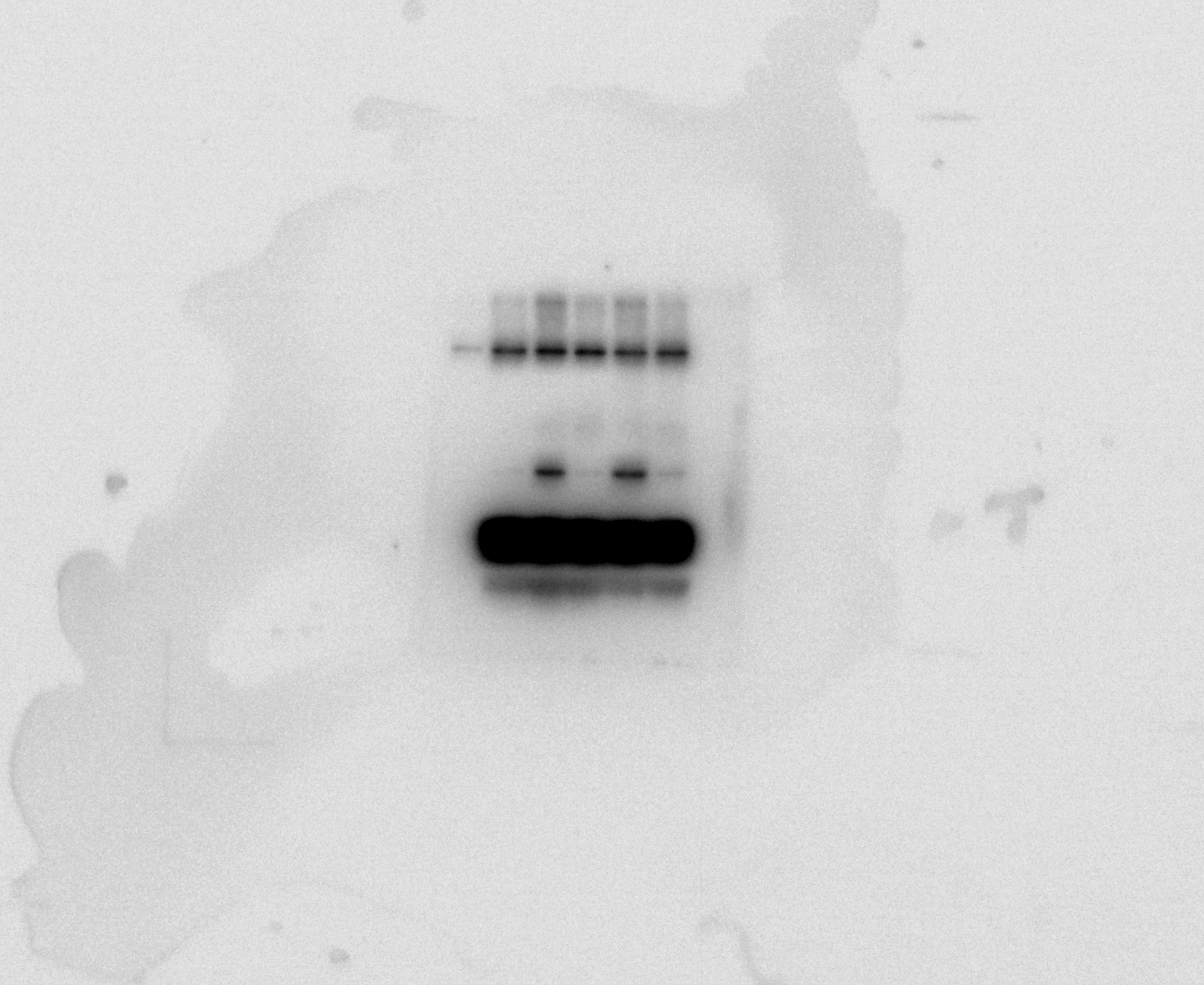

Supplement: Supplementary file 1 — Source Data Fig. 1 [file 44318_2024_66_MOESM1_ESM.zip › Figure 5/B-SFB-SLX4-UbPCNA-CoIP/pcna-coip.jpg]

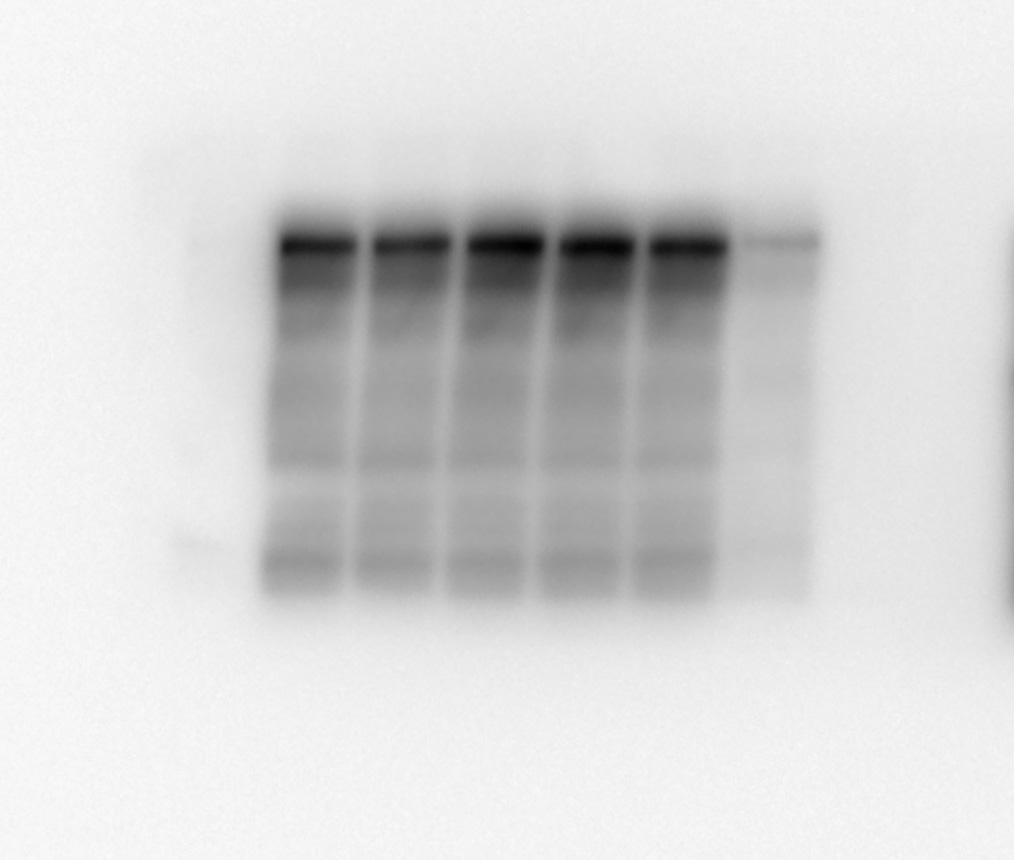

Supplement: Supplementary file 1 — Source Data Fig. 1 [file 44318_2024_66_MOESM1_ESM.zip › Figure 5/B-SFB-SLX4-UbPCNA-CoIP/flag-ip.jpg]

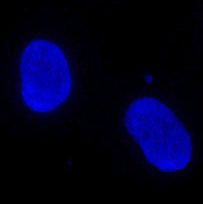

Supplement: Supplementary file 1 — Source Data Fig. 1 [file 44318_2024_66_MOESM1_ESM.zip › Figure 5/F-230727-SLX4-UBZ-PLA-image/No Click.tif]

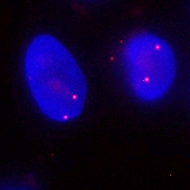

Supplement: Supplementary file 1 — Source Data Fig. 1 [file 44318_2024_66_MOESM1_ESM.zip › Figure 5/F-230727-SLX4-UBZ-PLA-image/UBZ+.tif]

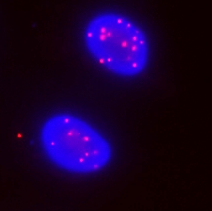

Supplement: Supplementary file 1 — Source Data Fig. 1 [file 44318_2024_66_MOESM1_ESM.zip › Figure 5/F-230727-SLX4-UBZ-PLA-image/WT+tif.tif]

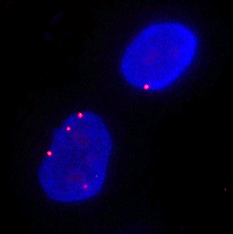

Supplement: Supplementary file 1 — Source Data Fig. 1 [file 44318_2024_66_MOESM1_ESM.zip › Figure 5/F-230727-SLX4-UBZ-PLA-image/WT-tif.tif]

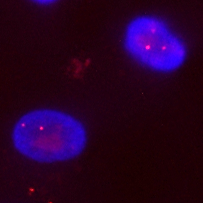

Supplement: Supplementary file 1 — Source Data Fig. 1 [file 44318_2024_66_MOESM1_ESM.zip › Figure 5/F-230727-SLX4-UBZ-PLA-image/UBZ-tif.tif]

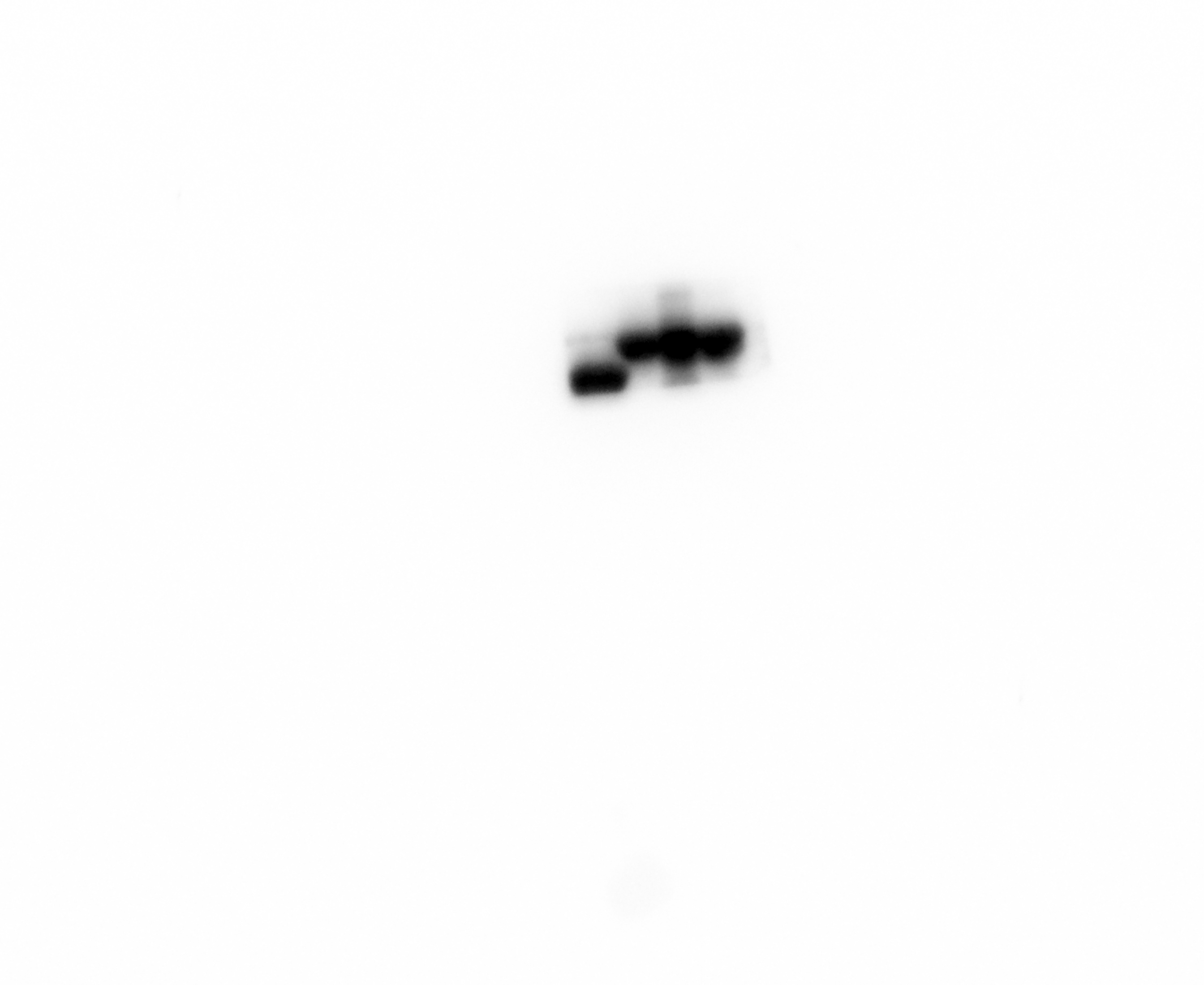

Supplement: Supplementary file 1 — Source Data Fig. 1 [file 44318_2024_66_MOESM1_ESM.zip › Figure 5/E-UbPCNA-Pulldown/GST-Pulldown.jpg]

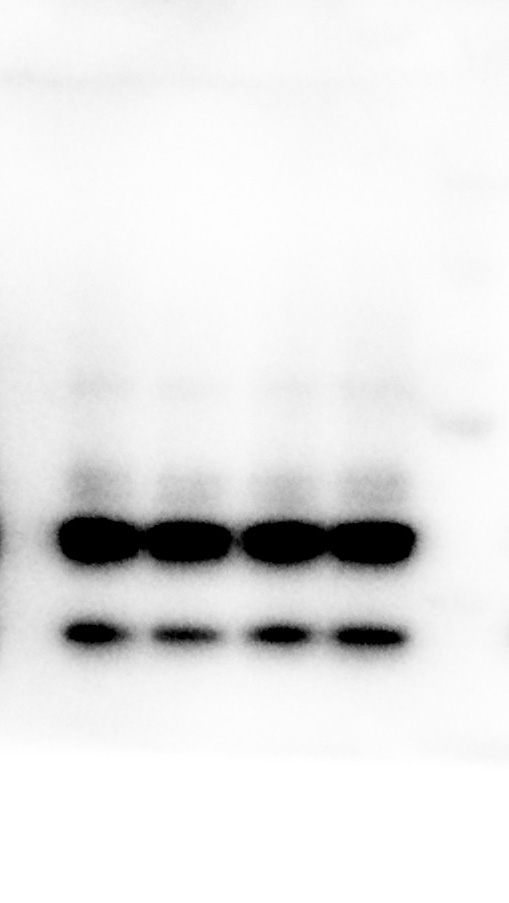

Supplement: Supplementary file 1 — Source Data Fig. 1 [file 44318_2024_66_MOESM1_ESM.zip › Figure 5/E-UbPCNA-Pulldown/SFB-PCNA-Ub-Input.jpg]

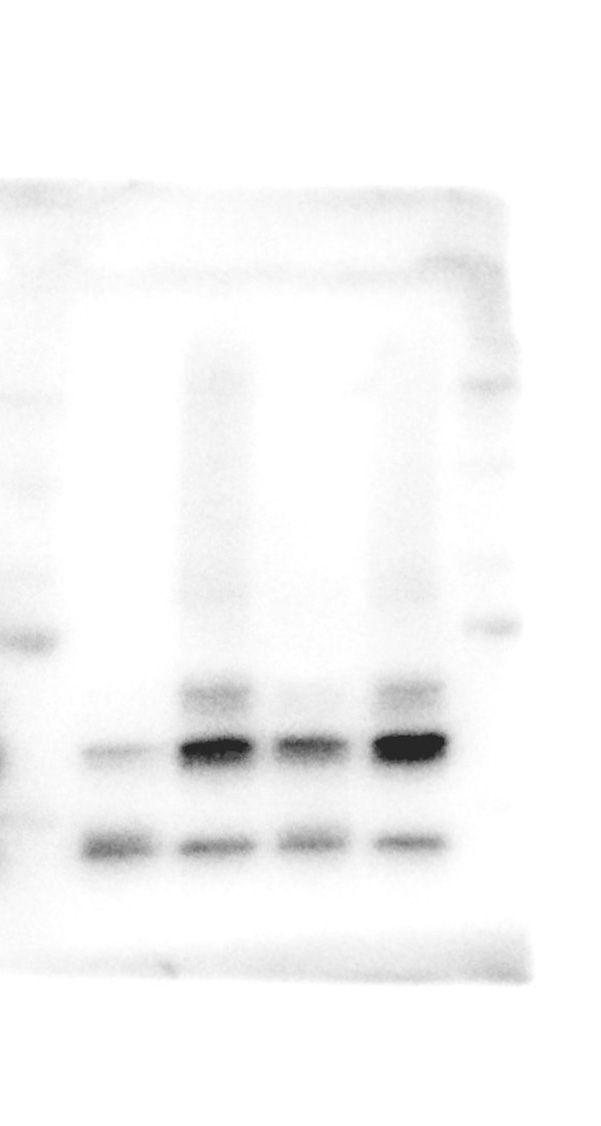

Supplement: Supplementary file 1 — Source Data Fig. 1 [file 44318_2024_66_MOESM1_ESM.zip › Figure 5/E-UbPCNA-Pulldown/SFB-PCNA-ub-pulldown.jpg]

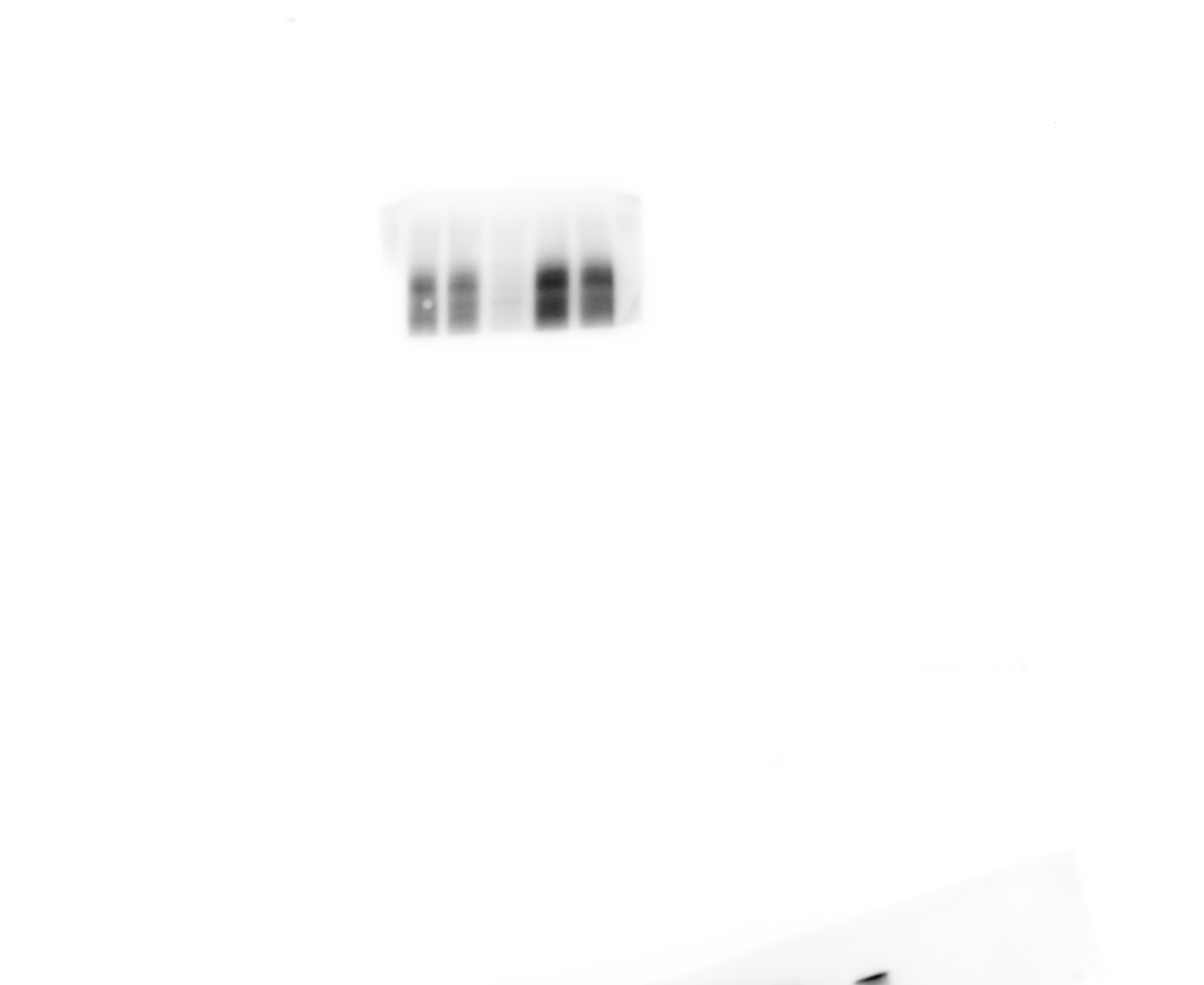

Supplement: Supplementary file 1 — Source Data Fig. 1 [file 44318_2024_66_MOESM1_ESM.zip › Figure 5/H-230911-p-ATM-WT-UBZ/slx4-Knockown.jpg]

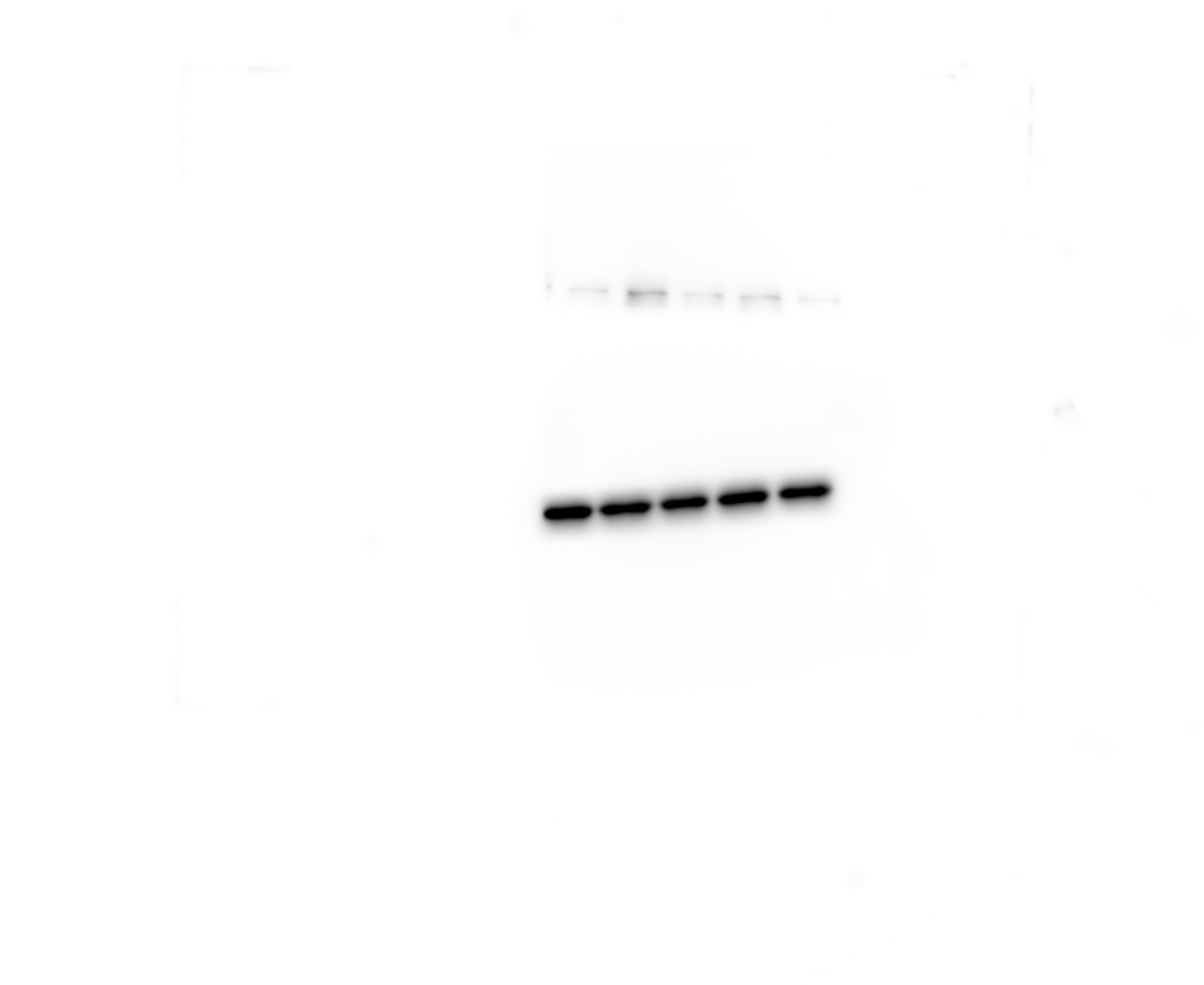

Supplement: Supplementary file 1 — Source Data Fig. 1 [file 44318_2024_66_MOESM1_ESM.zip › Figure 5/H-230911-p-ATM-WT-UBZ/GAPDH.jpg]

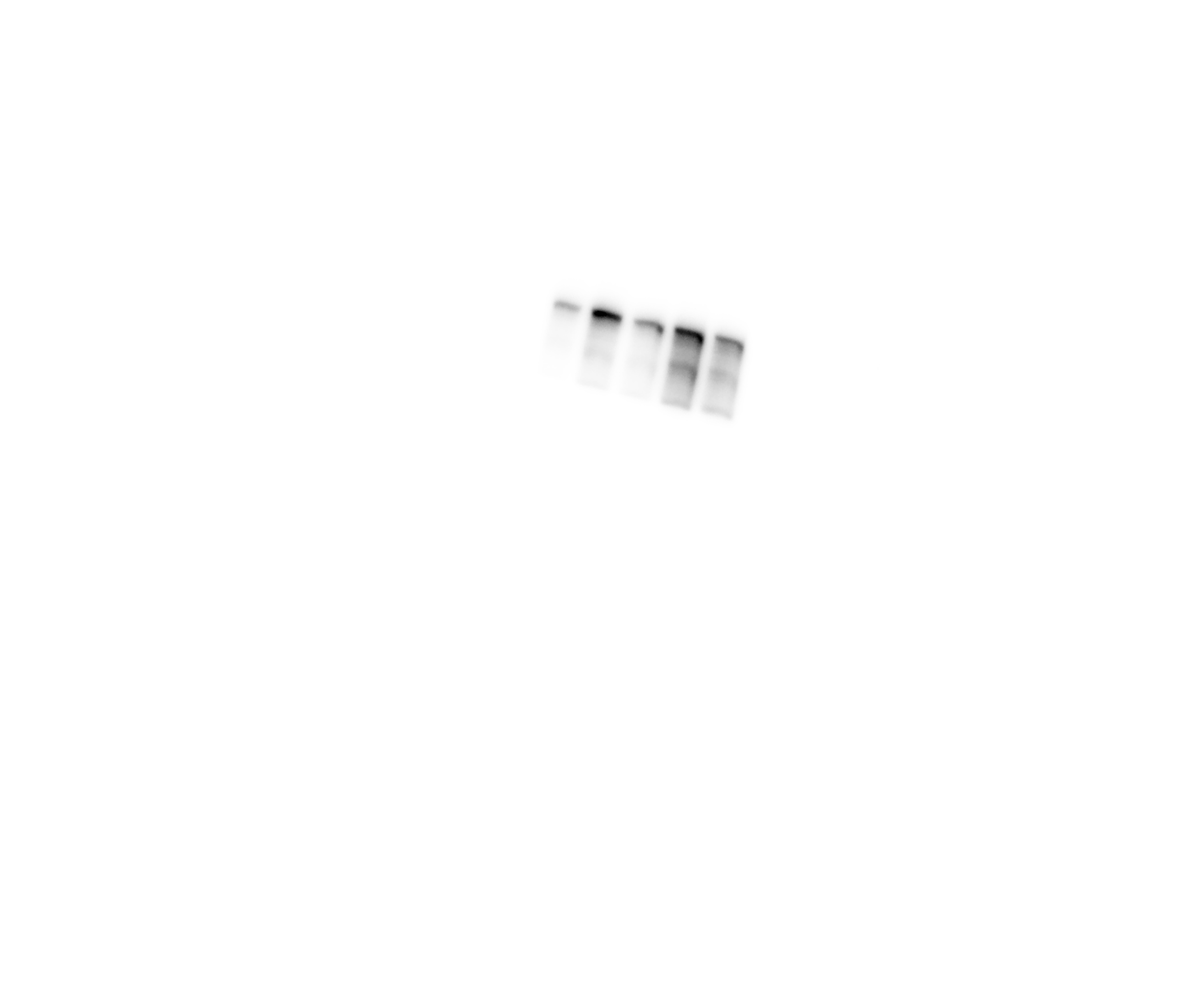

Supplement: Supplementary file 1 — Source Data Fig. 1 [file 44318_2024_66_MOESM1_ESM.zip › Figure 5/H-230911-p-ATM-WT-UBZ/pSer1981-ATM.jpg]

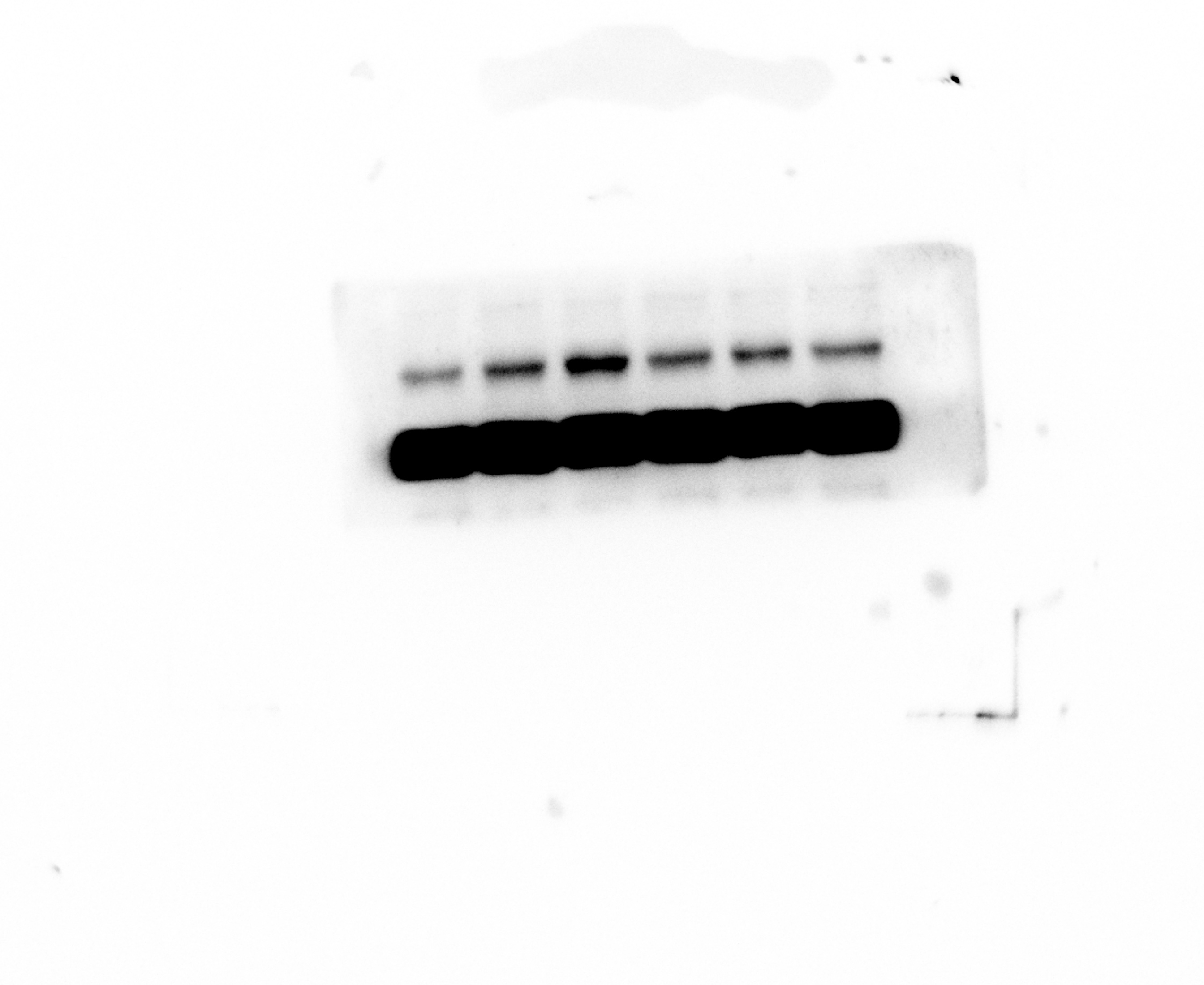

Supplement: Supplementary file 2 — Source Data Fig. 2 [file 44318_2024_66_MOESM2_ESM.zip › Figure 1/F-210704-pcna-chki-wee1i/pcna-LONG.jpg]

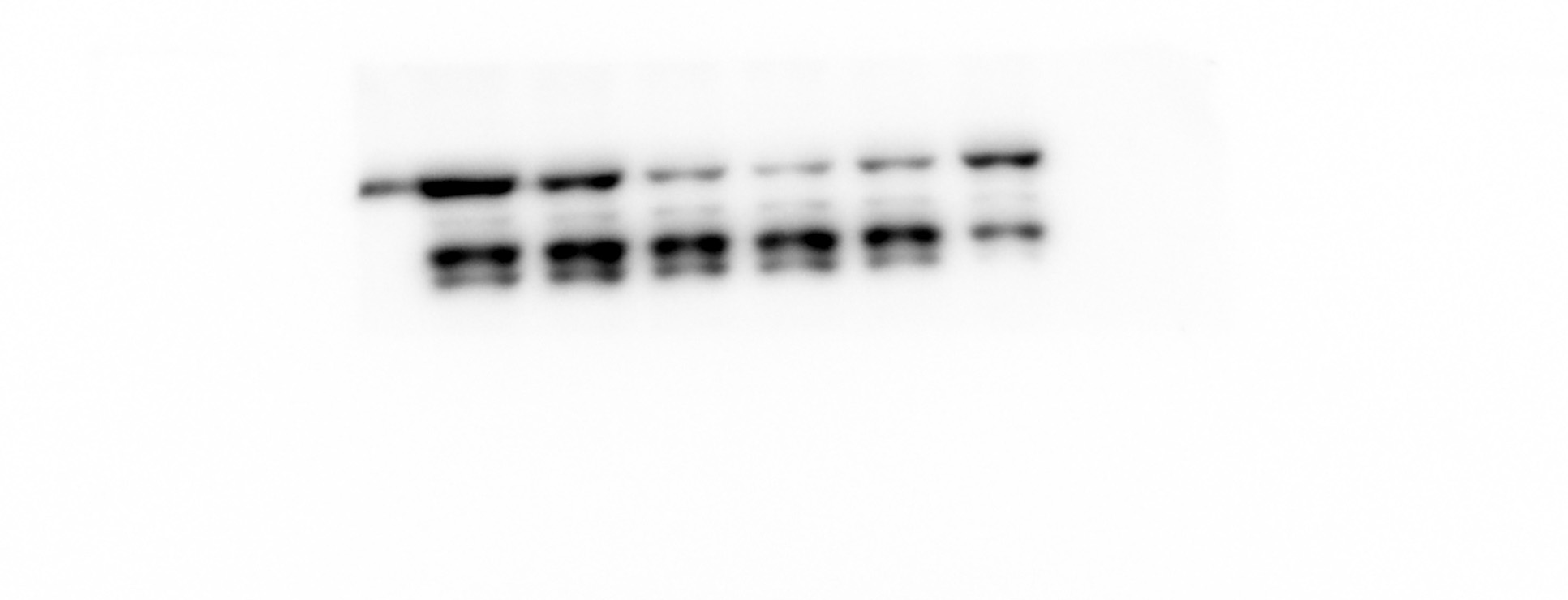

Supplement: Supplementary file 2 — Source Data Fig. 2 [file 44318_2024_66_MOESM2_ESM.zip › Figure 1/F-210704-pcna-chki-wee1i/pTyr15-CDK1.jpg]

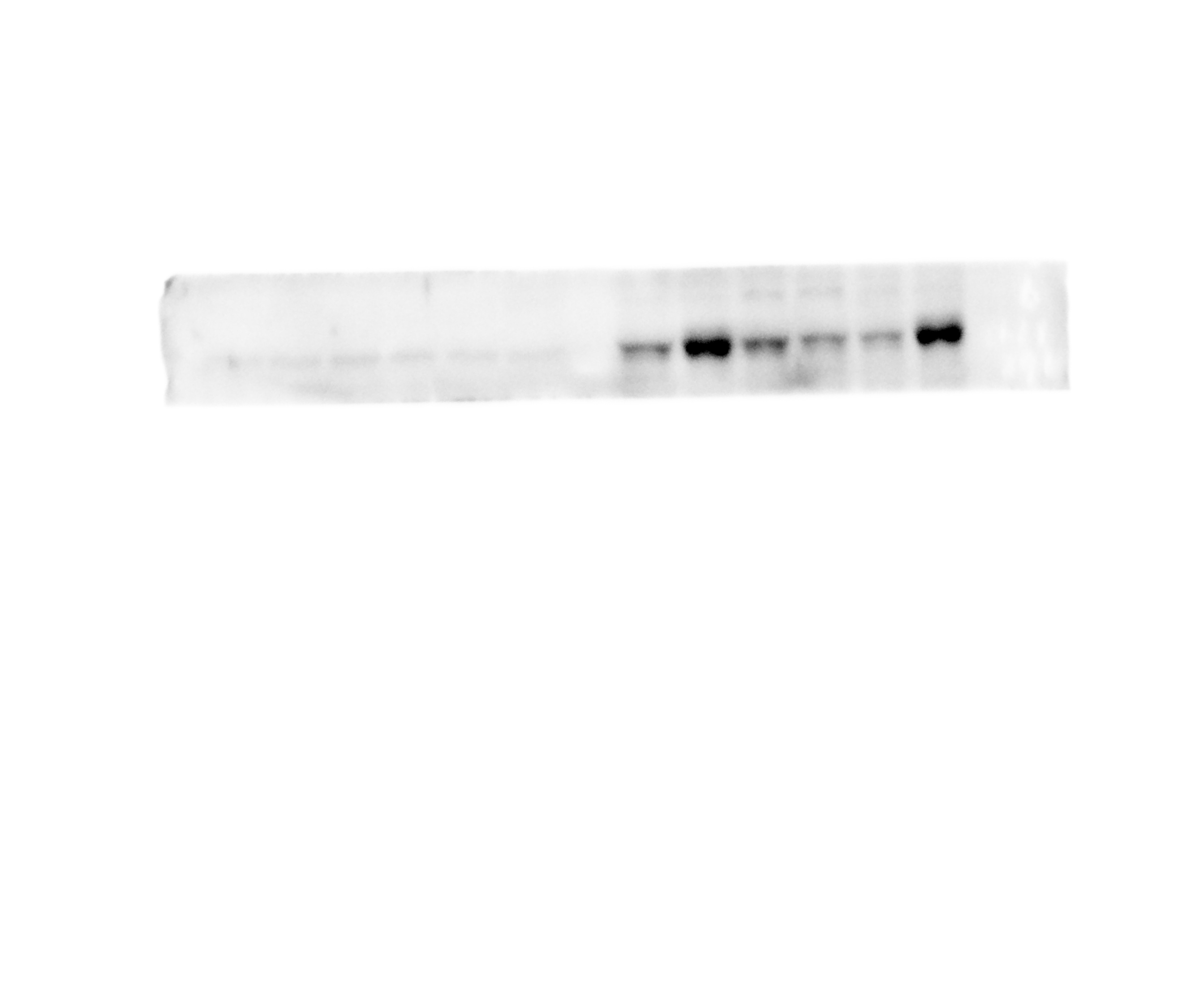

Supplement: Supplementary file 2 — Source Data Fig. 2 [file 44318_2024_66_MOESM2_ESM.zip › Figure 1/F-210704-pcna-chki-wee1i/pSer296-Chk1.jpg]

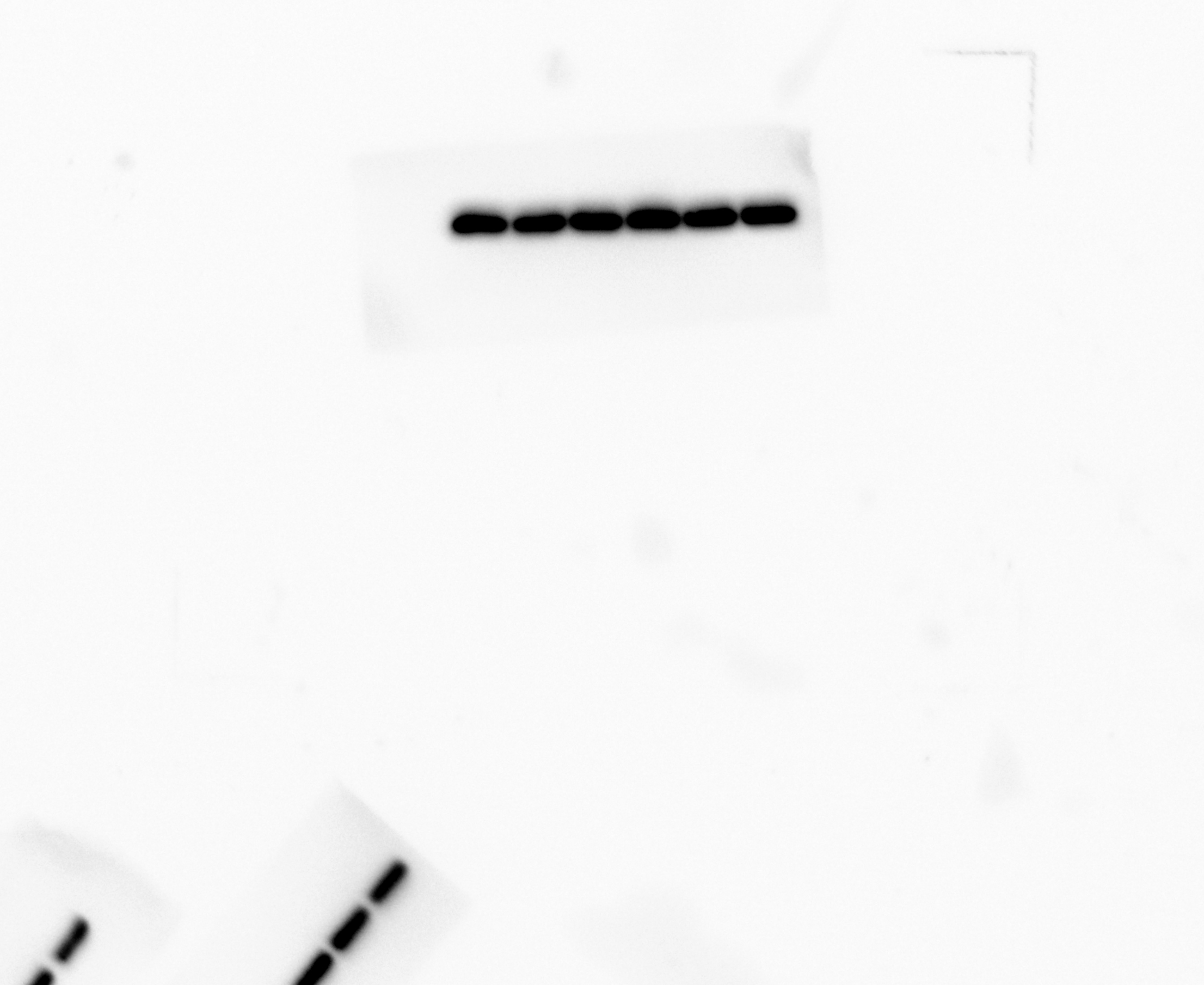

Supplement: Supplementary file 2 — Source Data Fig. 2 [file 44318_2024_66_MOESM2_ESM.zip › Figure 1/F-210704-pcna-chki-wee1i/H3.jpg]

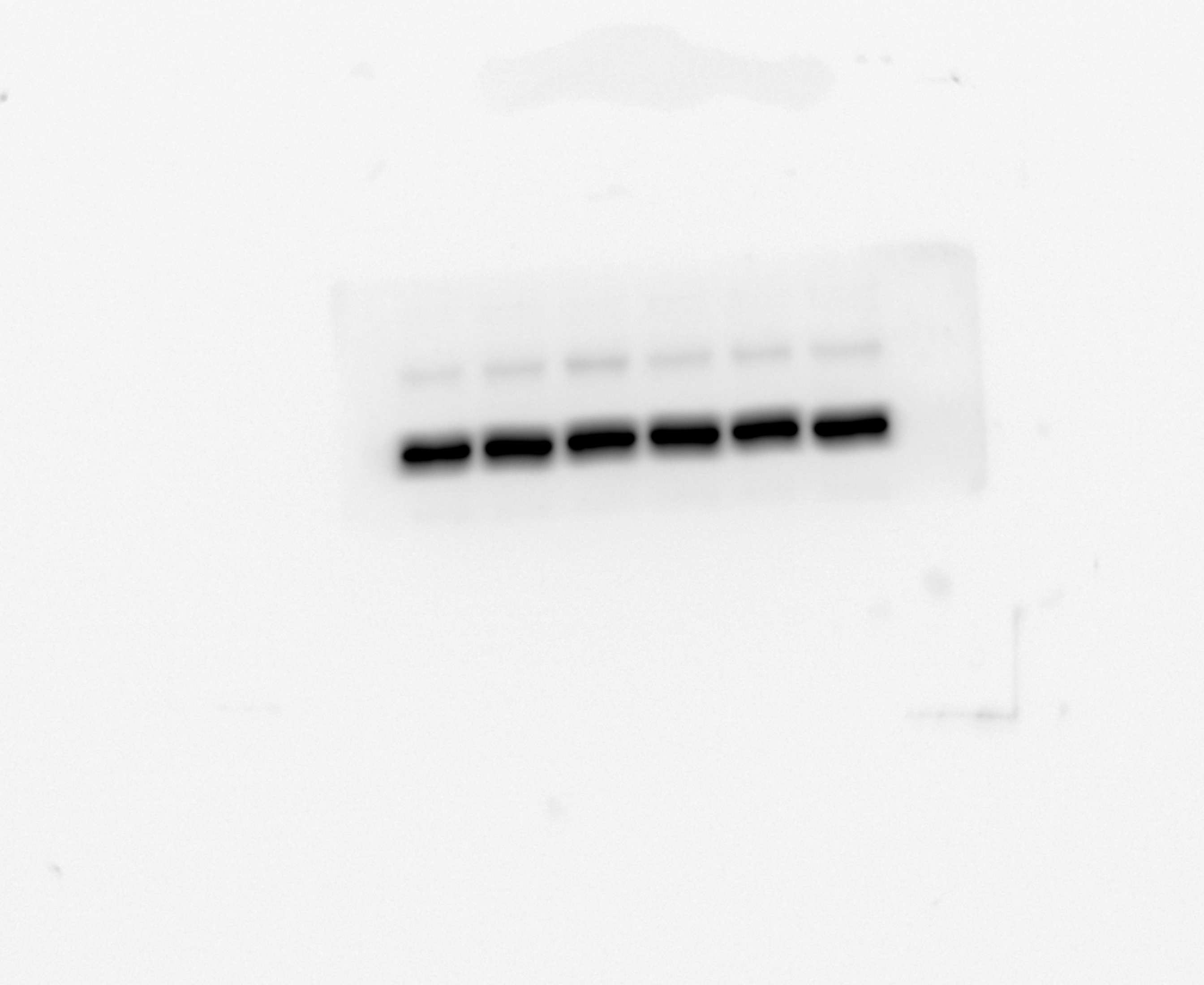

Supplement: Supplementary file 2 — Source Data Fig. 2 [file 44318_2024_66_MOESM2_ESM.zip › Figure 1/F-210704-pcna-chki-wee1i/pcna-short.jpg]

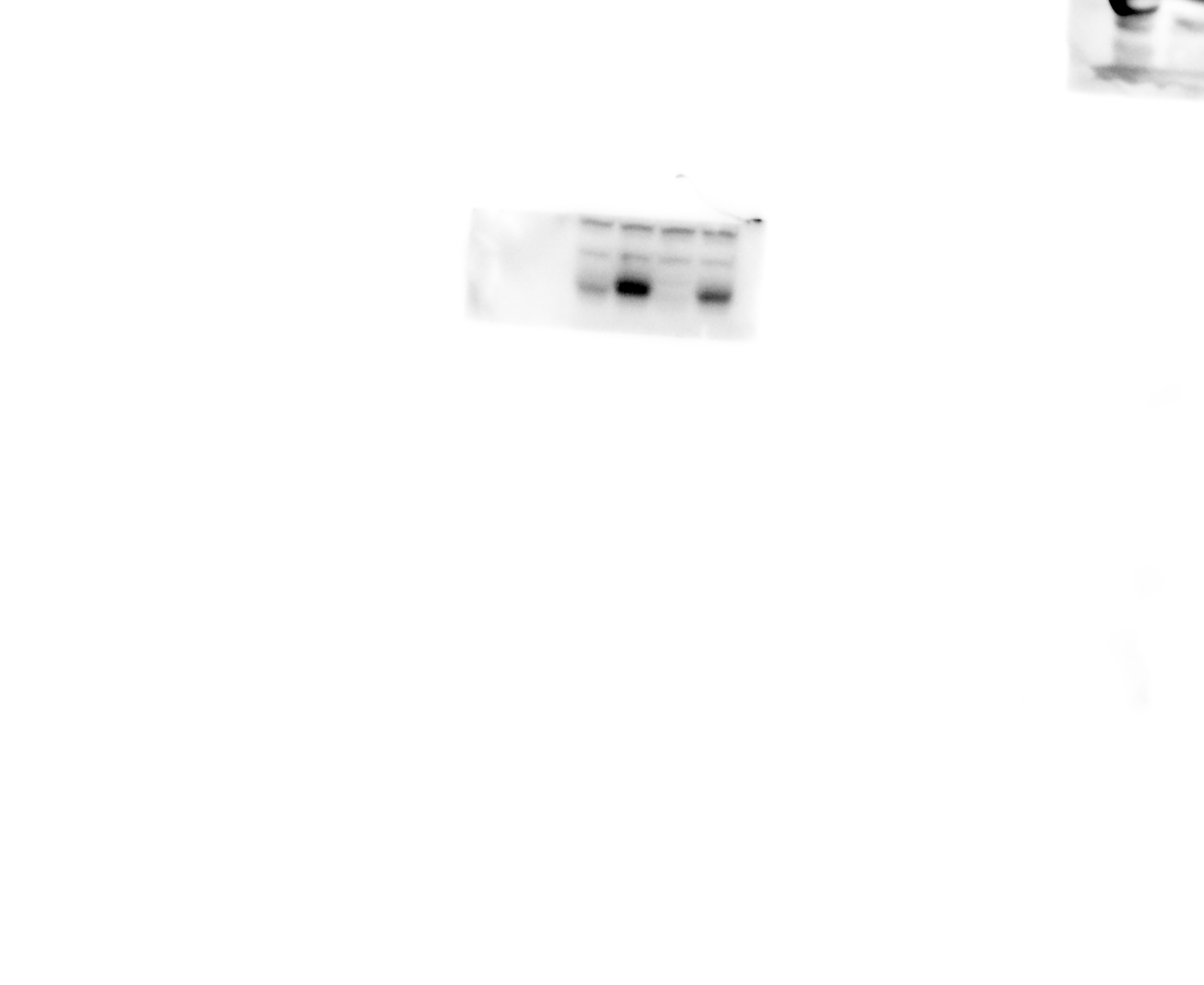

Supplement: Supplementary file 2 — Source Data Fig. 2 [file 44318_2024_66_MOESM2_ESM.zip › Figure 1/C-220125-PCNA-ub-APH/pSer345-Chk1.jpg]

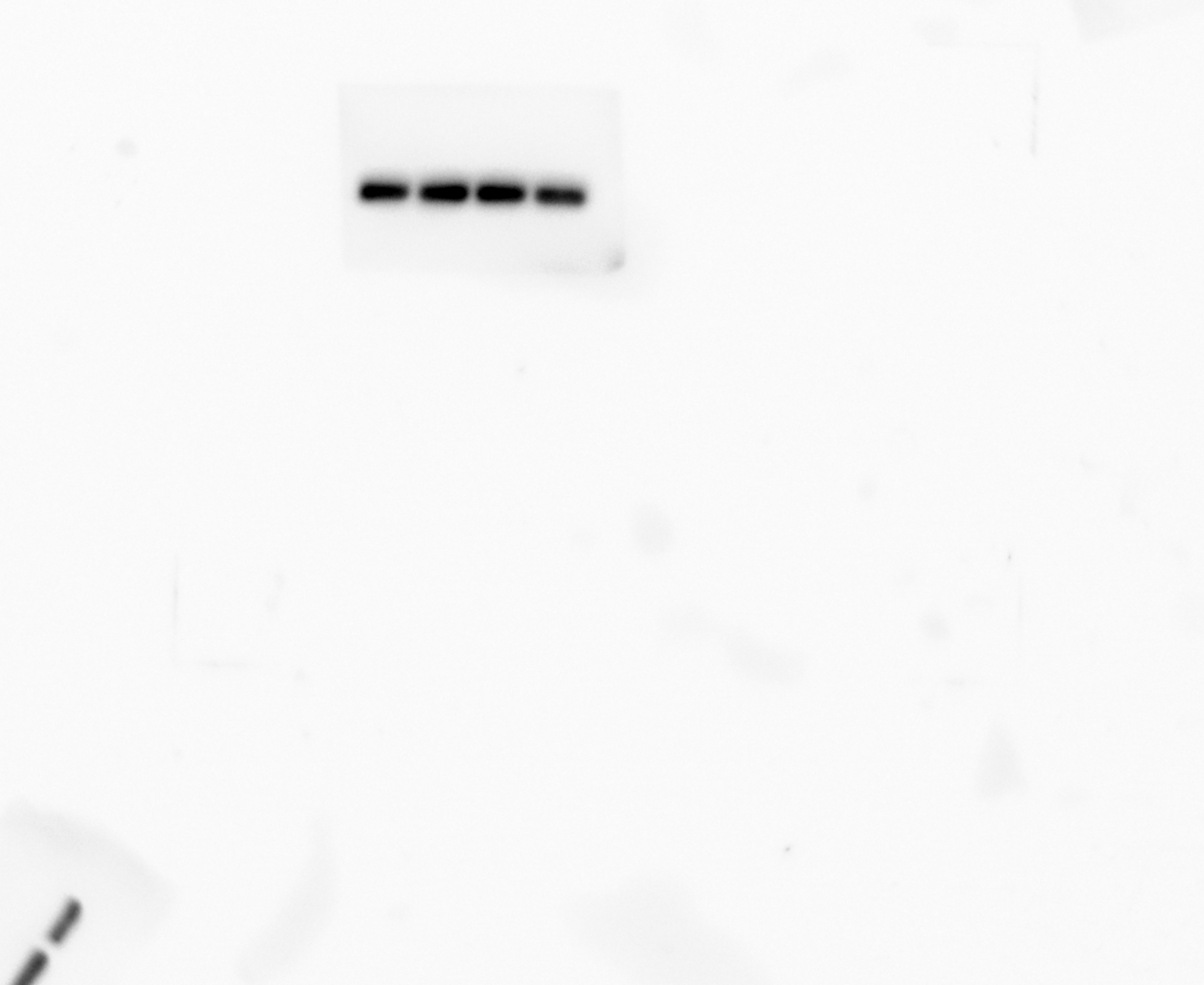

Supplement: Supplementary file 2 — Source Data Fig. 2 [file 44318_2024_66_MOESM2_ESM.zip › Figure 1/C-220125-PCNA-ub-APH/H3.jpg]

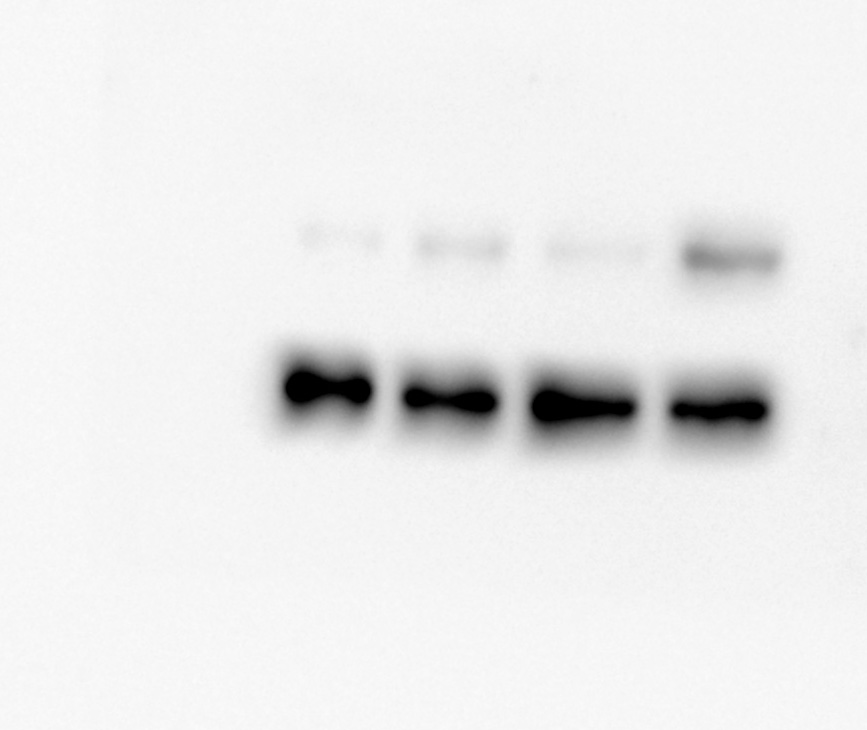

Supplement: Supplementary file 2 — Source Data Fig. 2 [file 44318_2024_66_MOESM2_ESM.zip › Figure 1/C-220125-PCNA-ub-APH/PCNA-CH-SHORT.jpg]

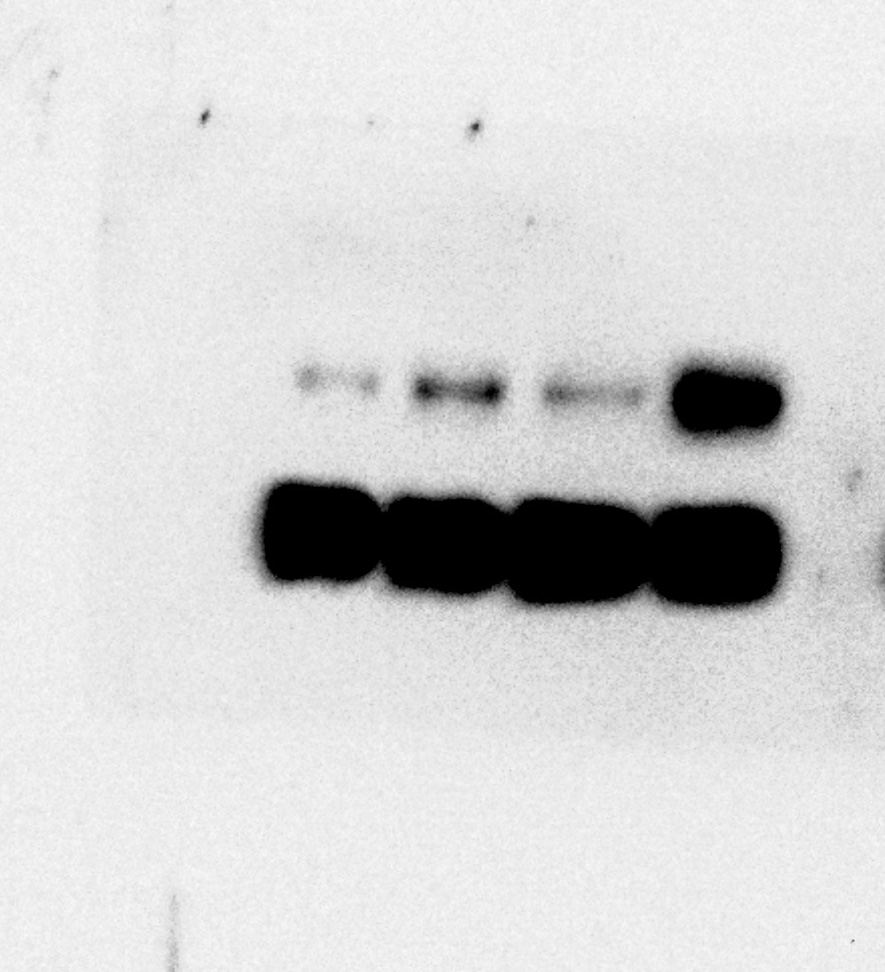

Supplement: Supplementary file 2 — Source Data Fig. 2 [file 44318_2024_66_MOESM2_ESM.zip › Figure 1/C-220125-PCNA-ub-APH/PCNA-CH-LONG.jpg]

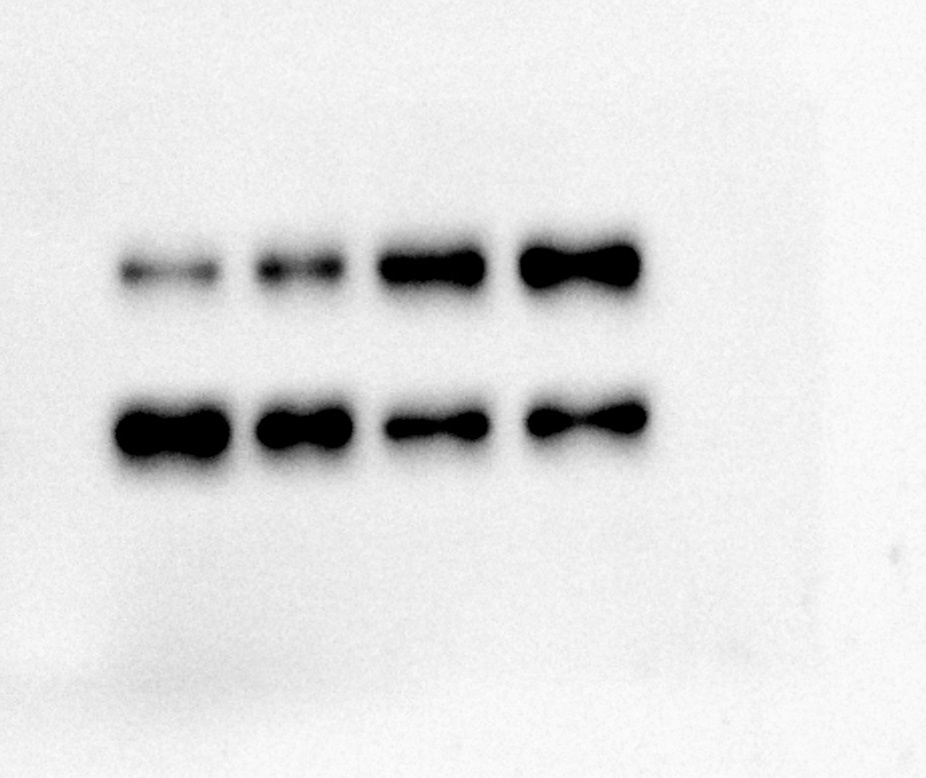

Supplement: Supplementary file 2 — Source Data Fig. 2 [file 44318_2024_66_MOESM2_ESM.zip › Figure 1/B-220122-PCNA-ATR-KD/PCNA-LONG.jpg]

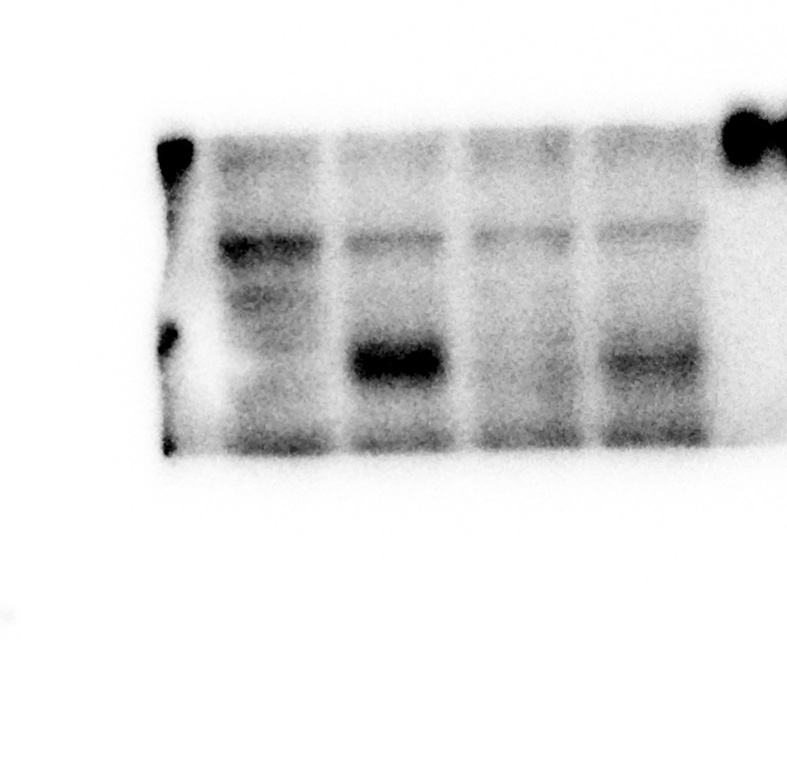

Supplement: Supplementary file 2 — Source Data Fig. 2 [file 44318_2024_66_MOESM2_ESM.zip › Figure 1/B-220122-PCNA-ATR-KD/pSer345-CHK1.jpg]

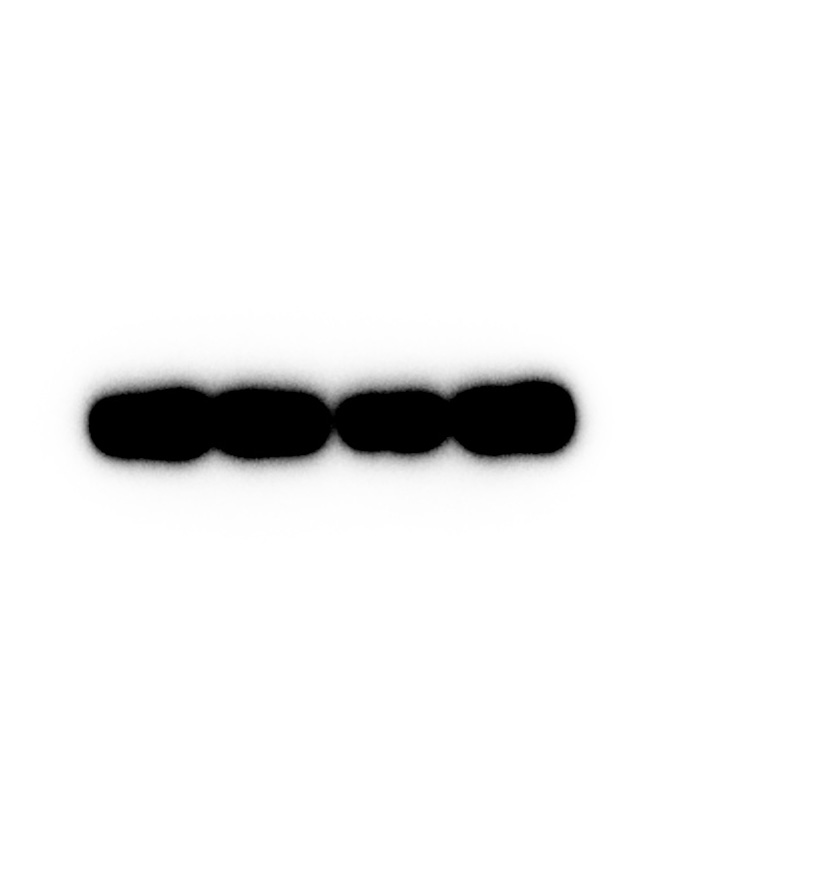

Supplement: Supplementary file 2 — Source Data Fig. 2 [file 44318_2024_66_MOESM2_ESM.zip › Figure 1/B-220122-PCNA-ATR-KD/H3.jpg]

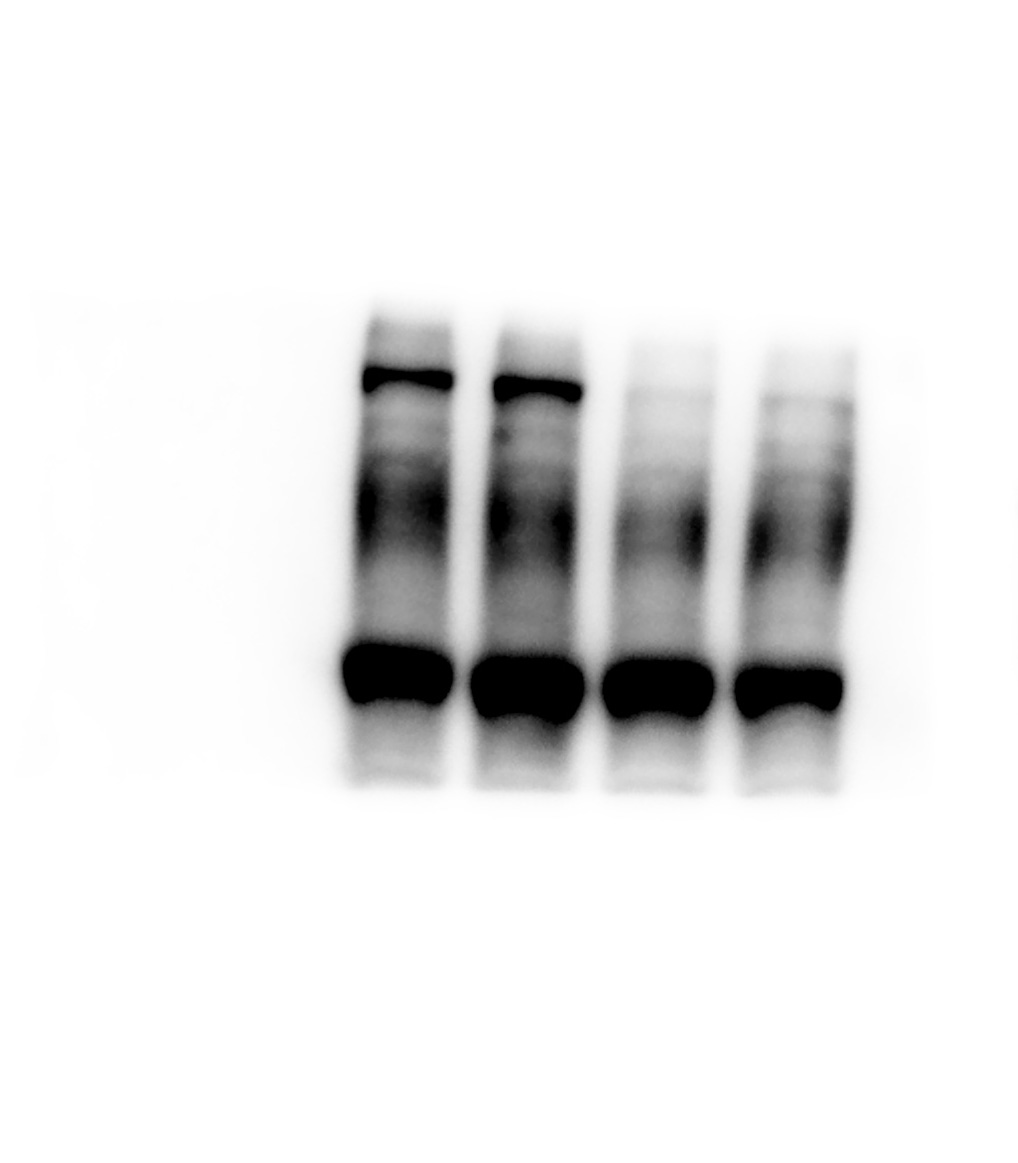

Supplement: Supplementary file 2 — Source Data Fig. 2 [file 44318_2024_66_MOESM2_ESM.zip › Figure 1/B-220122-PCNA-ATR-KD/ATR.jpg]

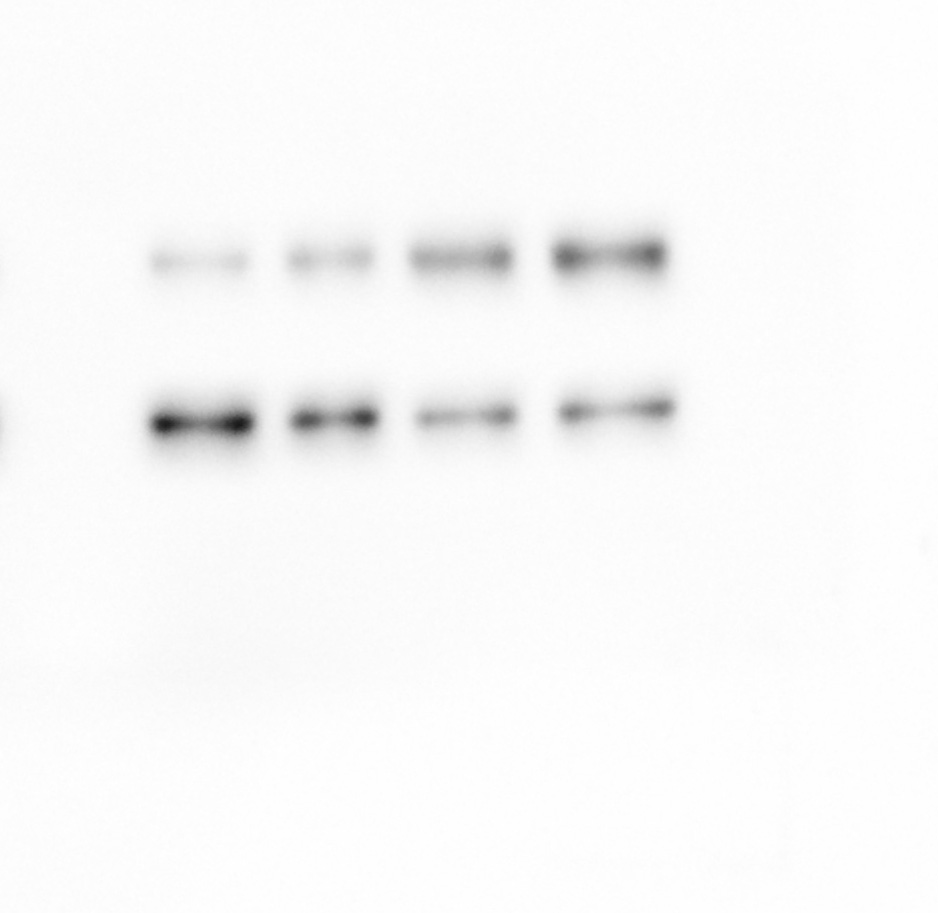

Supplement: Supplementary file 2 — Source Data Fig. 2 [file 44318_2024_66_MOESM2_ESM.zip › Figure 1/B-220122-PCNA-ATR-KD/PCNA-SHORT.jpg]

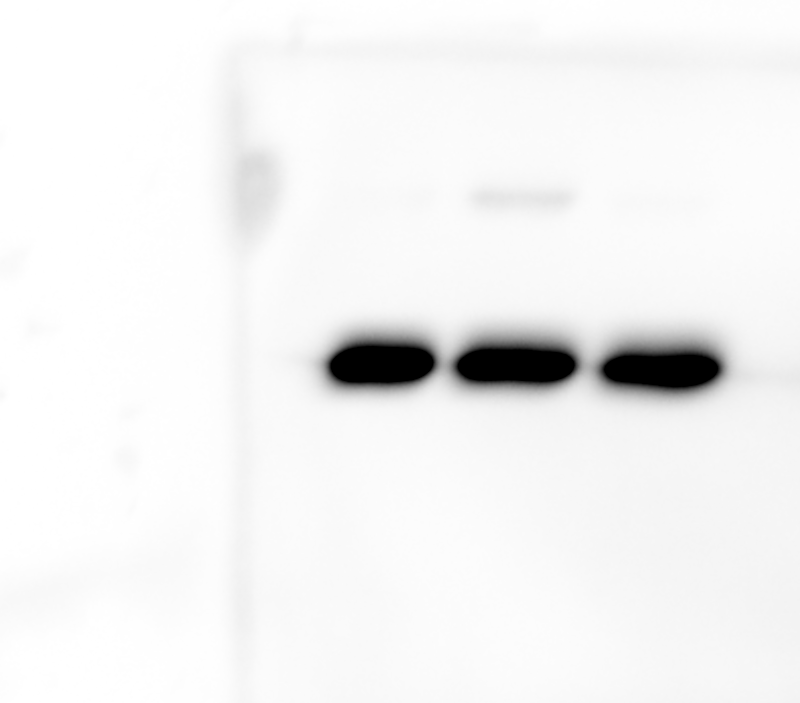

Supplement: Supplementary file 2 — Source Data Fig. 2 [file 44318_2024_66_MOESM2_ESM.zip › Figure 1/E-230407-PCNA-Ub-Rad18-Knowkdown/PCNA-SHORT-RAD18-KNOCKDOWN.tif]

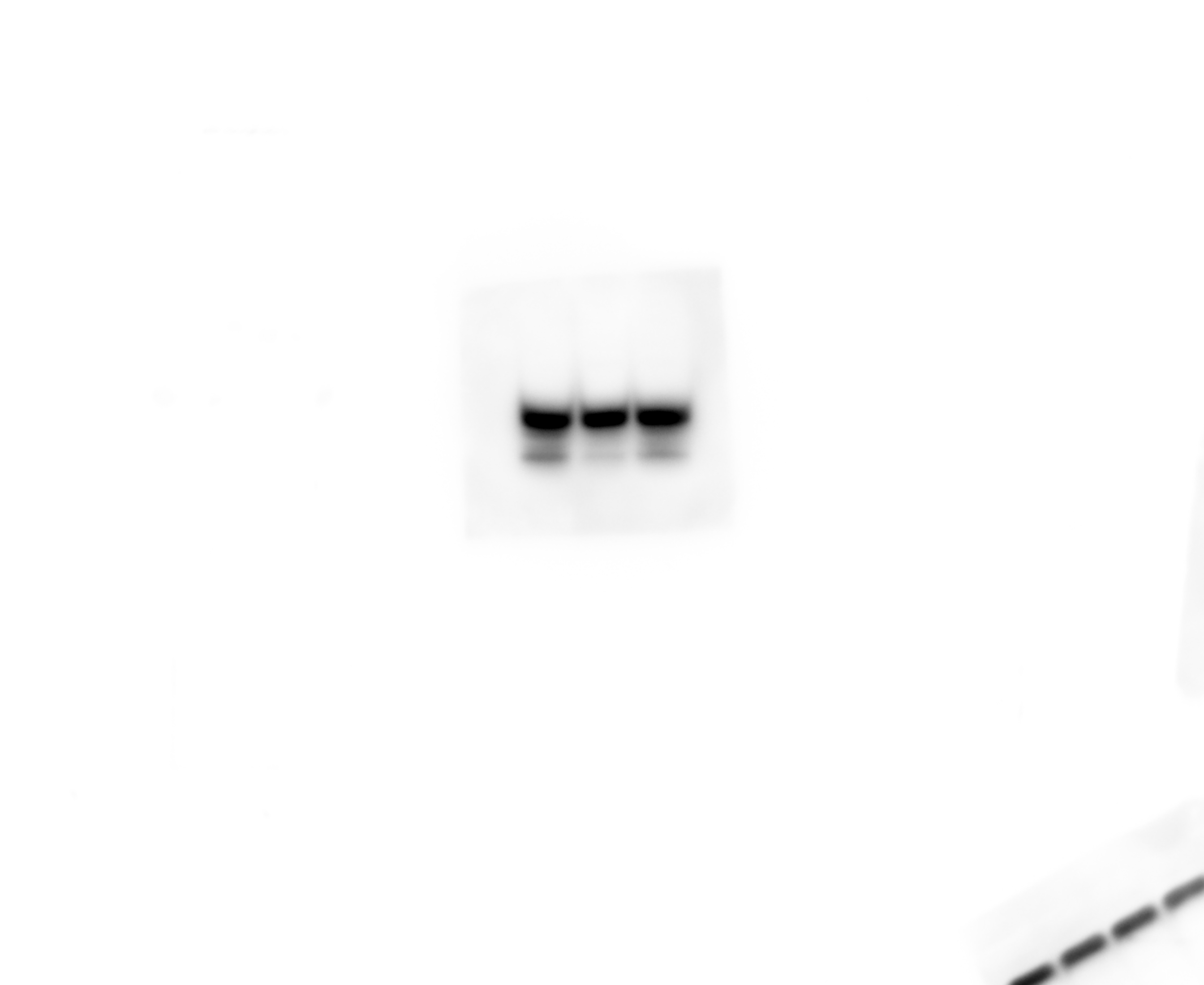

Supplement: Supplementary file 2 — Source Data Fig. 2 [file 44318_2024_66_MOESM2_ESM.zip › Figure 1/E-230407-PCNA-Ub-Rad18-Knowkdown/H3.jpg]

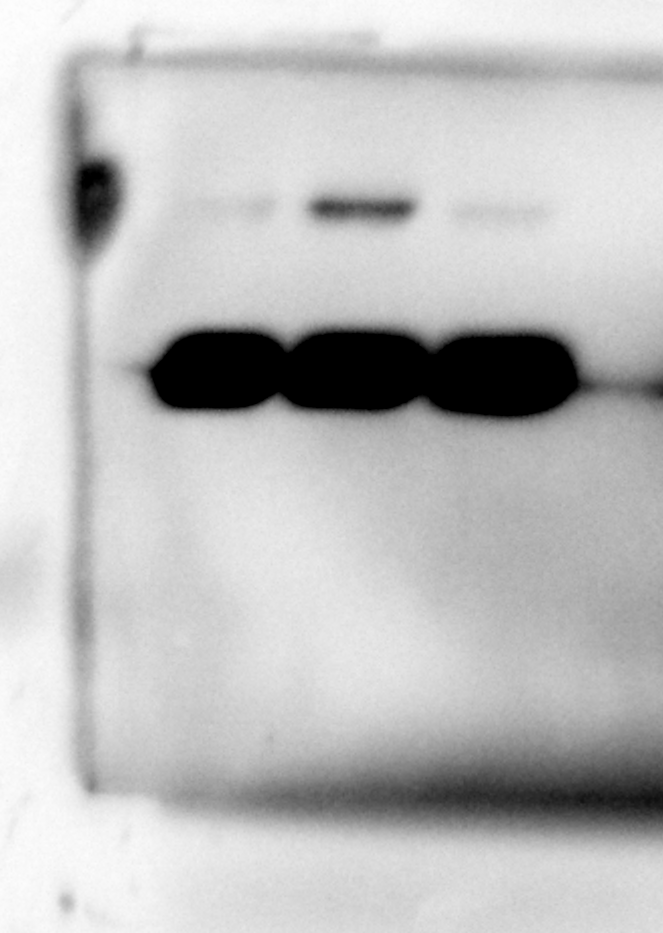

Supplement: Supplementary file 2 — Source Data Fig. 2 [file 44318_2024_66_MOESM2_ESM.zip › Figure 1/E-230407-PCNA-Ub-Rad18-Knowkdown/PCNA-LONG-RAD18-KNOCKDOWN.tif]

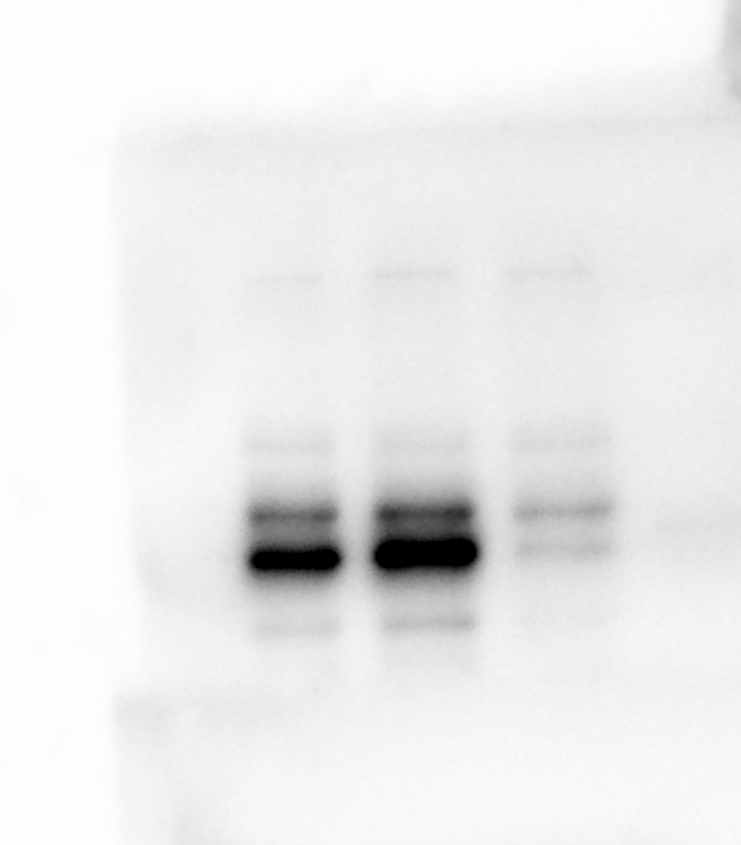

Supplement: Supplementary file 2 — Source Data Fig. 2 [file 44318_2024_66_MOESM2_ESM.zip › Figure 1/E-230407-PCNA-Ub-Rad18-Knowkdown/RAD18-KNOCKDOWN.tif]

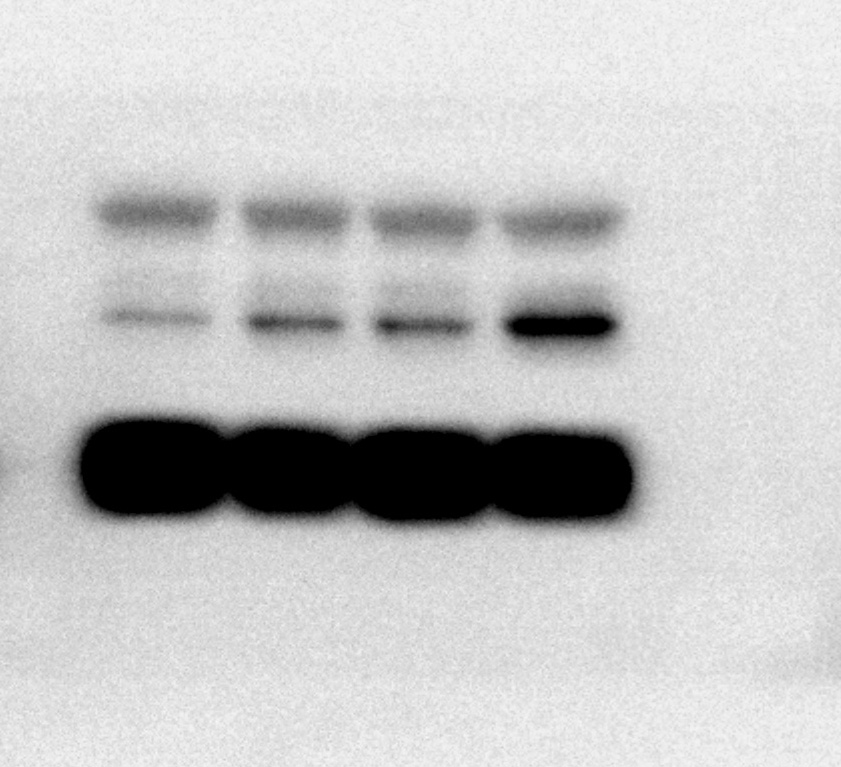

Supplement: Supplementary file 2 — Source Data Fig. 2 [file 44318_2024_66_MOESM2_ESM.zip › Figure 1/A-211109-PCNA-HU-ATRi-HU+ATRi/PCNA-LONG EXPOSURE.jpg]

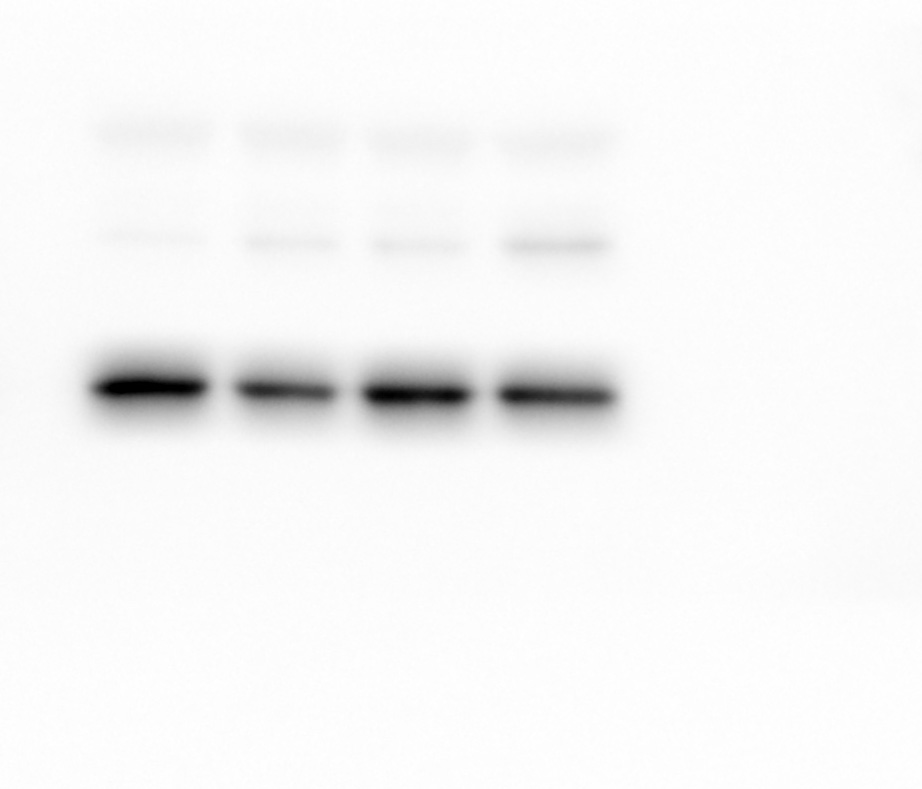

Supplement: Supplementary file 2 — Source Data Fig. 2 [file 44318_2024_66_MOESM2_ESM.zip › Figure 1/A-211109-PCNA-HU-ATRi-HU+ATRi/PCNA-SHORT EXPOSURE.jpg]

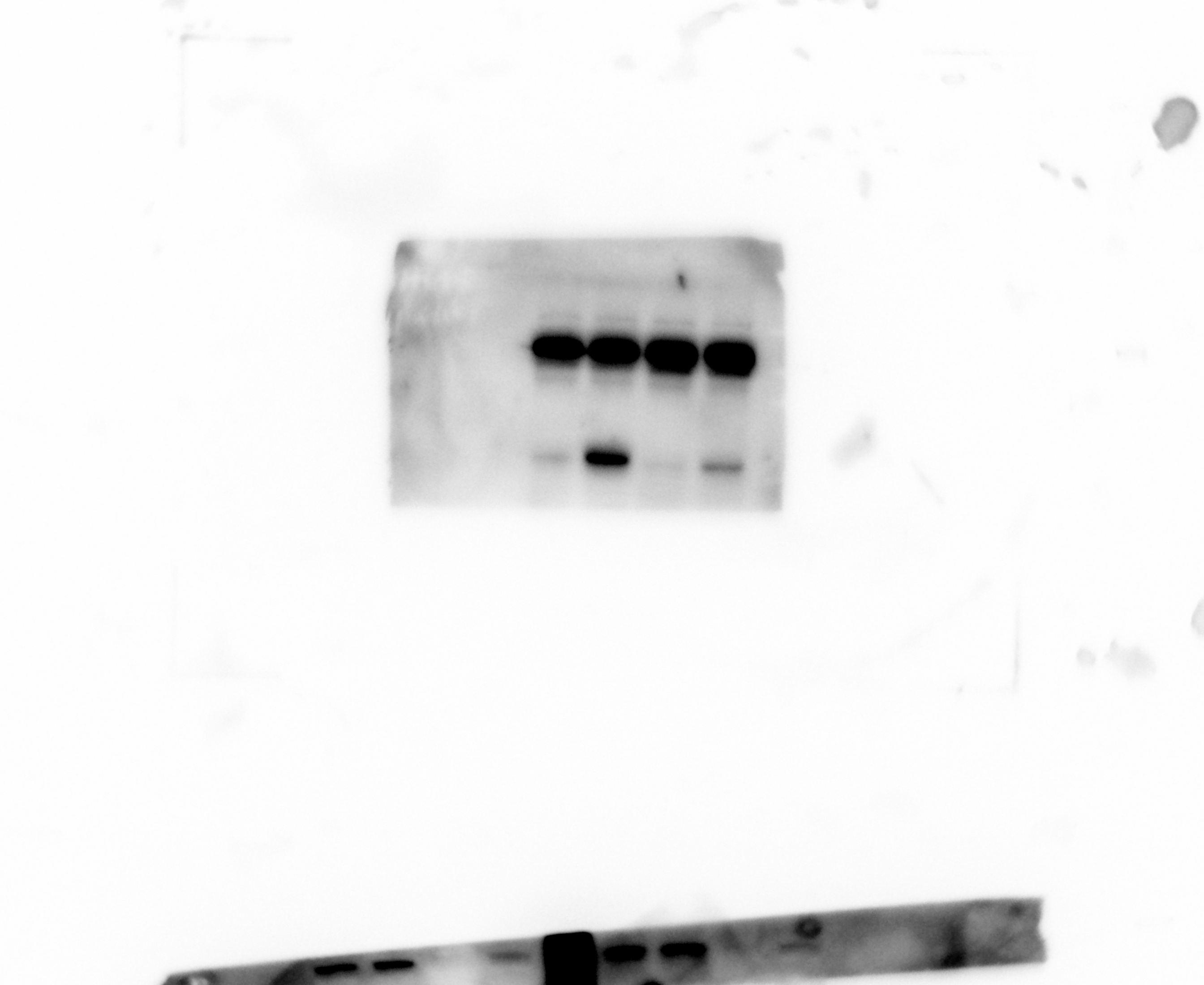

Supplement: Supplementary file 2 — Source Data Fig. 2 [file 44318_2024_66_MOESM2_ESM.zip › Figure 1/A-211109-PCNA-HU-ATRi-HU+ATRi/pSer345-Chk1.jpg]

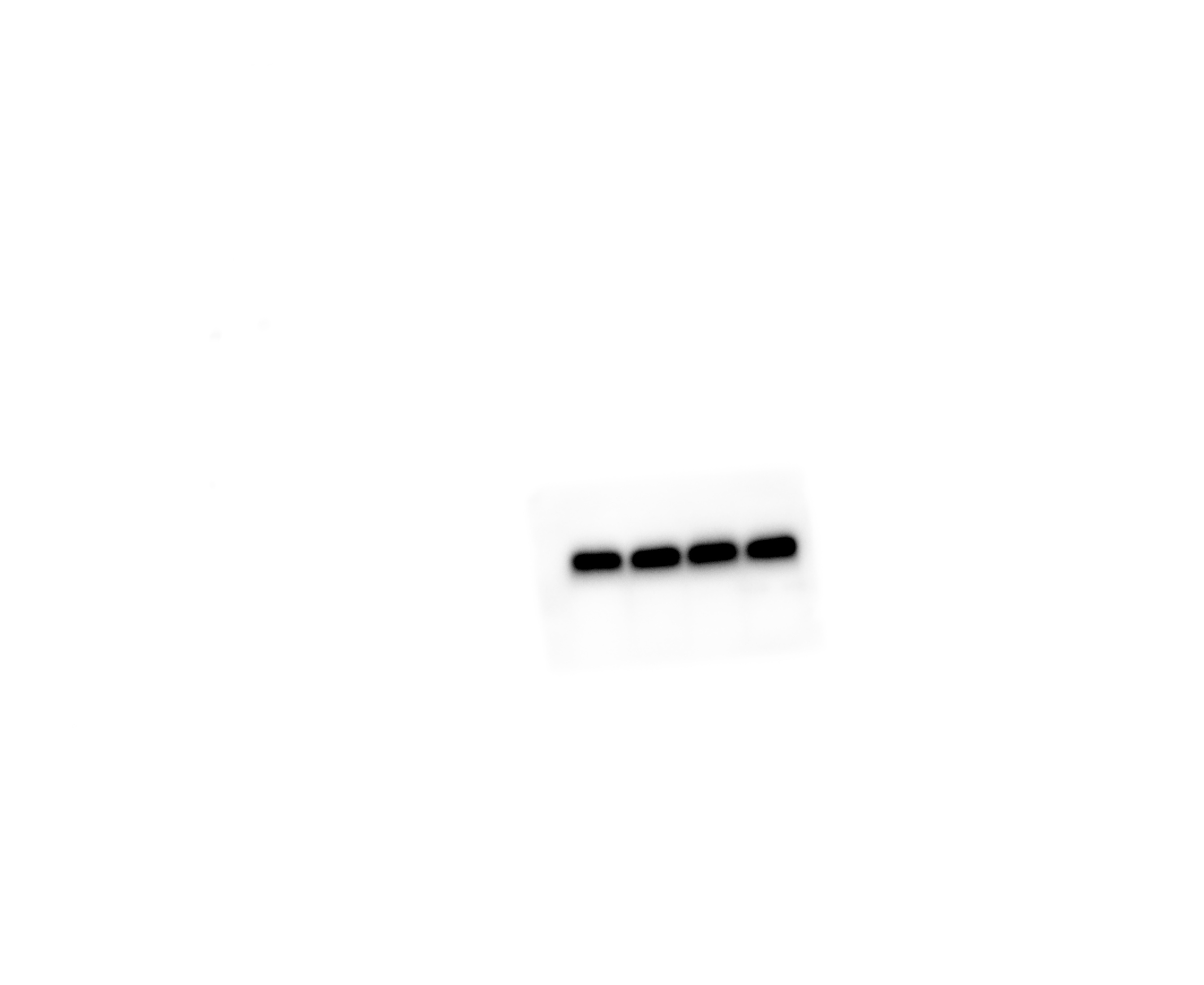

Supplement: Supplementary file 2 — Source Data Fig. 2 [file 44318_2024_66_MOESM2_ESM.zip › Figure 1/A-211109-PCNA-HU-ATRi-HU+ATRi/H3.jpg]

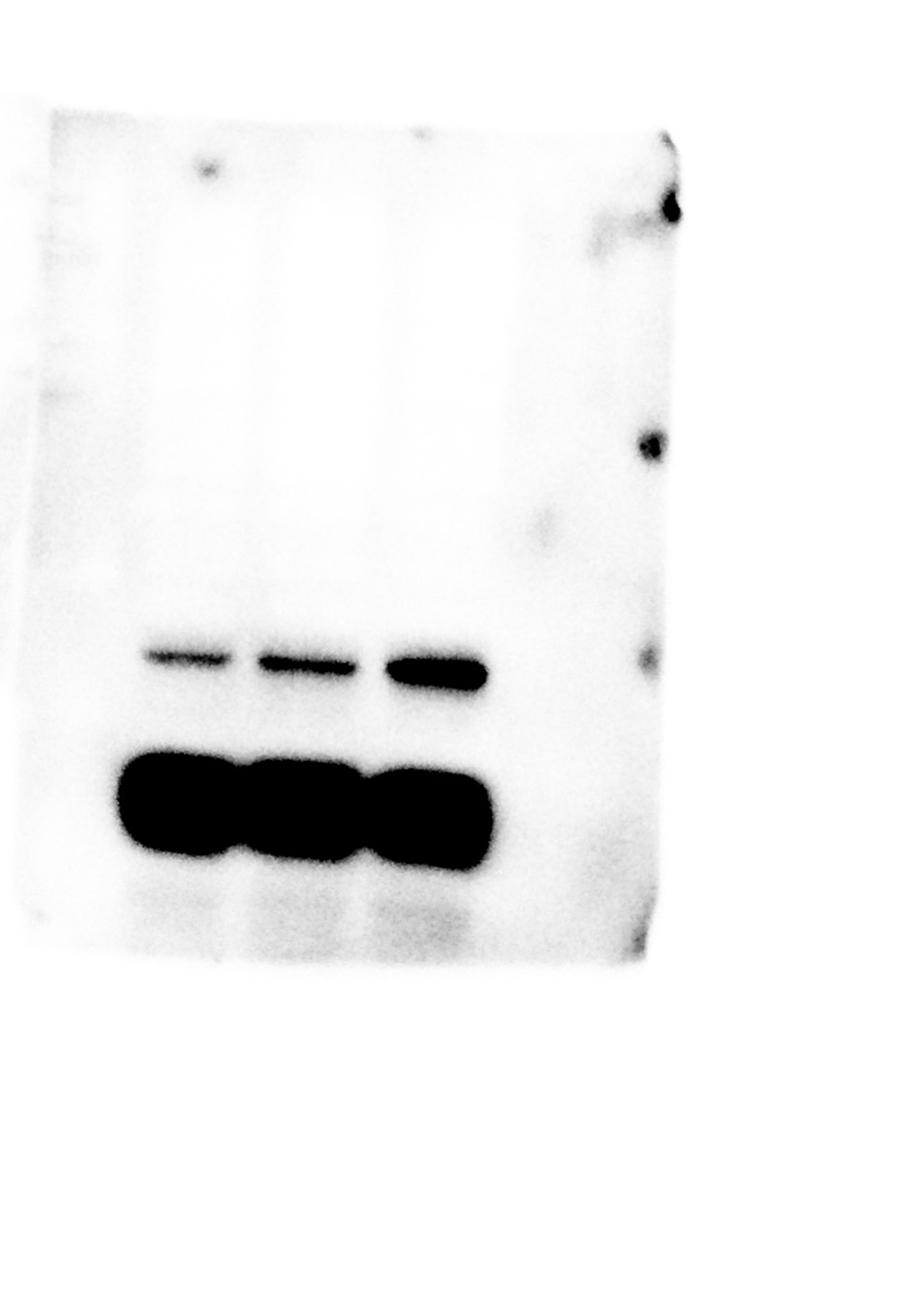

Supplement: Supplementary file 2 — Source Data Fig. 2 [file 44318_2024_66_MOESM2_ESM.zip › Figure 1/D-230418-PCNA-Ub-UV/pcna-uv-long.jpg]

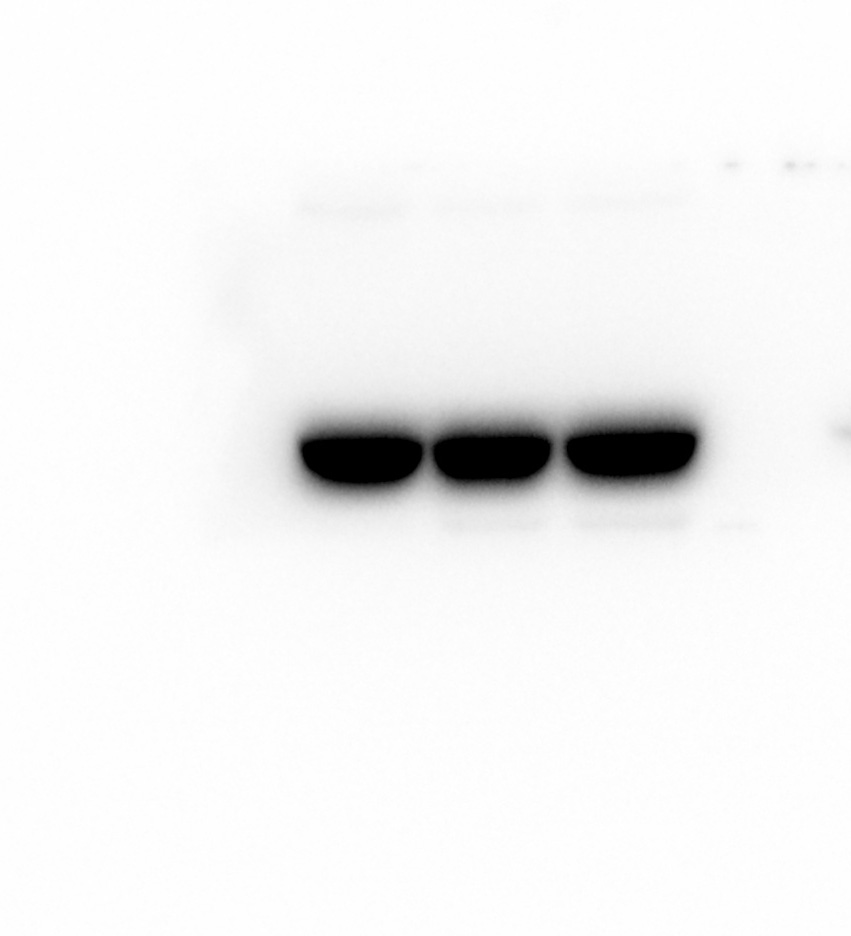

Supplement: Supplementary file 2 — Source Data Fig. 2 [file 44318_2024_66_MOESM2_ESM.zip › Figure 1/D-230418-PCNA-Ub-UV/H3.jpg]

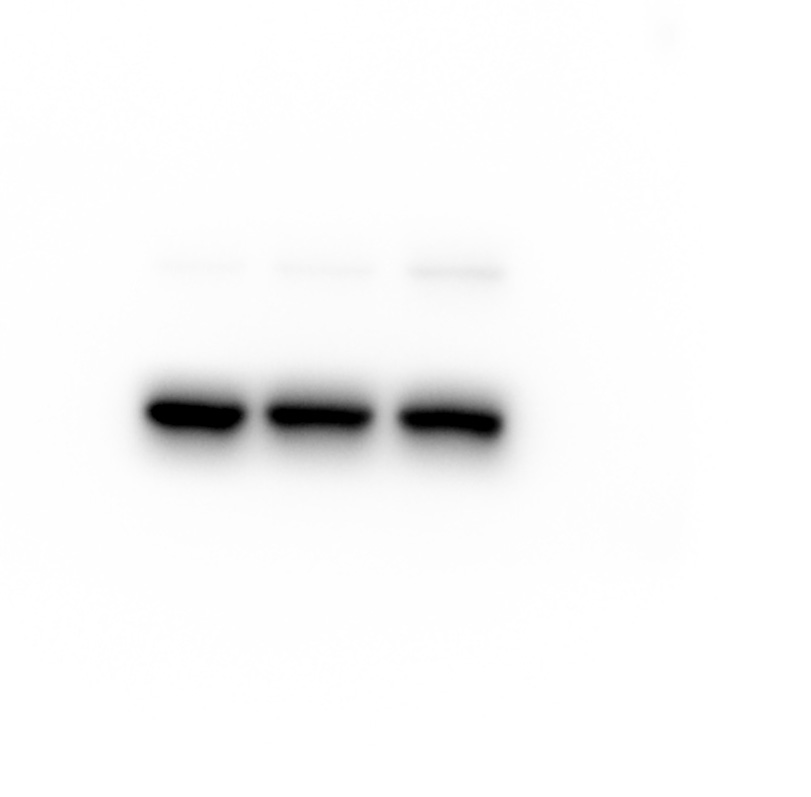

Supplement: Supplementary file 2 — Source Data Fig. 2 [file 44318_2024_66_MOESM2_ESM.zip › Figure 1/D-230418-PCNA-Ub-UV/pcna-uv-short.jpg]

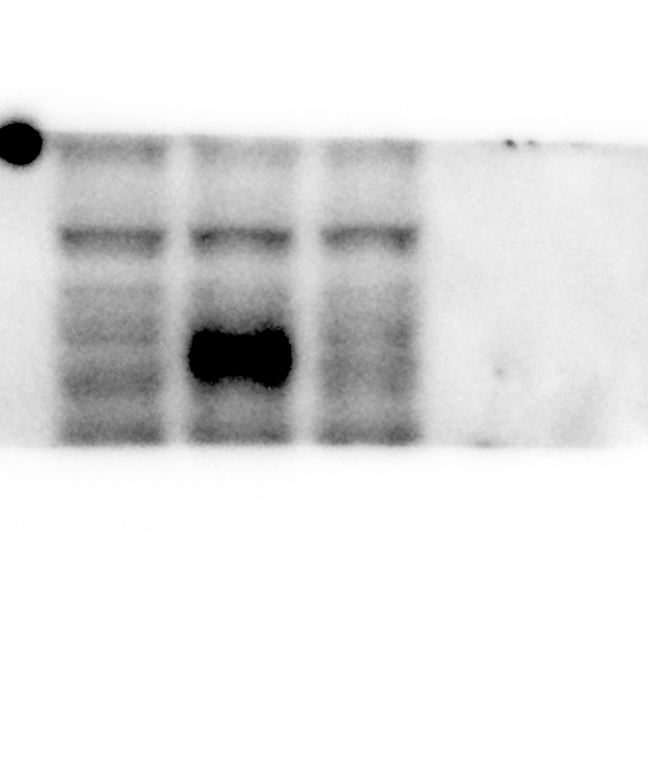

Supplement: Supplementary file 2 — Source Data Fig. 2 [file 44318_2024_66_MOESM2_ESM.zip › Figure 1/D-230418-PCNA-Ub-UV/pSer345-Chk1.tif]

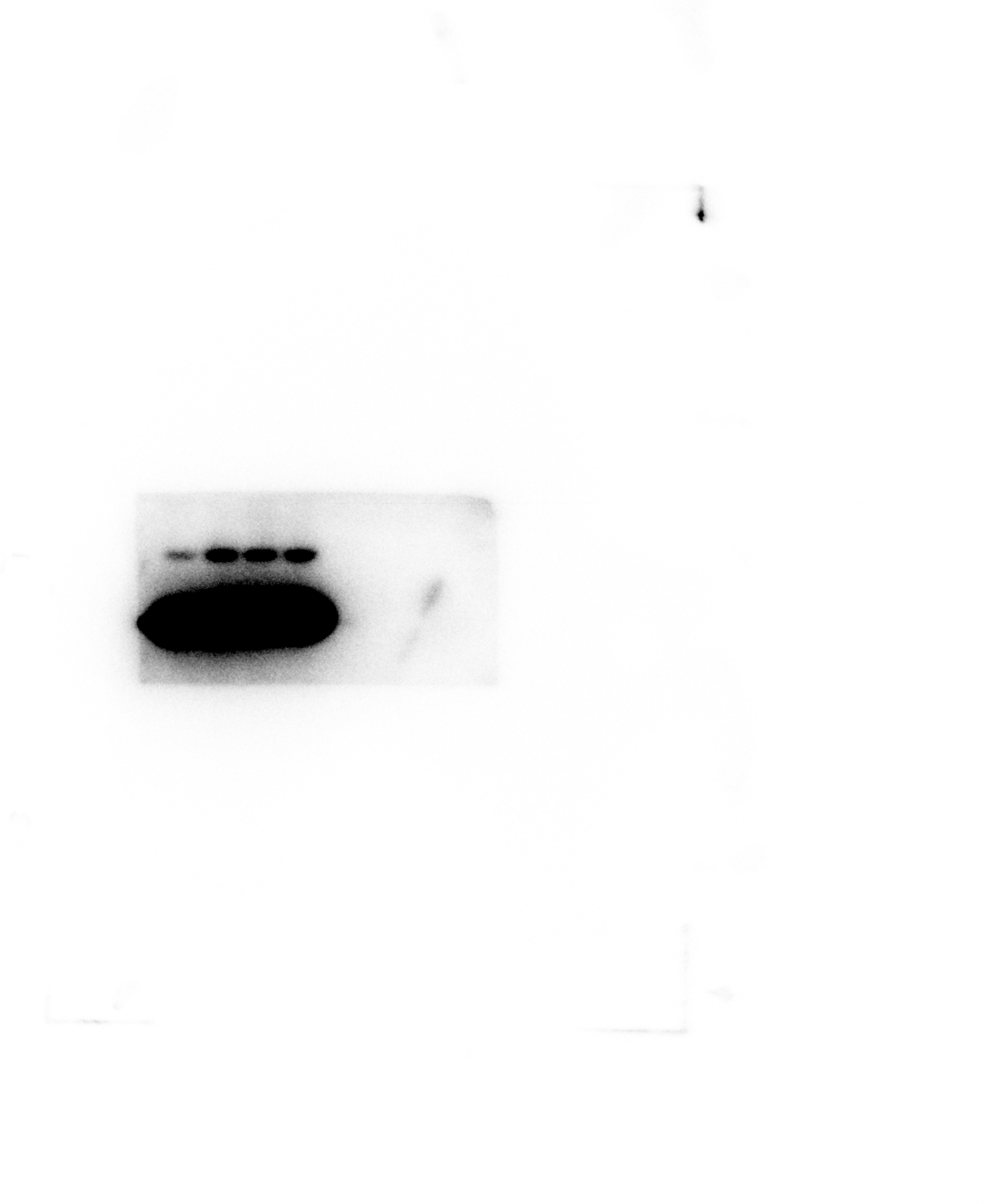

Supplement: Supplementary file 2 — Source Data Fig. 2 [file 44318_2024_66_MOESM2_ESM.zip › Figure 1/G-PCNA-Ub-CDC7i/PCNA-CDC7i-long.tif]

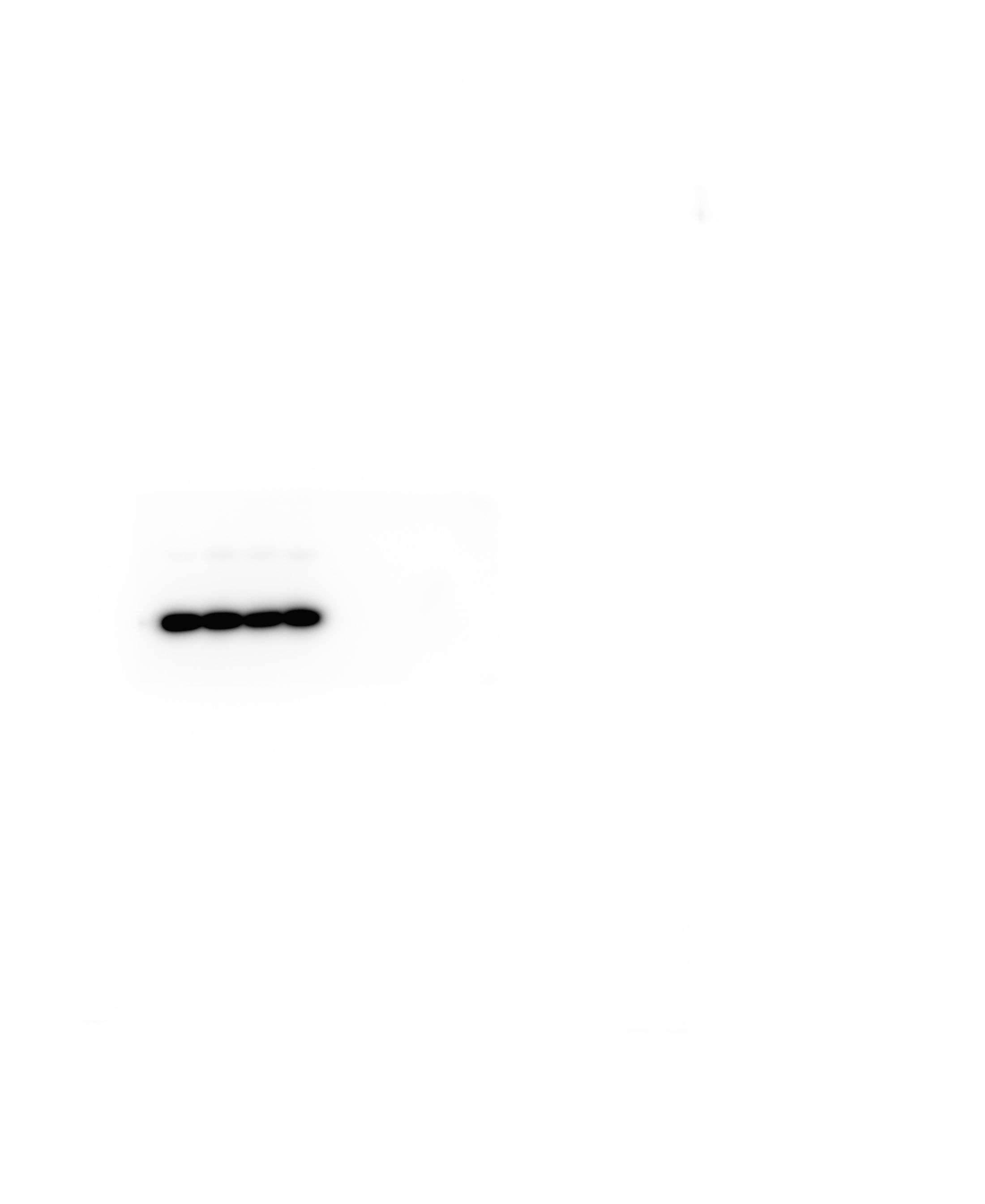

Supplement: Supplementary file 2 — Source Data Fig. 2 [file 44318_2024_66_MOESM2_ESM.zip › Figure 1/G-PCNA-Ub-CDC7i/PCNA-CDC7-1-short.tif]

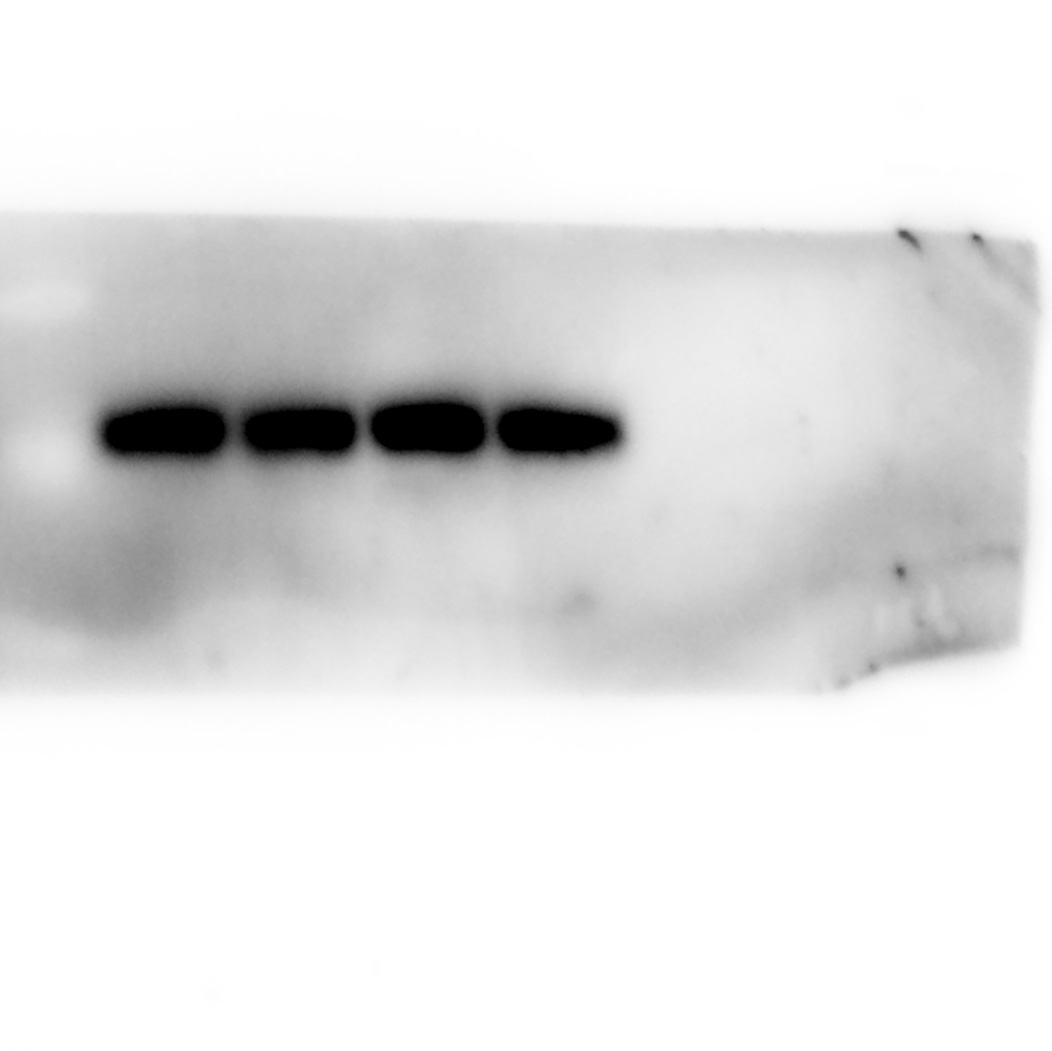

Supplement: Supplementary file 2 — Source Data Fig. 2 [file 44318_2024_66_MOESM2_ESM.zip › Figure 1/G-PCNA-Ub-CDC7i/H3-CDC7i.jpg]

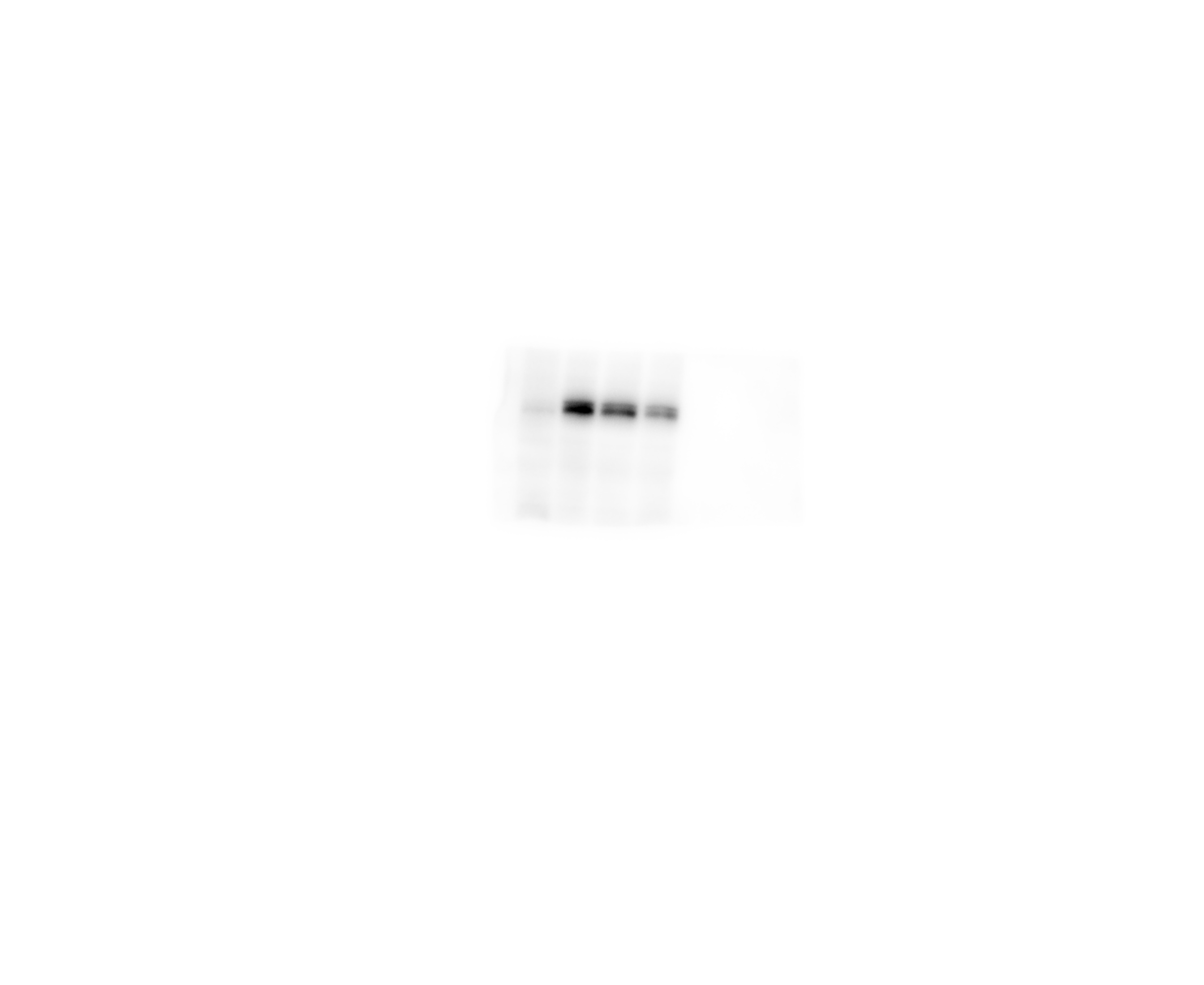

Supplement: Supplementary file 2 — Source Data Fig. 2 [file 44318_2024_66_MOESM2_ESM.zip › Figure 1/G-PCNA-Ub-CDC7i/pSer40-mcm2.jpg]

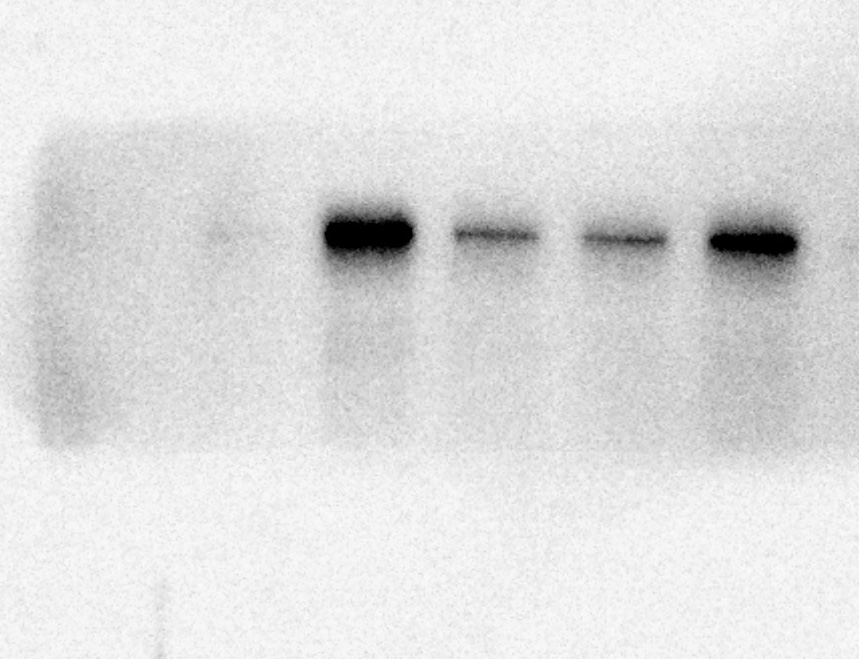

Supplement: Supplementary file 3 — Source Data Fig. 3 [file 44318_2024_66_MOESM3_ESM.zip › Figure 2/B-210916-P-ATM-RESCUE/P-Ser345-ATM.jpg]

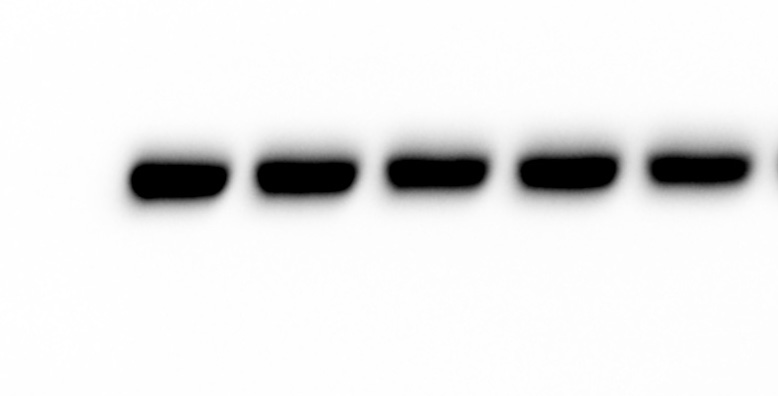

Supplement: Supplementary file 3 — Source Data Fig. 3 [file 44318_2024_66_MOESM3_ESM.zip › Figure 2/B-210916-P-ATM-RESCUE/TUBULIN.jpg]

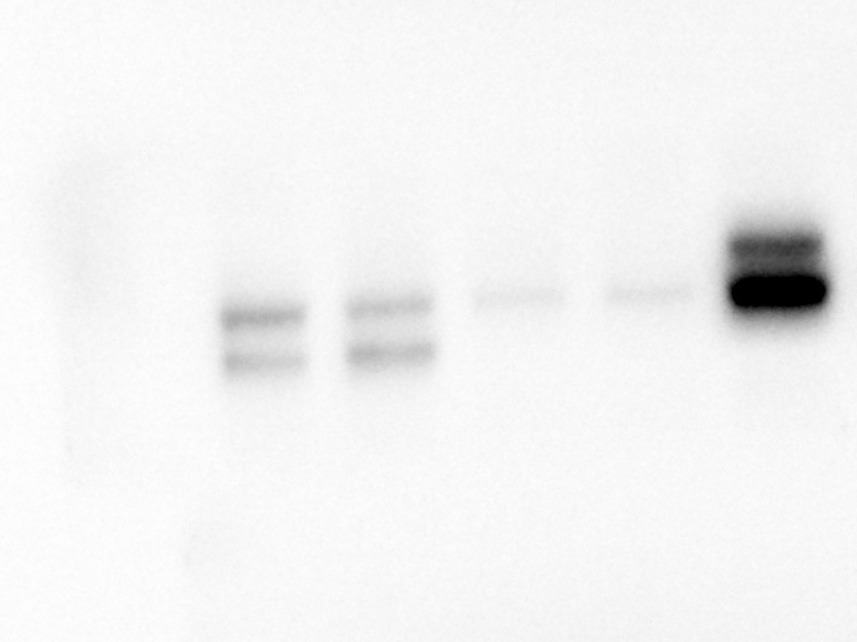

Supplement: Supplementary file 3 — Source Data Fig. 3 [file 44318_2024_66_MOESM3_ESM.zip › Figure 2/B-210916-P-ATM-RESCUE/RAD18-knockdown.jpg]

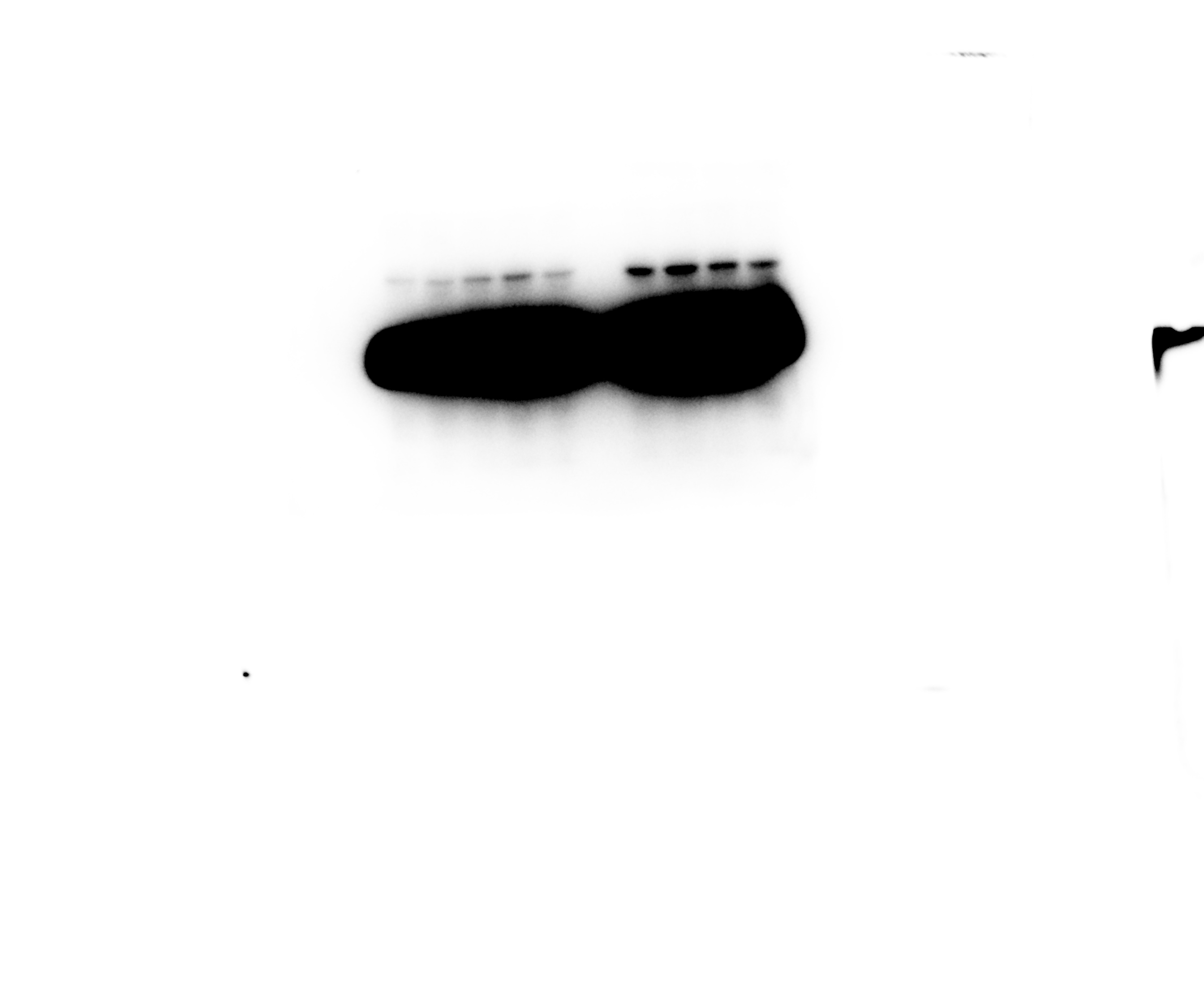

Supplement: Supplementary file 3 — Source Data Fig. 3 [file 44318_2024_66_MOESM3_ESM.zip › Figure 2/A-p-ATM-time course/PCNA-LONG.jpg]

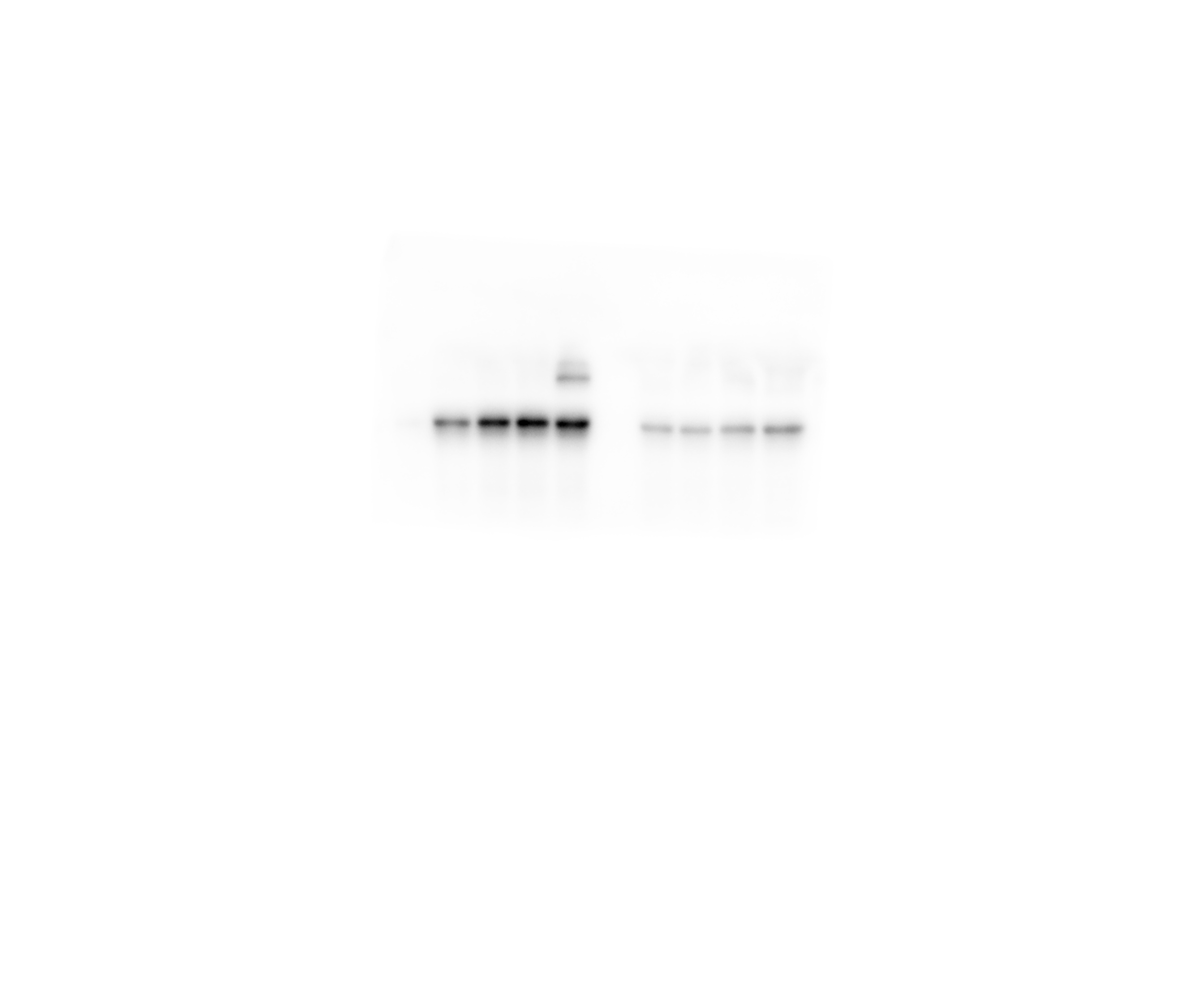

Supplement: Supplementary file 3 — Source Data Fig. 3 [file 44318_2024_66_MOESM3_ESM.zip › Figure 2/A-p-ATM-time course/pSer345-Chk1.jpg]

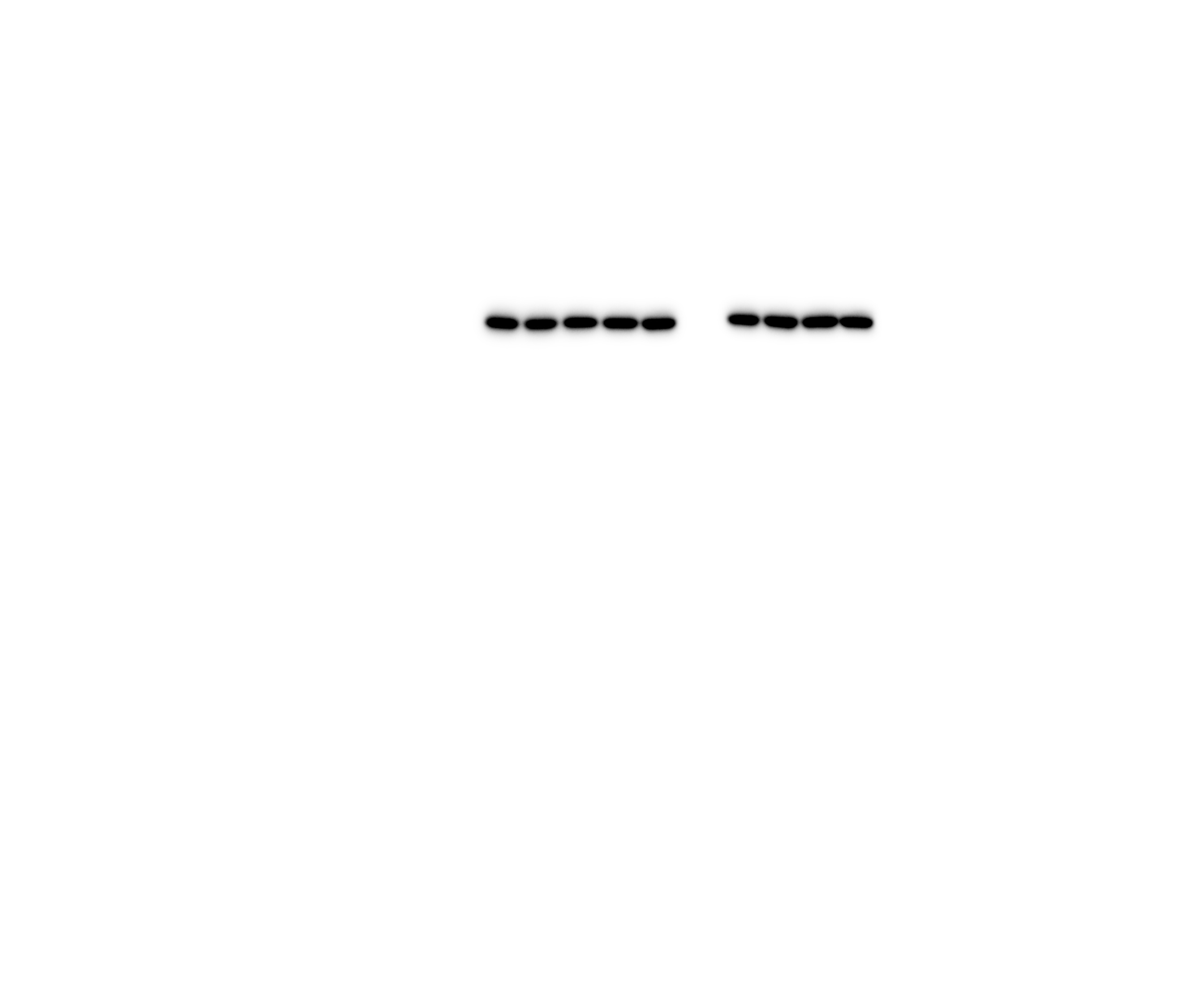

Supplement: Supplementary file 3 — Source Data Fig. 3 [file 44318_2024_66_MOESM3_ESM.zip › Figure 2/A-p-ATM-time course/H3.jpg]

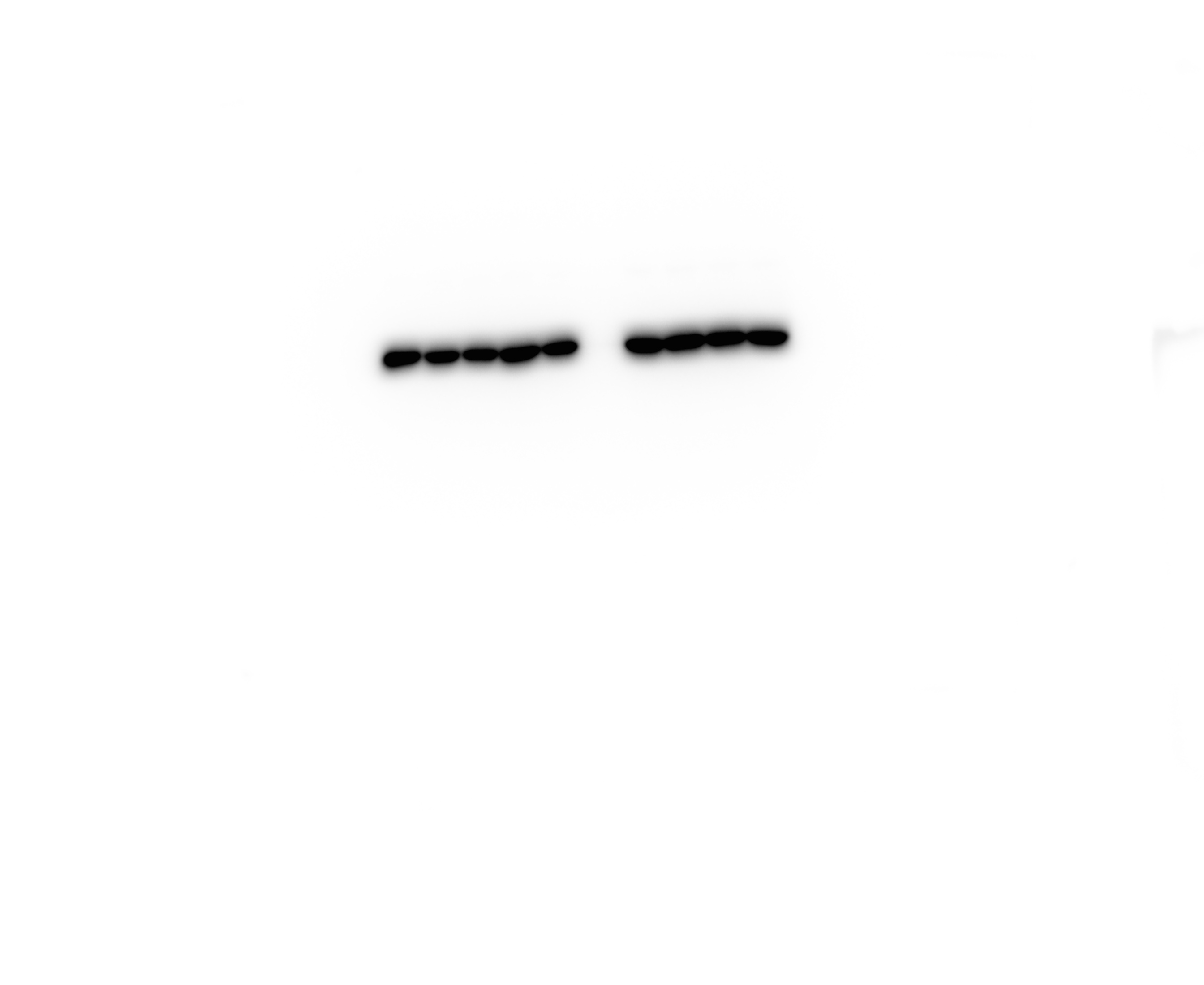

Supplement: Supplementary file 3 — Source Data Fig. 3 [file 44318_2024_66_MOESM3_ESM.zip › Figure 2/A-p-ATM-time course/PCNA-SHORT.jpg]

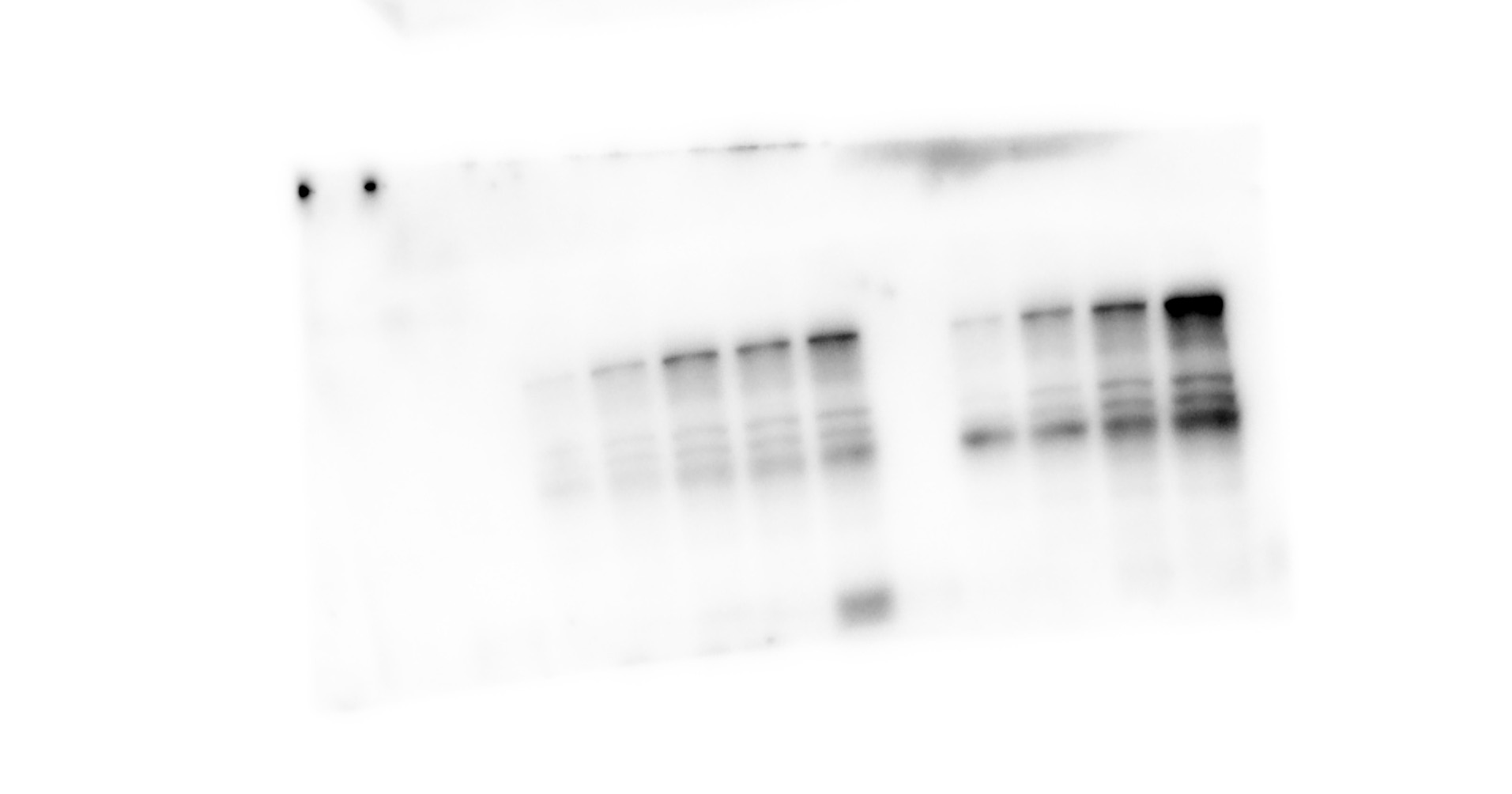

Supplement: Supplementary file 3 — Source Data Fig. 3 [file 44318_2024_66_MOESM3_ESM.zip › Figure 2/A-p-ATM-time course/pSer1981-ATM.jpg]

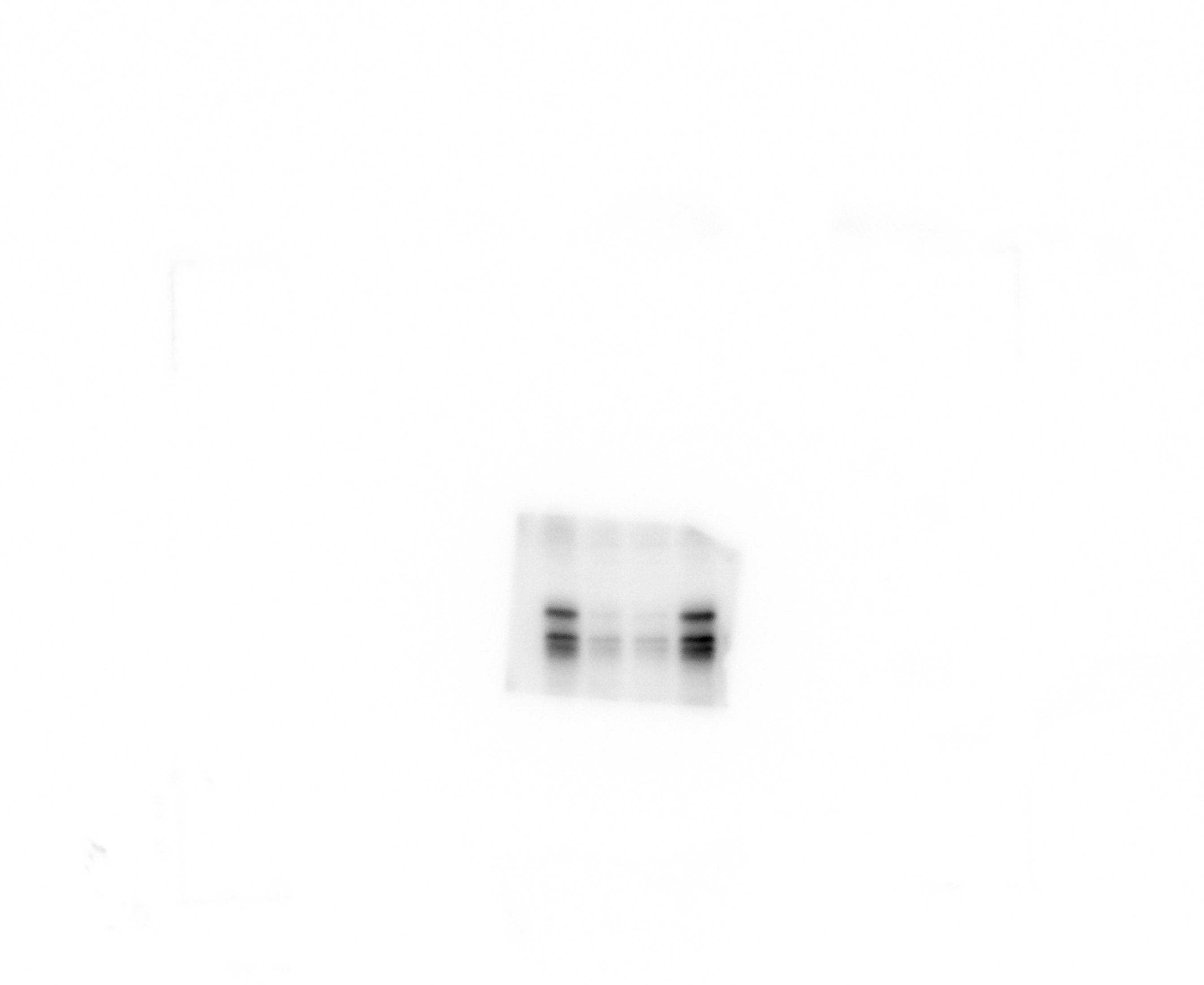

Supplement: Supplementary file 3 — Source Data Fig. 3 [file 44318_2024_66_MOESM3_ESM.zip › Figure 2/F-Rad18-knockdown/Rad18-kd.jpg]

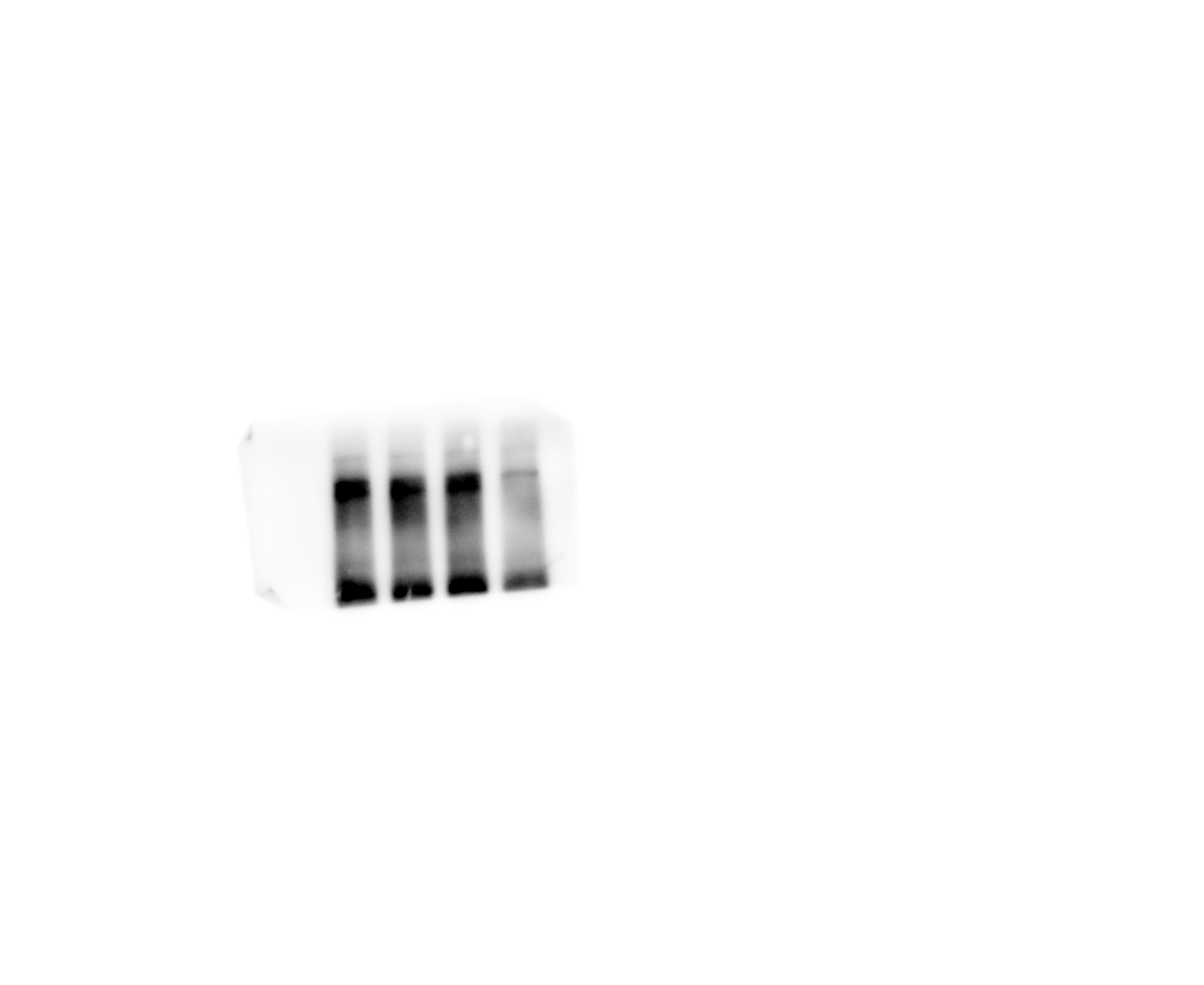

Supplement: Supplementary file 3 — Source Data Fig. 3 [file 44318_2024_66_MOESM3_ESM.zip › Figure 2/F-Rad18-knockdown/SLX4-KD.jpg]

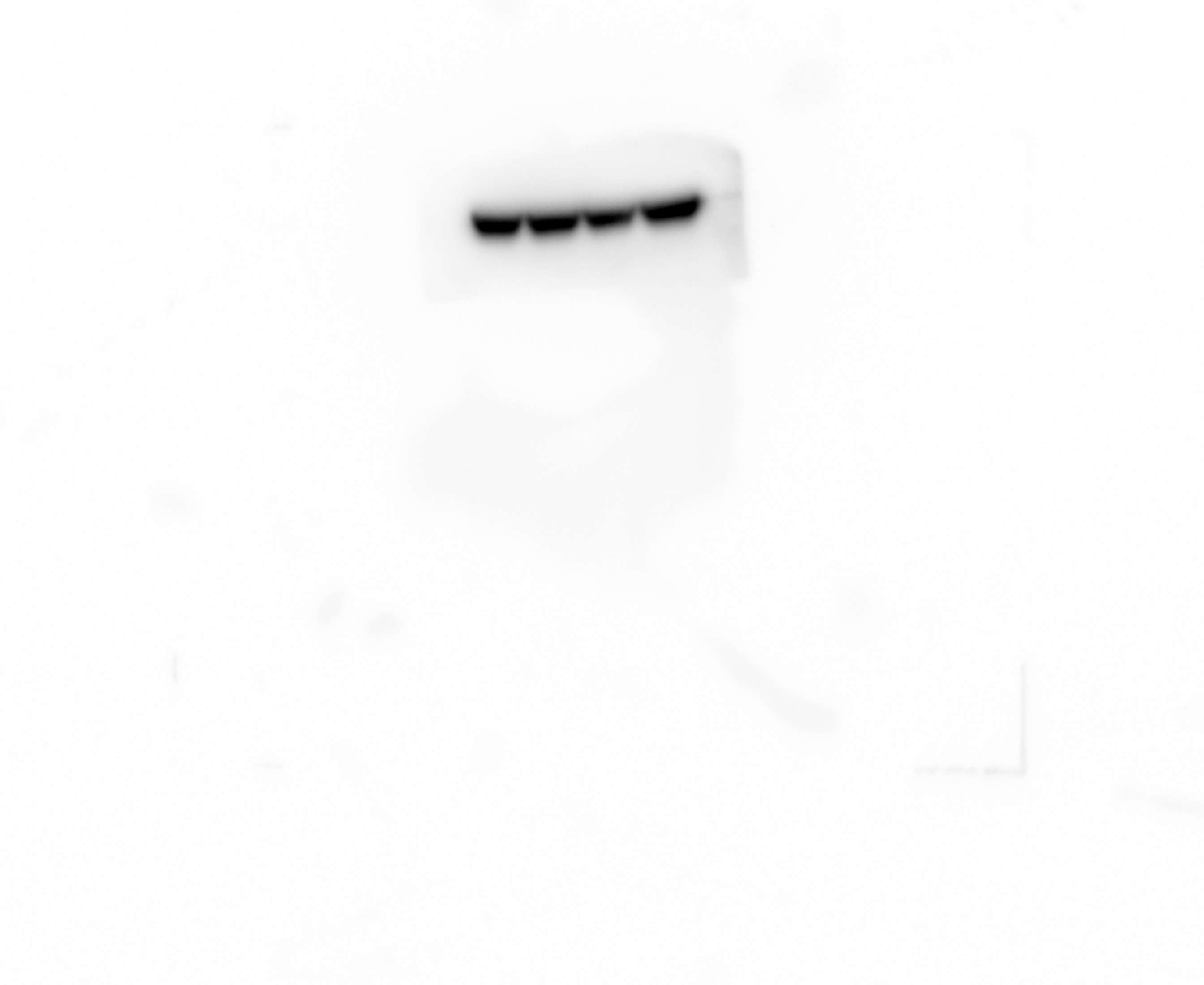

Supplement: Supplementary file 3 — Source Data Fig. 3 [file 44318_2024_66_MOESM3_ESM.zip › Figure 2/F-Rad18-knockdown/TUBULIN.jpg]

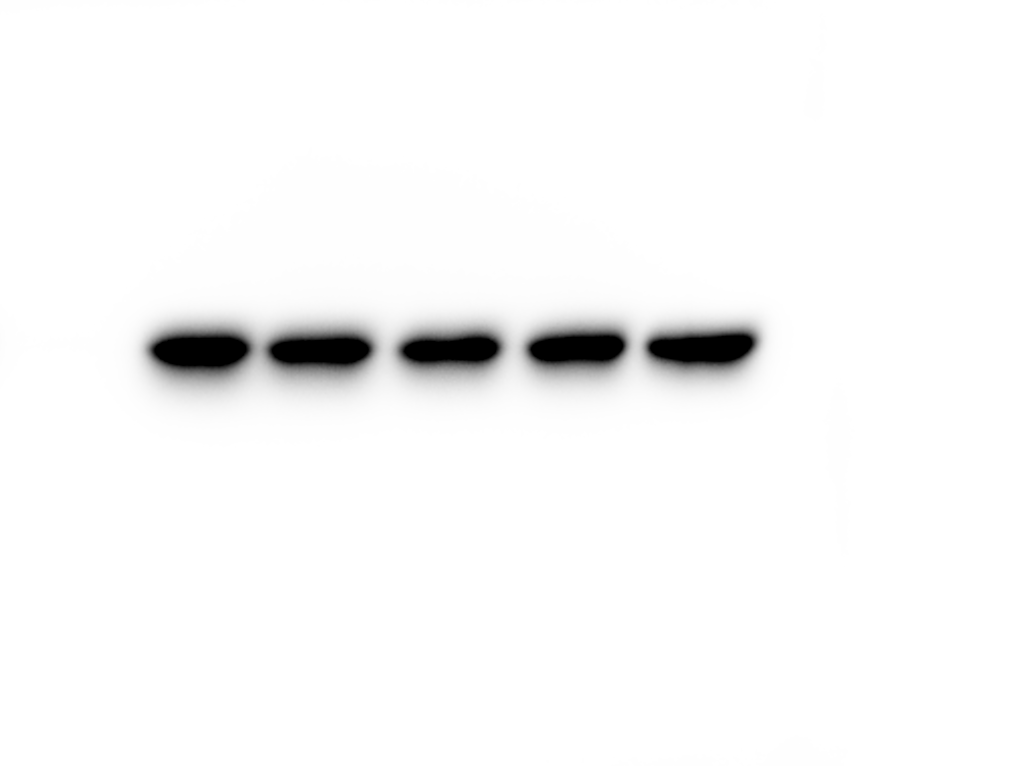

Supplement: Supplementary file 3 — Source Data Fig. 3 [file 44318_2024_66_MOESM3_ESM.zip › Figure 2/I-230407-Rad18-KD/Tubulin.tif]

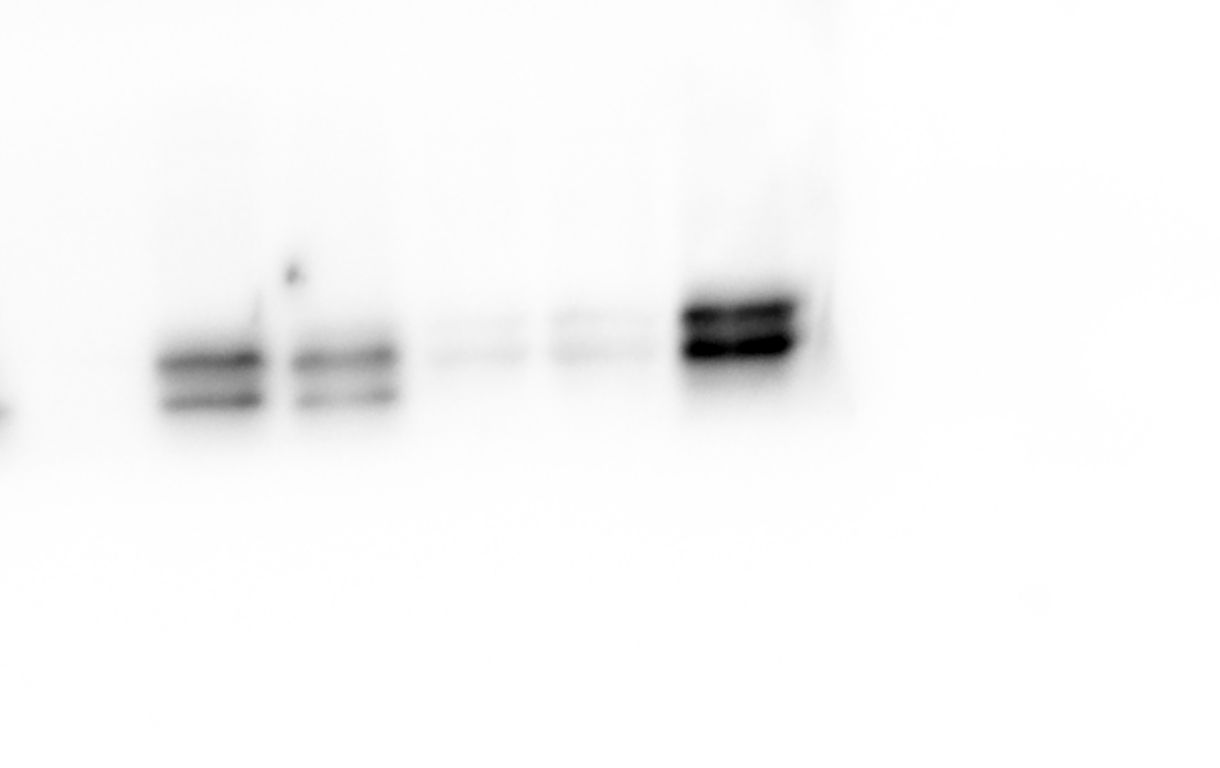

Supplement: Supplementary file 3 — Source Data Fig. 3 [file 44318_2024_66_MOESM3_ESM.zip › Figure 2/I-230407-Rad18-KD/RAD18-KD.tif]

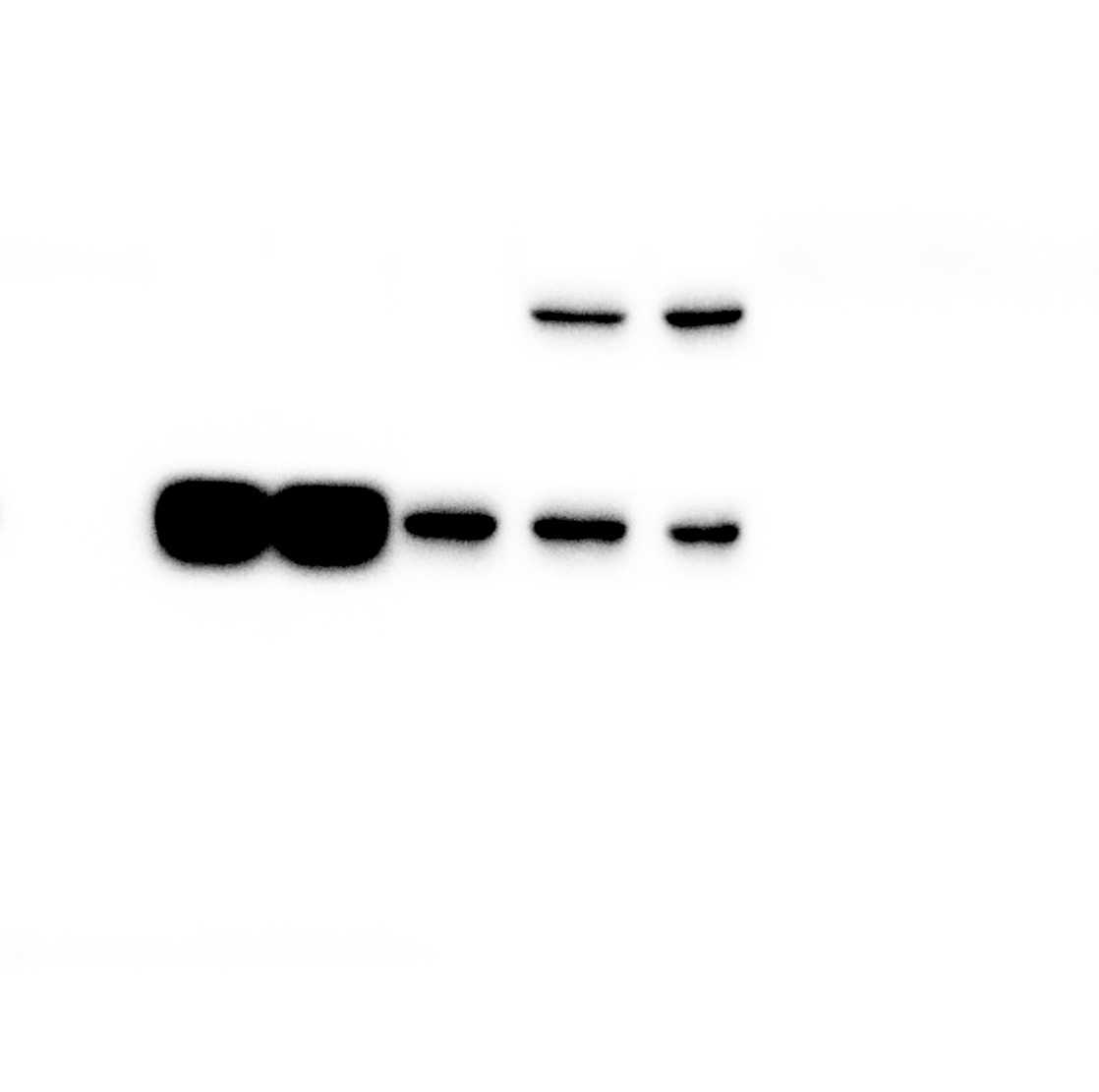

Supplement: Supplementary file 3 — Source Data Fig. 3 [file 44318_2024_66_MOESM3_ESM.zip › Figure 2/C-220628-PCNA-KR-pATM/PCNA.jpg]

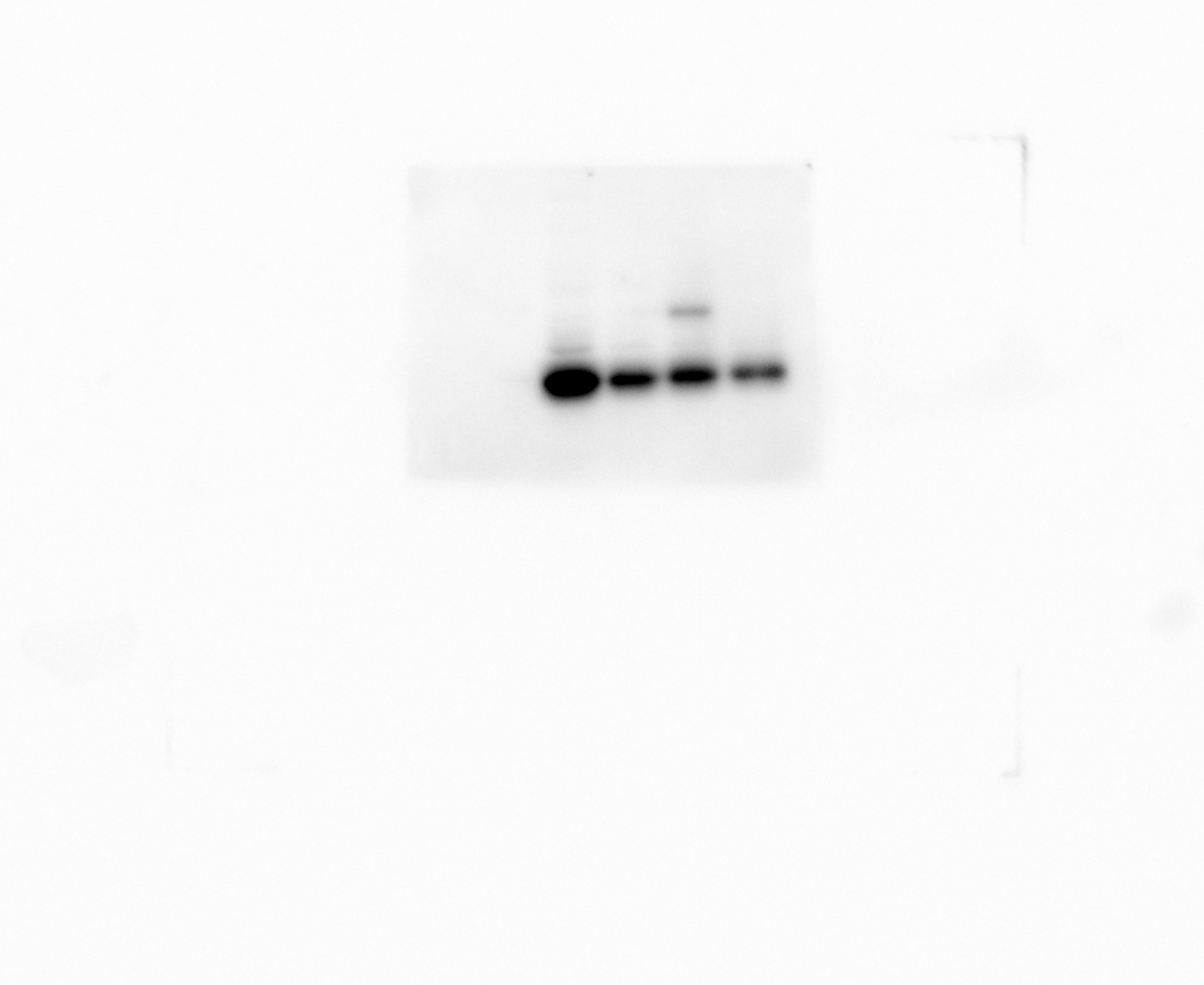

Supplement: Supplementary file 3 — Source Data Fig. 3 [file 44318_2024_66_MOESM3_ESM.zip › Figure 2/C-220628-PCNA-KR-pATM/PCNA-K164-UB-WT-KR.jpg]

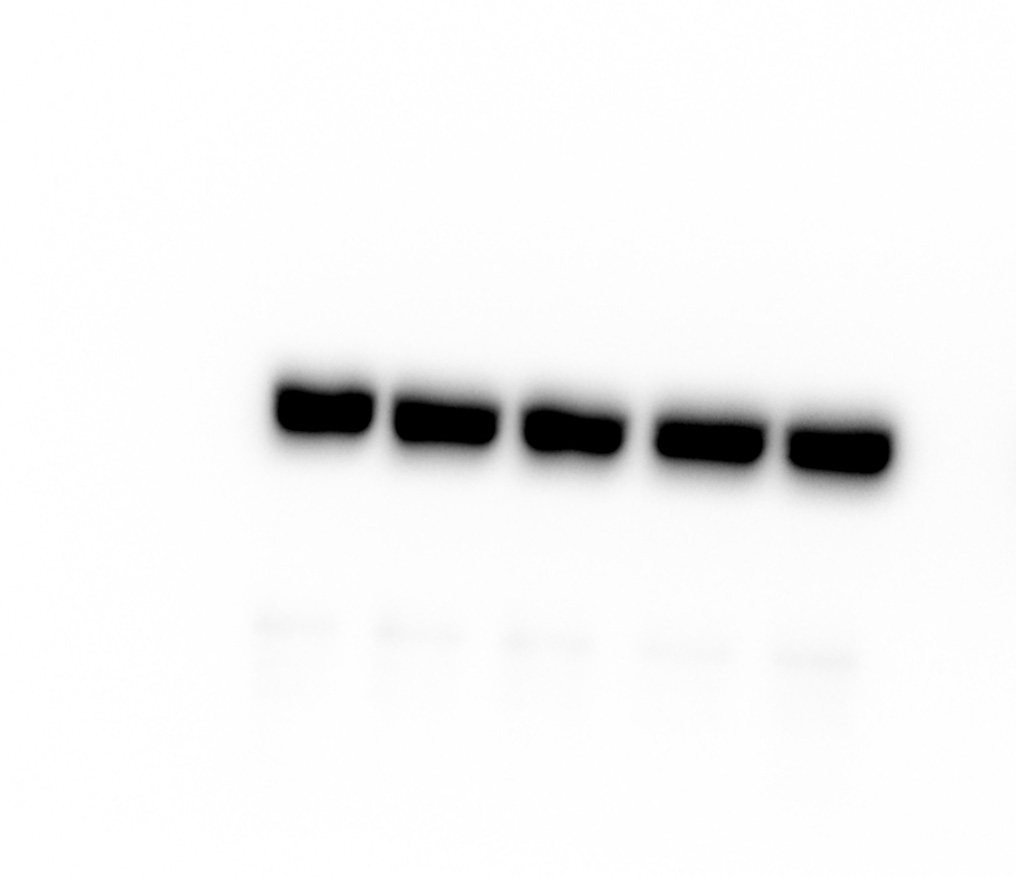

Supplement: Supplementary file 3 — Source Data Fig. 3 [file 44318_2024_66_MOESM3_ESM.zip › Figure 2/C-220628-PCNA-KR-pATM/GAPDH.jpg]

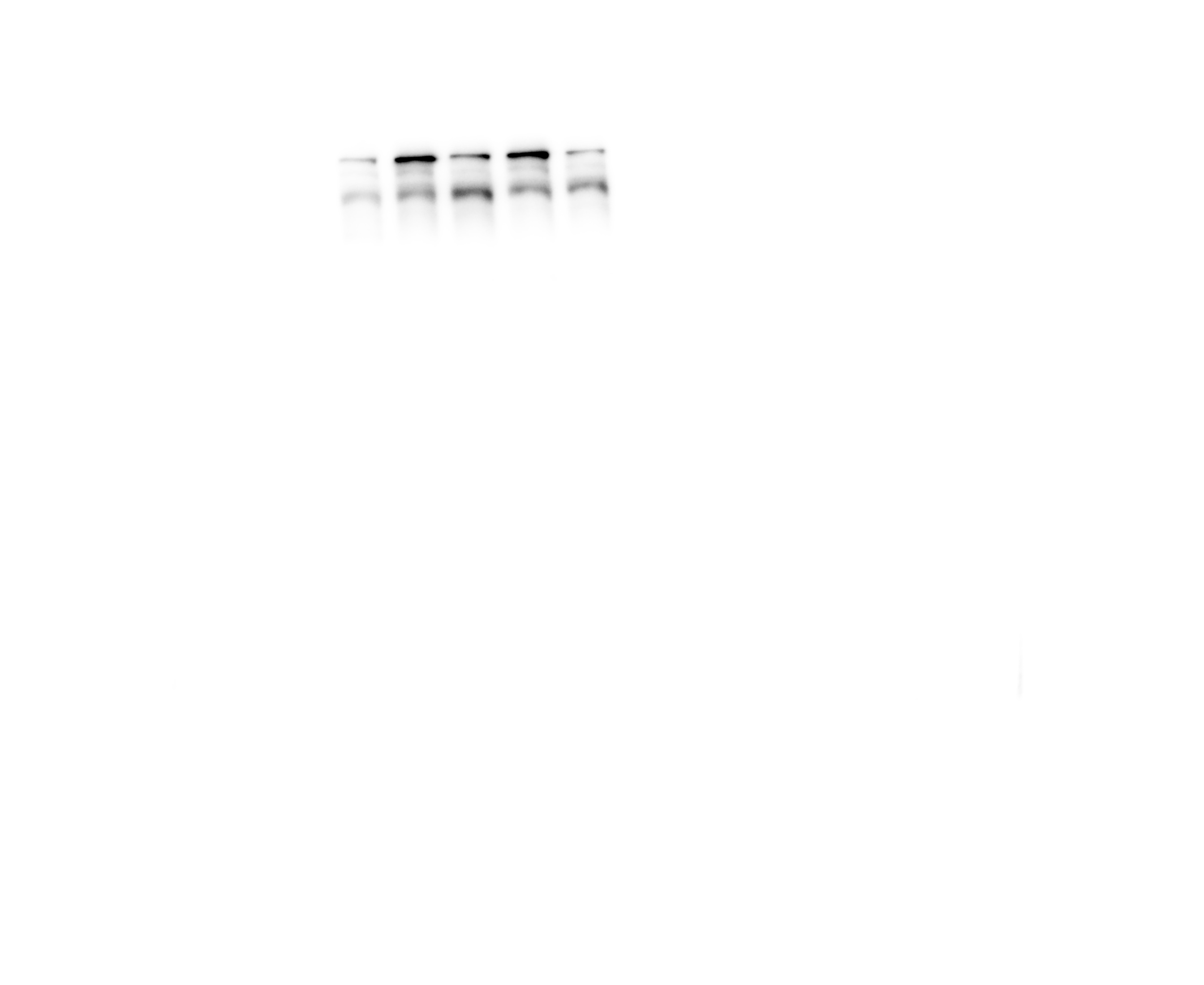

Supplement: Supplementary file 3 — Source Data Fig. 3 [file 44318_2024_66_MOESM3_ESM.zip › Figure 2/C-220628-PCNA-KR-pATM/pSer1981-ATM.jpg]

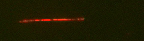

Supplement: Supplementary file 3 — Source Data Fig. 3 [file 44318_2024_66_MOESM3_ESM.zip › Figure 2/D-230720-fiber-collapsed image/Fiber-collasped fork.tif]

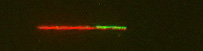

Supplement: Supplementary file 3 — Source Data Fig. 3 [file 44318_2024_66_MOESM3_ESM.zip › Figure 2/D-230720-fiber-collapsed image/Fiber-restarted fork.jpg]

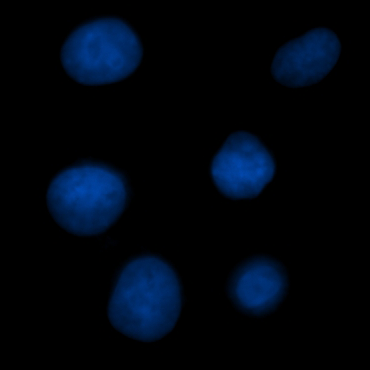

Supplement: Supplementary file 3 — Source Data Fig. 3 [file 44318_2024_66_MOESM3_ESM.zip › Figure 2/G-230228-Brdu-Foci image/Ctrl-/1-DAPI.tif]

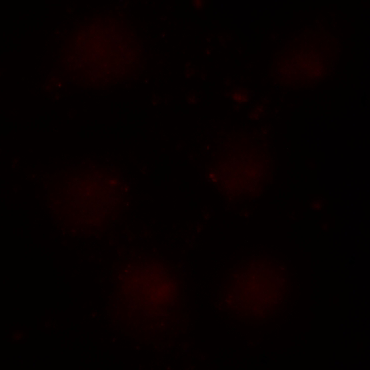

Supplement: Supplementary file 3 — Source Data Fig. 3 [file 44318_2024_66_MOESM3_ESM.zip › Figure 2/G-230228-Brdu-Foci image/Ctrl-/1-BRDU.tif]

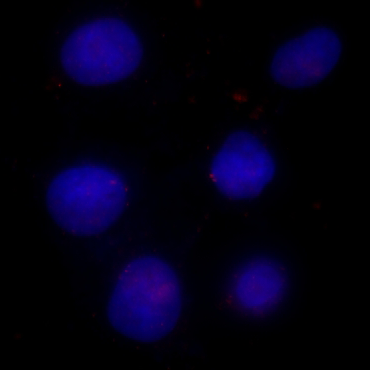

Supplement: Supplementary file 3 — Source Data Fig. 3 [file 44318_2024_66_MOESM3_ESM.zip › Figure 2/G-230228-Brdu-Foci image/Ctrl-/1-MERGE.tif]

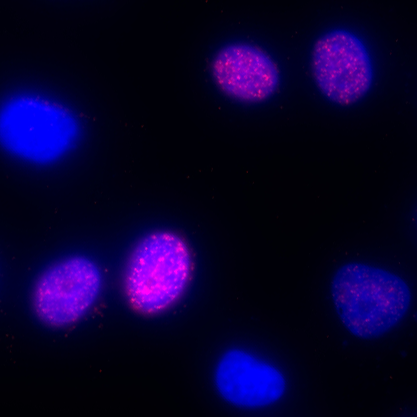

Supplement: Supplementary file 3 — Source Data Fig. 3 [file 44318_2024_66_MOESM3_ESM.zip › Figure 2/G-230228-Brdu-Foci image/Ctrl+/2-merge.tif]

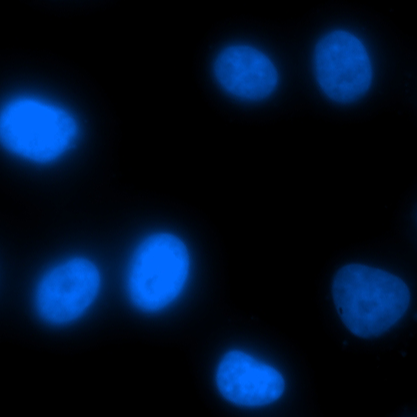

Supplement: Supplementary file 3 — Source Data Fig. 3 [file 44318_2024_66_MOESM3_ESM.zip › Figure 2/G-230228-Brdu-Foci image/Ctrl+/2-dapi.tif]

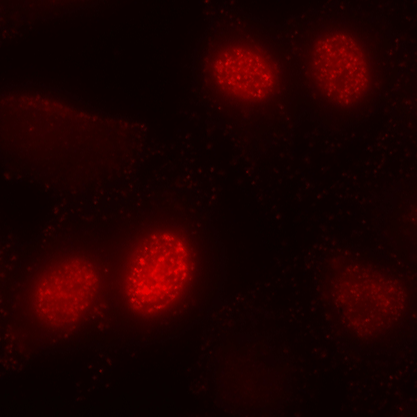

Supplement: Supplementary file 3 — Source Data Fig. 3 [file 44318_2024_66_MOESM3_ESM.zip › Figure 2/G-230228-Brdu-Foci image/Ctrl+/2-brdu.tif]

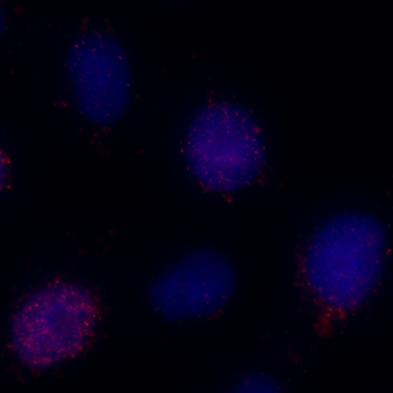

Supplement: Supplementary file 3 — Source Data Fig. 3 [file 44318_2024_66_MOESM3_ESM.zip › Figure 2/G-230228-Brdu-Foci image/siRad18#1/2-merge.tif]

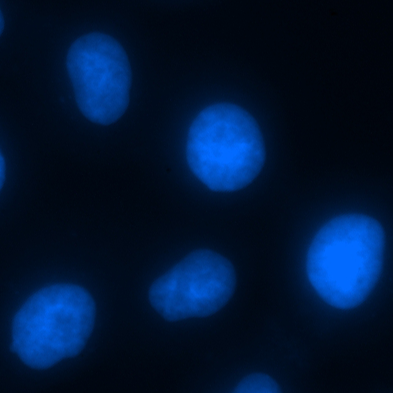

Supplement: Supplementary file 3 — Source Data Fig. 3 [file 44318_2024_66_MOESM3_ESM.zip › Figure 2/G-230228-Brdu-Foci image/siRad18#1/2-dapi.tif]

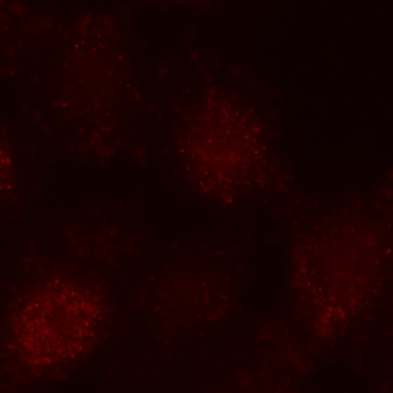

Supplement: Supplementary file 3 — Source Data Fig. 3 [file 44318_2024_66_MOESM3_ESM.zip › Figure 2/G-230228-Brdu-Foci image/siRad18#1/2-brdu.tif]

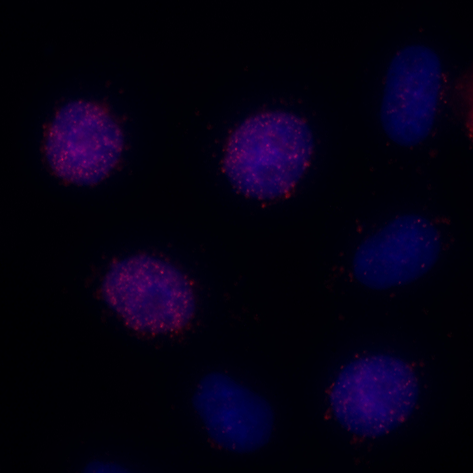

Supplement: Supplementary file 3 — Source Data Fig. 3 [file 44318_2024_66_MOESM3_ESM.zip › Figure 2/G-230228-Brdu-Foci image/siRad18#2/2-merge.tif]

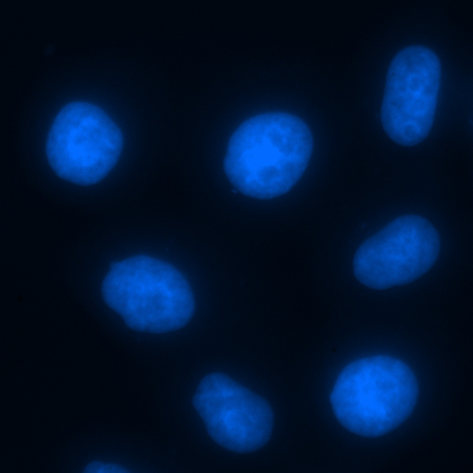

Supplement: Supplementary file 3 — Source Data Fig. 3 [file 44318_2024_66_MOESM3_ESM.zip › Figure 2/G-230228-Brdu-Foci image/siRad18#2/2-dapi.tif]

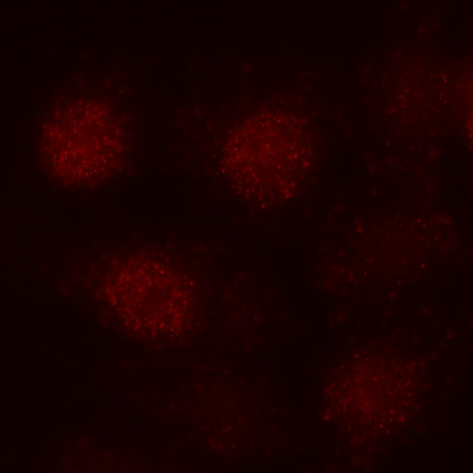

Supplement: Supplementary file 3 — Source Data Fig. 3 [file 44318_2024_66_MOESM3_ESM.zip › Figure 2/G-230228-Brdu-Foci image/siRad18#2/2-brdu.tif]

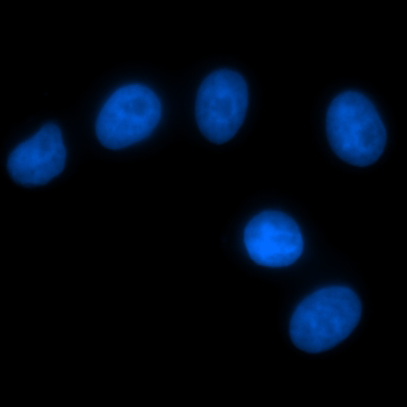

Supplement: Supplementary file 3 — Source Data Fig. 3 [file 44318_2024_66_MOESM3_ESM.zip › Figure 2/G-230228-Brdu-Foci image/siRad18#1+WT/1-dapi.tif]

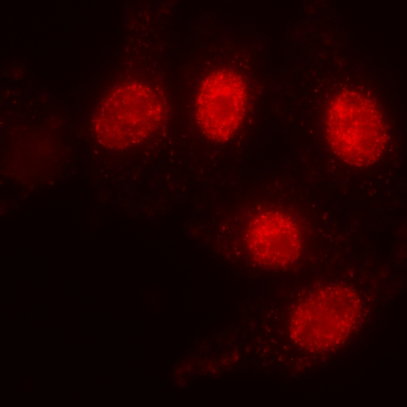

Supplement: Supplementary file 3 — Source Data Fig. 3 [file 44318_2024_66_MOESM3_ESM.zip › Figure 2/G-230228-Brdu-Foci image/siRad18#1+WT/1-brdu.tif]

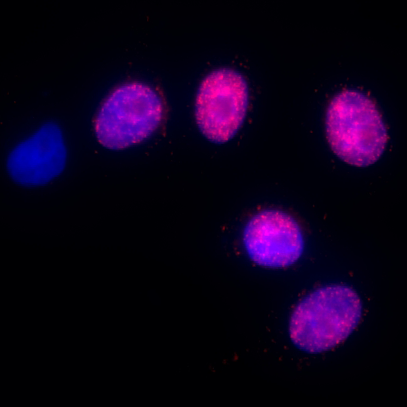

Supplement: Supplementary file 3 — Source Data Fig. 3 [file 44318_2024_66_MOESM3_ESM.zip › Figure 2/G-230228-Brdu-Foci image/siRad18#1+WT/1-merge.tif]

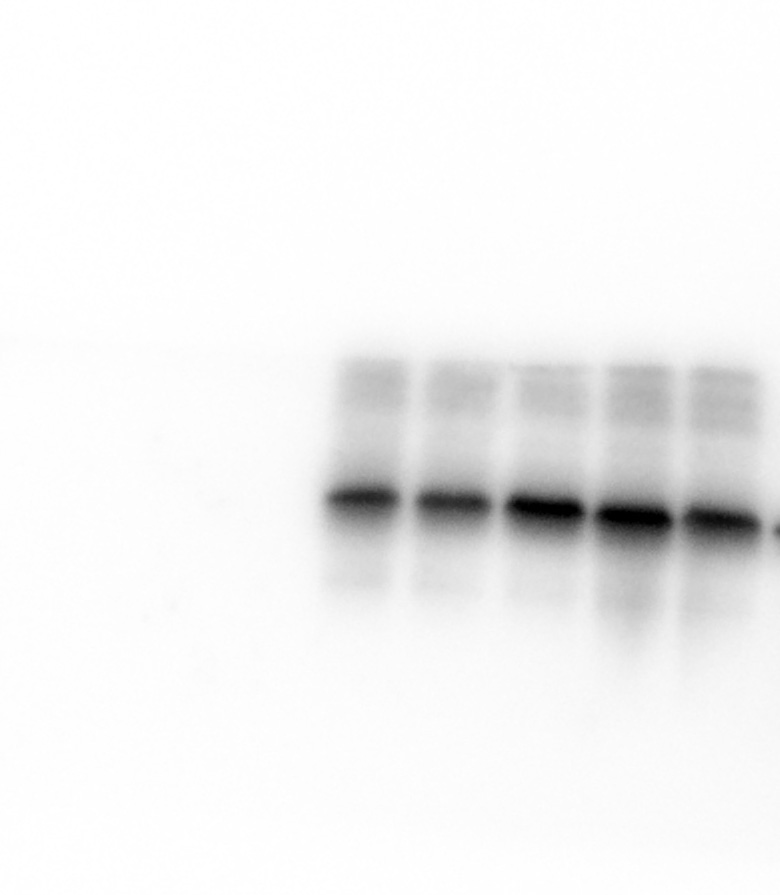

Supplement: Supplementary file 4 — Source Data Fig. 4 [file 44318_2024_66_MOESM4_ESM.zip › Figure 3/H-230826-Rad18-over-PCNA-Ub/FLAG.jpg]

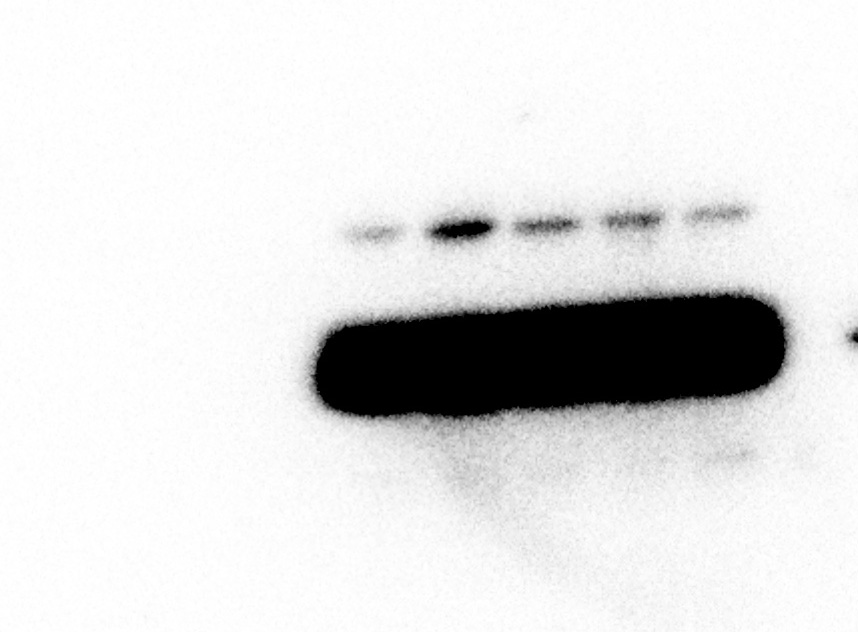

Supplement: Supplementary file 4 — Source Data Fig. 4 [file 44318_2024_66_MOESM4_ESM.zip › Figure 3/H-230826-Rad18-over-PCNA-Ub/PCNA-LONG-UB.jpg]

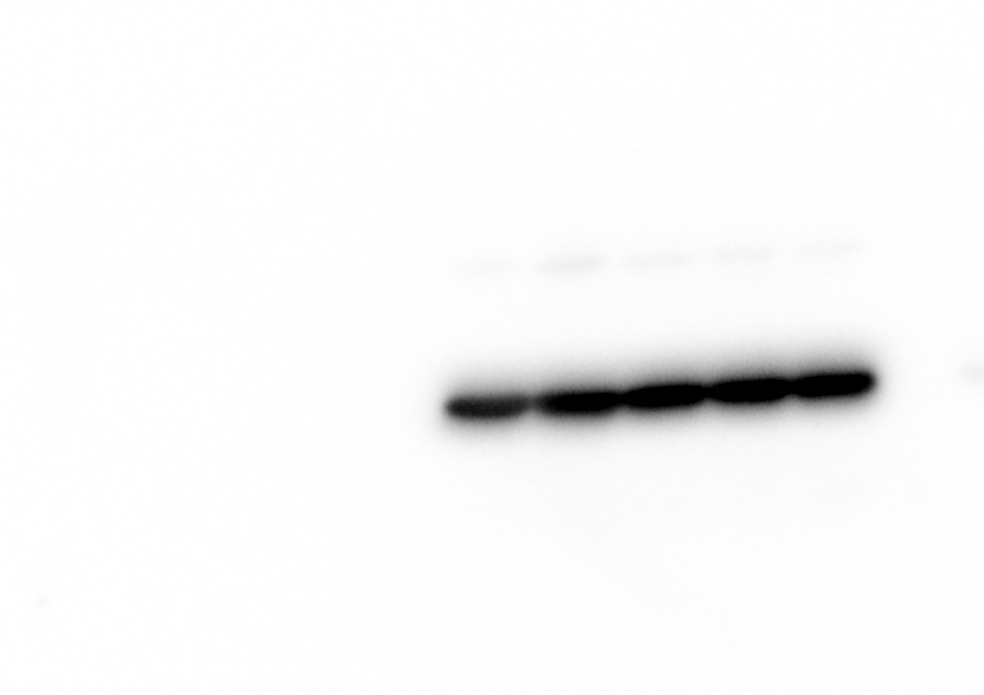

Supplement: Supplementary file 4 — Source Data Fig. 4 [file 44318_2024_66_MOESM4_ESM.zip › Figure 3/H-230826-Rad18-over-PCNA-Ub/PCNA-SHORT.jpg]

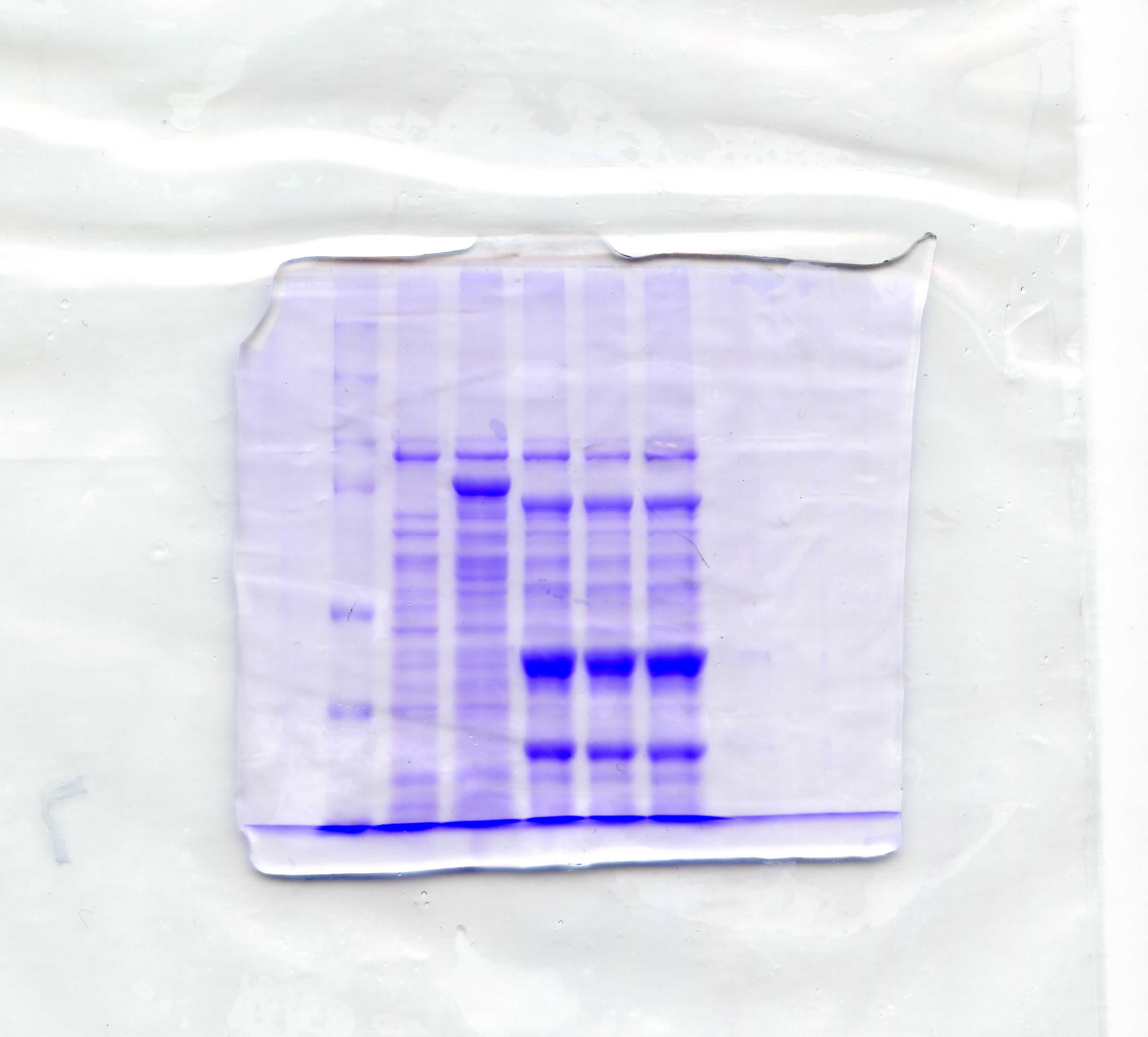

Supplement: Supplementary file 4 — Source Data Fig. 4 [file 44318_2024_66_MOESM4_ESM.zip › Figure 3/G-220406-PIP-Pd/Coomassie staining.jpg]

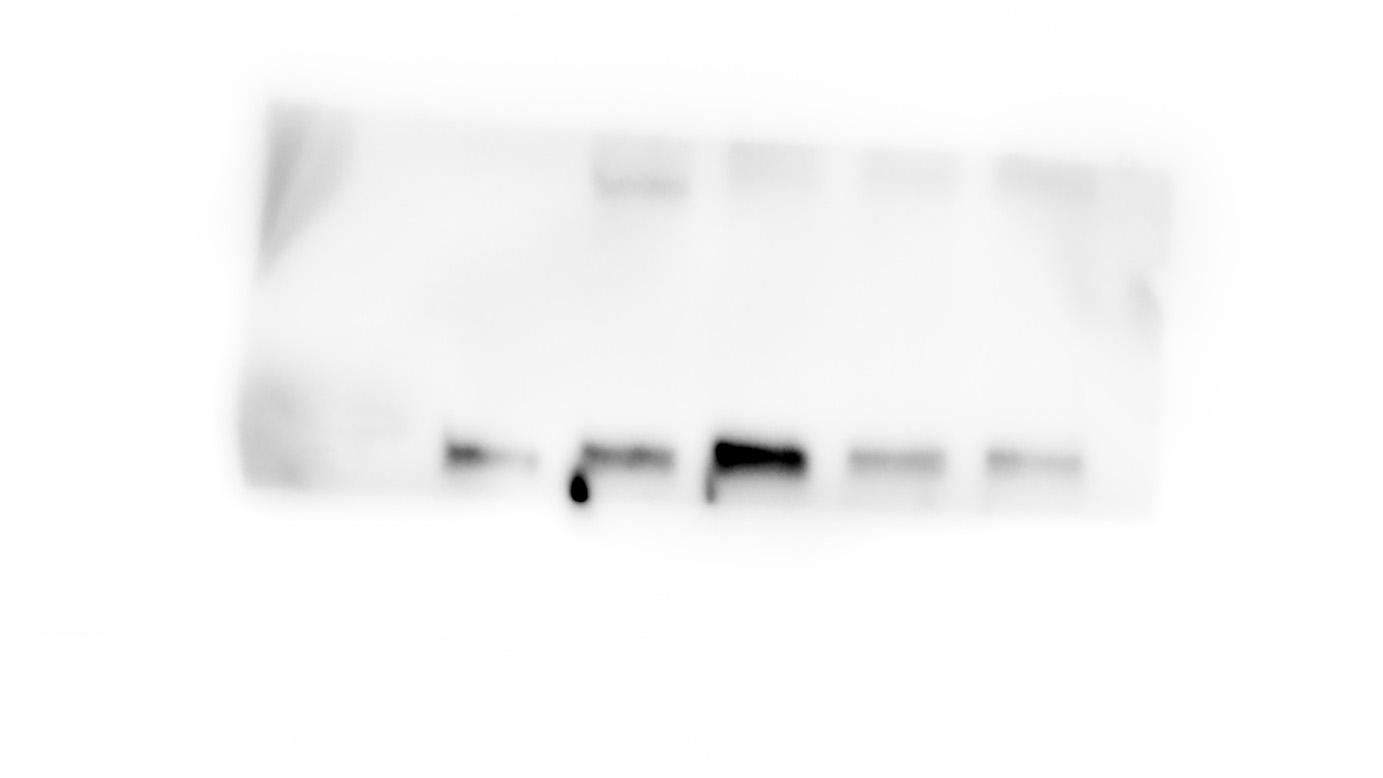

Supplement: Supplementary file 4 — Source Data Fig. 4 [file 44318_2024_66_MOESM4_ESM.zip › Figure 3/G-220406-PIP-Pd/Rad18-Pulldown.jpg]

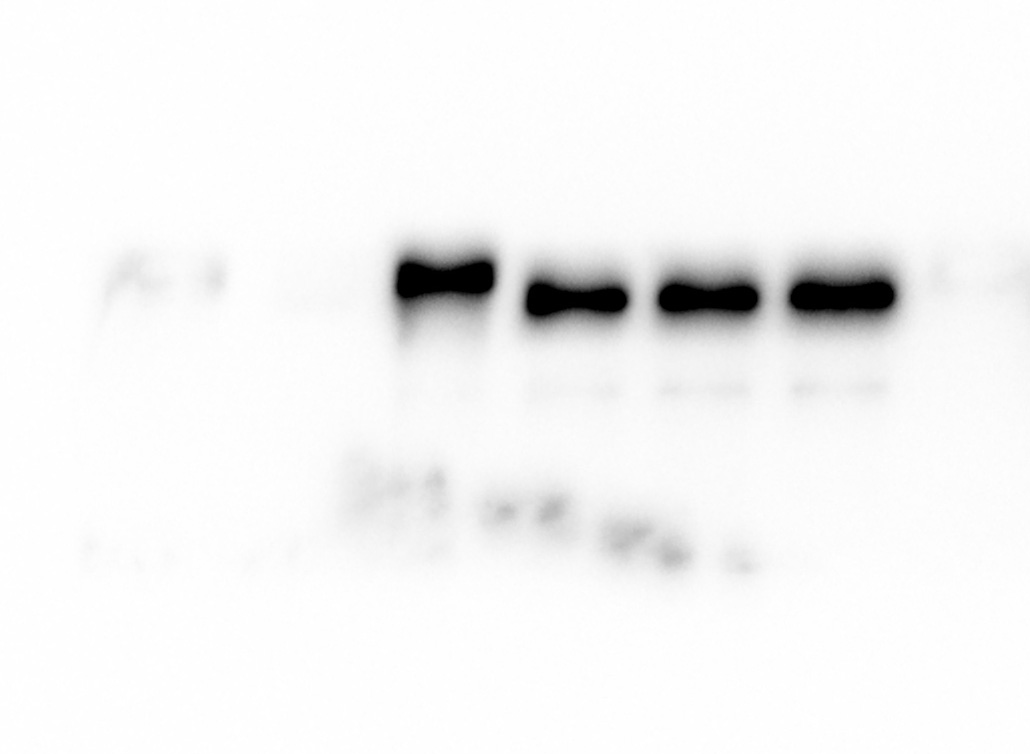

Supplement: Supplementary file 4 — Source Data Fig. 4 [file 44318_2024_66_MOESM4_ESM.zip › Figure 3/G-220406-PIP-Pd/MBP-INPUT.jpg]

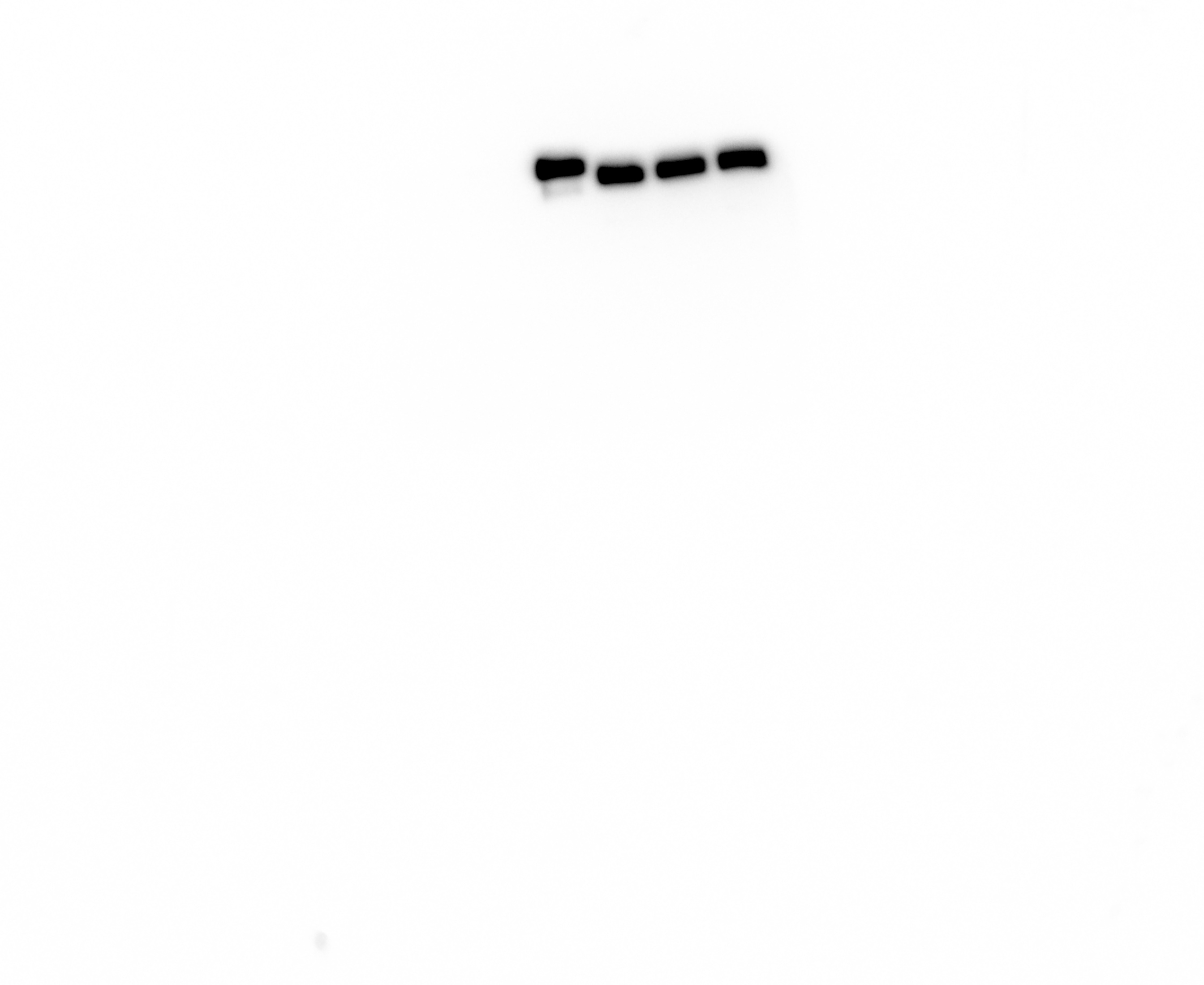

Supplement: Supplementary file 4 — Source Data Fig. 4 [file 44318_2024_66_MOESM4_ESM.zip › Figure 3/G-220406-PIP-Pd/MBP-Pulldown.jpg]

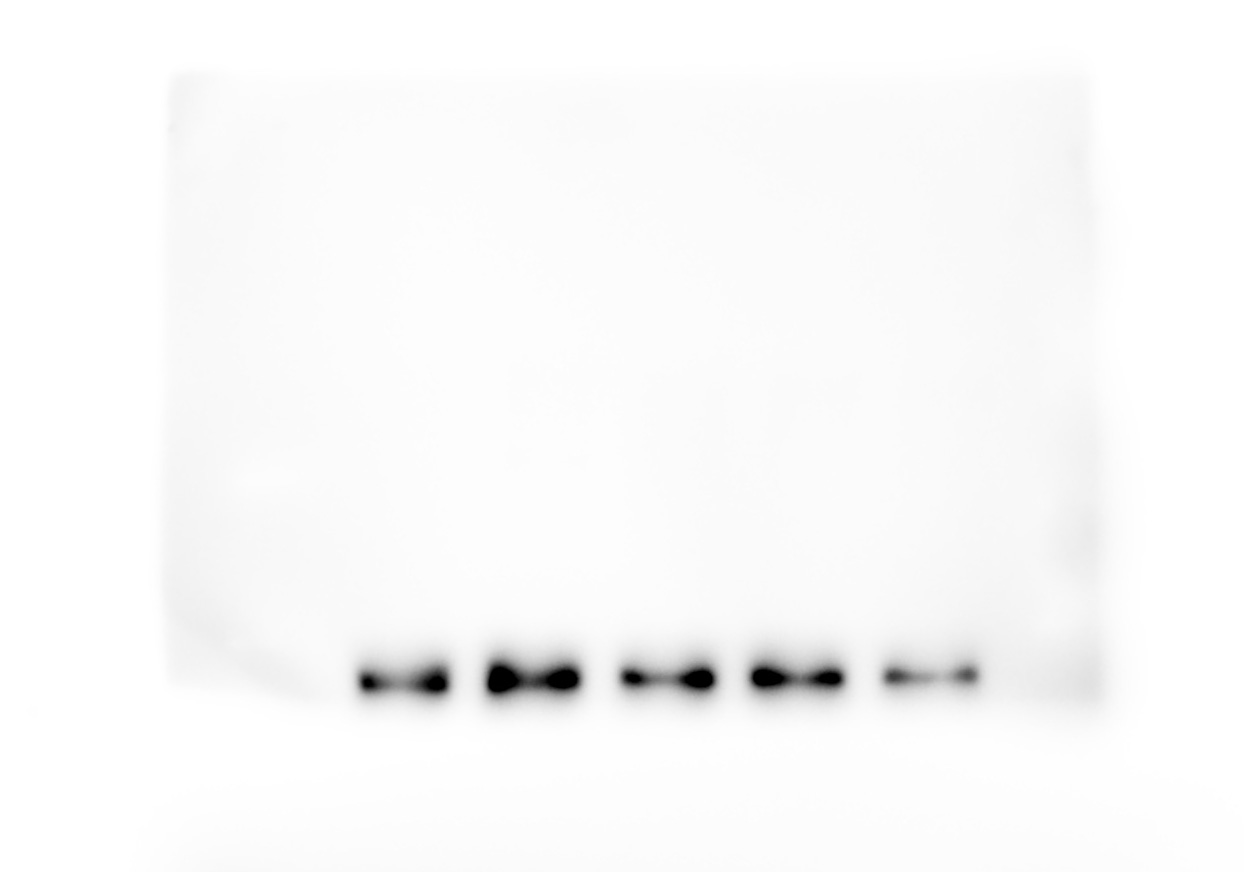

Supplement: Supplementary file 4 — Source Data Fig. 4 [file 44318_2024_66_MOESM4_ESM.zip › Figure 3/G-220406-PIP-Pd/Rad18-INPUT.jpg]

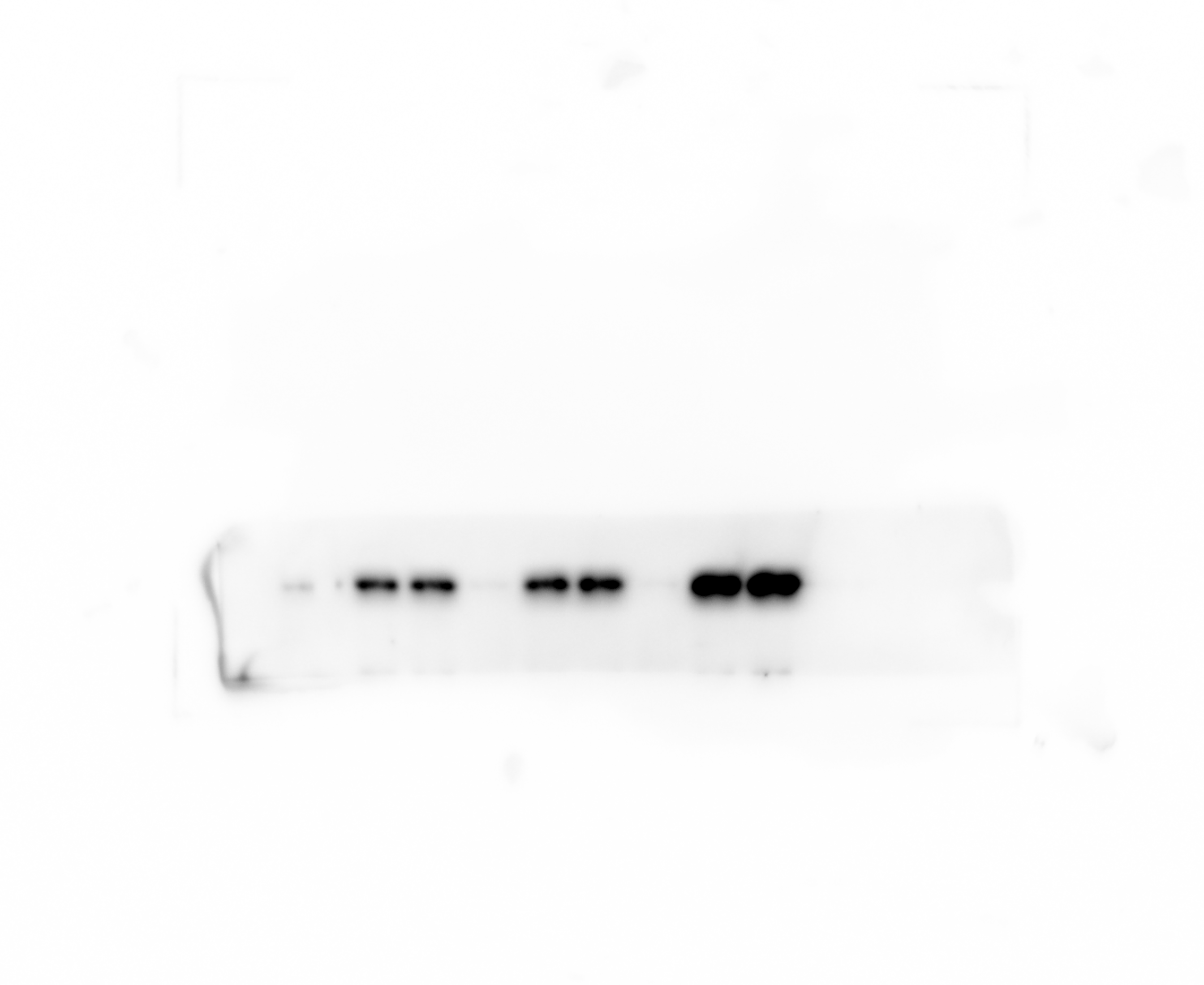

Supplement: Supplementary file 4 — Source Data Fig. 4 [file 44318_2024_66_MOESM4_ESM.zip › Figure 3/L-231114-rH2AX-MEF/H2AX-MEF.tif]

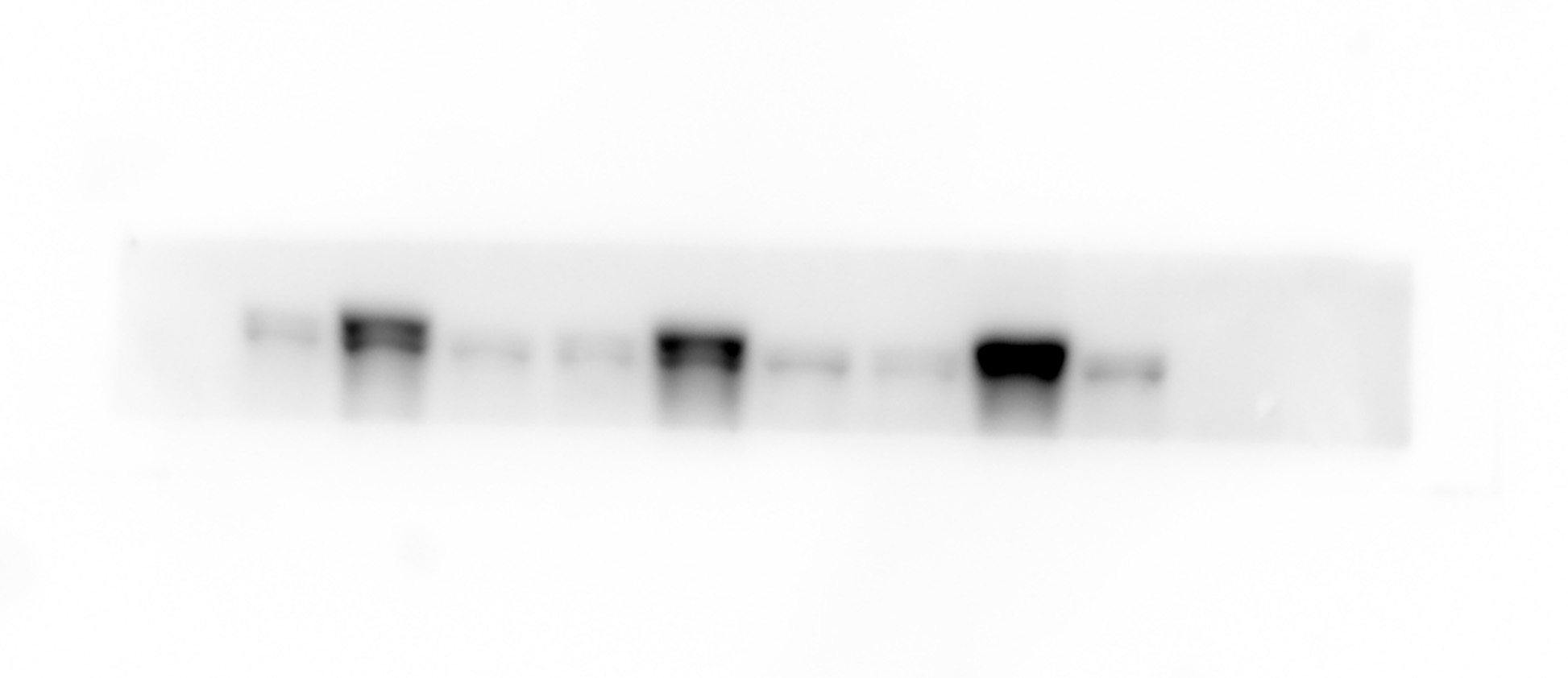

Supplement: Supplementary file 4 — Source Data Fig. 4 [file 44318_2024_66_MOESM4_ESM.zip › Figure 3/L-231114-rH2AX-MEF/p-Chk1-MEF2.jpg]

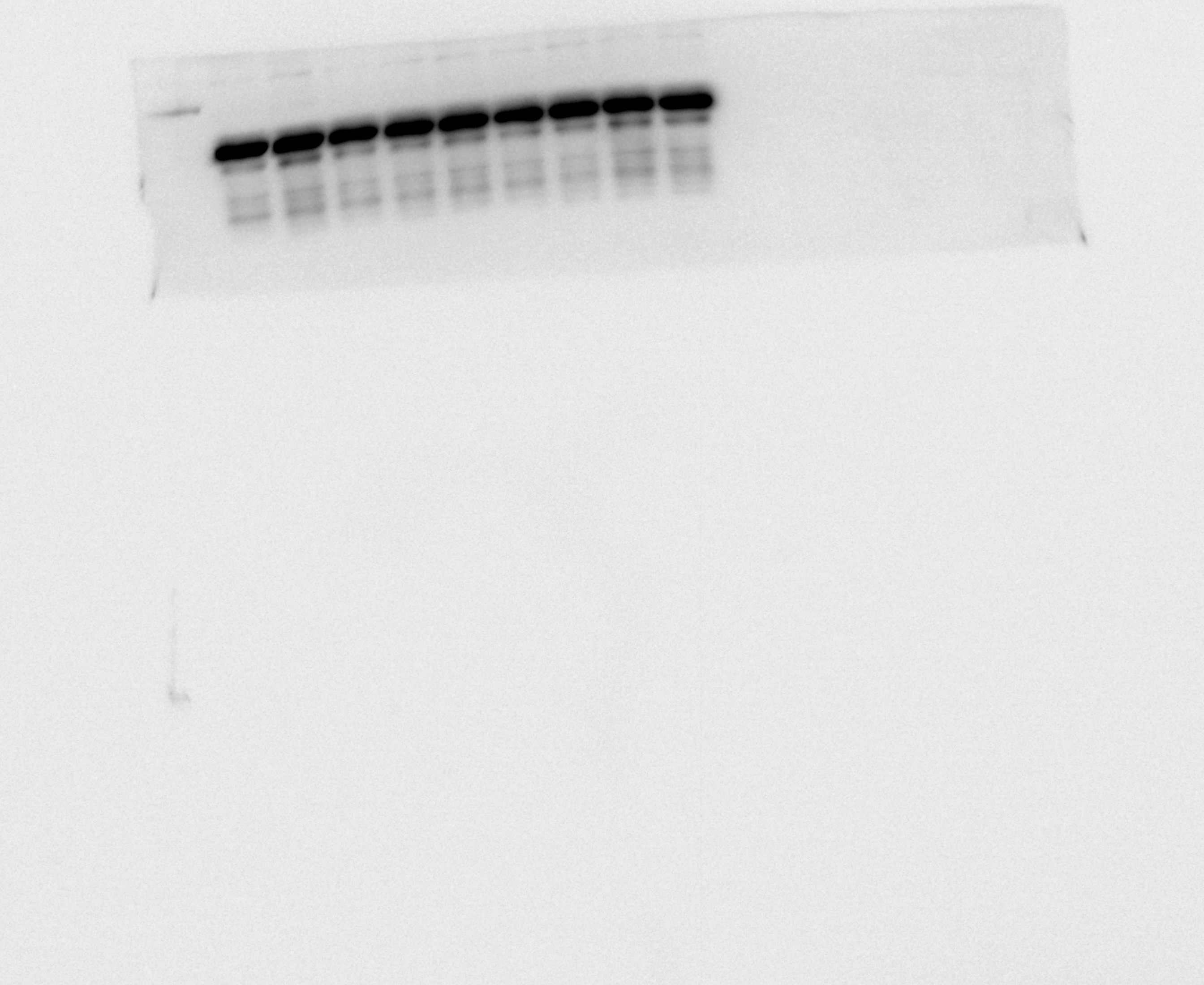

Supplement: Supplementary file 4 — Source Data Fig. 4 [file 44318_2024_66_MOESM4_ESM.zip › Figure 3/L-231114-rH2AX-MEF/PCNA-MEF.jpg]

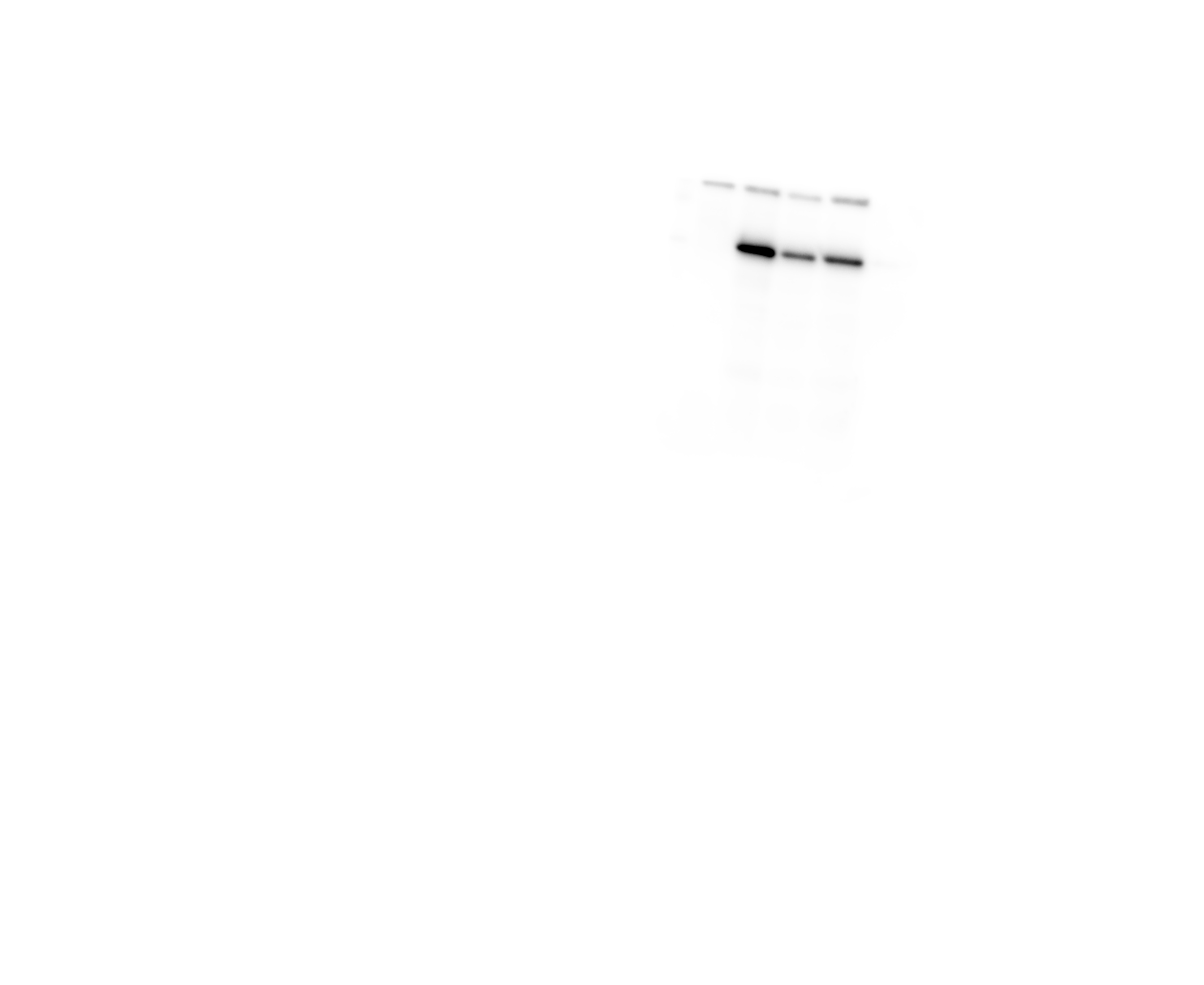

Supplement: Supplementary file 4 — Source Data Fig. 4 [file 44318_2024_66_MOESM4_ESM.zip › Figure 3/E-Rad18-Pulldown-in vitro/GST-Pulldown.jpg]

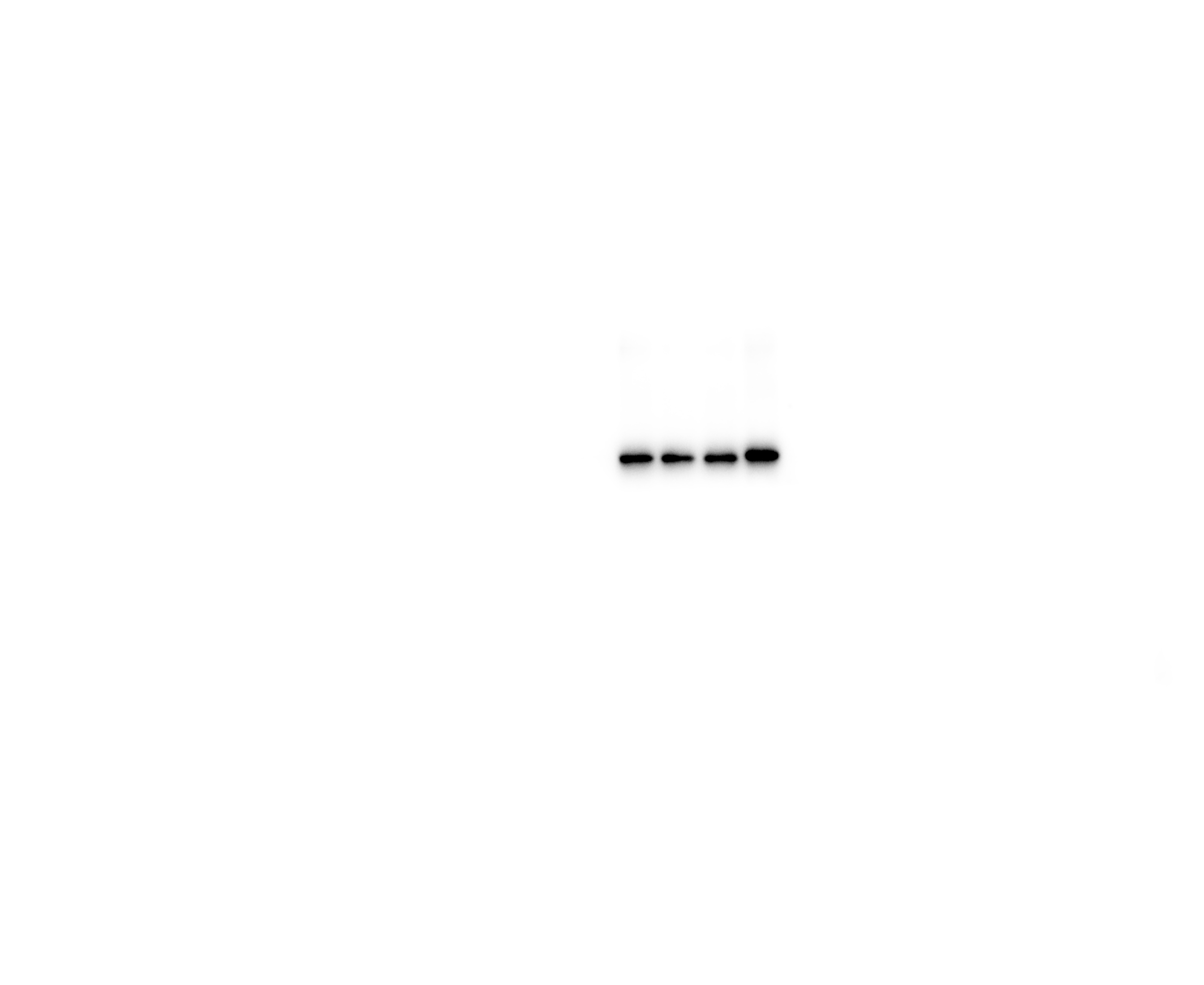

Supplement: Supplementary file 4 — Source Data Fig. 4 [file 44318_2024_66_MOESM4_ESM.zip › Figure 3/E-Rad18-Pulldown-in vitro/RAD18-Pulldown.jpg]

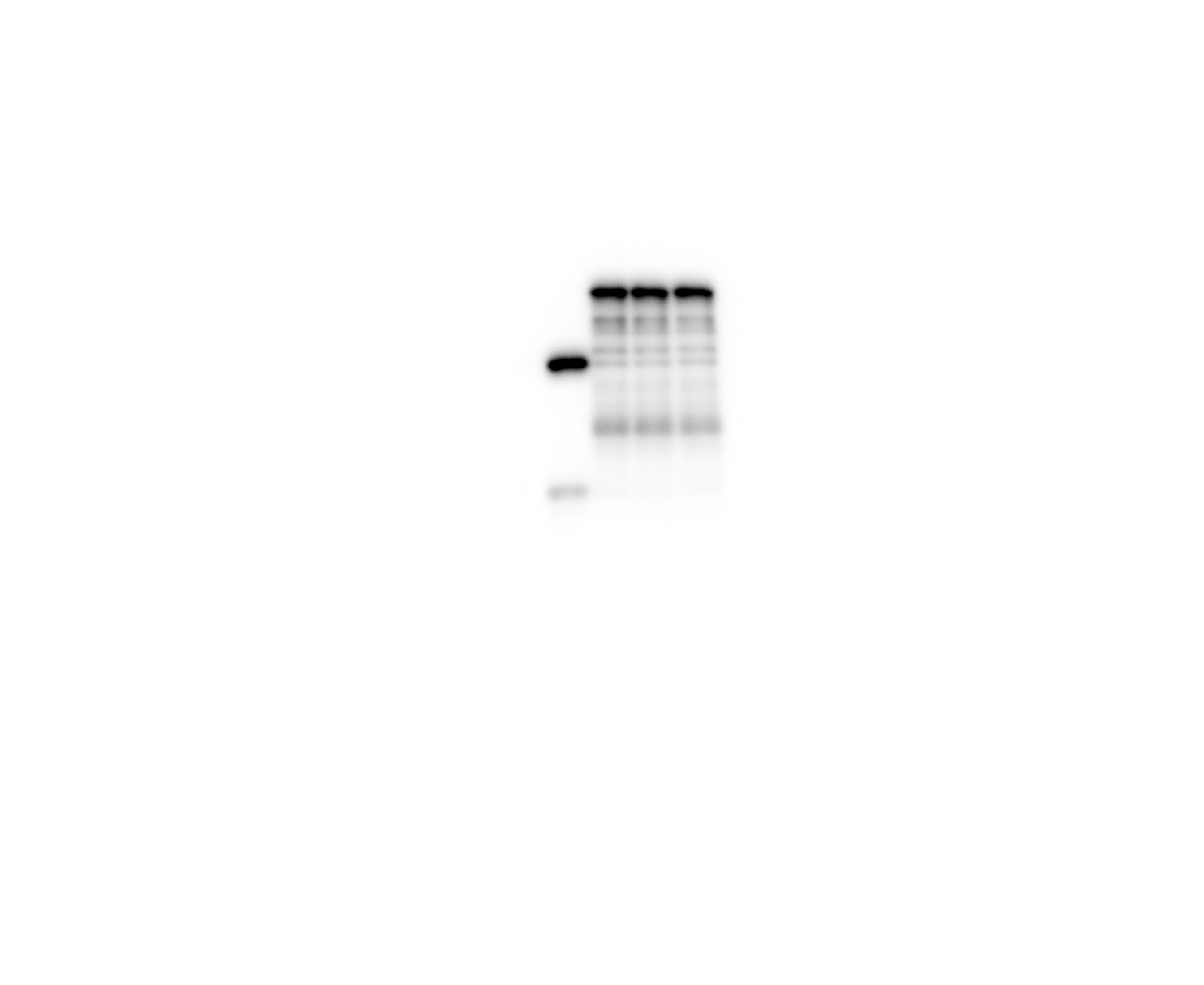

Supplement: Supplementary file 4 — Source Data Fig. 4 [file 44318_2024_66_MOESM4_ESM.zip › Figure 3/E-Rad18-Pulldown-in vitro/GST-INPUT.jpg]

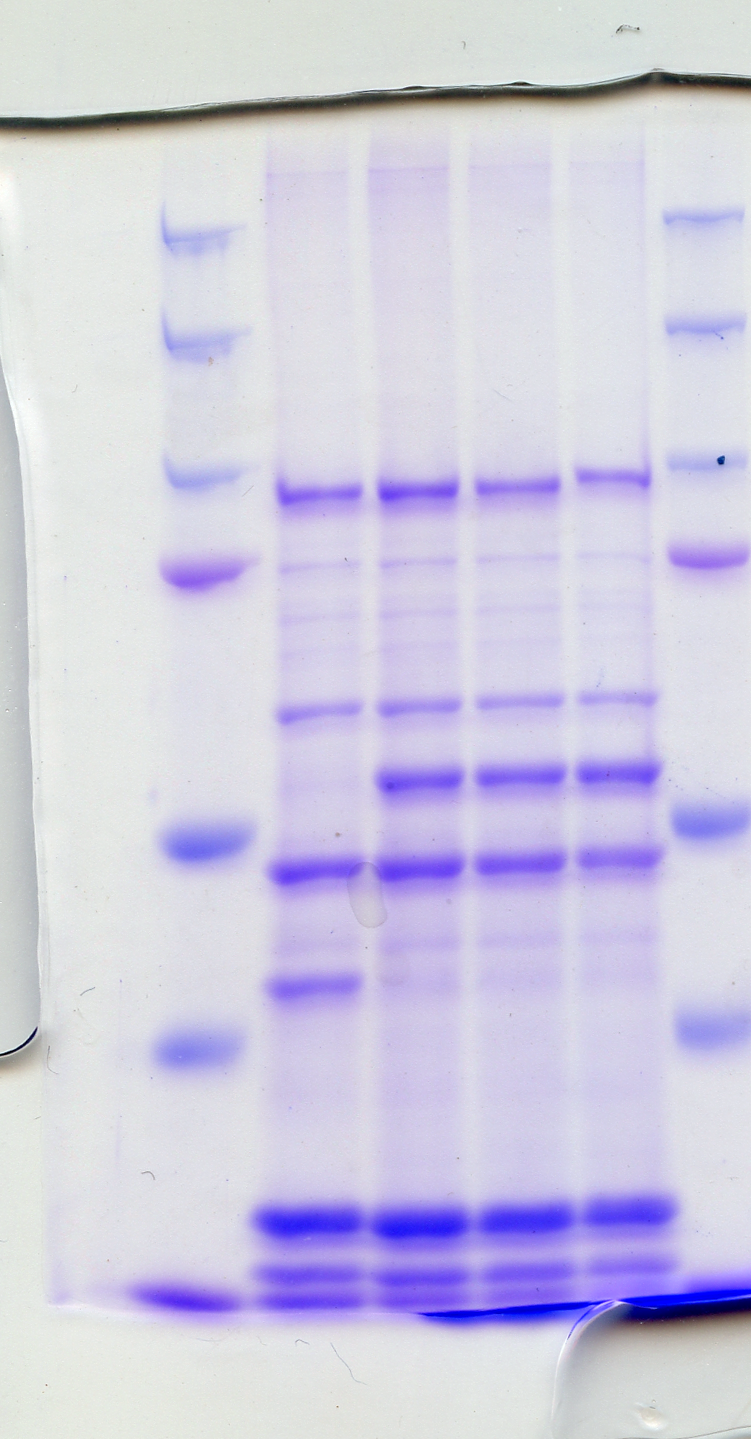

Supplement: Supplementary file 4 — Source Data Fig. 4 [file 44318_2024_66_MOESM4_ESM.zip › Figure 3/E-Rad18-Pulldown-in vitro/Coomassie staining.tif]

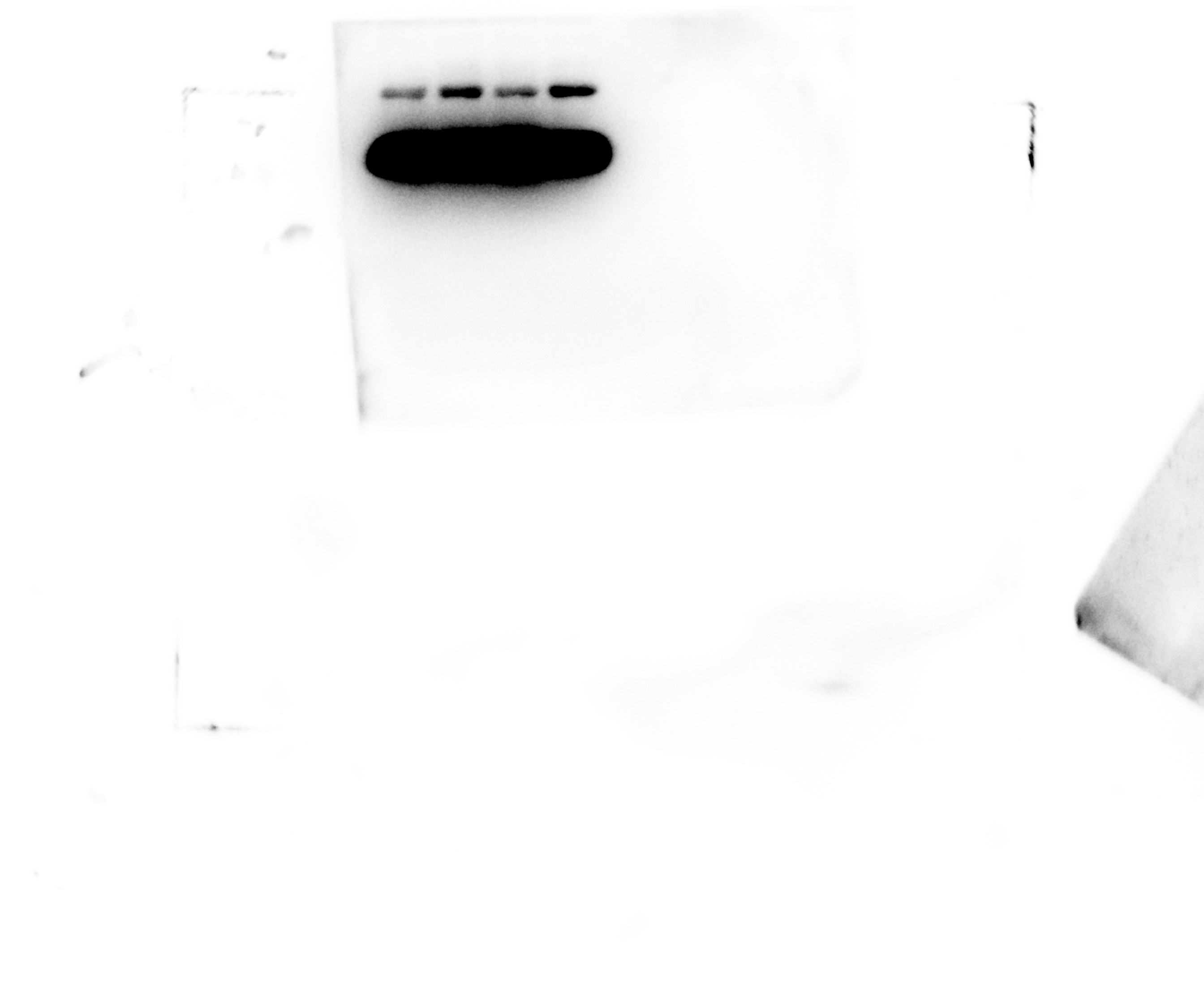

Supplement: Supplementary file 4 — Source Data Fig. 4 [file 44318_2024_66_MOESM4_ESM.zip › Figure 3/I-NIH-3T3-PCNA/MOUSE-PCNA-NIH-LONG.jpg]
