# Supplementary figures and images for: ATR limits Rad18-mediated PCNA monoubiquitination to preserve replication fork and telomerase-independent telomere stability (part 2 of 2)
Source: EMBO J. 2024 Mar 11;43(7):9. doi: 10.1038/s44318-024-00066-9 (PMC10987609; doi:10.1038/s44318-024-00066-9)

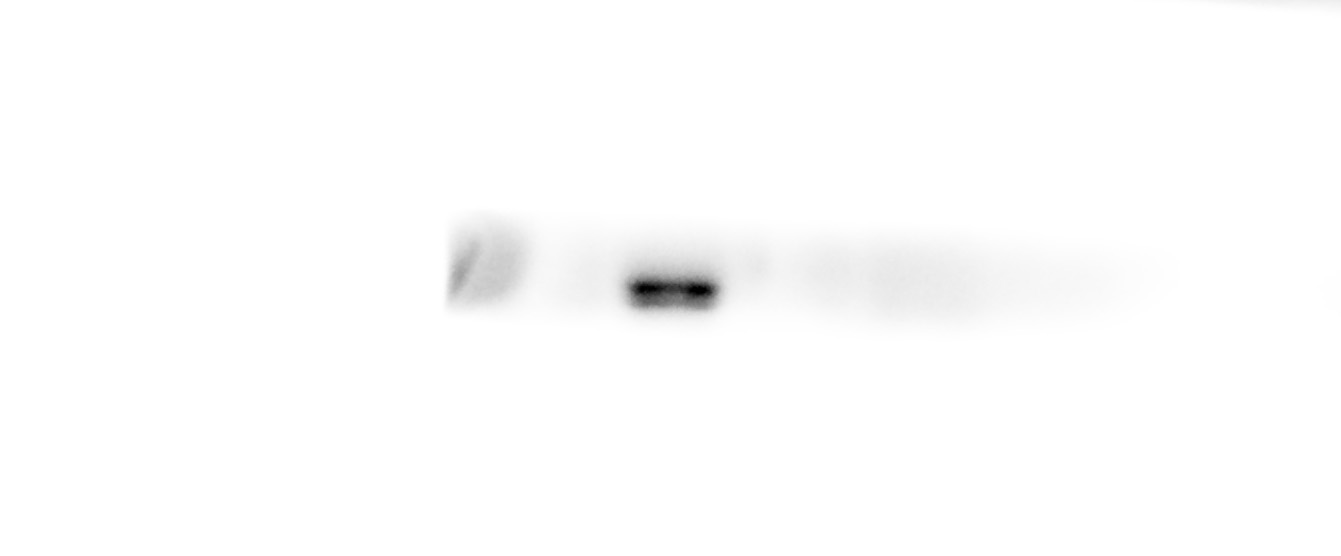

Supplement: Supplementary file 4 — Source Data Fig. 4 [file 44318_2024_66_MOESM4_ESM.zip › Figure 3/I-NIH-3T3-PCNA/pSer345-chk1.jpg]

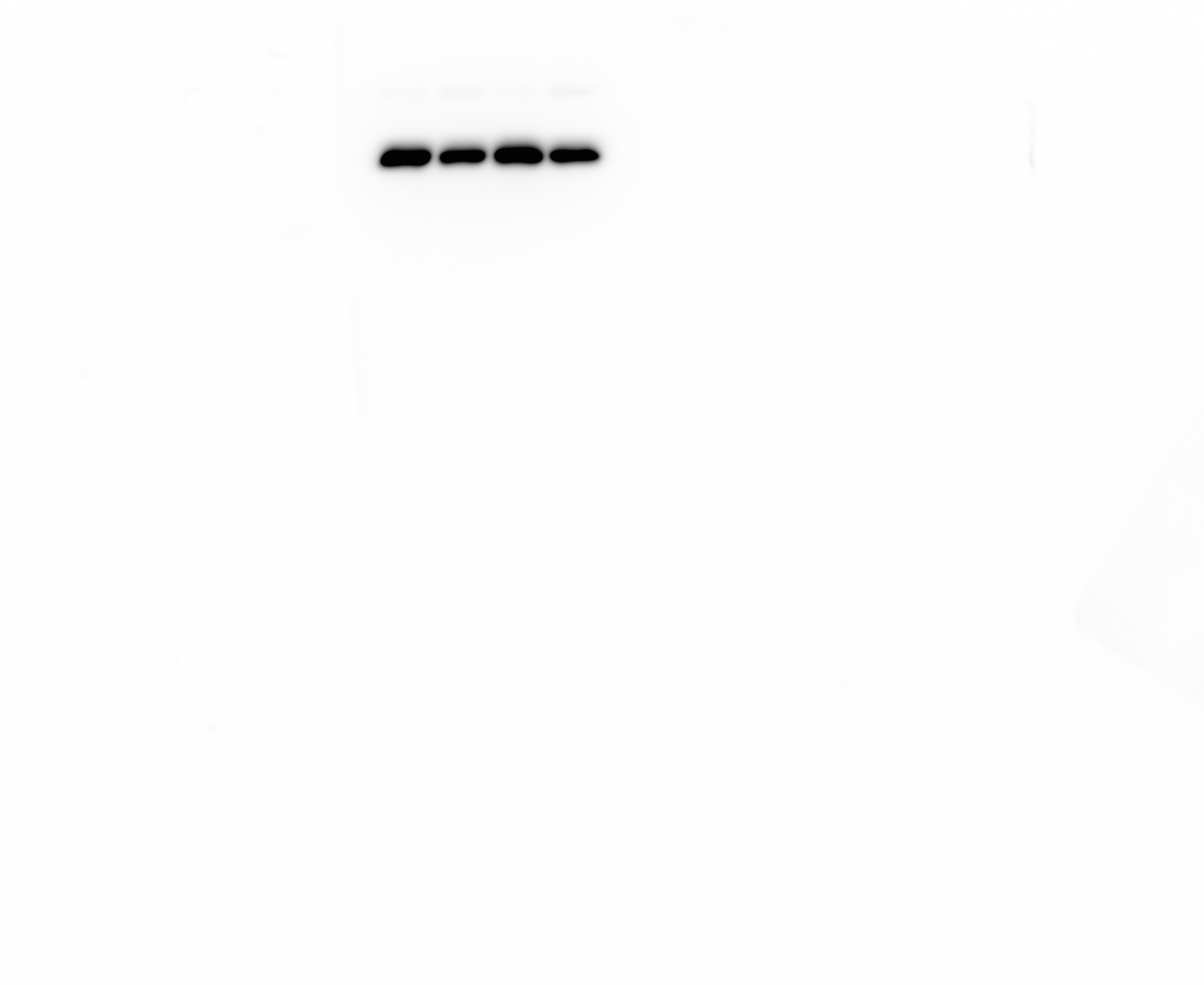

Supplement: Supplementary file 4 — Source Data Fig. 4 [file 44318_2024_66_MOESM4_ESM.zip › Figure 3/I-NIH-3T3-PCNA/MOUSE-PCNA-NIH-short.tif]

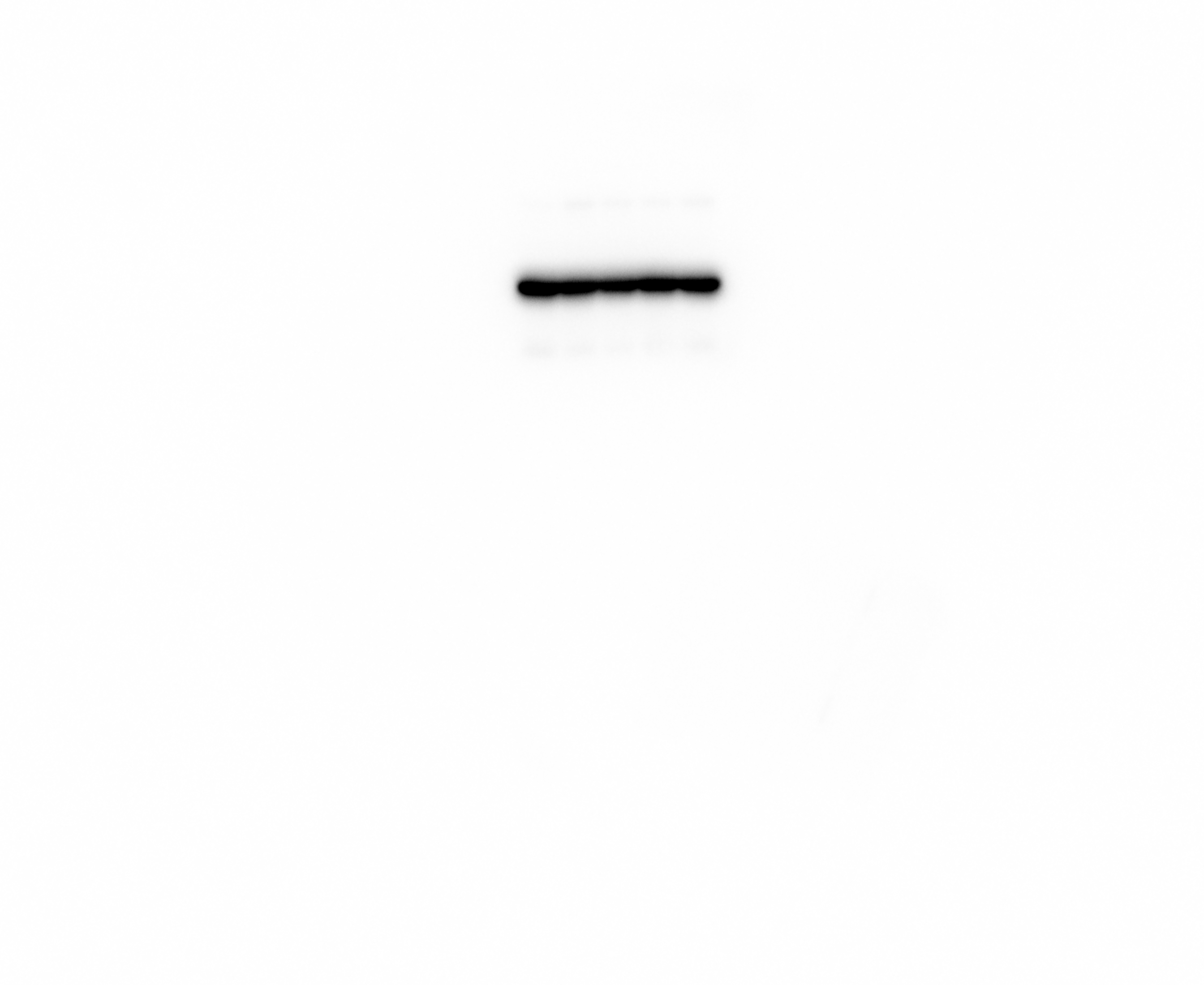

Supplement: Supplementary file 4 — Source Data Fig. 4 [file 44318_2024_66_MOESM4_ESM.zip › Figure 3/F-Cell-SFB-Rad18-PCNA-CoIP/PCNA-Input.tif]

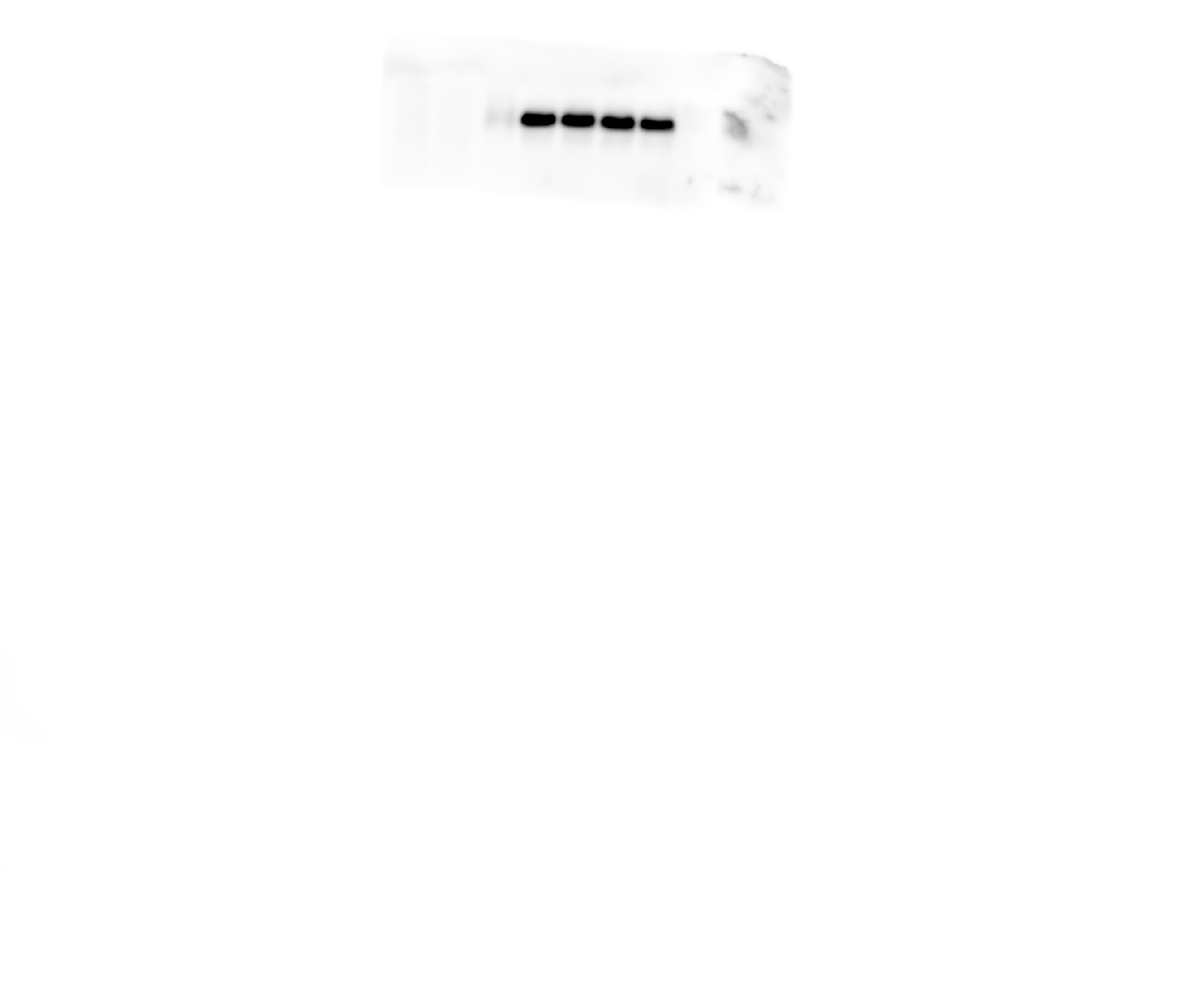

Supplement: Supplementary file 4 — Source Data Fig. 4 [file 44318_2024_66_MOESM4_ESM.zip › Figure 3/F-Cell-SFB-Rad18-PCNA-CoIP/FLAG-Input.jpg]

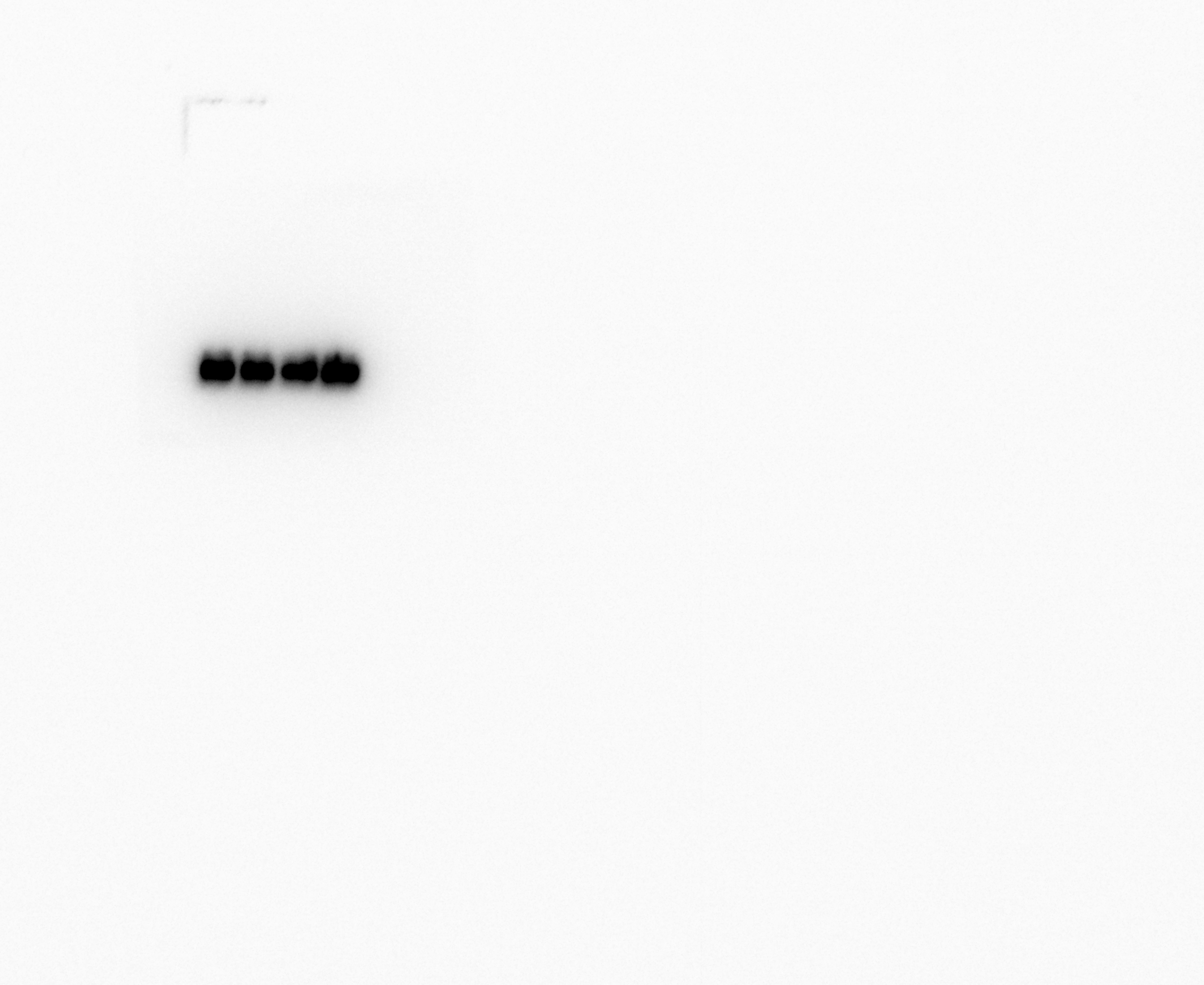

Supplement: Supplementary file 4 — Source Data Fig. 4 [file 44318_2024_66_MOESM4_ESM.zip › Figure 3/F-Cell-SFB-Rad18-PCNA-CoIP/FLAG-IP.tif]

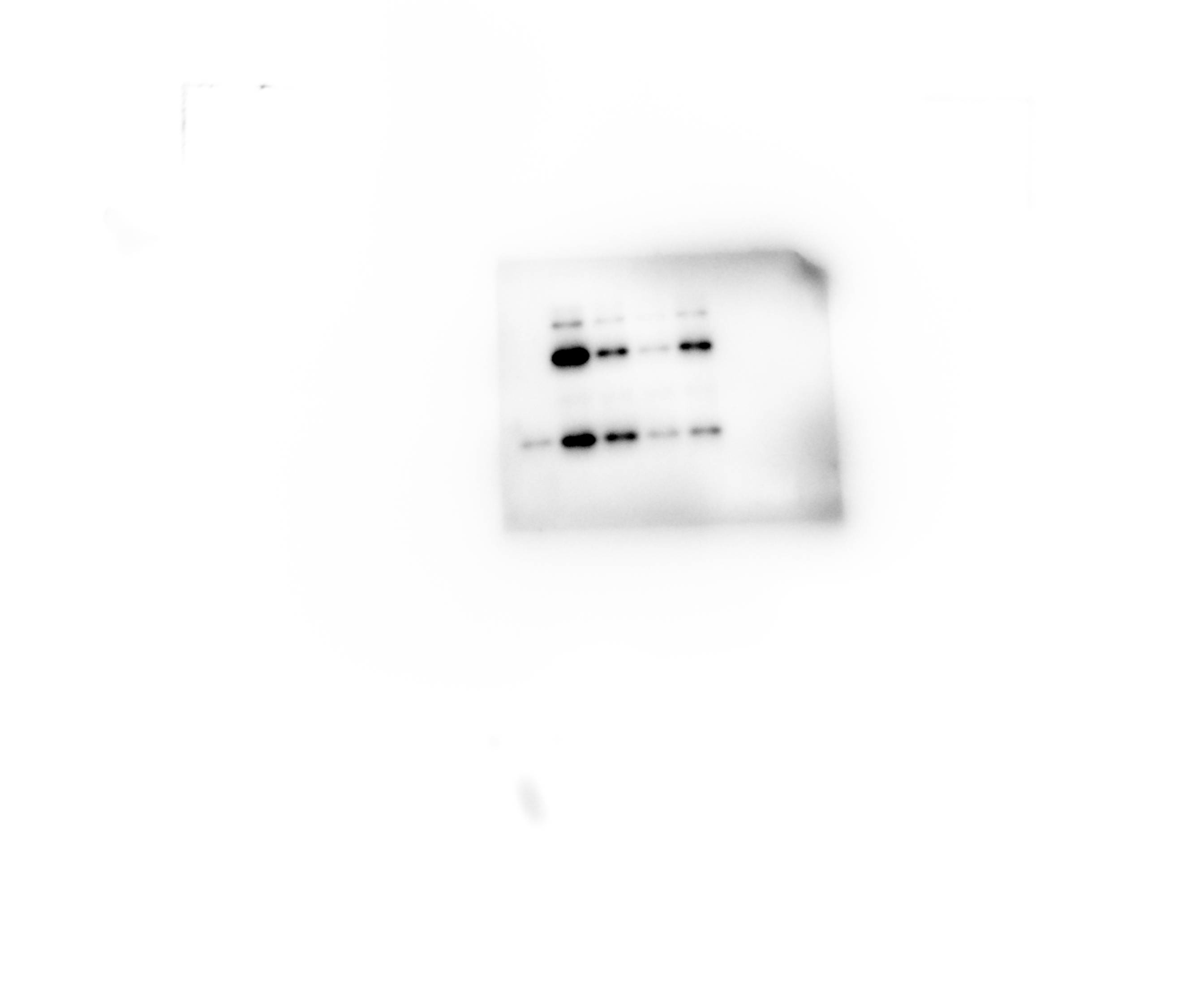

Supplement: Supplementary file 4 — Source Data Fig. 4 [file 44318_2024_66_MOESM4_ESM.zip › Figure 3/F-Cell-SFB-Rad18-PCNA-CoIP/PCNA-COIP.tif]

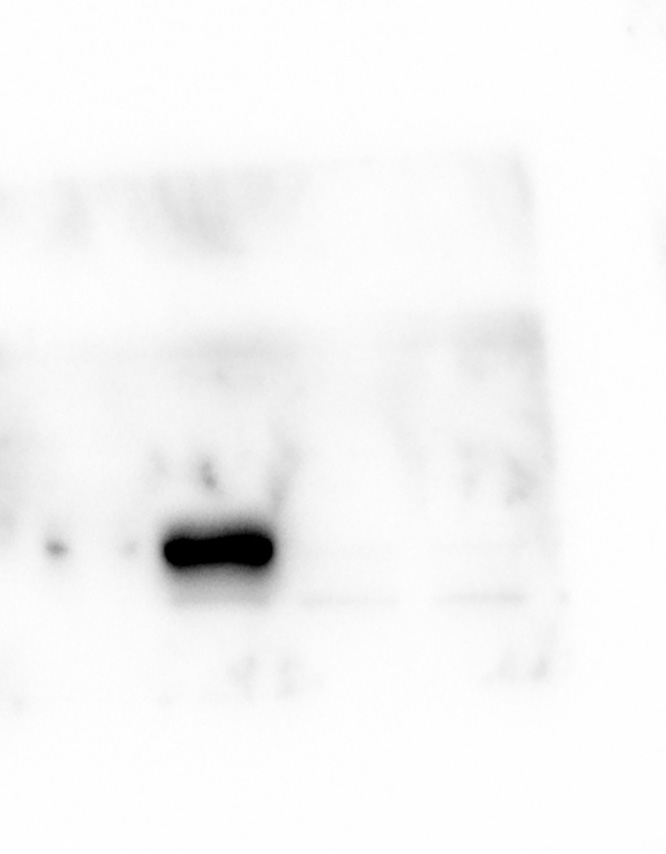

Supplement: Supplementary file 4 — Source Data Fig. 4 [file 44318_2024_66_MOESM4_ESM.zip › Figure 3/C-220731-SFB-Ser403/SER-403.jpg]

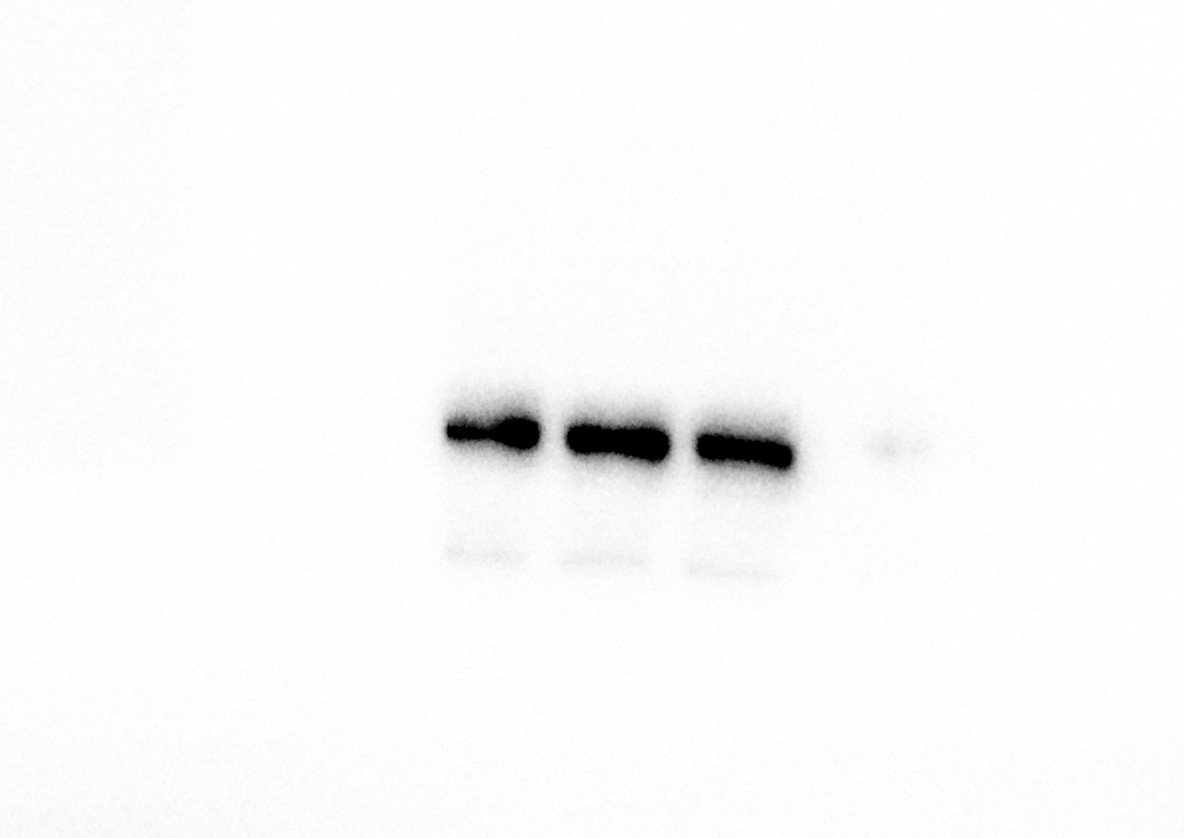

Supplement: Supplementary file 4 — Source Data Fig. 4 [file 44318_2024_66_MOESM4_ESM.zip › Figure 3/C-220731-SFB-Ser403/SER-403-FLAG-IN.jpg]

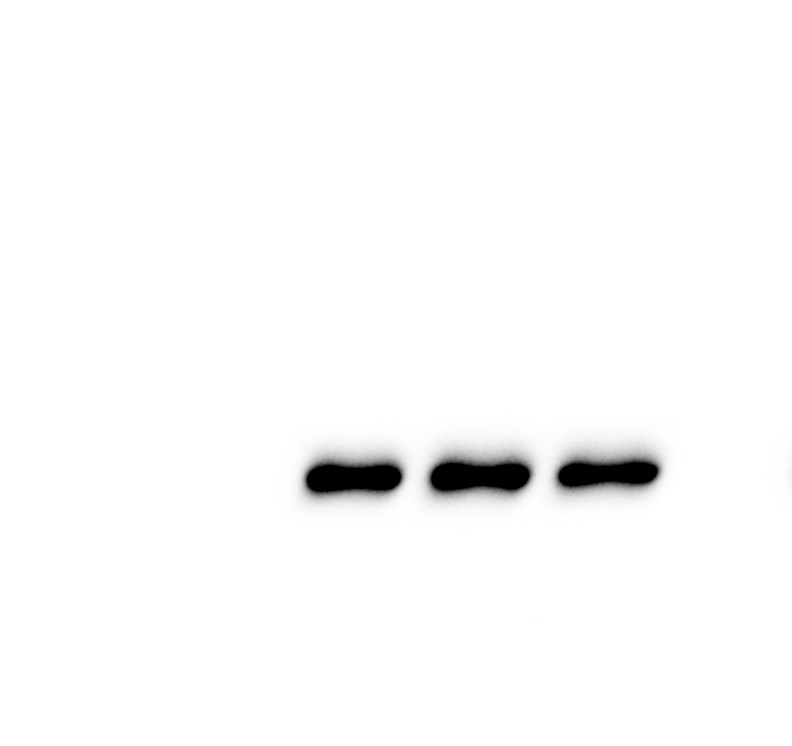

Supplement: Supplementary file 4 — Source Data Fig. 4 [file 44318_2024_66_MOESM4_ESM.zip › Figure 3/C-220731-SFB-Ser403/FLAG-IP.jpg]

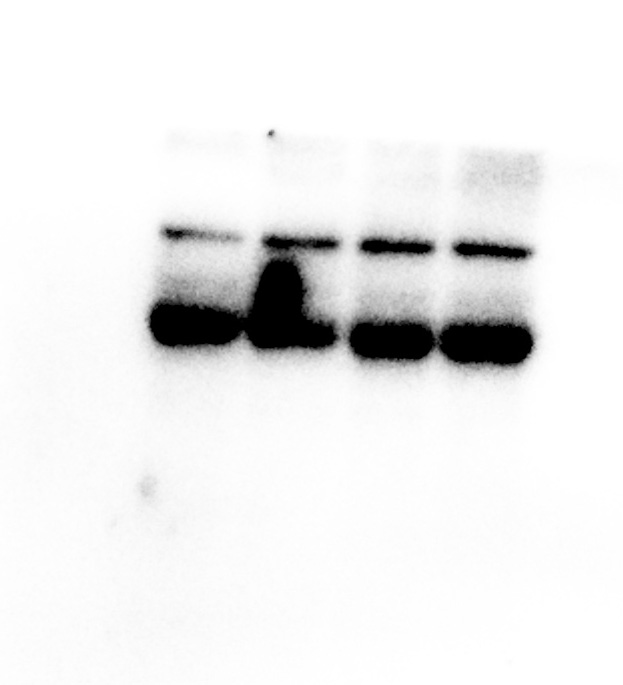

Supplement: Supplementary file 4 — Source Data Fig. 4 [file 44318_2024_66_MOESM4_ESM.zip › Figure 3/D-Cell Lysis-Pd/RPA1-CELL-Pulldown.jpg]

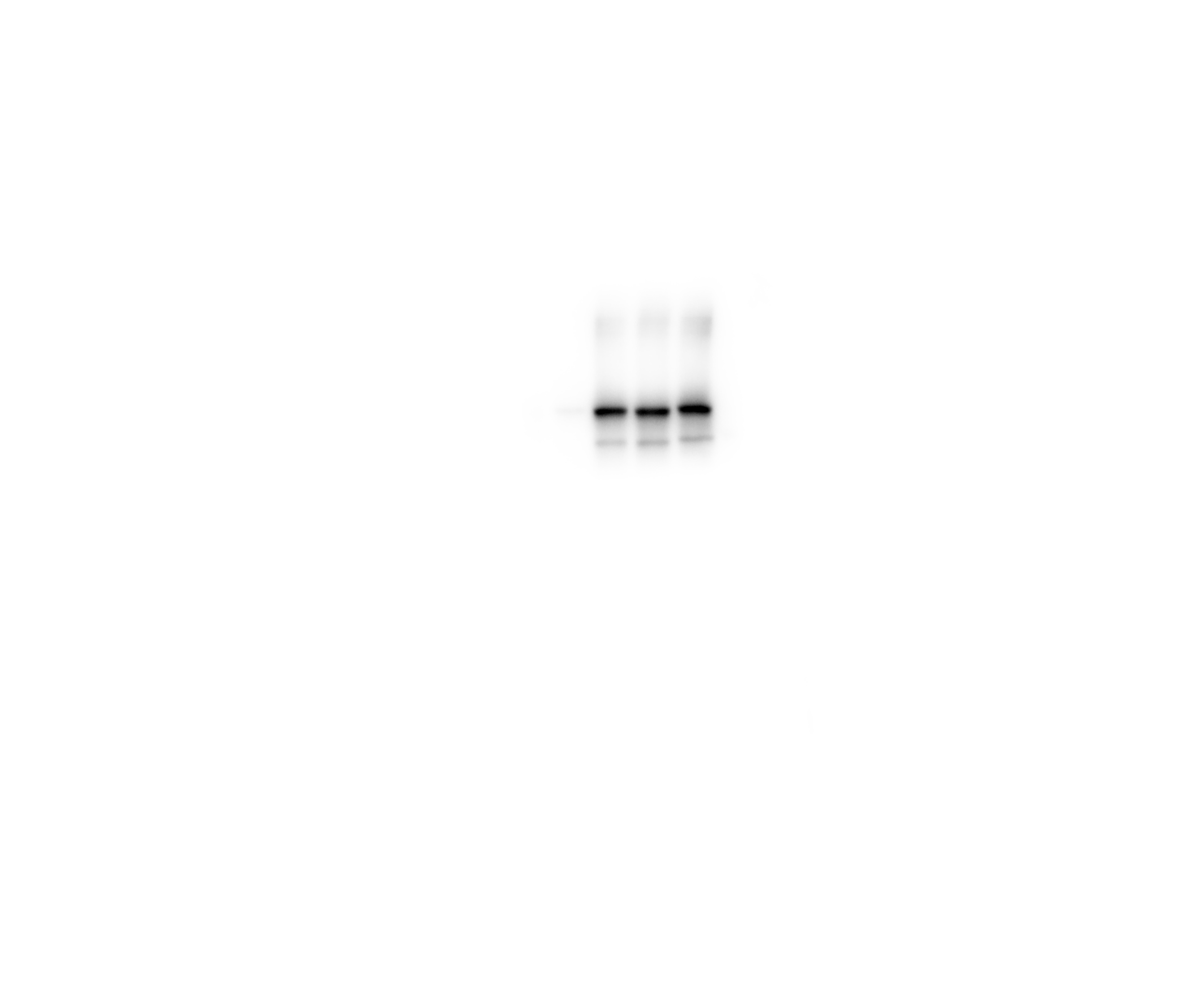

Supplement: Supplementary file 4 — Source Data Fig. 4 [file 44318_2024_66_MOESM4_ESM.zip › Figure 3/D-Cell Lysis-Pd/RAD18-CELL-Pulldown.jpg]

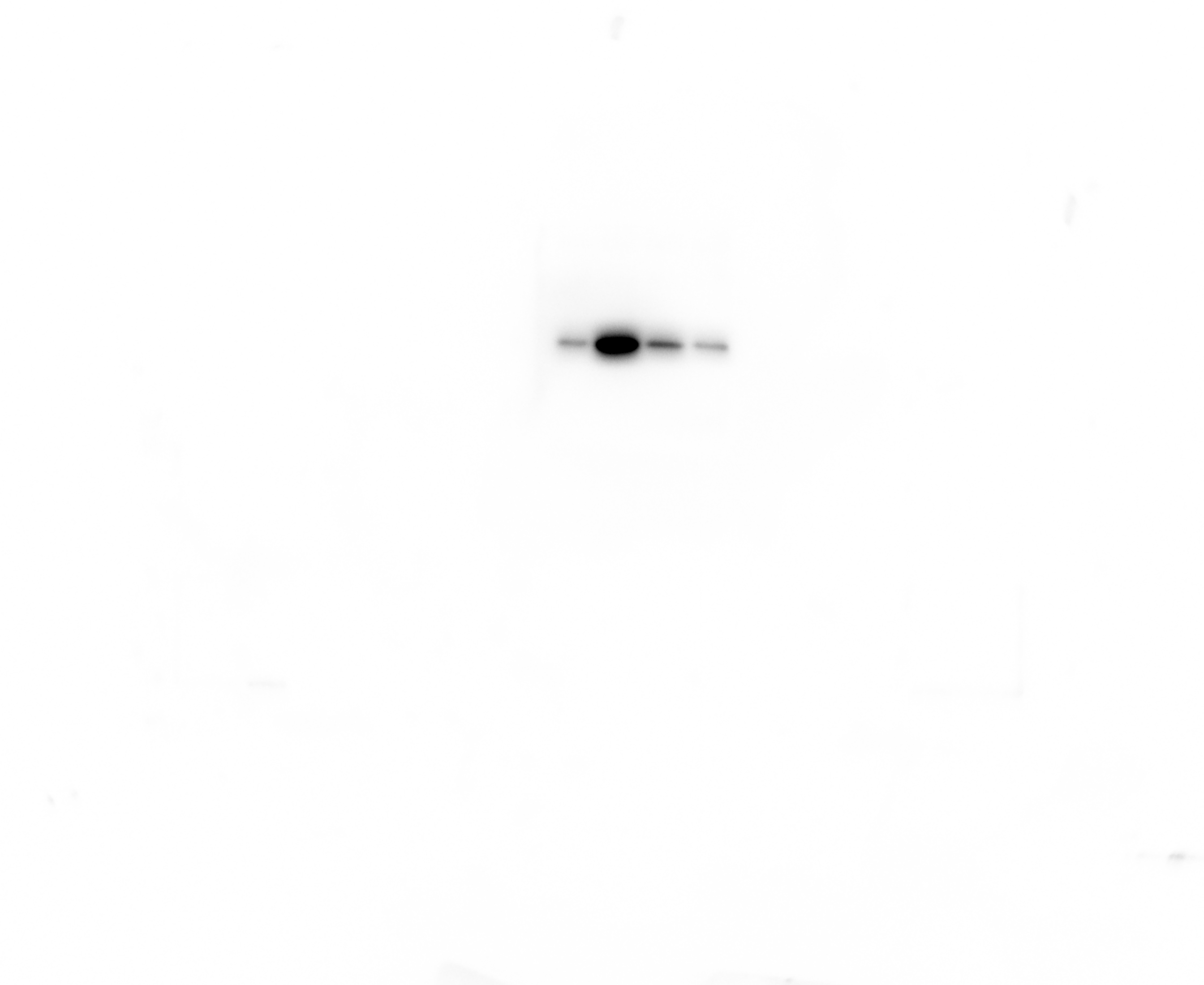

Supplement: Supplementary file 4 — Source Data Fig. 4 [file 44318_2024_66_MOESM4_ESM.zip › Figure 3/D-Cell Lysis-Pd/PCNA-CELL-Pulldown.jpg]

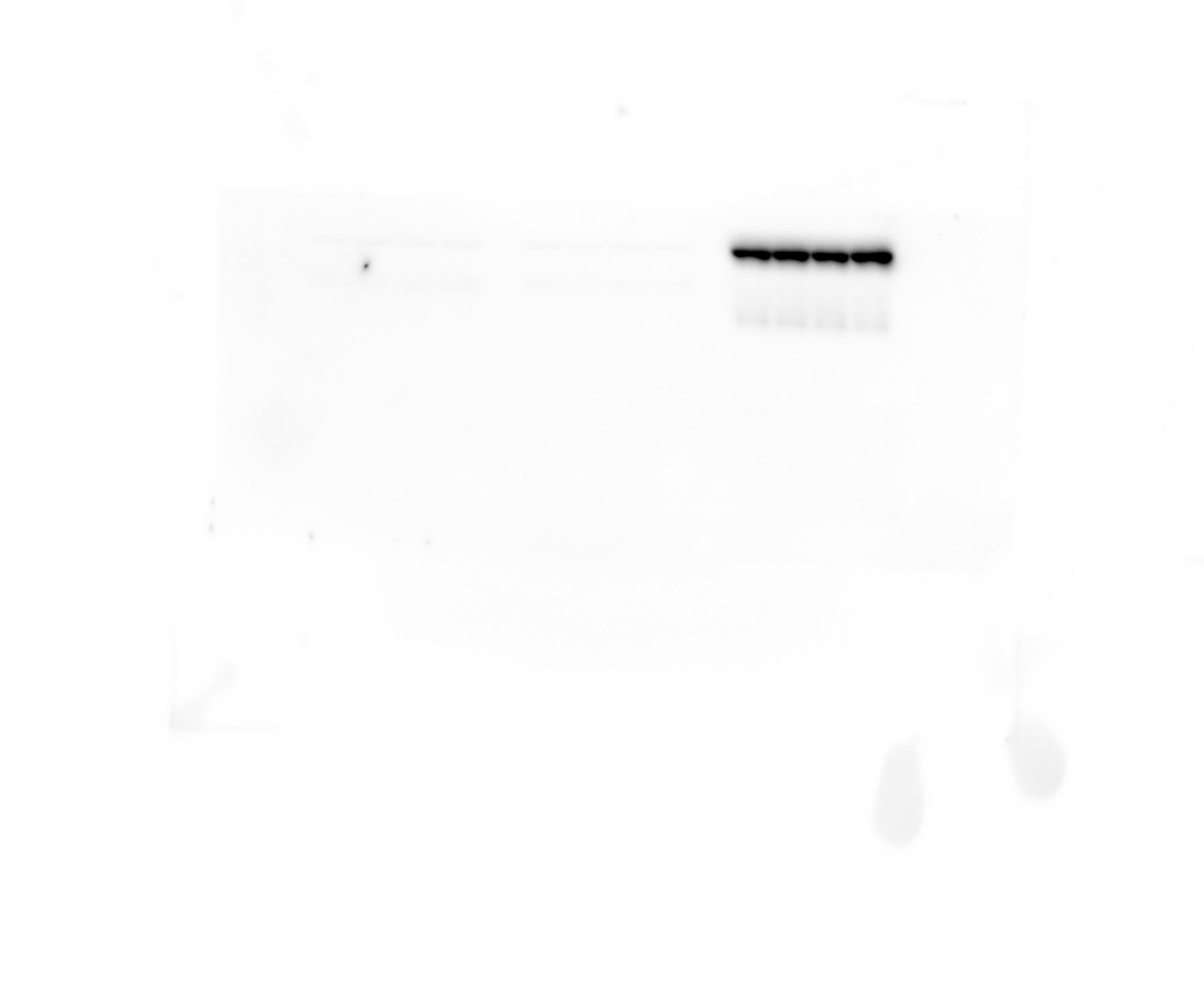

Supplement: Supplementary file 4 — Source Data Fig. 4 [file 44318_2024_66_MOESM4_ESM.zip › Figure 3/D-Cell Lysis-Pd/RPA1-CELL-INPUT.jpg]

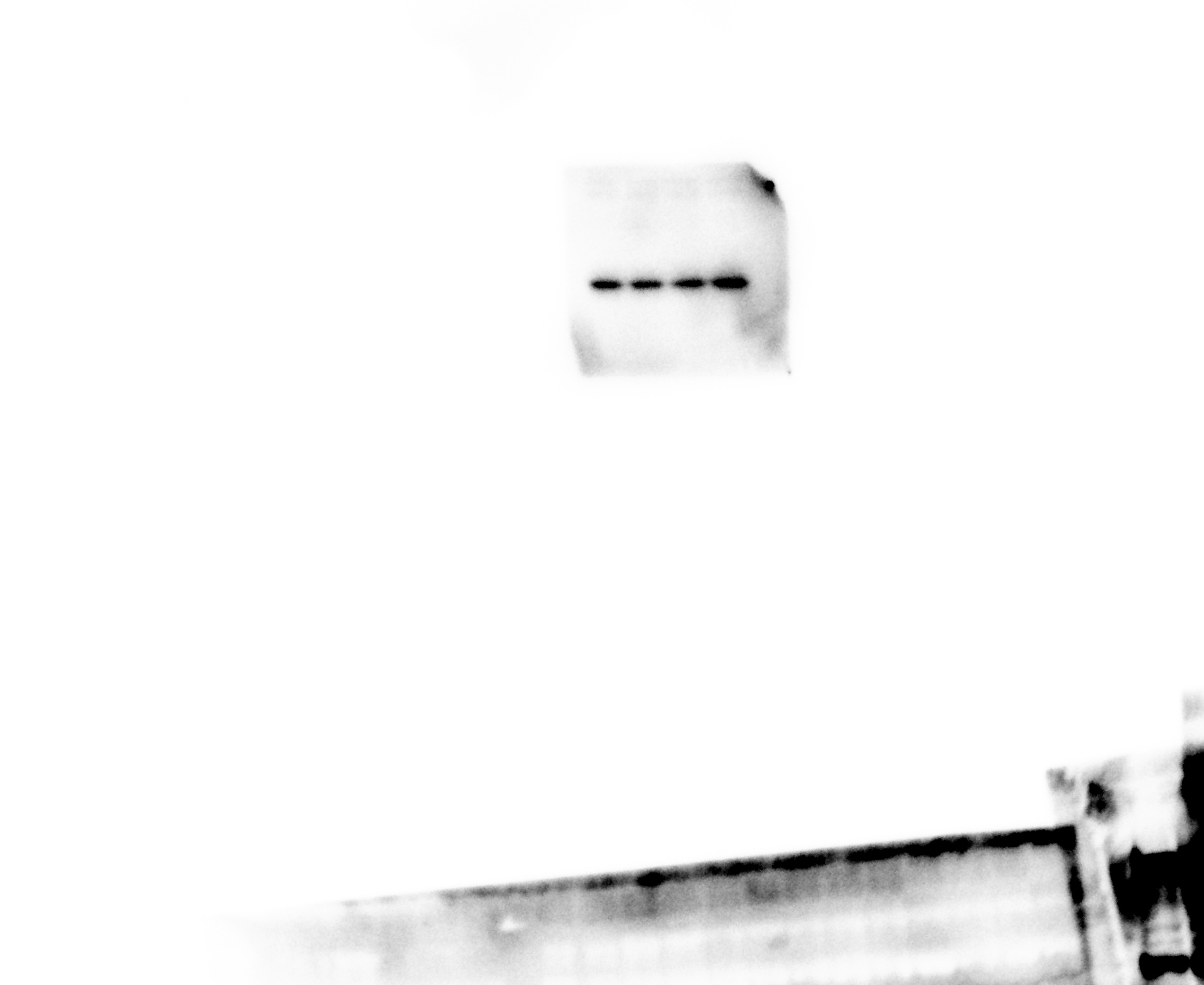

Supplement: Supplementary file 4 — Source Data Fig. 4 [file 44318_2024_66_MOESM4_ESM.zip › Figure 3/D-Cell Lysis-Pd/PCNA-CELL-INPUT.jpg]

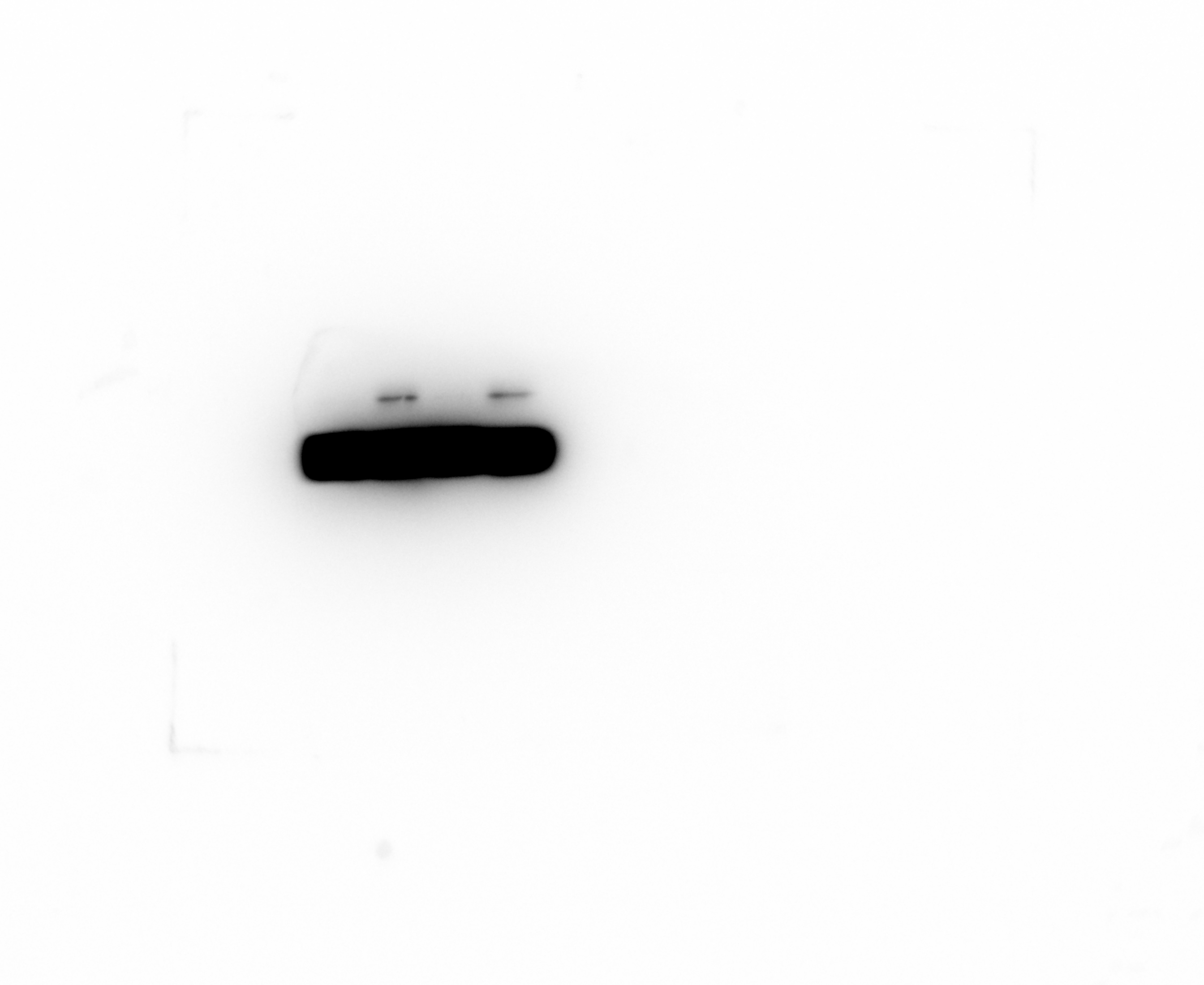

Supplement: Supplementary file 4 — Source Data Fig. 4 [file 44318_2024_66_MOESM4_ESM.zip › Figure 3/J-MEF-PCNA/PCNA-MEF-long.jpg]

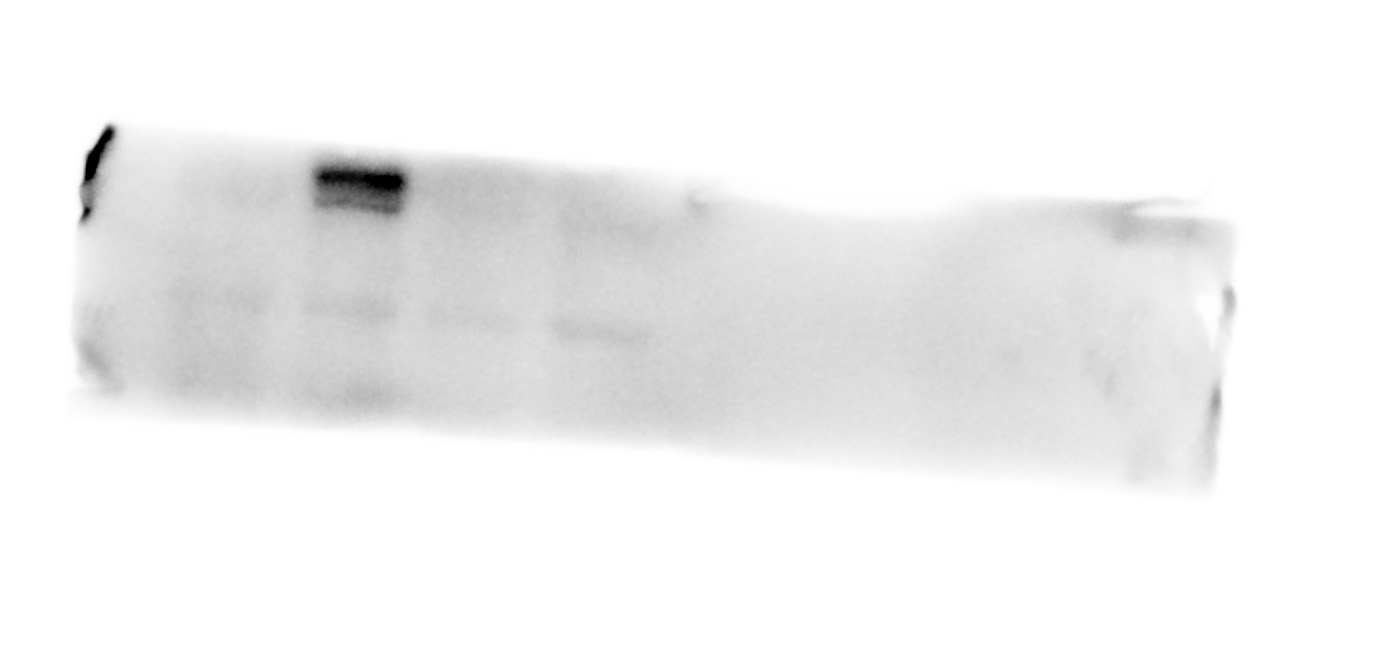

Supplement: Supplementary file 4 — Source Data Fig. 4 [file 44318_2024_66_MOESM4_ESM.zip › Figure 3/J-MEF-PCNA/pSer345-Chk1.jpg]

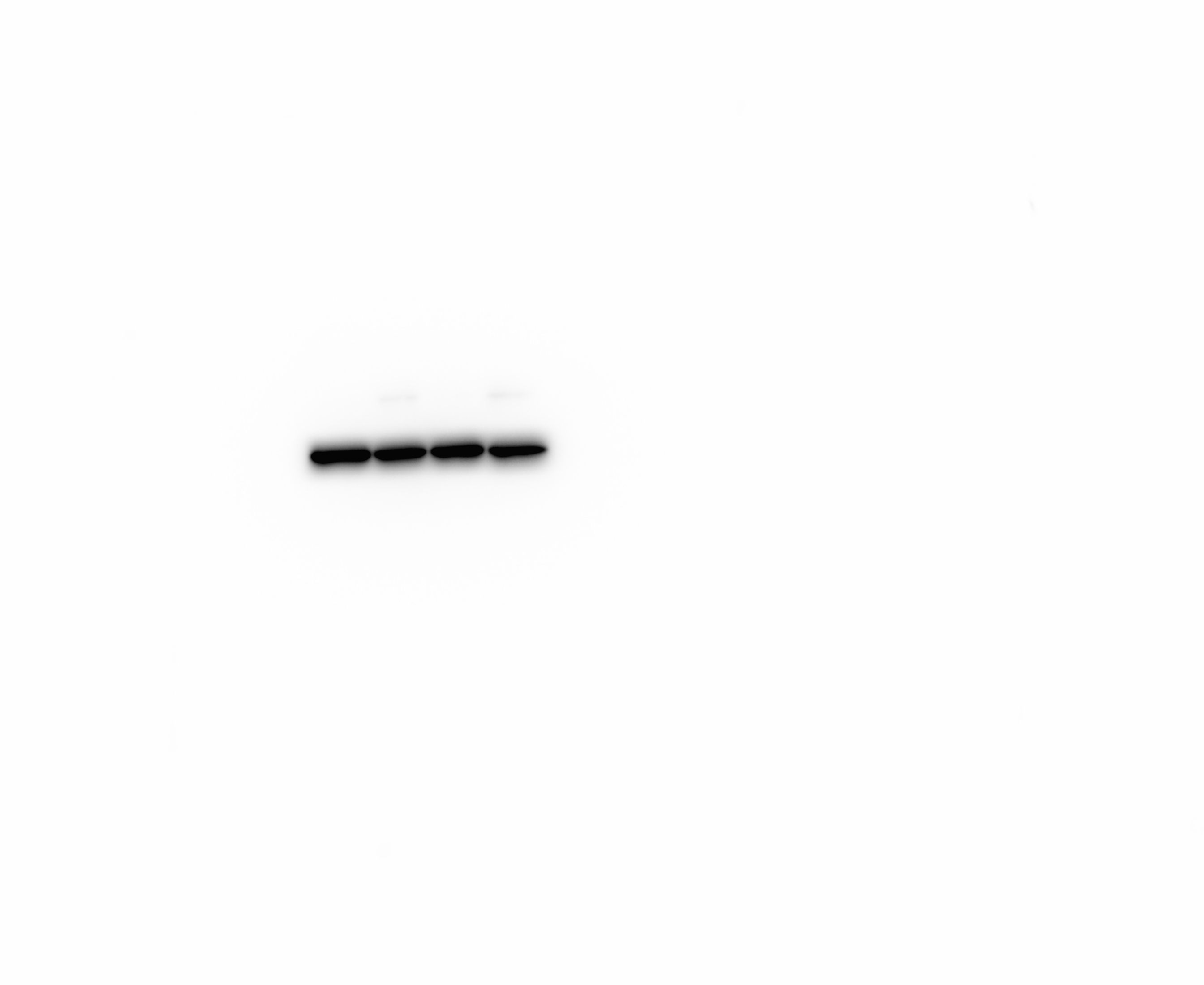

Supplement: Supplementary file 4 — Source Data Fig. 4 [file 44318_2024_66_MOESM4_ESM.zip › Figure 3/J-MEF-PCNA/PCNA-MEF-short.tif]

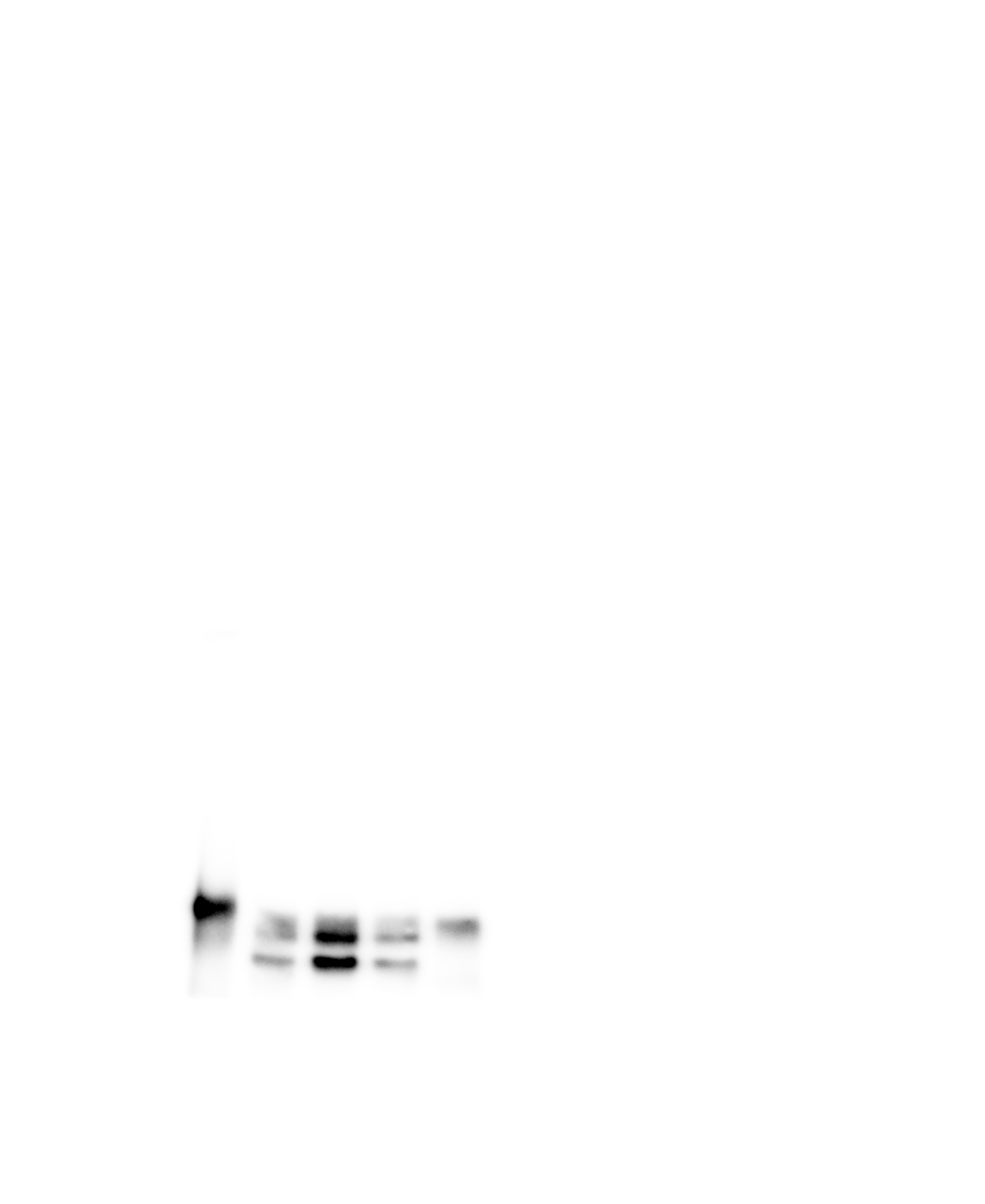

Supplement: Supplementary file 4 — Source Data Fig. 4 [file 44318_2024_66_MOESM4_ESM.zip › Figure 3/B-230401-Rad18-Ser403-endo/pSer403-Rad18.jpg]

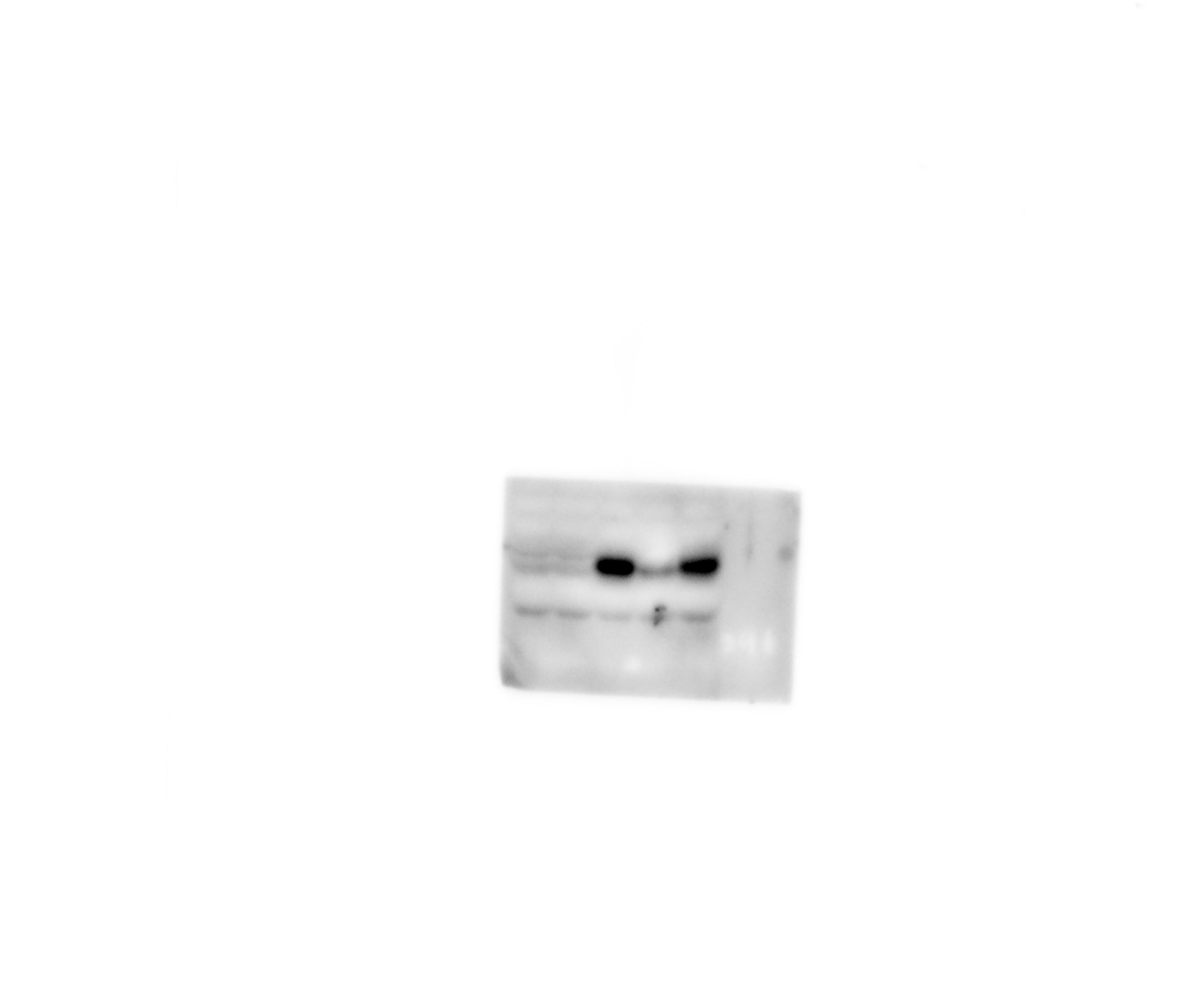

Supplement: Supplementary file 4 — Source Data Fig. 4 [file 44318_2024_66_MOESM4_ESM.zip › Figure 3/B-230401-Rad18-Ser403-endo/pSer345-Chk1.jpg]

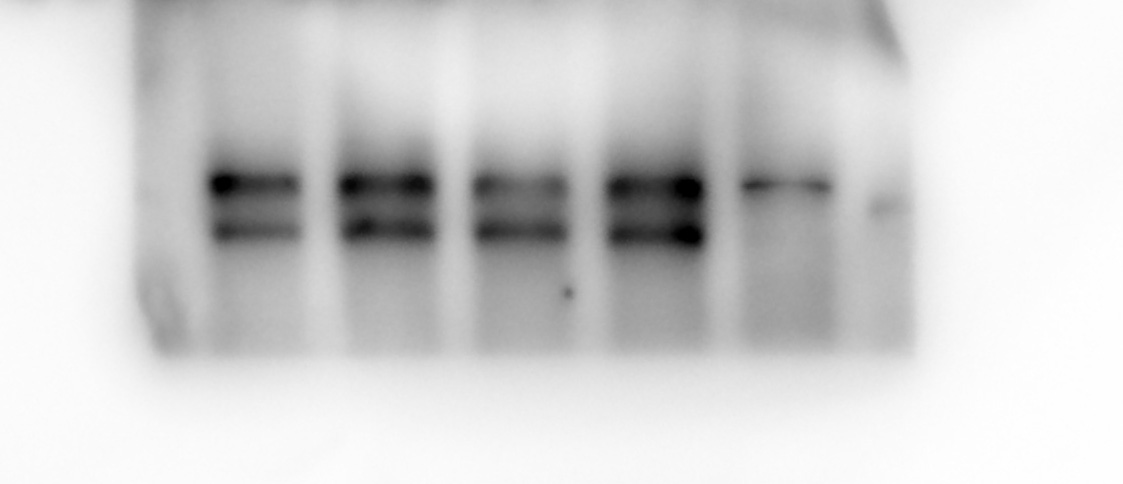

Supplement: Supplementary file 4 — Source Data Fig. 4 [file 44318_2024_66_MOESM4_ESM.zip › Figure 3/B-230401-Rad18-Ser403-endo/RAD18-Input.jpg]

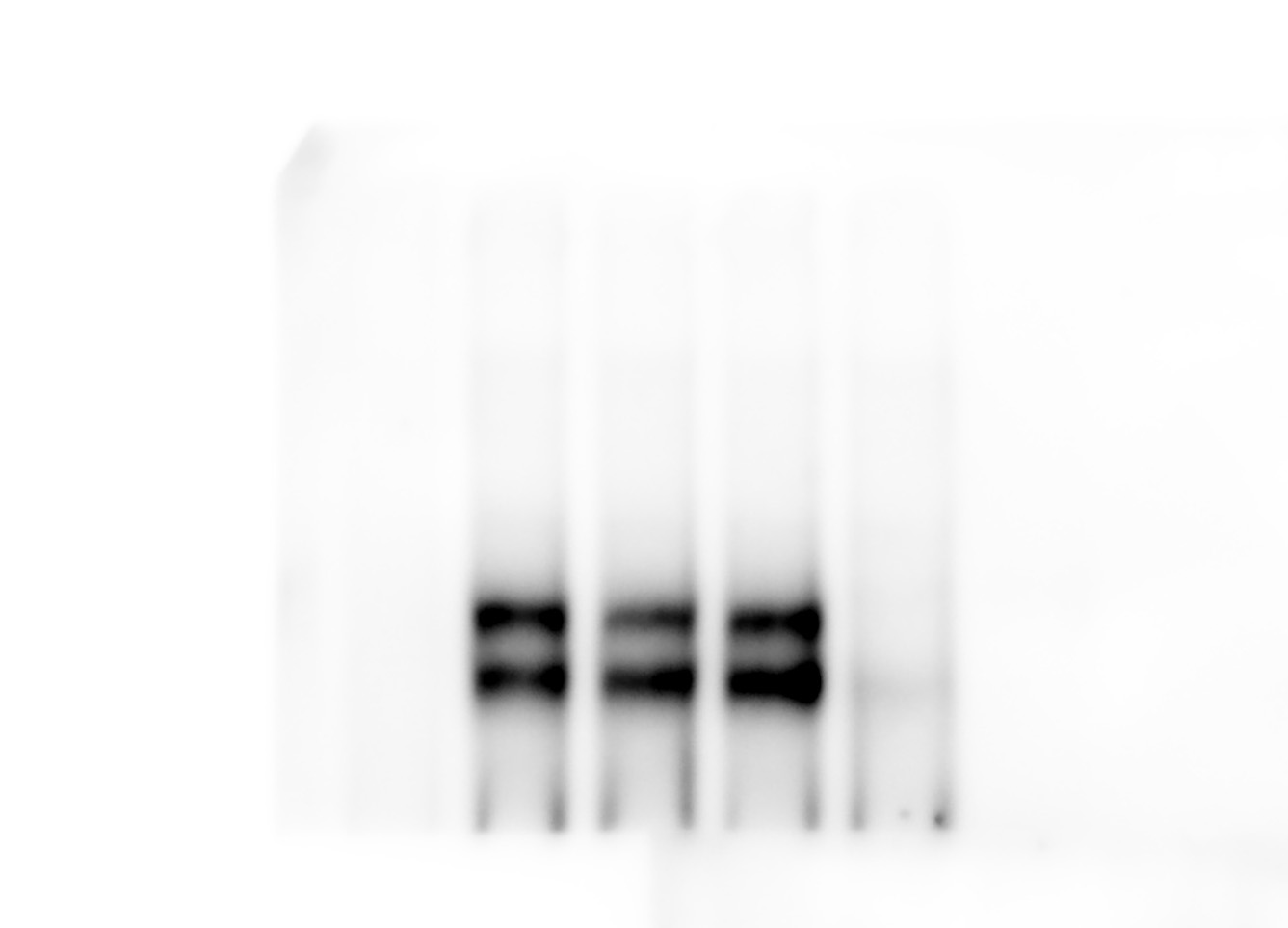

Supplement: Supplementary file 4 — Source Data Fig. 4 [file 44318_2024_66_MOESM4_ESM.zip › Figure 3/B-230401-Rad18-Ser403-endo/RAD18-IP.jpg]

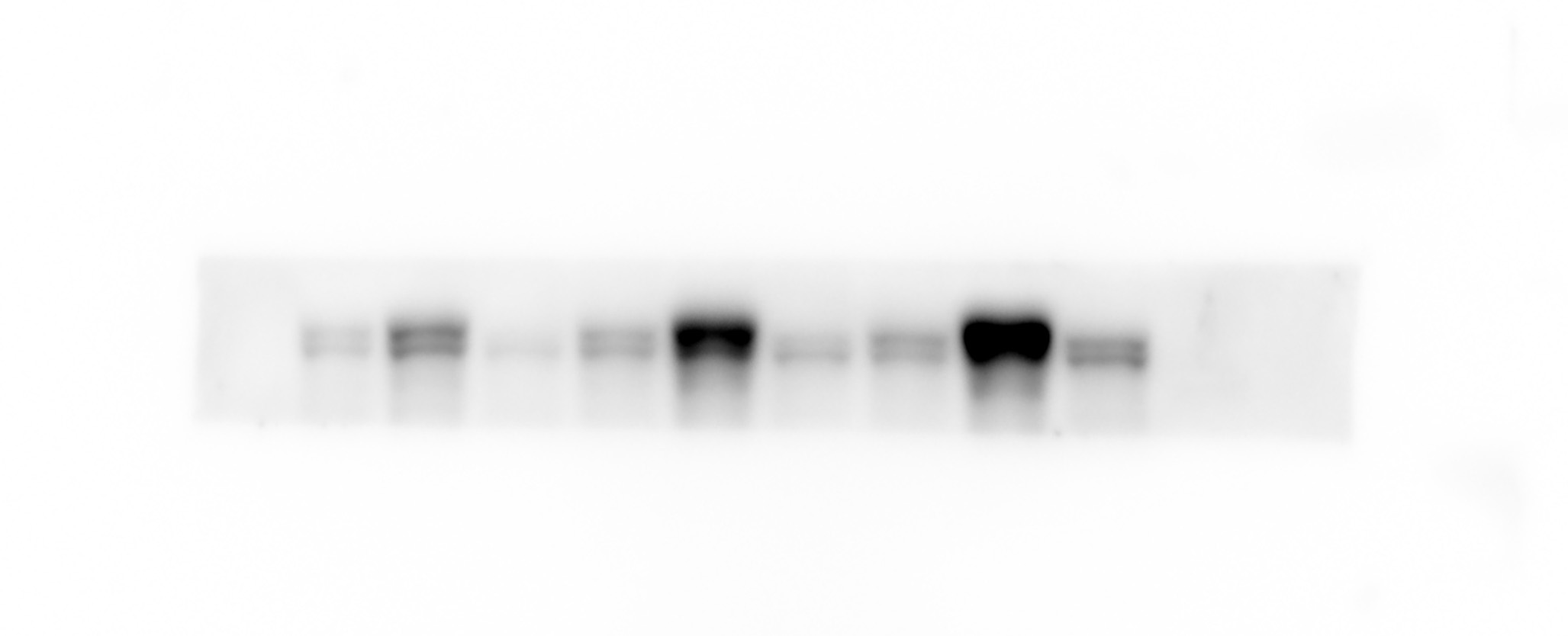

Supplement: Supplementary file 4 — Source Data Fig. 4 [file 44318_2024_66_MOESM4_ESM.zip › Figure 3/K-231114-rH2AX-NIH3T3/p-Chk1-NIH3T3.jpg]

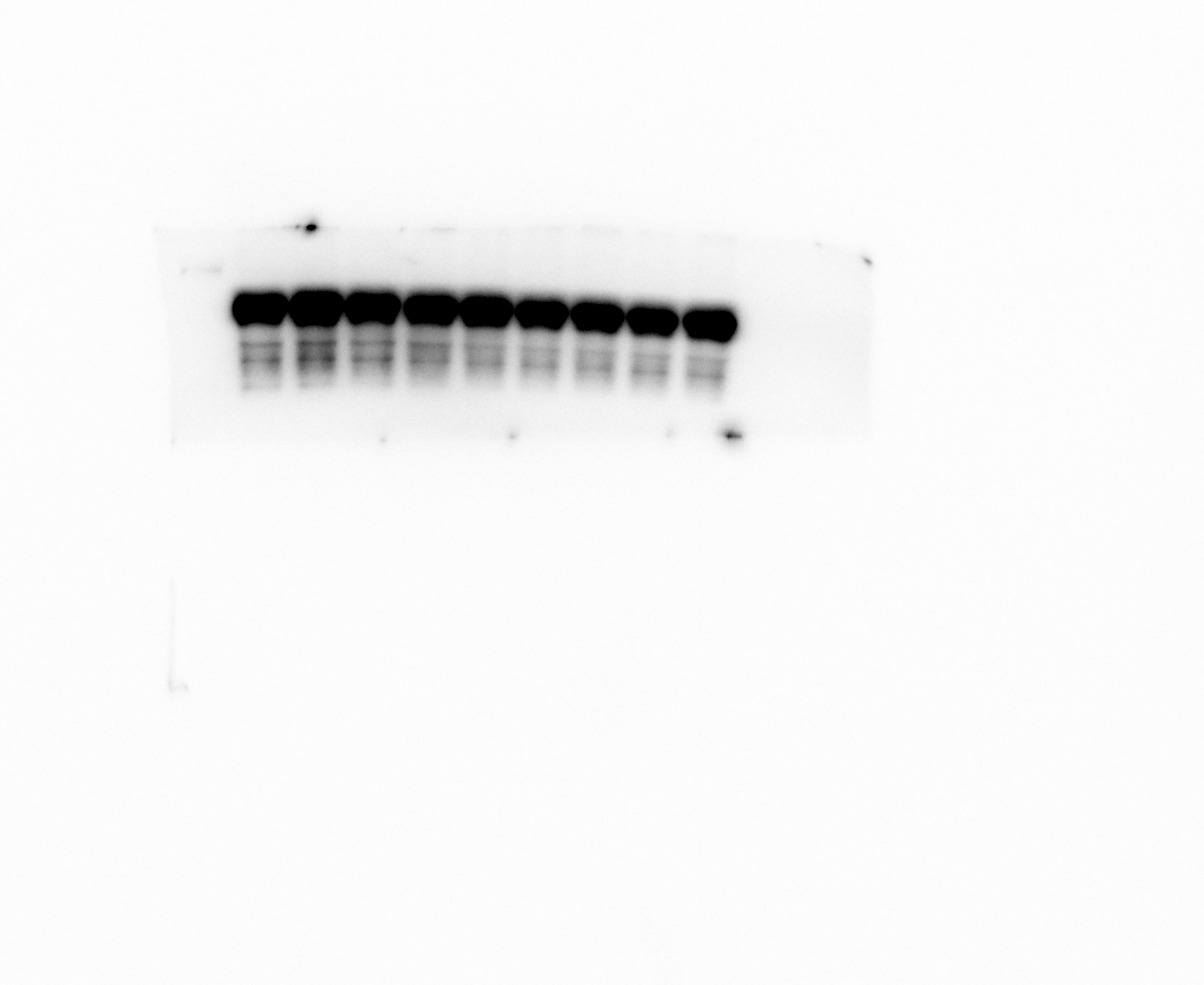

Supplement: Supplementary file 4 — Source Data Fig. 4 [file 44318_2024_66_MOESM4_ESM.zip › Figure 3/K-231114-rH2AX-NIH3T3/PCNA-NIH3T3.tif]

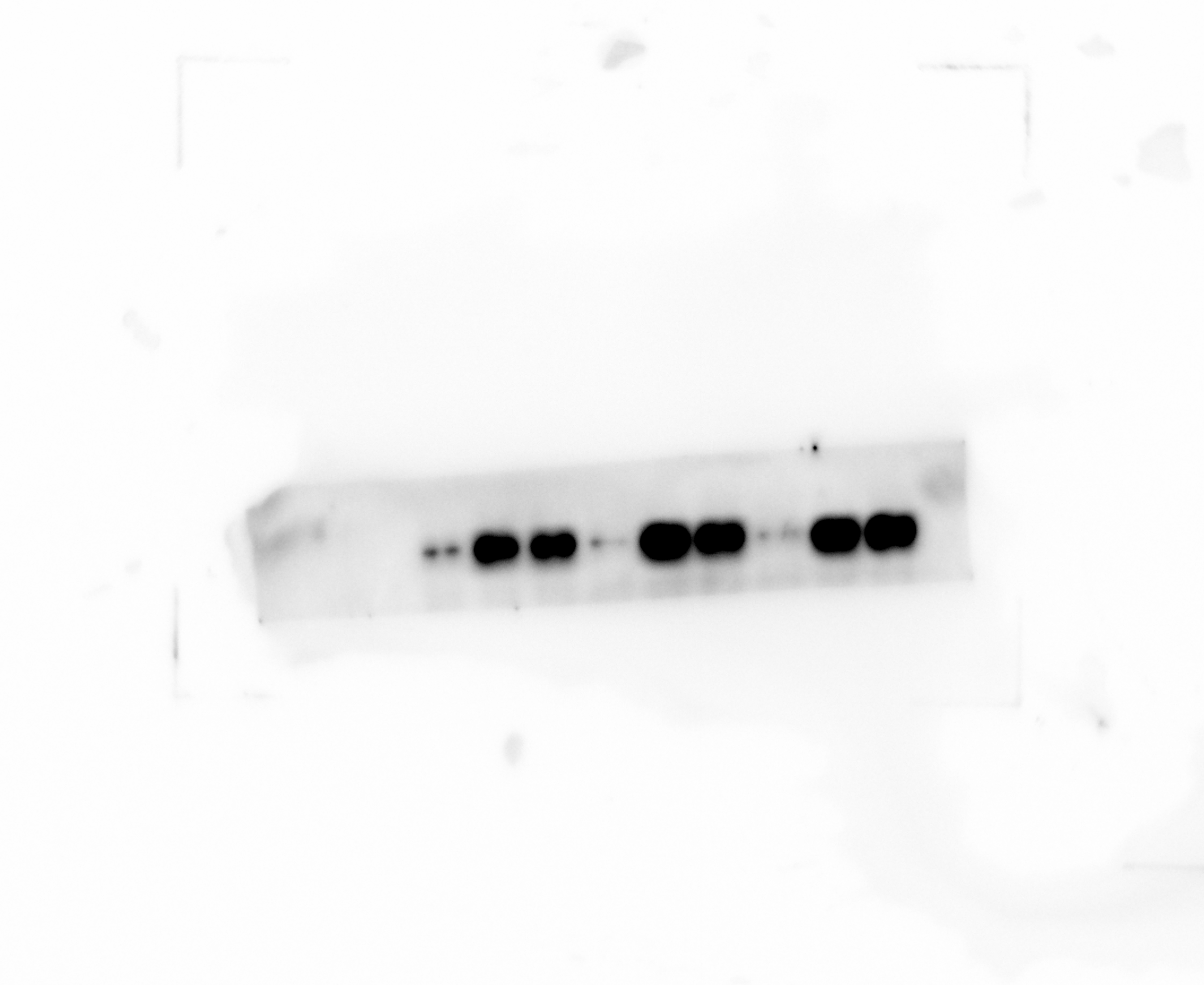

Supplement: Supplementary file 4 — Source Data Fig. 4 [file 44318_2024_66_MOESM4_ESM.zip › Figure 3/K-231114-rH2AX-NIH3T3/H2AX-NIH3T3.tif]

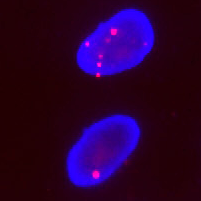

Supplement: Supplementary file 5 — Source Data Fig. 5 [file 44318_2024_66_MOESM5_ESM.zip › Figure 4/E-230913-Rad18ser403 rescue SLX4 PLA-image/siRad18-Vector.tif]

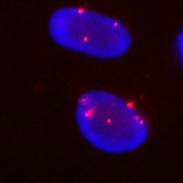

Supplement: Supplementary file 5 — Source Data Fig. 5 [file 44318_2024_66_MOESM5_ESM.zip › Figure 4/E-230913-Rad18ser403 rescue SLX4 PLA-image/siCon-NT.tif]

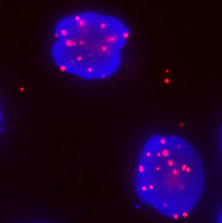

Supplement: Supplementary file 5 — Source Data Fig. 5 [file 44318_2024_66_MOESM5_ESM.zip › Figure 4/E-230913-Rad18ser403 rescue SLX4 PLA-image/siRad18-WT.tif]

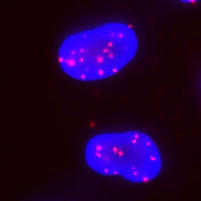

Supplement: Supplementary file 5 — Source Data Fig. 5 [file 44318_2024_66_MOESM5_ESM.zip › Figure 4/E-230913-Rad18ser403 rescue SLX4 PLA-image/siCon-HU+ATRi.tif]

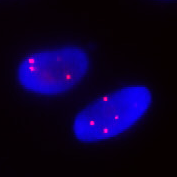

Supplement: Supplementary file 5 — Source Data Fig. 5 [file 44318_2024_66_MOESM5_ESM.zip › Figure 4/E-230913-Rad18ser403 rescue SLX4 PLA-image/siRad18-DEL PIP.tif]

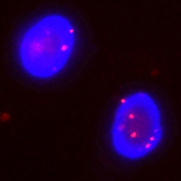

Supplement: Supplementary file 5 — Source Data Fig. 5 [file 44318_2024_66_MOESM5_ESM.zip › Figure 4/E-230913-Rad18ser403 rescue SLX4 PLA-image/siRad18-S403E.tif]

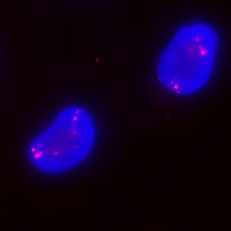

Supplement: Supplementary file 5 — Source Data Fig. 5 [file 44318_2024_66_MOESM5_ESM.zip › Figure 4/E-230913-Rad18ser403 rescue SLX4 PLA-image/siRad18-S403A.tif]

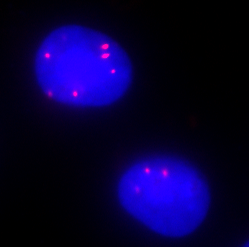

Supplement: Supplementary file 5 — Source Data Fig. 5 [file 44318_2024_66_MOESM5_ESM.zip › Figure 4/C-210918-Rad18 kd SLX4 PLA-image/siCon-NT.tif]

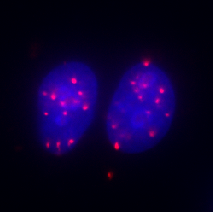

Supplement: Supplementary file 5 — Source Data Fig. 5 [file 44318_2024_66_MOESM5_ESM.zip › Figure 4/C-210918-Rad18 kd SLX4 PLA-image/siCon-HU+ATRi.tif]

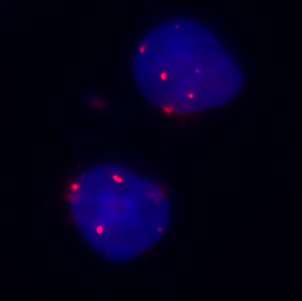

Supplement: Supplementary file 5 — Source Data Fig. 5 [file 44318_2024_66_MOESM5_ESM.zip › Figure 4/C-210918-Rad18 kd SLX4 PLA-image/siRad18#2.tif]

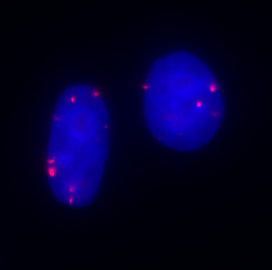

Supplement: Supplementary file 5 — Source Data Fig. 5 [file 44318_2024_66_MOESM5_ESM.zip › Figure 4/C-210918-Rad18 kd SLX4 PLA-image/siRad18#1.tif]

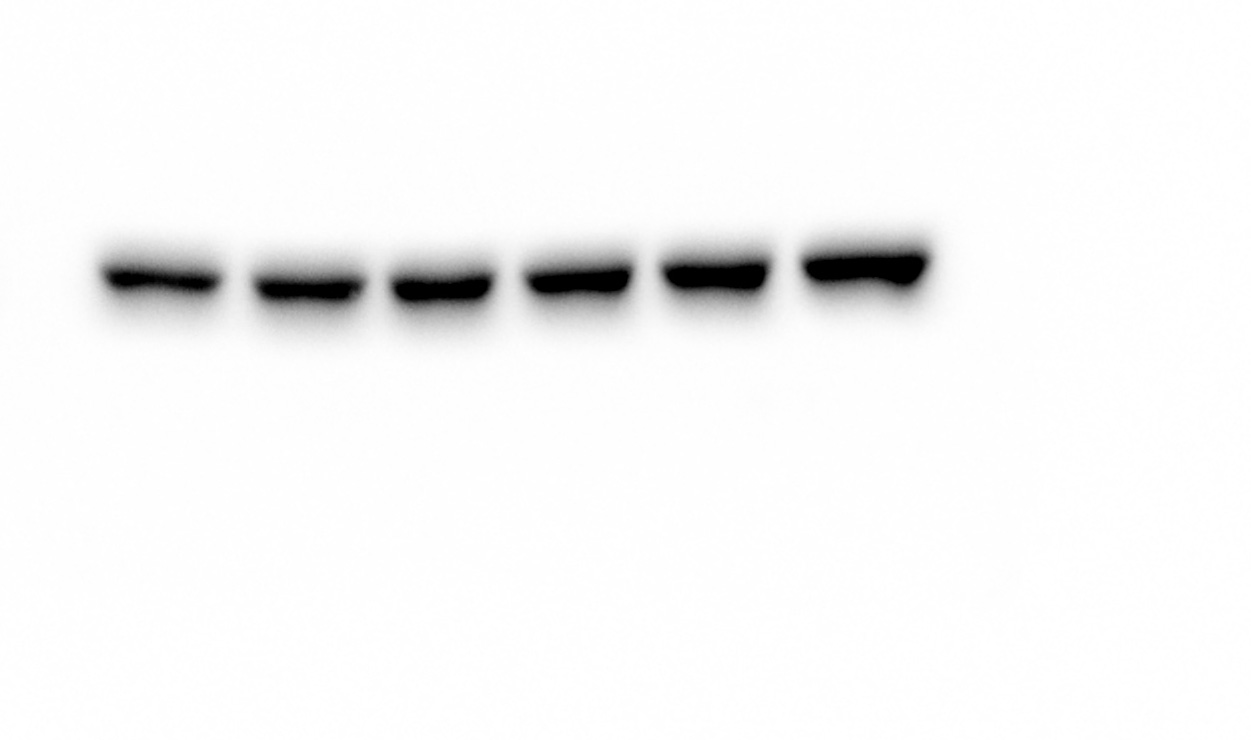

Supplement: Supplementary file 5 — Source Data Fig. 5 [file 44318_2024_66_MOESM5_ESM.zip › Figure 4/H-p-ATM-Rad18:SLX4-Knockdown/TUBULIN.jpg]

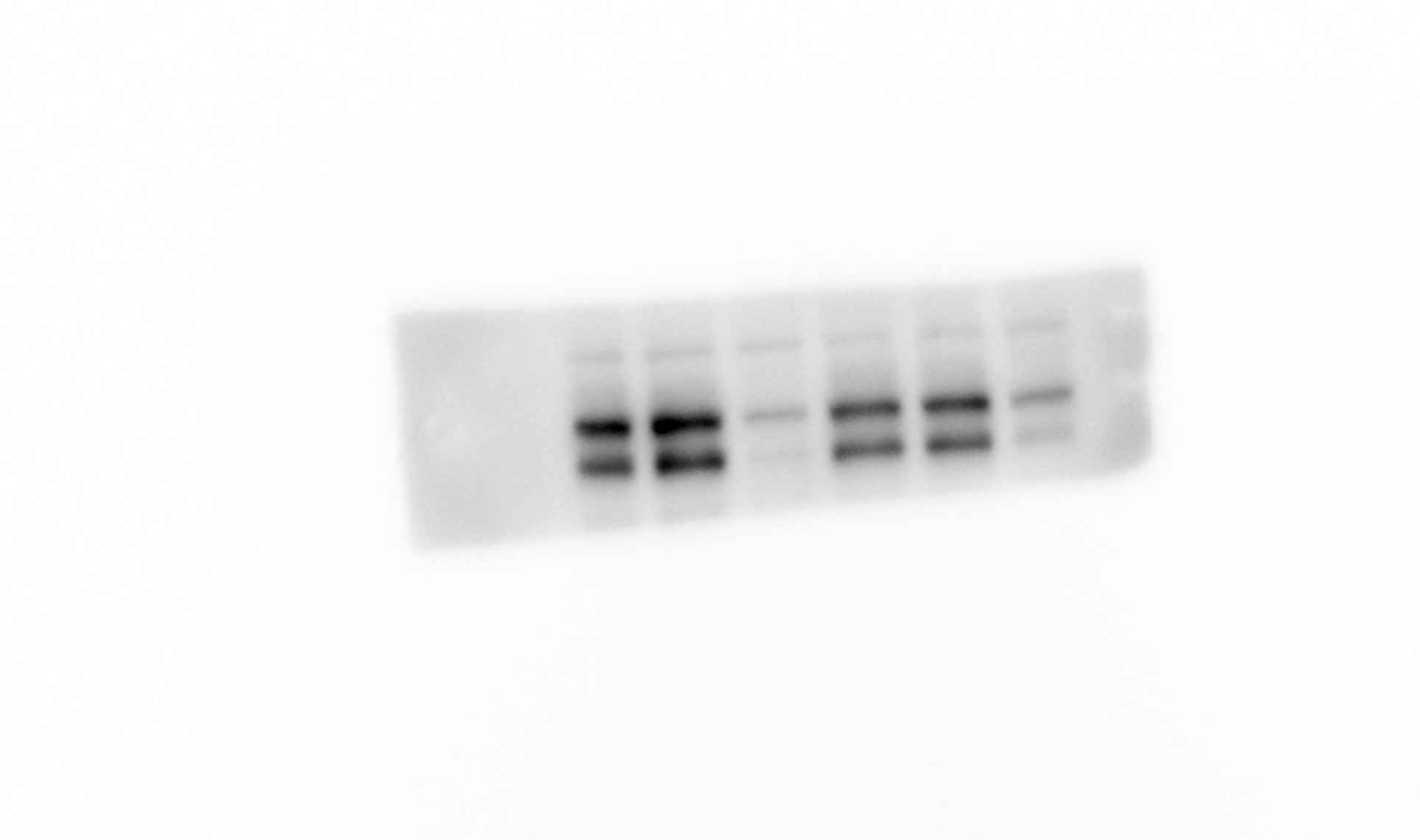

Supplement: Supplementary file 5 — Source Data Fig. 5 [file 44318_2024_66_MOESM5_ESM.zip › Figure 4/H-p-ATM-Rad18:SLX4-Knockdown/rad18-knockdown.jpg]

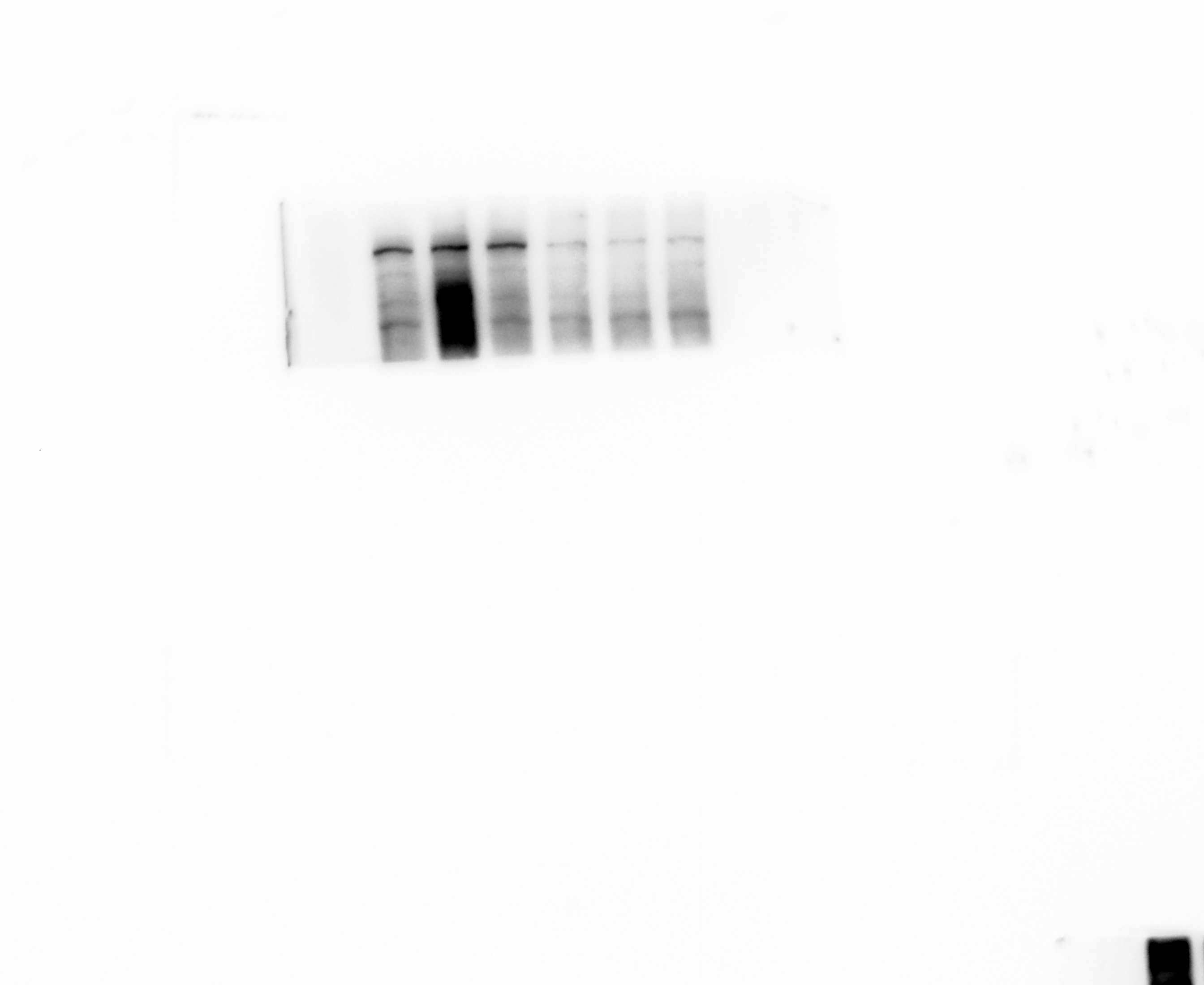

Supplement: Supplementary file 5 — Source Data Fig. 5 [file 44318_2024_66_MOESM5_ESM.zip › Figure 4/H-p-ATM-Rad18:SLX4-Knockdown/slx4-knockdown.jpg]

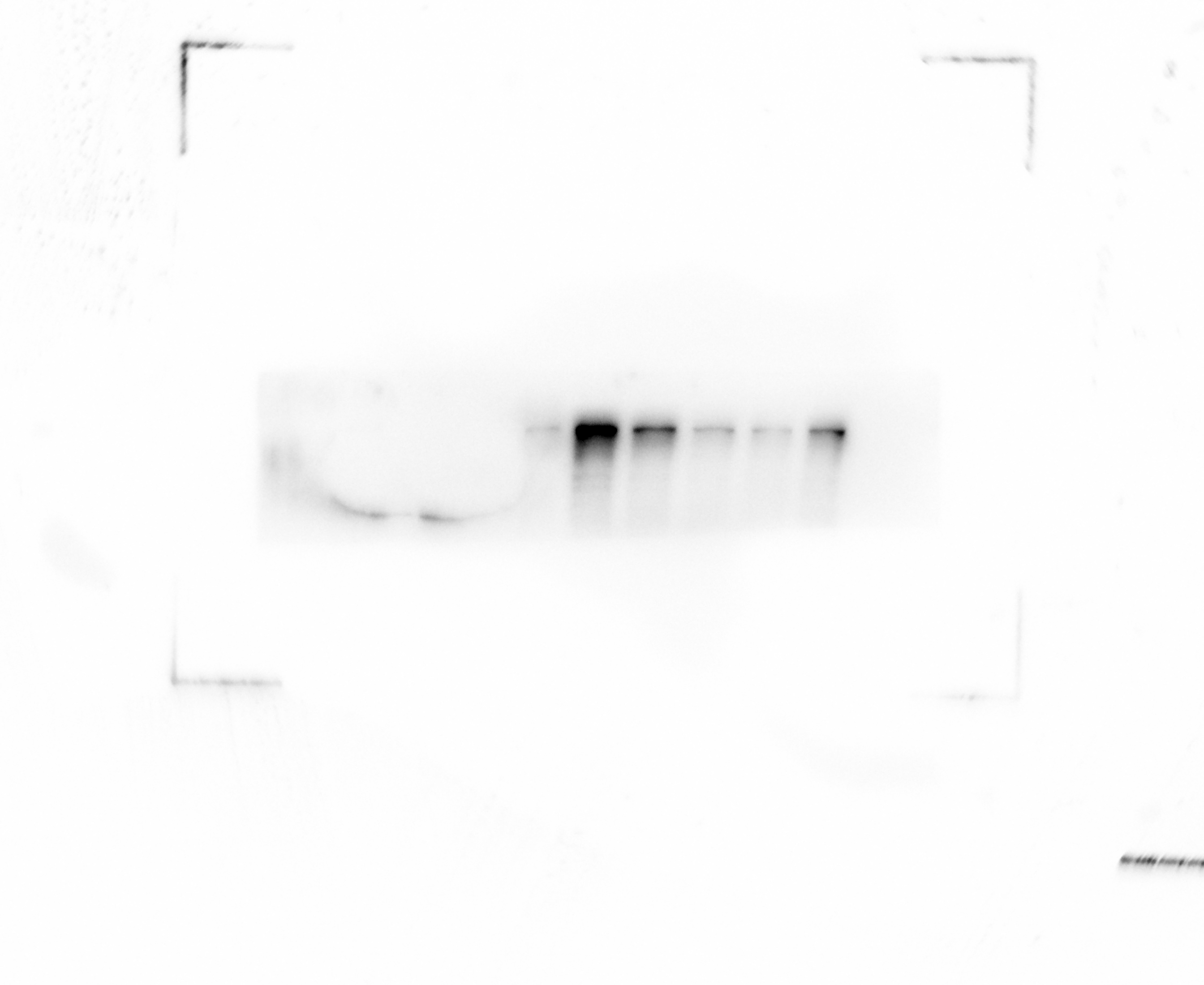

Supplement: Supplementary file 5 — Source Data Fig. 5 [file 44318_2024_66_MOESM5_ESM.zip › Figure 4/H-p-ATM-Rad18:SLX4-Knockdown/pSer1981-atm.jpg]

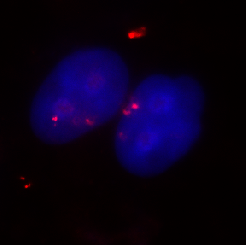

Supplement: Supplementary file 5 — Source Data Fig. 5 [file 44318_2024_66_MOESM5_ESM.zip › Figure 4/A-210713-SLX4-PLA-Image/siSLX4 Knockdown.tif]

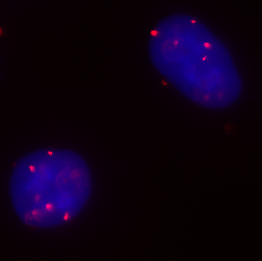

Supplement: Supplementary file 5 — Source Data Fig. 5 [file 44318_2024_66_MOESM5_ESM.zip › Figure 4/A-210713-SLX4-PLA-Image/NT.tif]

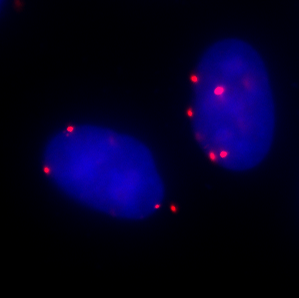

Supplement: Supplementary file 5 — Source Data Fig. 5 [file 44318_2024_66_MOESM5_ESM.zip › Figure 4/A-210713-SLX4-PLA-Image/HU.tif]

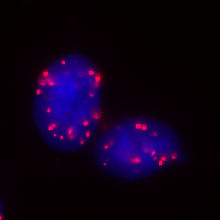

Supplement: Supplementary file 5 — Source Data Fig. 5 [file 44318_2024_66_MOESM5_ESM.zip › Figure 4/A-210713-SLX4-PLA-Image/HU+ATRi.tif]

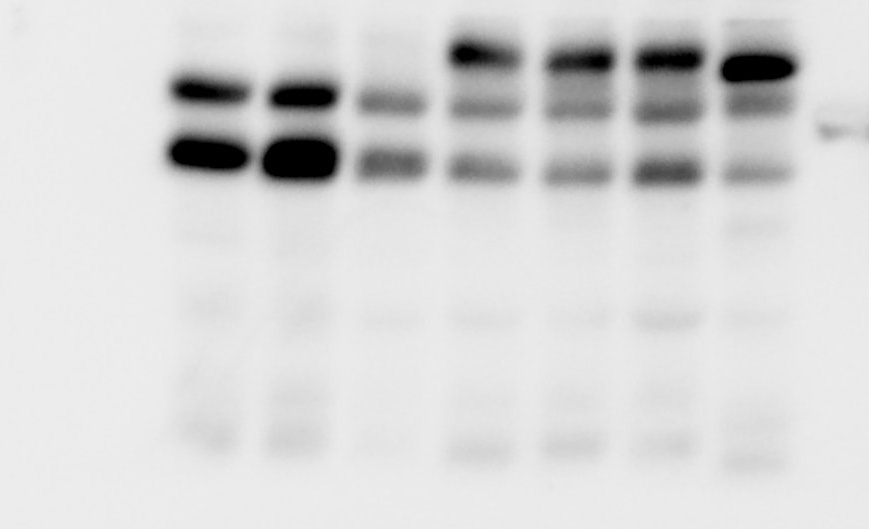

Supplement: Supplementary file 5 — Source Data Fig. 5 [file 44318_2024_66_MOESM5_ESM.zip › Figure 4/G-p-ATM-Rescue/Rad18-knockwodn.jpg]

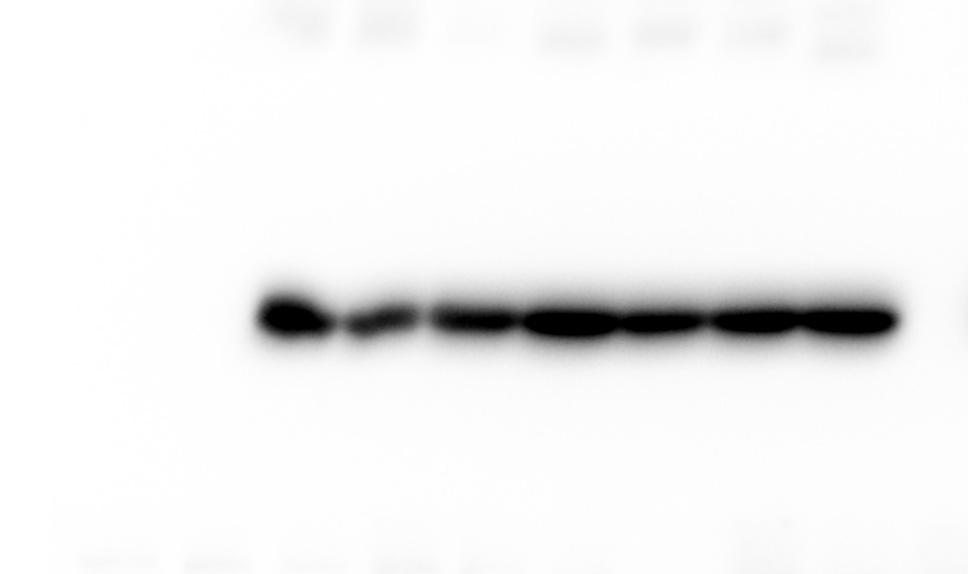

Supplement: Supplementary file 5 — Source Data Fig. 5 [file 44318_2024_66_MOESM5_ESM.zip › Figure 4/G-p-ATM-Rescue/gapdh.jpg]

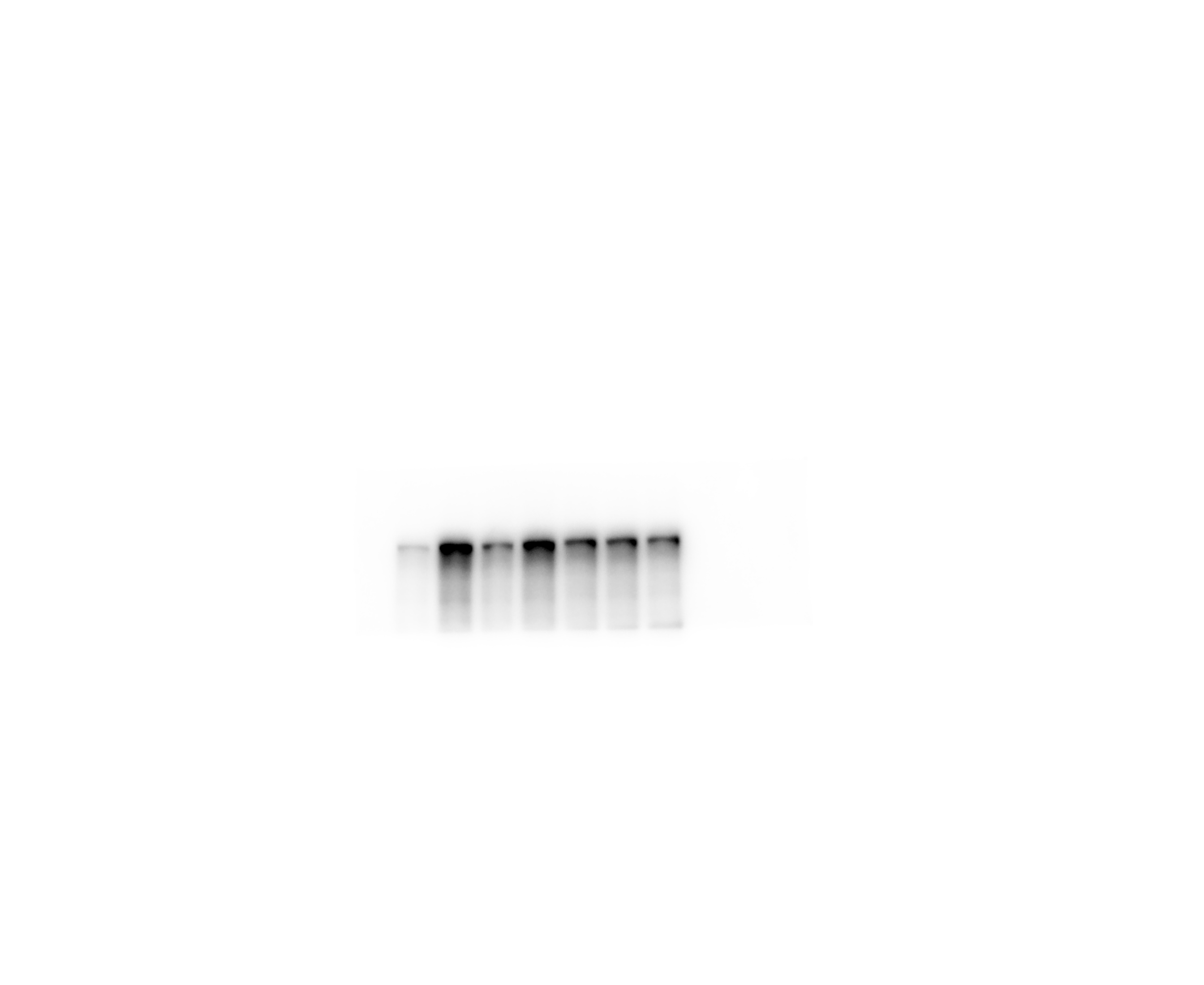

Supplement: Supplementary file 5 — Source Data Fig. 5 [file 44318_2024_66_MOESM5_ESM.zip › Figure 4/G-p-ATM-Rescue/pSer1981-atm.jpg]

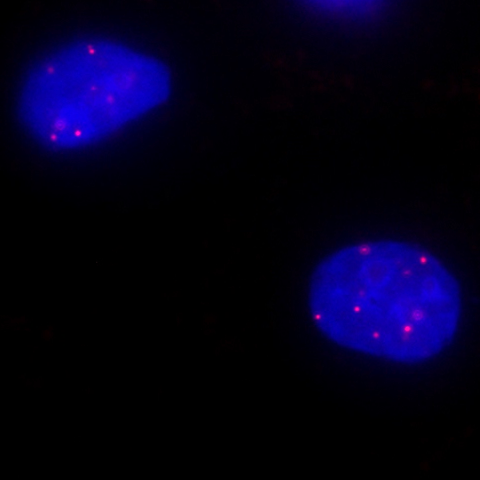

Supplement: Supplementary file 6 — Source Data Fig. 6 [file 44318_2024_66_MOESM6_ESM.zip › Figure 6/F-230411-image-SLX4:TRF1-PLA/Rad18#1.tif]

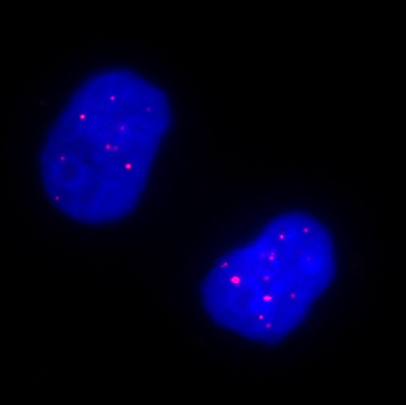

Supplement: Supplementary file 6 — Source Data Fig. 6 [file 44318_2024_66_MOESM6_ESM.zip › Figure 6/F-230411-image-SLX4:TRF1-PLA/Rad18#2.tif]

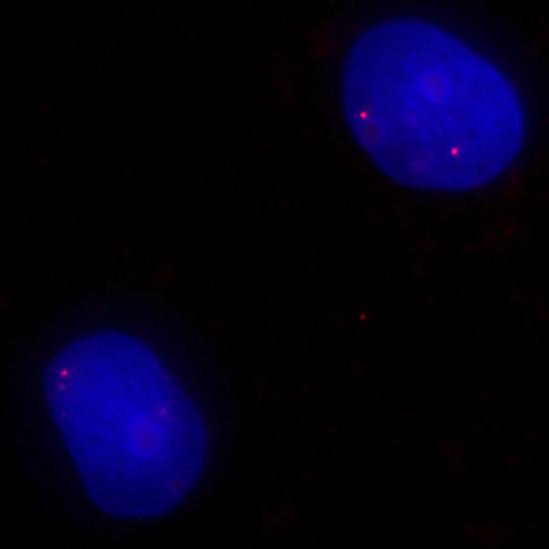

Supplement: Supplementary file 6 — Source Data Fig. 6 [file 44318_2024_66_MOESM6_ESM.zip › Figure 6/F-230411-image-SLX4:TRF1-PLA/SLX4#1.tif]

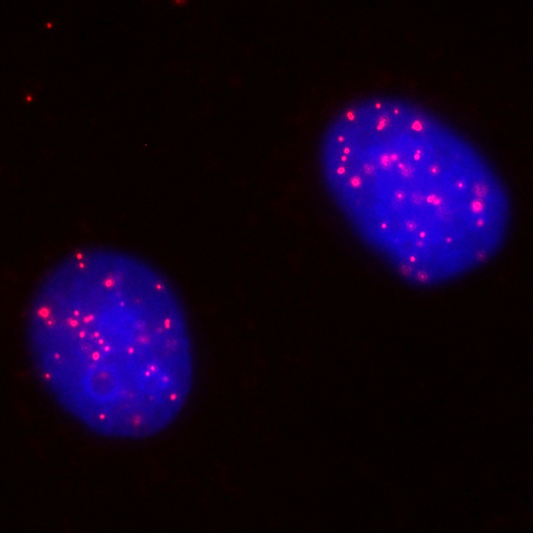

Supplement: Supplementary file 6 — Source Data Fig. 6 [file 44318_2024_66_MOESM6_ESM.zip › Figure 6/F-230411-image-SLX4:TRF1-PLA/con+.tif]

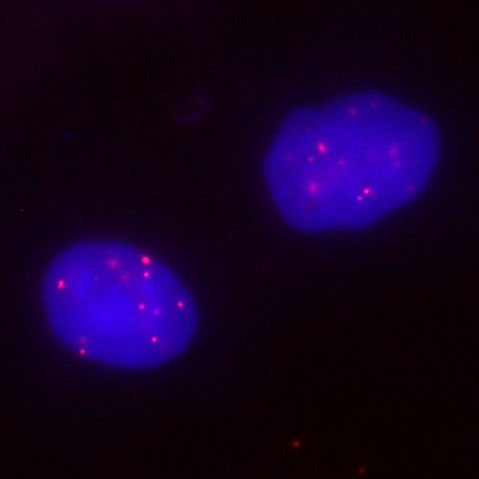

Supplement: Supplementary file 6 — Source Data Fig. 6 [file 44318_2024_66_MOESM6_ESM.zip › Figure 6/F-230411-image-SLX4:TRF1-PLA/con-.tif]

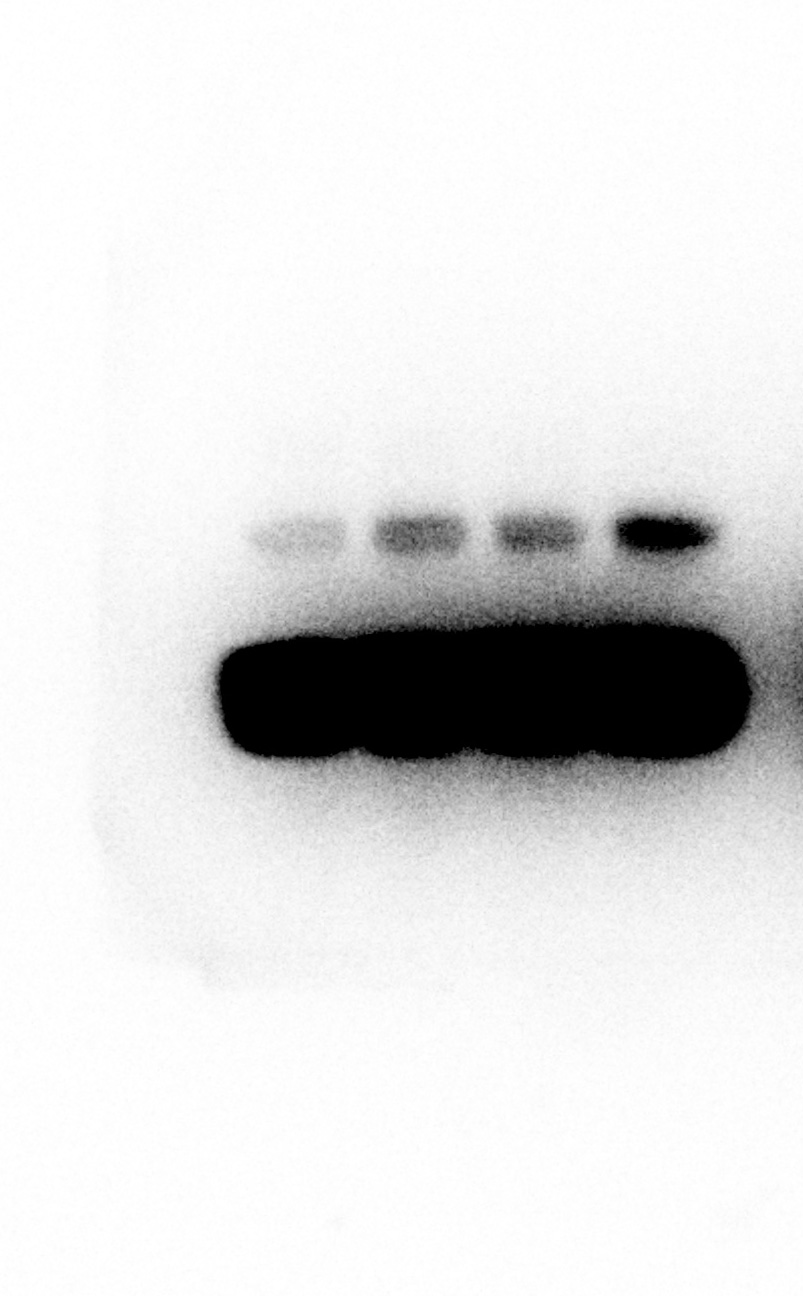

Supplement: Supplementary file 6 — Source Data Fig. 6 [file 44318_2024_66_MOESM6_ESM.zip › Figure 6/B-220910-U2OS-PCNA-ENDO-Thy-CDK1i/pcna-ub-long.jpg]

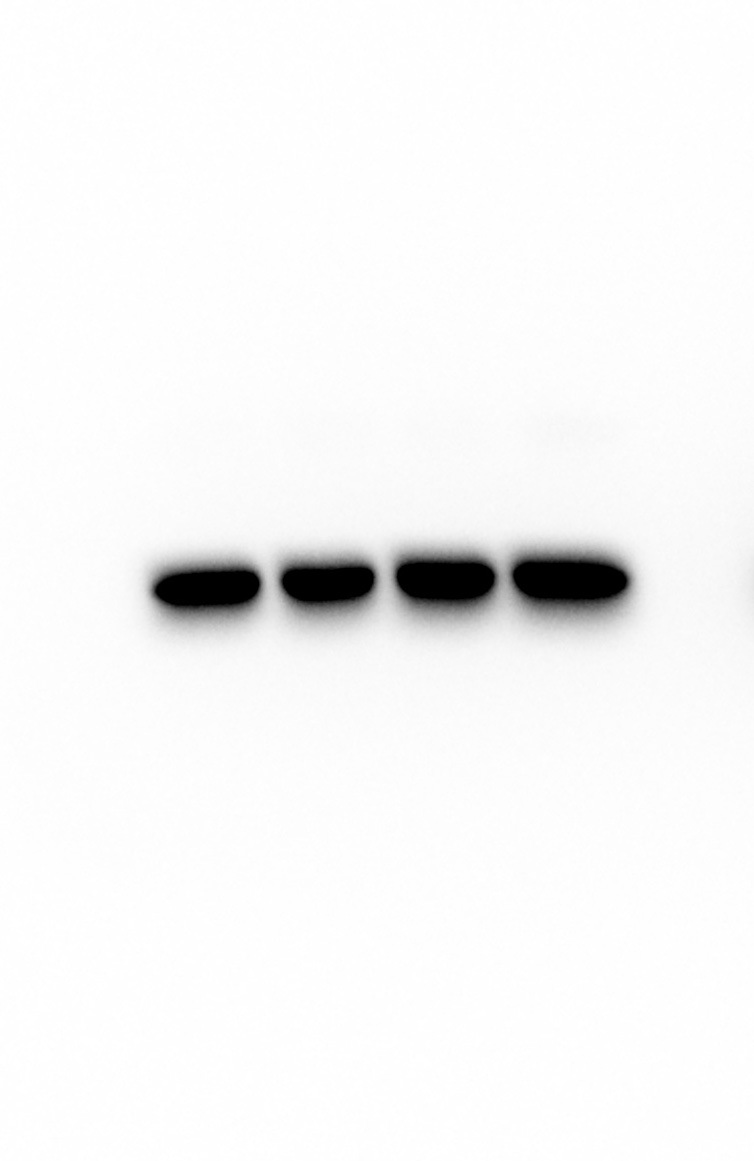

Supplement: Supplementary file 6 — Source Data Fig. 6 [file 44318_2024_66_MOESM6_ESM.zip › Figure 6/B-220910-U2OS-PCNA-ENDO-Thy-CDK1i/pcna-ub-short.jpg]

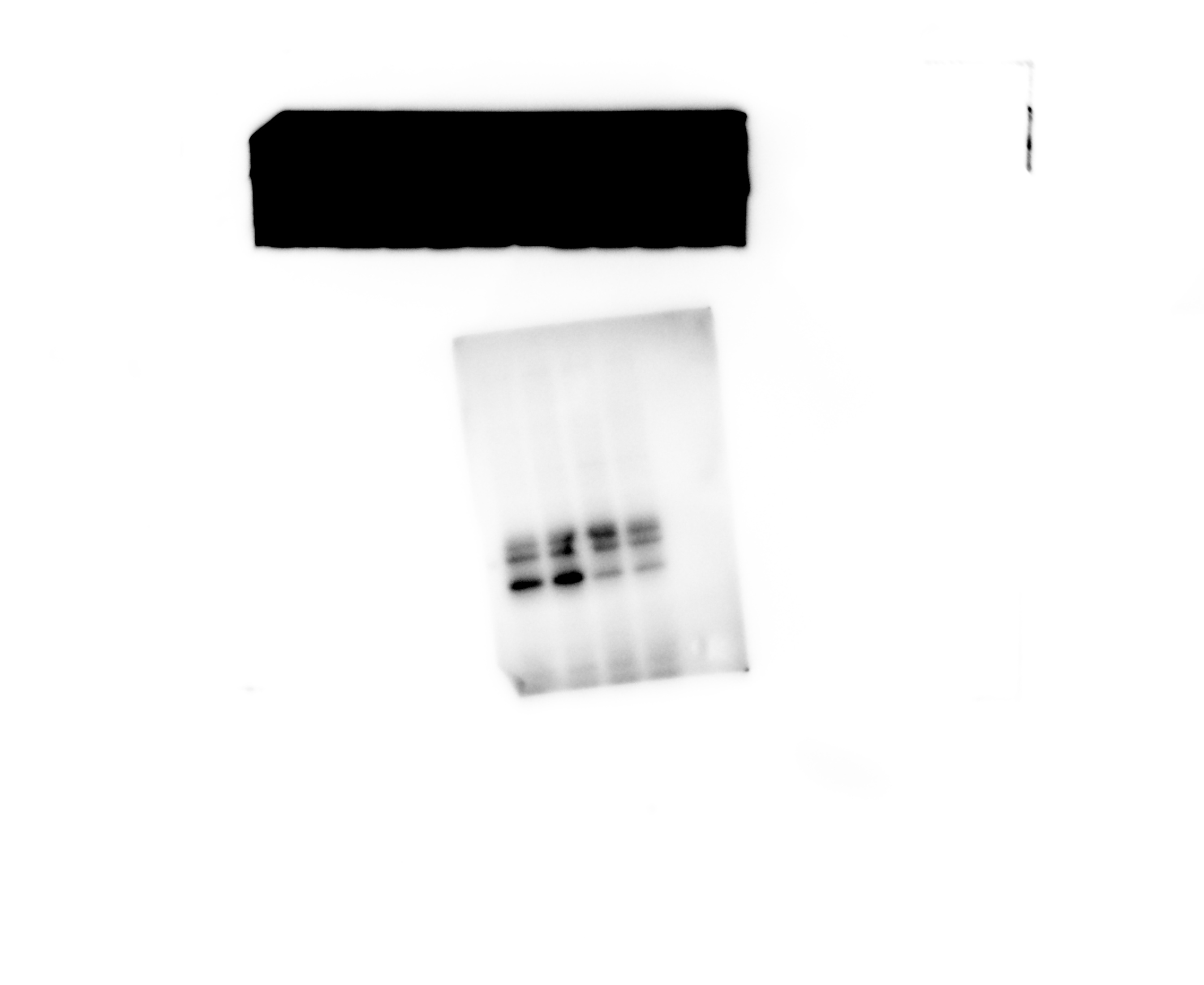

Supplement: Supplementary file 6 — Source Data Fig. 6 [file 44318_2024_66_MOESM6_ESM.zip › Figure 6/J-230909-KnockDown-Western/Rad18-Knockdown.jpg]

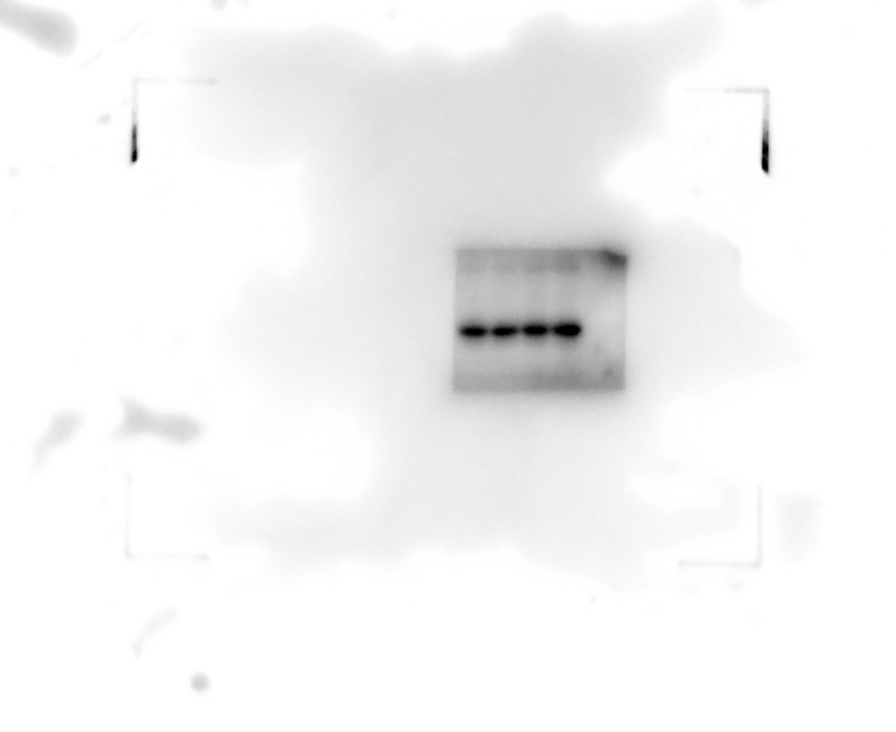

Supplement: Supplementary file 6 — Source Data Fig. 6 [file 44318_2024_66_MOESM6_ESM.zip › Figure 6/J-230909-KnockDown-Western/GAPDH.jpg]

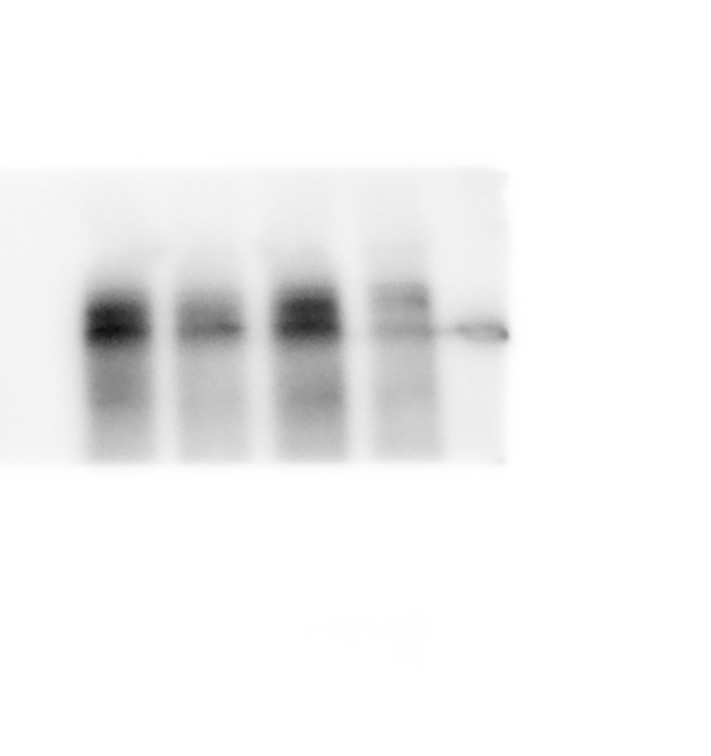

Supplement: Supplementary file 6 — Source Data Fig. 6 [file 44318_2024_66_MOESM6_ESM.zip › Figure 6/J-230909-KnockDown-Western/slx4-Knockdown.jpg]

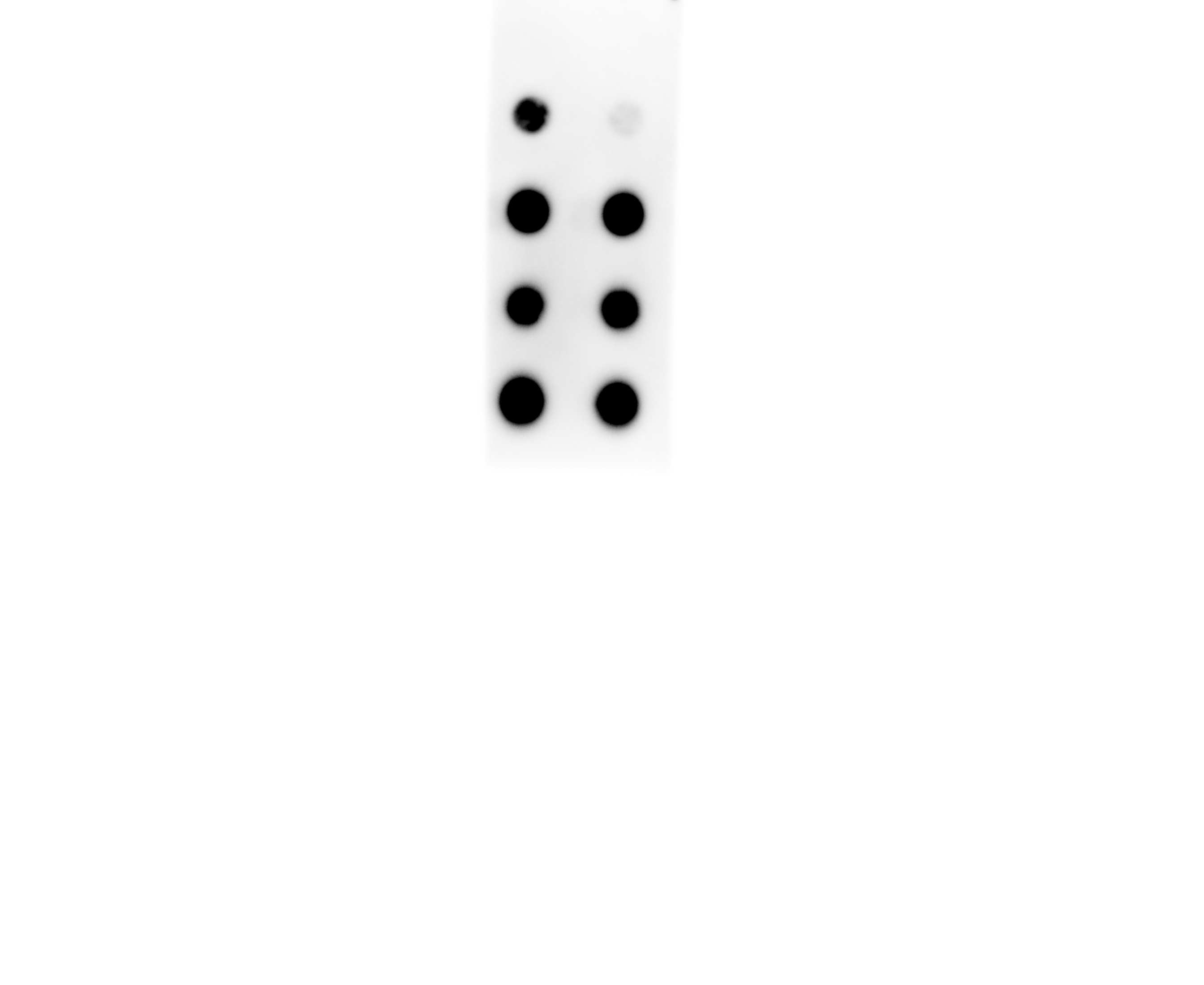

Supplement: Supplementary file 6 — Source Data Fig. 6 [file 44318_2024_66_MOESM6_ESM.zip › Figure 6/I-230903-ALT-C circle Blot/ALT-Blot.jpg]

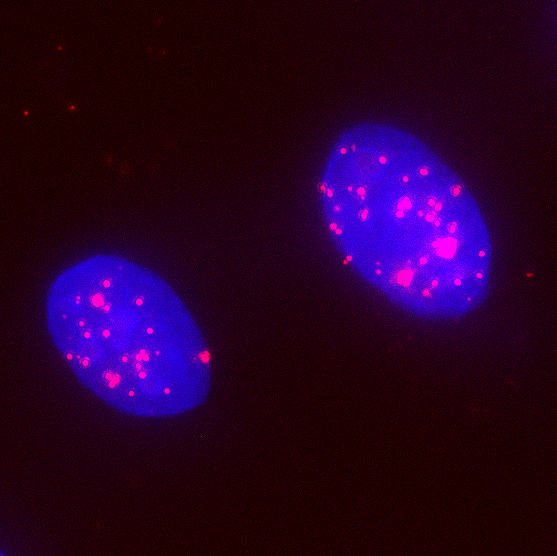

Supplement: Supplementary file 6 — Source Data Fig. 6 [file 44318_2024_66_MOESM6_ESM.zip › Figure 6/D-231110-image-PLA-U2OS-G1:S:G2-SLX4-TRF1/G1-.tif]

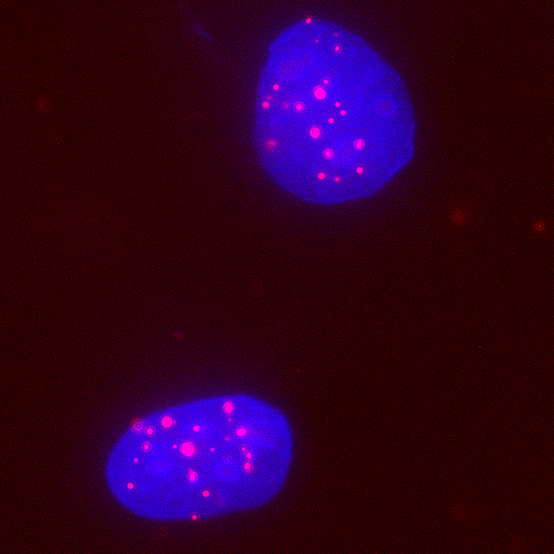

Supplement: Supplementary file 6 — Source Data Fig. 6 [file 44318_2024_66_MOESM6_ESM.zip › Figure 6/D-231110-image-PLA-U2OS-G1:S:G2-SLX4-TRF1/G1+.tif]

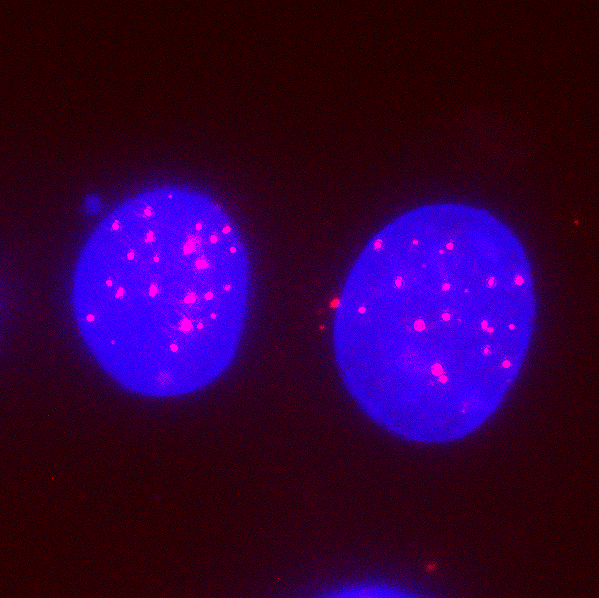

Supplement: Supplementary file 6 — Source Data Fig. 6 [file 44318_2024_66_MOESM6_ESM.zip › Figure 6/D-231110-image-PLA-U2OS-G1:S:G2-SLX4-TRF1/G2+.tif]

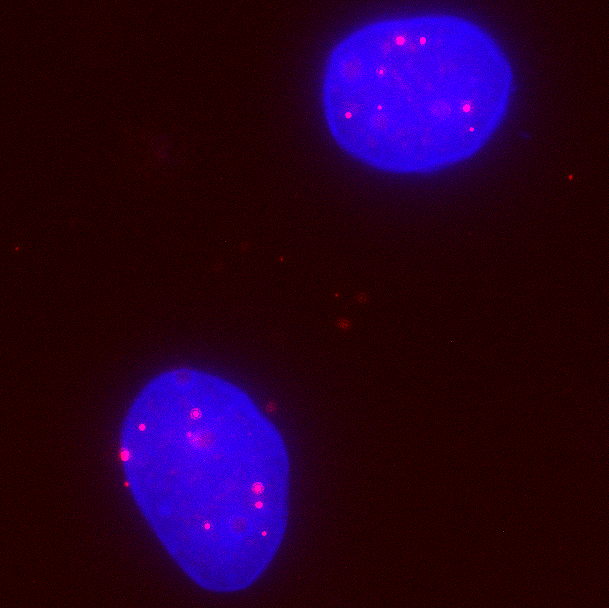

Supplement: Supplementary file 6 — Source Data Fig. 6 [file 44318_2024_66_MOESM6_ESM.zip › Figure 6/D-231110-image-PLA-U2OS-G1:S:G2-SLX4-TRF1/G2-.tif]

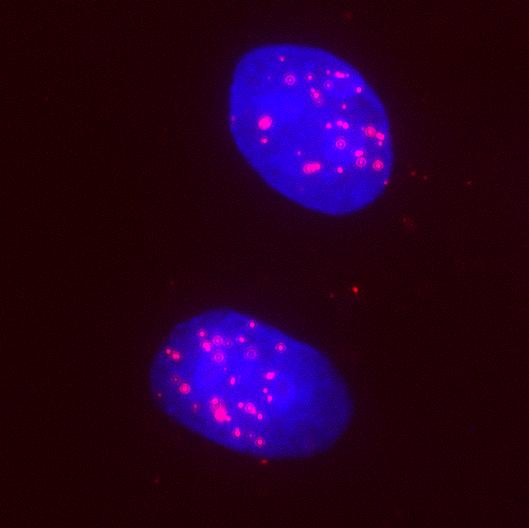

Supplement: Supplementary file 6 — Source Data Fig. 6 [file 44318_2024_66_MOESM6_ESM.zip › Figure 6/D-231110-image-PLA-U2OS-G1:S:G2-SLX4-TRF1/S-.tif]

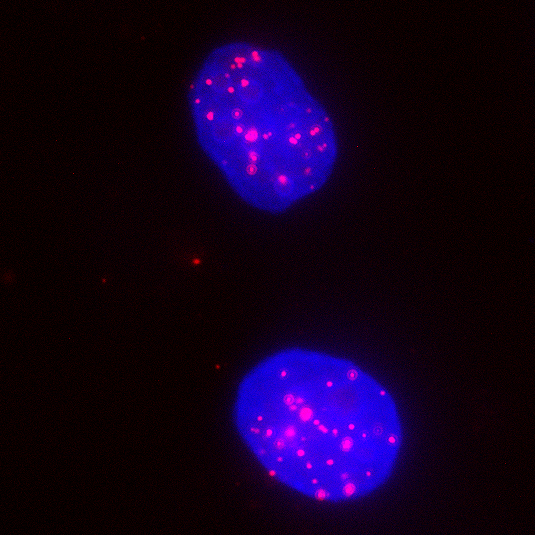

Supplement: Supplementary file 6 — Source Data Fig. 6 [file 44318_2024_66_MOESM6_ESM.zip › Figure 6/D-231110-image-PLA-U2OS-G1:S:G2-SLX4-TRF1/S+.tif]

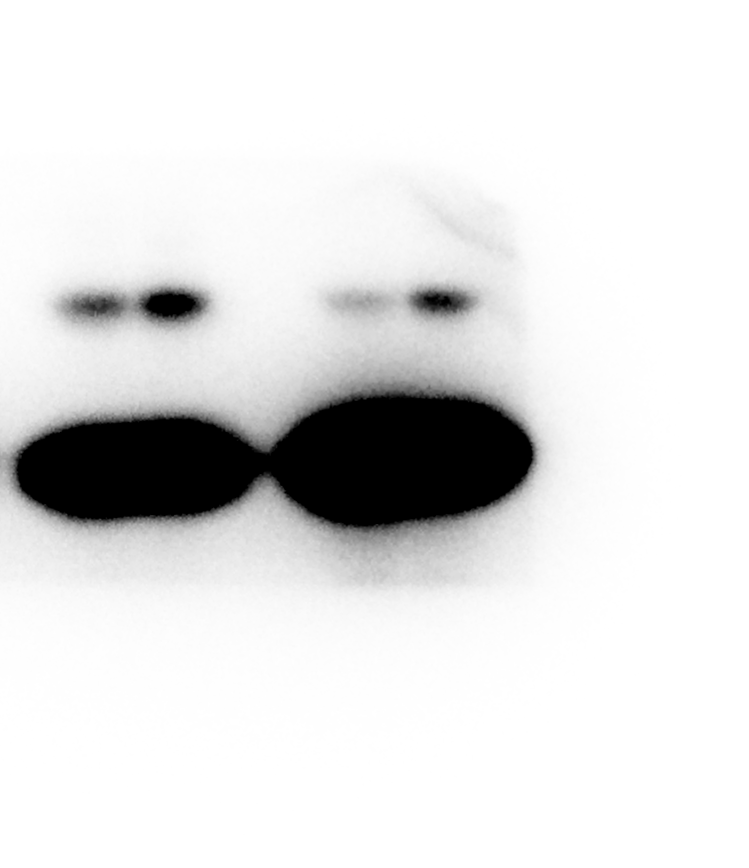

Supplement: Supplementary file 6 — Source Data Fig. 6 [file 44318_2024_66_MOESM6_ESM.zip › Figure 6/C-PCNA-Ub-U2OS-G2:S/PCNA-long-G2-S.tif]

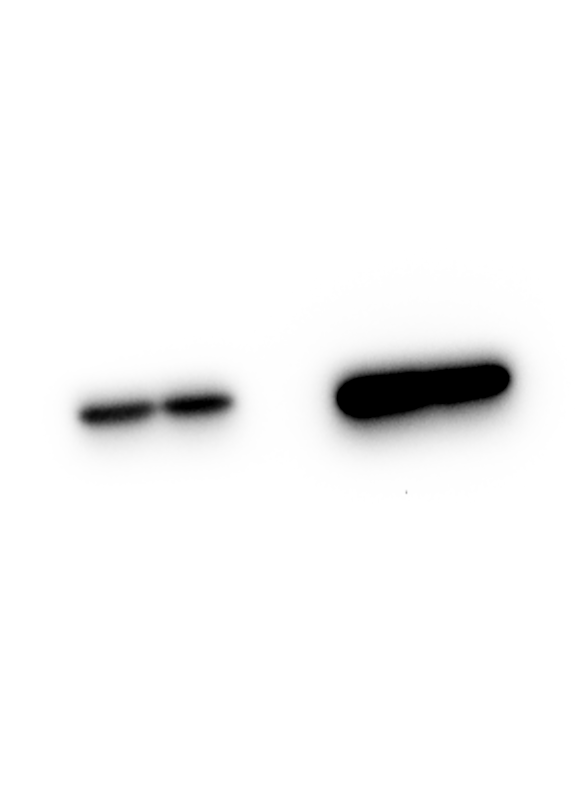

Supplement: Supplementary file 6 — Source Data Fig. 6 [file 44318_2024_66_MOESM6_ESM.zip › Figure 6/C-PCNA-Ub-U2OS-G2:S/PCNA-short-G2-S.tif]

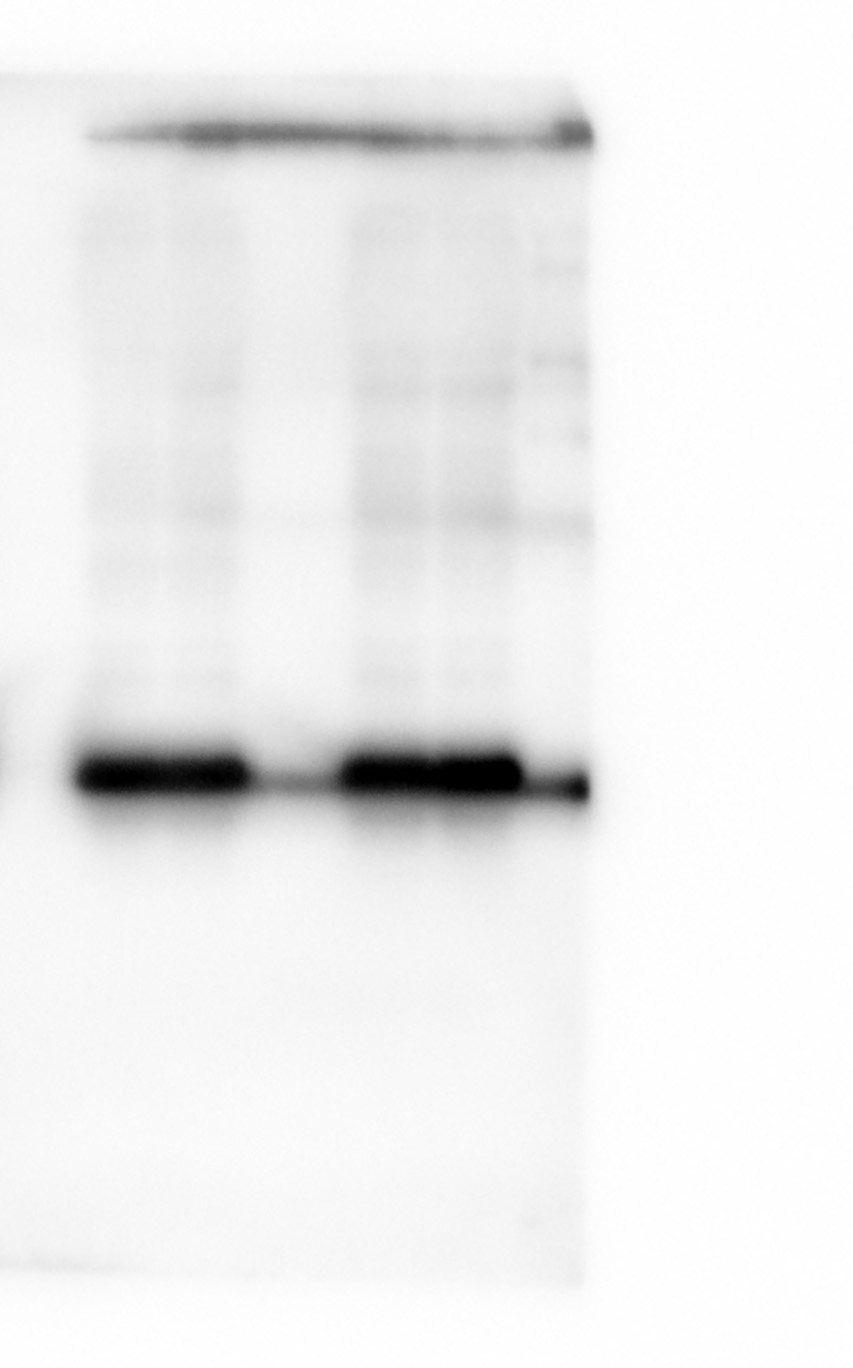

Supplement: Supplementary file 6 — Source Data Fig. 6 [file 44318_2024_66_MOESM6_ESM.zip › Figure 6/C-PCNA-Ub-U2OS-G2:S/H3.jpg]

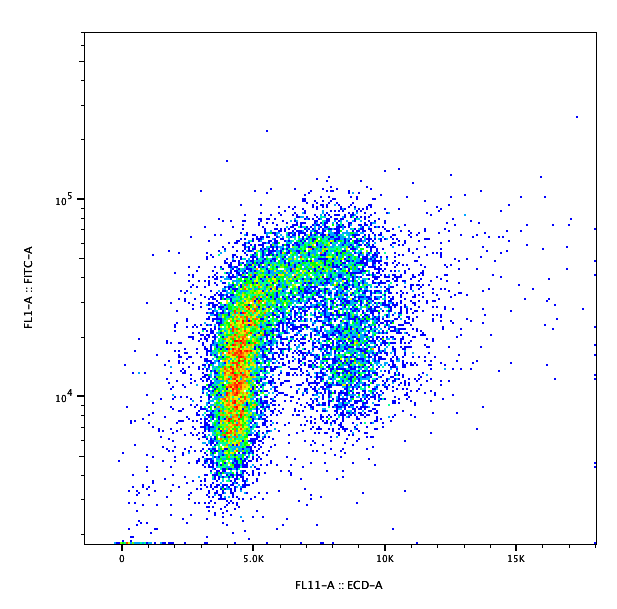

Supplement: Supplementary file 6 — Source Data Fig. 6 [file 44318_2024_66_MOESM6_ESM.zip › Figure 6/A-220726-cell cycle-flow/Asynchronous.png]

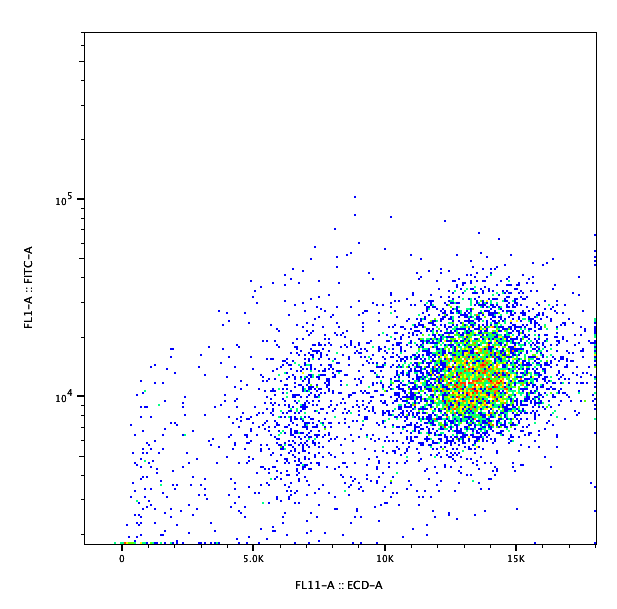

Supplement: Supplementary file 6 — Source Data Fig. 6 [file 44318_2024_66_MOESM6_ESM.zip › Figure 6/A-220726-cell cycle-flow/G2 Phase.png]

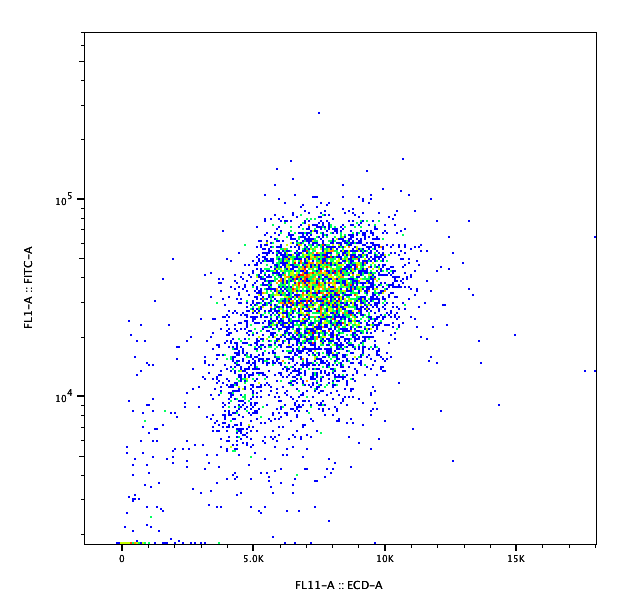

Supplement: Supplementary file 6 — Source Data Fig. 6 [file 44318_2024_66_MOESM6_ESM.zip › Figure 6/A-220726-cell cycle-flow/S phase.png]
