# Supplementary material for: ATR limits Rad18-mediated PCNA monoubiquitination to preserve replication fork and telomerase-independent telomere stability
Source: EMBO J. 2024 Mar 11;43(7):9. doi: 10.1038/s44318-024-00066-9 (PMC10987609; doi:10.1038/s44318-024-00066-9)
Supplement: Supplementary file 8 — Expanded View Figures [file 44318_2024_66_MOESM8_ESM.pdf]

Expanded View Figures

Figure EV1. ATR inactivation hampered the restart of stalled forks.

(**A**) Rad18 depletion improves fork restart efficiency in the absence of ATR under replication stress. Top: Schematic of the DNA fiber experiment. HeLa cells were transfected with the indicated siRNAs. Forty-eight hours after transfection, cells were incubated with 50 μM IdU for 30 min, treated with 2 mM HU with or without 2 μM VE-821 for 1 h, and incubated with 100 μM CldU for 30 min. Bottom: representative IdU and CldU replication tracks in cells transfected with indicated siRNAs. (**B**) Dot plot of CldU to IdU track length ratios for individual replication forks. Data represent means ± SD of three independent experiments. More than 100 fibers were analysed for each sample. n.s. indicates not significant, ****$P < 0.0001$, one-way ANOVA test. (**C**)Short-term ATR inhibition does not affect fork degradation under replication stress. Top: schematic of the DNA fiber experiment. HeLa cells were incubated with 50 μM IdU for 30 min, washed with PBS three times, and incubated with 100 μM CldU for 30 min. Then, cells were washed with PBS three times and treated with 2 mM HU with or without 2 μM VE-821 for 1 h. Bottom: representative IdU and CldU replication tracks. (**D**) Dot plot of CldU to IdU track length ratios for individual replication forks. Data represent means ± SD of three independent experiments. More than 100 fibers were analysed for each sample. n.s. indicates not significant, one-way ANOVA test. (**E**) Simultaneous depletion of Rad18 and SLX4 did not induce a further increase in fork restart efficiency. Top: Schematic of the DNA fiber experiment. HeLa cells were transfected with the indicated siRNAs. Forty-eight hours after transfection, cells were incubated with 50 μM IdU for 30 min, treated with 2 mM HU and 2 μM VE-821 for 1 h, and incubated with 100 μM CldU for 30 min. Bottom: representative IdU and CldU replication tracks in cells transfected with indicated siRNAs. (**F**) Dot plot of CldU to IdU track length ratios for individual replication forks. Data represent means ± SD of three independent experiments. More than 100 fibers were analysed for each sample. n.s. indicates not significant, ****$P < 0.0001$, one-way ANOVA test. (**G**) ZRANB3-mediated fork reversal is not responsible for fork collapse in the absence of ATR under replication stress. HeLa cells were transfected with the indicated siRNAs. After 48 h of transfection, cells were treated with 2 mM HU and 2 μM VE-821 for 3 h. Cell lysates were then prepared, and western blot analysis was carried out as indicated. (**H**) ZRANB3 is not required for nascent-strand ssDNA generation at stalled forks when ATR is inhibited. Top: Schematic of the native BrdU immunofluorescence assay for nascent ssDNA detection. HeLa cells were transfected with the indicated siRNAs. After 48 h of transfection, cells were labeled with 10 μM BrdU for 15 min and then either mock-treated or treated with 2 mM HU and 2 μM VE-821. After 3 h, cells were fixed and stained with an antibody against BrdU without DNA denaturation to selectively detect nascent-strand ssDNA. Bottom: Representative BrdU foci in cells transfected with the indicated siRNAs. Scale bar, 10 μm. (**I**) Quantification of BrdU foci. Cells with more than five BrdU foci were considered positive. Each black dot in the graph represents the percentage of BrdU-positive cells in each measurement. Data represent means ± SD of three independent experiments. More than 200 cells were counted for each sample. n.s. indicates not significant, ****$P < 0.0001$, one-way ANOVA test.

© The Author(s)

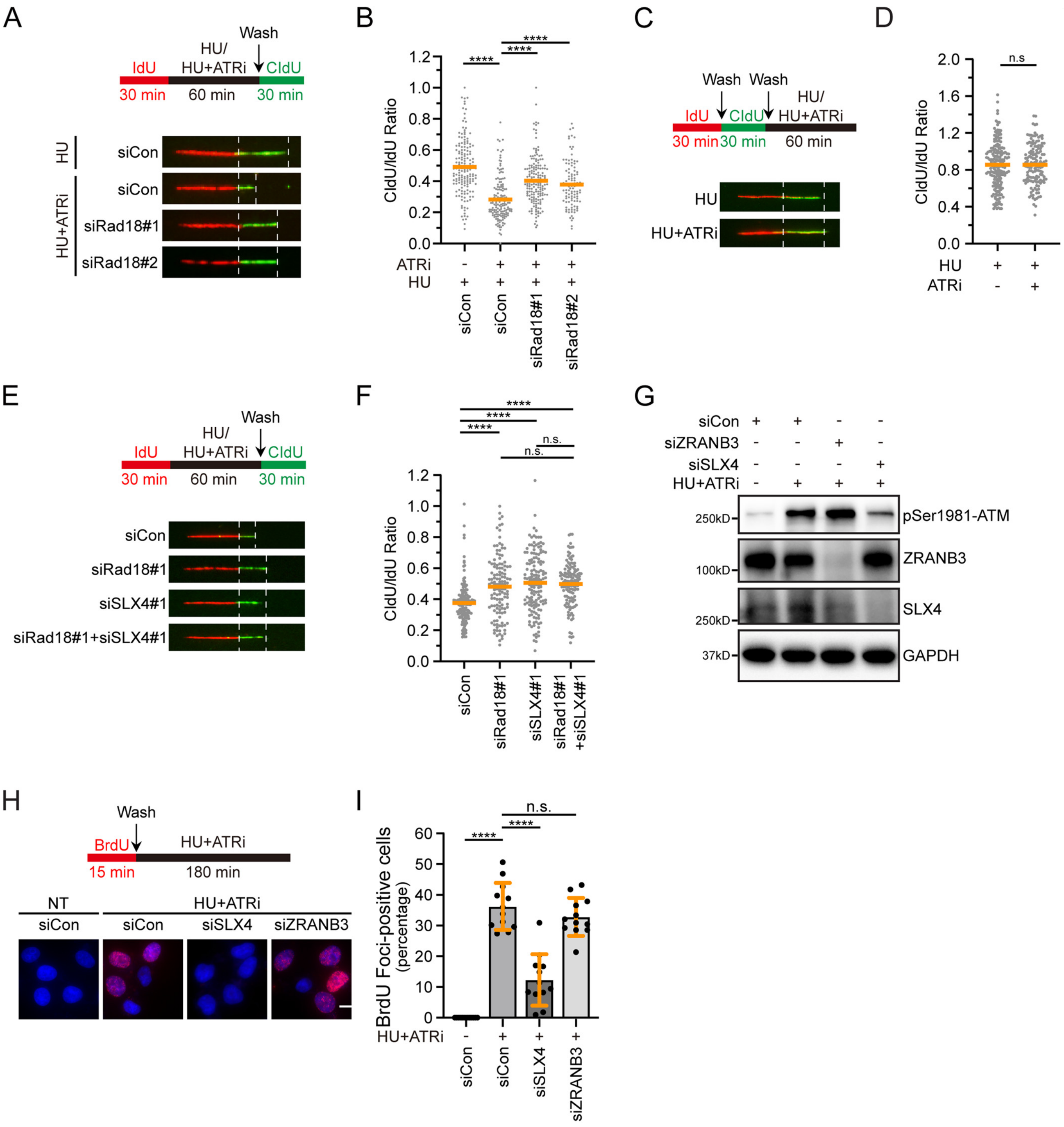
A
Wash
HU/
HU+ATRi
IdU
CldU
30 min
60 min
30 min
HU
HU+ATRi
siCon
siCon
siRad18#1
siRad18#2
B
CldU/IdU Ratio
1.2
1.0
0.8
0.6
0.4
0.2
0.0
****
****
****
ATRi
HU
siCon
siCon
siRad18#1
siRad18#2
C
Wash Wash
HU/
HU+ATRi
IdU
CldU
30 min
30 min
60 min
HU
HU+ATRi
D
CldU/IdU Ratio
2.0
1.6
1.2
0.8
0.4
0.0
n.s
HU
ATRi
E
Wash
HU/
HU+ATRi
IdU
CldU
30 min
60 min
30 min
siCon
siRad18#1
siSLX4#1
siRad18#1+siSLX4#1
F
CldU/IdU Ratio
****
****
****
n.s.
n.s.
siCon
siRad18#1
siSLX4#1
siRad18#1
+siSLX4#1
G
siCon
siZRANB3
siSLX4
HU+ATRi
250kD
100kD
250kD
37kD
pSer1981-ATM
ZRANB3
SLX4
GAPDH
H
Wash
BrdU
HU+ATRi
15 min
180 min
NT
HU+ATRi
siCon
siCon
siSLX4
siZRANB3
I
BrdU Foci-positive cells
(percentage)
60
50
40
30
20
10
0
****
****
n.s.
HU+ATRi
siCon
siCon
siSLX4
siZRANB3

 © The Author(s)

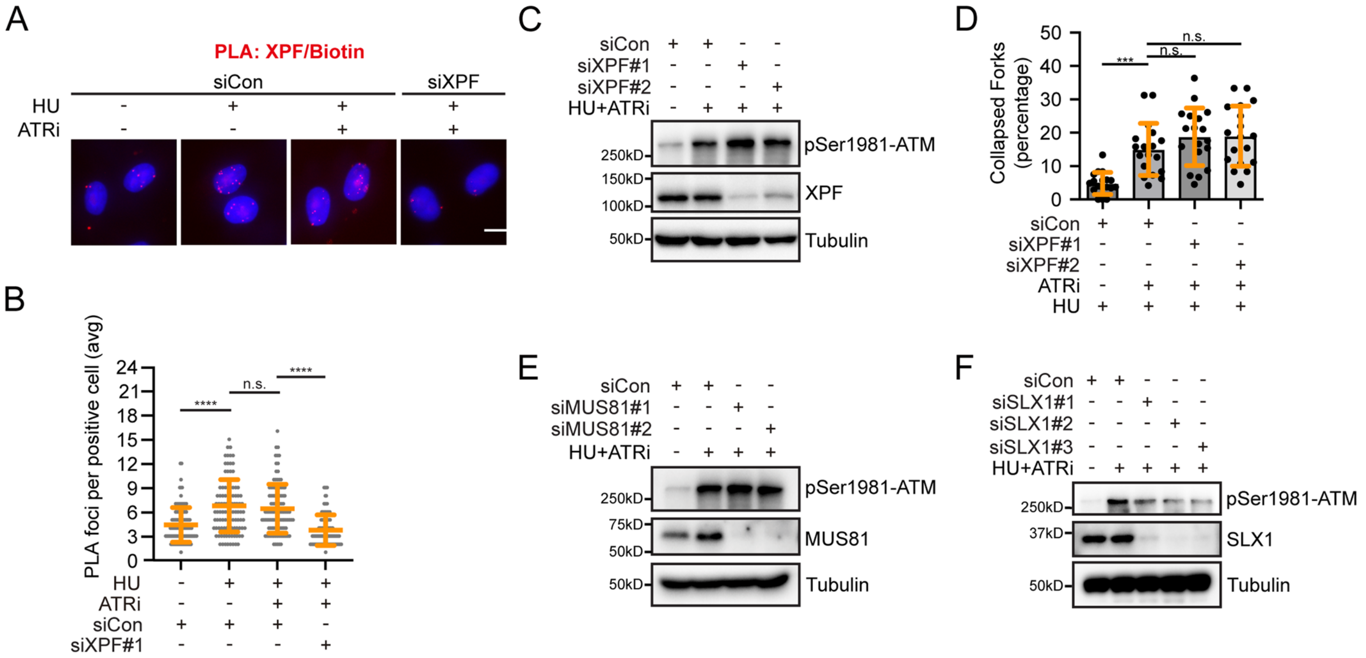

Figure EV2. SLX1 is responsible for SLX4-dependent stalled fork collapse following ATR inhibition.

(**A**, **B**) XPF accumulates at the stalled fork. HeLa cells were pulse-labeled with 10 μM EdU for 15 min, and then treated with 2 mM HU alone or in combination with 2 μM VE-821 for 1 h. Cells were then subjected to PLA with anti-XPF and anti-biotin antibodies. Representative images of PLA foci (red) (**A**). DNA was stained with DAPI. Scale bar, 10 μm. Quantification of PLA foci number per focus-positive cell (**B**). Data represent means ± SD of three independent experiments. More than 100 cells were counted for each sample. n.s., not significant, ****$P < 0.0001$, one-way ANOVA test. (**C**) HeLa cells were transfected with the siRNAs for XPF. After 48 h of transfection, cells were treated with 2 mM HU and 2 μM VE-821 for 3 h. Cell lysates were then prepared and western blot analysis was carried out as indicated. (**D**) HeLa cells were transfected with the siRNAs for XPF. After 48 h of transfection, cells were incubated with 50 μM IdU for 30 min, treated with 2 mM HU and 2 μM VE-821 for 1 h, and incubated with 100 μM CldU for 30 min. Each black dot in the graph represents the percentage of collapsed forks in each measurement, and more than 200 fibers were measured for each sample. Data represent means ± SD of three independent experiments. n.s., not significant, ***$P < 0.001$, one-way ANOVA test. (**E**) MUS81 is not responsible for the fork collapse in the absence of ATR under replication stress. HeLa cells were transfected with the siRNAs for MUS81. After 48 h of transfection, cells were treated with 2 mM HU and 2 μM VE-821 for 3 h. Cell lysates were then prepared and western blot analysis was carried out as indicated. (**F**) SLX1 plays a role in fork collapse in the absence of ATR under replication stress. HeLa cells were transfected with the siRNAs for SLX1. After 48 h of transfection, cells were treated with 2 mM HU and 2 μM VE-821 for 3 h. Cell lysates were then prepared and western blot analysis was carried out as indicated.

© The Author(s)

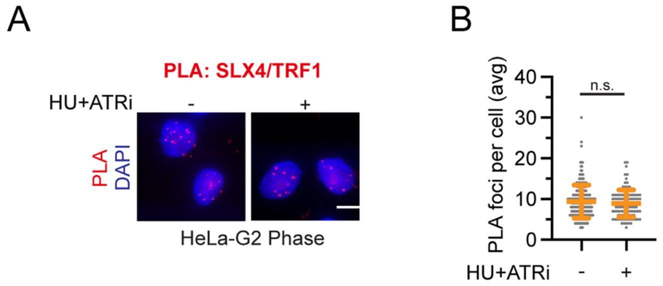

Figure EV3. ATR inhibition does not affect SLX4 accumulation at telomeres in ALT-negative HeLa cells in the G2 phase.

(**A**, **B**) For Hela cells enriched in the G2 phase, cells were treated with thymidine for 20 h, released into DMEM for 4 h, and then treated with CDK1 inhibitor, 10 μM RO-3306, for 15 h. The synchronized cells were mock-treated or treated with 2 mM HU and 2 μM VE-821 for 3 h, subjected to PLA using anti-SLX4 and anti-TRF1 antibodies. Representative images of PLA foci (**A**). Scale bar, 10 μm. Quantification of PLA foci number per focus-positive cell (**B**). Data represent means ± SD of three independent experiments. More than 100 cells were counted for each sample. n.s. indicates not significant, one-way ANOVA test.

 © The Author(s)
